# Total Synthesis of Tosyl‐Samroiyotmycin A and Its Biological Profiling

**DOI:** 10.1002/chem.202403408

**Published:** 2024-11-12

**Authors:** Benedikt Kolb, Fabian Schmid, Jessica Weng, Luca Altevogt, Ruben Pereira Rebelo, Bianca Wank, Angelika Baro, Anna Zens, Aditya Shekhar, Ursula Bilitewski, Sibylle Sax, Sergio Wittlin, Dale Taylor, Rudolf Müller, Sabine Laschat

**Affiliations:** ^1^ Institut für Organische Chemie Universität Stuttgart Pfaffenwaldring 55 D-70569 Stuttgart Germany; ^2^ AG Compound Profiling and Screening Helmholtz Zentrum für Infektionsforschung Inhoffenstr. 7 D-38124 Braunschweig Germany; ^3^ Drug Discovery and Development Centre (H3D) Department of Chemistry University of Cape Town Rondebosch 7700 South Africa; ^4^ Drug Discovery and Development Centre (H3D) Division of Clinical Pharmacology Department of Medicine University of Cape Town Observatory 7925 South Africa; ^5^ Parasite Chemotherapy, Swiss Tropical and Public Health Institute University of Basel Kreuzstr. 2, CH-4123 Allschwil Basel Switzerland

**Keywords:** Antimalarial activity, Biological profiling, Macrocycles, Macrodiolide, Natural products

## Abstract

A total synthesis of the enantiopure *syn,syn‐*tosyl‐samroiyotmycin A, a C_2_‐symmetric 20‐membered antimalarial macrodiolide with *syn,syn‐*configuration of the 8,24‐dihydroxy‐9,25‐dimethyl units and it's enantiopure *anti,anti‐*derivative is described. The synthesis was accomplished utilizing a linear approach in 7 steps and 3 % overall yield *via* a sequence of diastereoselective methylation of SuperQuat oxazolidinone auxiliary, cross metathesis and Yamaguchi macrolactonization of fully functionalized *seco*‐acids. By a similar approach we gained access to several samroiyotmycin analogues and precursors. Antimalarial activity was tested on multi‐resistant (K1) and sensitive (Nf54) *P. falciparum* strains providing insight into structure activity relationships. Both tosyl‐oxazol unit as well as the *syn*‐configuration of the two contiguous stereogenic centers turned out to be beneficial for antiplasmodial activity. For instance, *syn,syn‐*tosyl‐samroiyotmycin A showed 3.4 times higher activities than the “tosyl‐free” natural product.

## Graphical abstract

**3.4 times more active than the natural product**: The tosyl group plays an important role in the synthesis of the enantiopure tosyl‐samroiyotmycin A and its antimalarial activity. The synthetic strategy also gains access to a library of analogues. Antimalarial activity tests reveal promising results and provide insight into structure activity relationships.

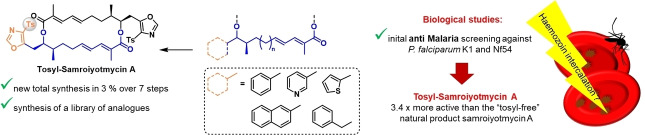

## Introduction

**Figure 1:**
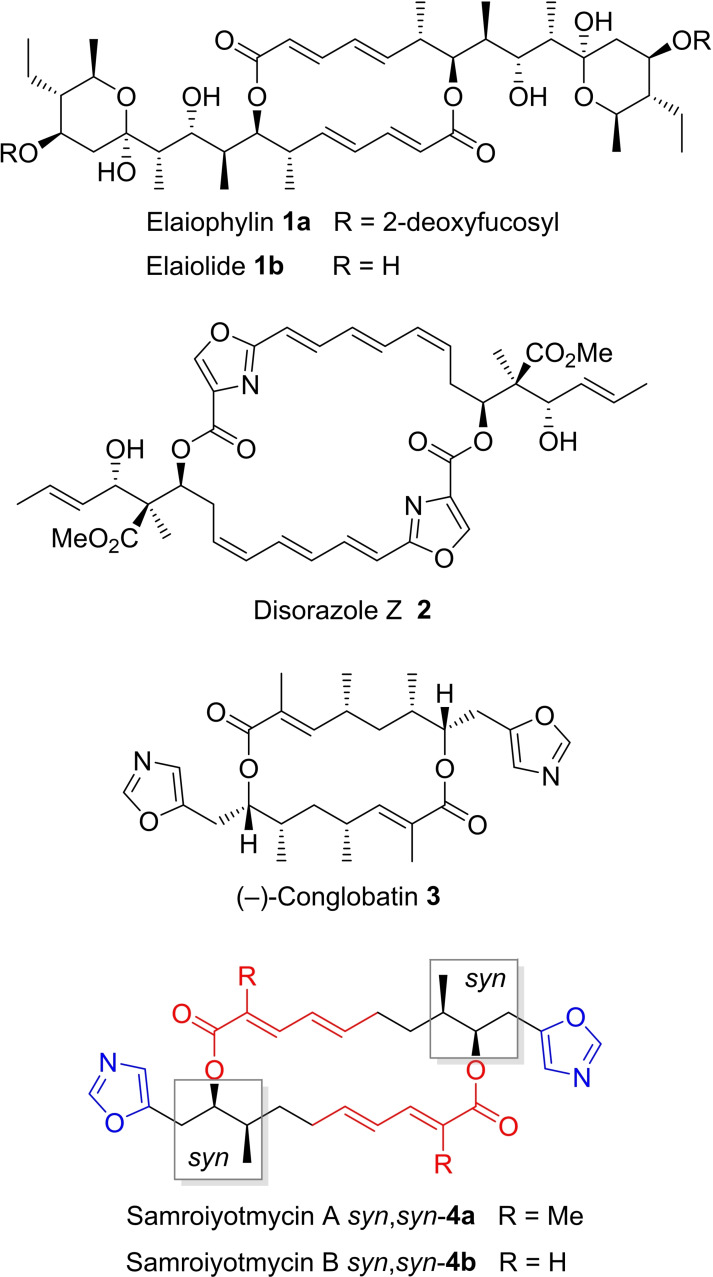
Some prominent macrodiolides **1**–**4**.

Macrodiolides constitute an important class of natural products among polyketide secondary metabolites displaying a broad range of biological activities such as inhibition of K^+^‐dependent triphosphatase,^[1]^ actin polymerization[^2^, ^3^] and heat shock protein 90 (Hsp90),[^4^, ^5^] antiproliferative activity or induction of apoptosis, which resulted in various synthetic, biosynthetic and biological studies of these compounds.^[6]^ Prominent examples are elaiophylin **1 a** and its aglycon elaiolide **1 b**, macrodiolides from *Streptomyces melanosporus*,[^7^, ^8^] disorazole Z **2** isolated from *Sorangium cellulosum*,^[9]^ or (−)‐conglobatin **3** from *Streptomyces conglobatus* ATCC 31005,[^10^, ^11^] (Figure 1). Synthetic strategies have been developed for these compounds[^12^, ^13^, ^14^, ^15^] complemented by investigations of their biosynthesis.[^16^, ^17^, ^18^] In 2013, Pittayakhajonwut and coworkers isolated two new macrodiolides samroiyotmycin A and B *syn*,*syn*‐**4 a**,**b** from *Streptomyces sp*. BCC33756 (Figure 1) and determined their structure by NMR and X‐ray crystallography.^[19]^ A biological screening revealed weak cytotoxicity and antifungal activity but promising antimalarial activity against *Plasmodium falciparum* K1.^[19]^ Key structural features of samroiyotmycin A *syn*,*syn*‐**4 a** are the *C_2_*‐symmetrical 20‐membered macrodiolide, four stereogenic centers, two 2‐methylsorbic acid moieties and two peripheral oxazole units. By *in vitro* cloning, the Leadlay group obtained the entire biosynthetic gene cluster encoding a modular polyketide synthase assembly line for conglobatin **3** and proposed that this biosynthetic pathway might also give access to *syn*,*syn*–**4 a**,**b**.^[20]^

**Scheme 1:**
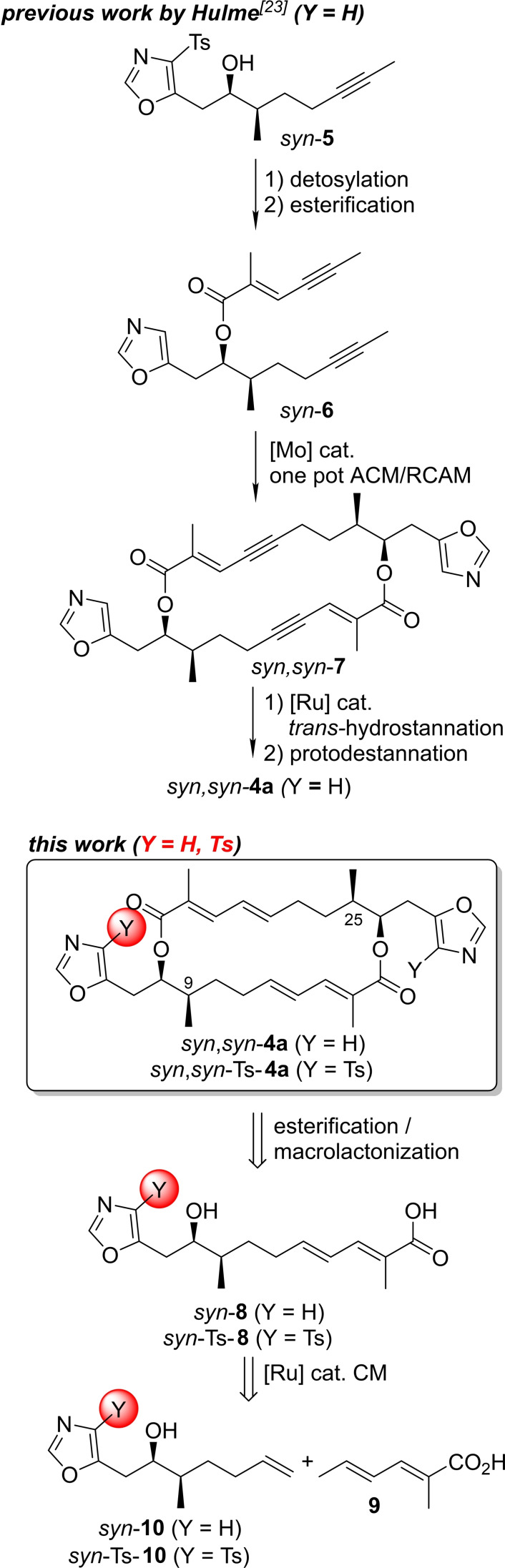
Pathways to samroiyotmycin A *syn*,*syn*–**4 a** and its tosylated analogue *syn*,*syn*–Ts–**4 a**.

The structural and biosynthetic similarities of conglobatin **3** and samroiyotmycins A, B *syn*,*syn*–**4 a**,**b** are also mirrored in the reported antimalarial activity of **3**.[^21^, ^22^] Recently, Hulme disclosed the first total synthesis of samroiyotmycin A *syn*,*syn*‐**4 a** featuring a Schöllkopf type cyclocondensation to access the tosyloxazolylalcohol *syn*‐**5**, followed by detosylation, esterification to give ester *syn*–**6** as key intermediate (Scheme 1).^[23]^ Subsequent Mo‐catalyzed alkyne cross metathesis/ring closing alkyne metathesis (ACM/RCAM) provided the macrocyclic bis‐enyne *syn*,*syn*–**7**, which was converted to the target molecule *syn*,*syn*–**4 a** *via* a delicate Ru‐catalyzed *trans*‐hydrostannation/protodestannation.

Motivated by these promising studies and the scarce biological information on samroiyotmycins, we aimed for an alternative synthetic strategy. We envisaged an esterification/macrolactonization approach of *seco*‐acid *syn*‐**8** (Scheme 1, bottom), which should be assembled *via* CM from the acid **9** and the alcohol *syn*–**10**. In addition, our aim was to access samroiyotmycin derivatives, such as *syn,syn‐*Ts‐**4 a**, in order to evaluate the biological properties in more detail and to identify structure–activity relationships (SAR). Motivated by the high medicinal relevance of Malaria and the need to develop novel treatments in light of increasing resistance,[^24^, ^25^, ^26^, ^27^, ^28^, ^29^, ^30^, ^31^, ^32^, ^33^, ^34^, ^35^, ^36^, ^37^, ^38^, ^39^] we performed a biological profiling of samroiyotmycin A *syn*,*syn*‐**4 a**, its derivatives and precursors. Our results not only revealed the crucial role of the tosyl‐protecting group in the synthesis but also for the biological properties. In addition, our study provided some hints on the pharmakophoric subunits of samroiyotmycin A *syn*,*syn*–**4 a**, relevant for its antiplasmodial activity.

## Results and Discussion

### Synthesis of Tosyl‐Samroiyotmycin

**Scheme 2:**
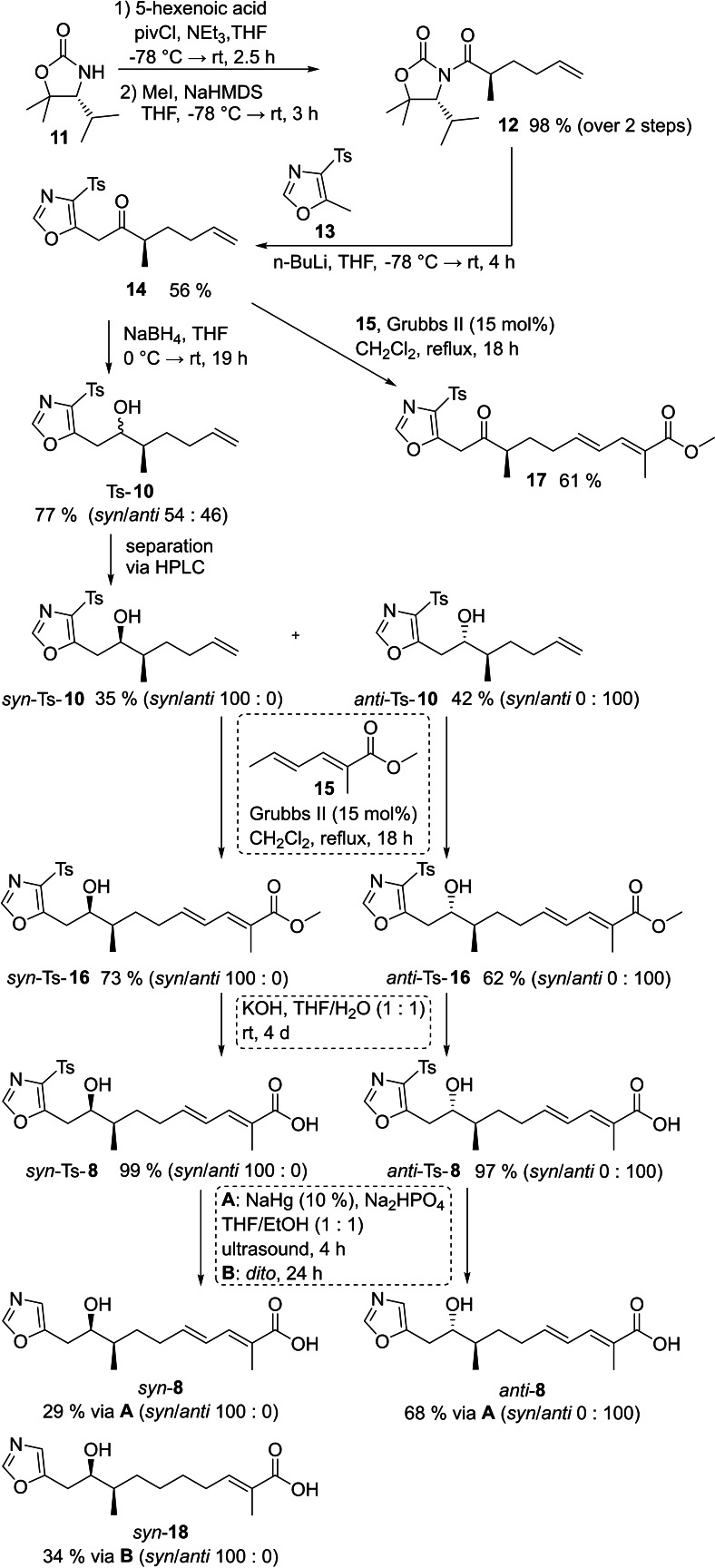
Synthetic routes to *seco*‐acids *syn*‐**8** and *anti*‐**8**.

As outlined in Scheme 2, we initially focused on the synthesis of *seco*‐acids *syn*‐**8** for further esterification/macrolactonization studies towards *syn*,*syn*‐**4 a**. To control the configuration of the methyl groups 9‐CH_3_ and 25‐CH_3_ in *syn*,*syn*‐**4 a**, we relied on the SuperQuat auxiliary **11**.[^40^, ^41^] The latter and 5‐hexenoic acid were converted to *N*‐acyloxazolidinone, followed by asymmetric methylation^[42]^ providing derivative **12** in 98 % yield (over 2 steps) as a single diastereomer. Direct treatment of *N*‐acyloxazolidinone **12** with lithiated methyltosyloxazole **13** gave ketone **14** in 56 % yield (for optimization see Table S1). Subsequent reduction of the ketone **14** with NaBH_4_ yielded Ts‐**10** in 77 % as a diastereomeric mixture (*syn*/*anti* 54 : 46). It should be noted that various attempts to install the stereogenic center at C‐2 of the tosyloxazolylalcohol Ts‐**10** failed (for details see Table S2). Thus, the diastereomeric mixture Ts‐**10** (*syn*/*anti* 59 : 41) was separated by preparative HPLC. The assignment of the relative configuration of *syn*‐Ts‐**10** and *anti*‐Ts–**10** was achieved by the modified Mosher method (for details see Table S3).[^43^, ^44^] Then the *syn*‐toxyloxazolylalcohol *syn*‐Ts‐**10** was treated with 2‐methylsorbic acid methylester **15** and 15 mol % of Grubbs II catalyst to yield *seco‐*acid methyl ester *syn*‐Ts‐**16** in 73 % (*syn*/*anti* 100 : 0) (for further optimization see Table S4), followed by saponification to give the *syn*‐tosyl‐*seco‐*acid *syn*‐Ts‐**8** in 99 % (*syn*/*anti* 100 : 0). No traces of the minor diastereomer were visible in the ^1^H‐NMR‐spectrum of the product. In a similar fashion *anti*‐Ts‐**10** was converted in 2 steps to the corresponding *anti*‐Ts‐**8** in 60 % (*syn*/*anti* 0 : 100, over 2 steps). As we aimed to investigate the influence of stereochemistry on the antiplasmodial properties, also the ketone **14** was submitted to cross metathesis with **15** to give keto methyl ester **17** in 61 %.

The removal of the C‐tosyl group was a very challenging step. Several conditions e. g. Mg in methanol,^[45]^ SmI_2_ in THF or MeOH/THF[^46^, ^47^] were tested on *seco*‐acids *syn*‐Ts‐**8**, *anti*–Ts‐**8**, the corresponding methylesters *syn*–Ts‐**16**, *anti*–Ts‐**16** as well as more simplified precursors. Only Na/Hg with Na_2_HPO_4_ in different solvents gave the desired detosylation (Scheme S1, Tables S5 ‐ S7). After considerable experimentation upon treatment of *syn*–Ts‐**8** for 4 h with 10 % Na/Hg in the presence of Na_2_HPO_4_ in THF/EtOH following a modified method by Addie,^[48]^ the desired *seco*‐acid *syn*‐**8** was isolated in 29 % (*syn*/*anti* 100 : 0) (method A, Scheme 2). Under similar conditions *anti*‐Ts‐**8** was converted to *anti*‐**8** in 68 % (*syn*/*anti* 0 : 100) respectively. However, the reaction time was identified as a critical factor. Prolonged reaction times (24 h, method B) resulted in clean reduction of the γ,δ‐C=C‐double bond and the α,β‐unsaturated acid *syn*‐**18** was isolated in 34 % as crude product.

The attempted macrolactonization of *seco*‐acids turned out to be highly capricious. Several methods were investigated. For example, in preliminary experiments using a (1 : 1) mixture of *syn*‐Ts‐**8** and *anti*‐Ts‐**8** under Collins method^[49]^ with Hf(OTf)_4_ (10 mol %) provided up to 20 % of a complex mixture of *syn*,*syn*‐Ts‐**4 a**, *syn*,*anti*‐Ts‐**4 a** and *anti*,*anti*‐Ts‐**4 a** which could not be separated (for stereochemical details see Scheme S2 and Table S8).

However, attempts to isolate the desired product in pure form, unfortunately, failed. Other methods such as Mitsunobu,[^50^, ^51^] Steglich conditions,^[52]^ TsCl/pyridine,^[53]^ or Shiina's method^[54]^ did not provide any trace of the macrocyclic product (Table S8). Next, a Yamaguchi methodology was tested (Table 1; for initial experiments see Table S8).^[55]^

**Table 1:** Macrolactonization of *seco*‐acids under various conditions.

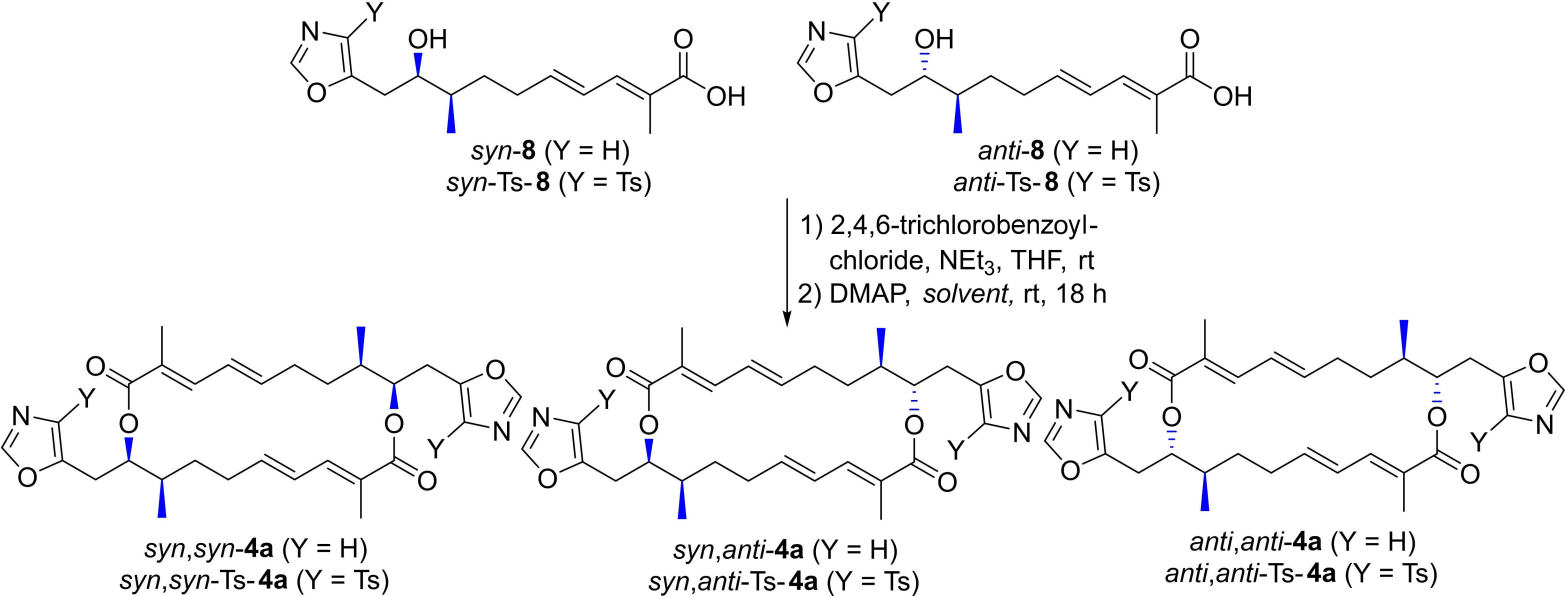						
entry	starting material	dr (*syn* : *anti*)	solvent	product	yield	dr (*syn*,*syn* : *syn*,*anti* : *anti*,*anti*)
1	**8**	56 : 44	benzene	**4 a** ^[a]^	(9 %) ^[b]^	n.d.^[c]^
2	*syn*‐**8**	100 : 0^[d]^	benzene	*syn,syn*‐**4 a**	traces	
3	*anti*‐**8**	0 : 100^[d]^	benzene	*anti,anti*‐**4 a**	traces	
4	Ts‐**8**	54 : 46	DMF	Ts‐**4 a** ^[a]^	22 %	n.d.^[c]^
5	Ts‐**8**	54 : 46	benzene	Ts‐**4 a** ^[a]^	29 %	n.d.^[c]^
6	*syn*‐Ts‐**8**	100 : 0^[d]^	benzene	*syn,syn*‐Ts‐**4 a**	20 %	100 : 0 : 0^[e]^
7	*anti*‐Ts‐**8**	0 : 100^[d]^	benzene	*anti,anti*‐Ts‐**4 a**	23 %	0 : 0 : 100^[e]^

[a] mixture of *syn*,*syn*‐/*syn*,*anti*‐/*anti,anti*‐products (see Scheme S2); [b] crude product (contains impurities despite repetitive HPLC purifications); [c] due to overlapping ^1^H NMR signals, determination of dr not possible; [d] according to NMR, no trace of minor diastereomer was visible; [e] according to NMR, no trace of minor diastereomers were visible.

In preliminary experiments the diastereomeric mixture of *seco*‐acids **8** (*syn/anti* 56 : 44) was submitted to Yamaguchi lactonization with 2,4,6‐trichlorobenzoylchloride in THF followed by treatment of the mixed anhydrides with DMAP in benzene yielding macrodiolide **4 a** as a mixture of *syn*,*syn*‐**4 a**, *syn*,*anti*‐**4 a** and *anti*,*anti*‐**4 a** (Scheme S2) in only 9 % (Table 1, entry 1). Unfortunately, from this mixture no pure diastereomer could be isolated in pure form despite multiple HPLC runs. However, the diastereomeric pure *syn*–**8** and *anti*‐**8** without the tosyl‐group respectively gave only traces of the corresponding macrodiolides *syn*,*syn*‐**4 a** and *anti*,*anti*‐**4 a** (according to ESI‐MS) (entry 2, 3). Due to difficulties in accomplishing the desired macrocyclization with the diastereomeric pure and unprotected *seco*‐acids **8**, we focused on the cyclization of tosyl‐protected *seco‐*acids Ts‐**8**. Gratifyingly, Yamaguchi lactonization of the diastereomeric mixture Ts‐**8** (*syn*/*anti* 54 : 46) in DMF yielded 22 % of macrodiolides Ts‐**4 a** (entry 4). When the reaction was performed in benzene, the yield was slightly improved to 29 % (entry 5). Next, the pure diastereomers *syn*‐Ts‐**8** and *anti*‐Ts‐**8** respectively were employed in parallel runs in the Yamaguchi esterification,^[56]^ giving the *syn*,*syn‐*macrodiolide *syn*,*syn*‐Ts‐**4 a** in 20 % (dr 100 : 0) and the *anti*,*anti*‐macrodiolide *anti*,*anti*‐Ts‐**4 a** in 23 % (dr 0 : 100) respectively (entry 6, 7). No traces of the minor diastereomers were visible in the ^1^H‐NMR‐spectrum of the product. Unfortunately, any attempts to remove the tosyl‐protecting group with the Na/Hg method met with little success. For example, treatment of *syn*,*syn*‐Ts‐**4 a** with Na/Hg under the above discussed conditions yielded only decomposition of the starting material. (For an alternative attempt towards samroiyotmycin A *syn*,*syn*‐**4 a** via sequential esterification and cross metathesis see Scheme S3).

**Scheme 3:**
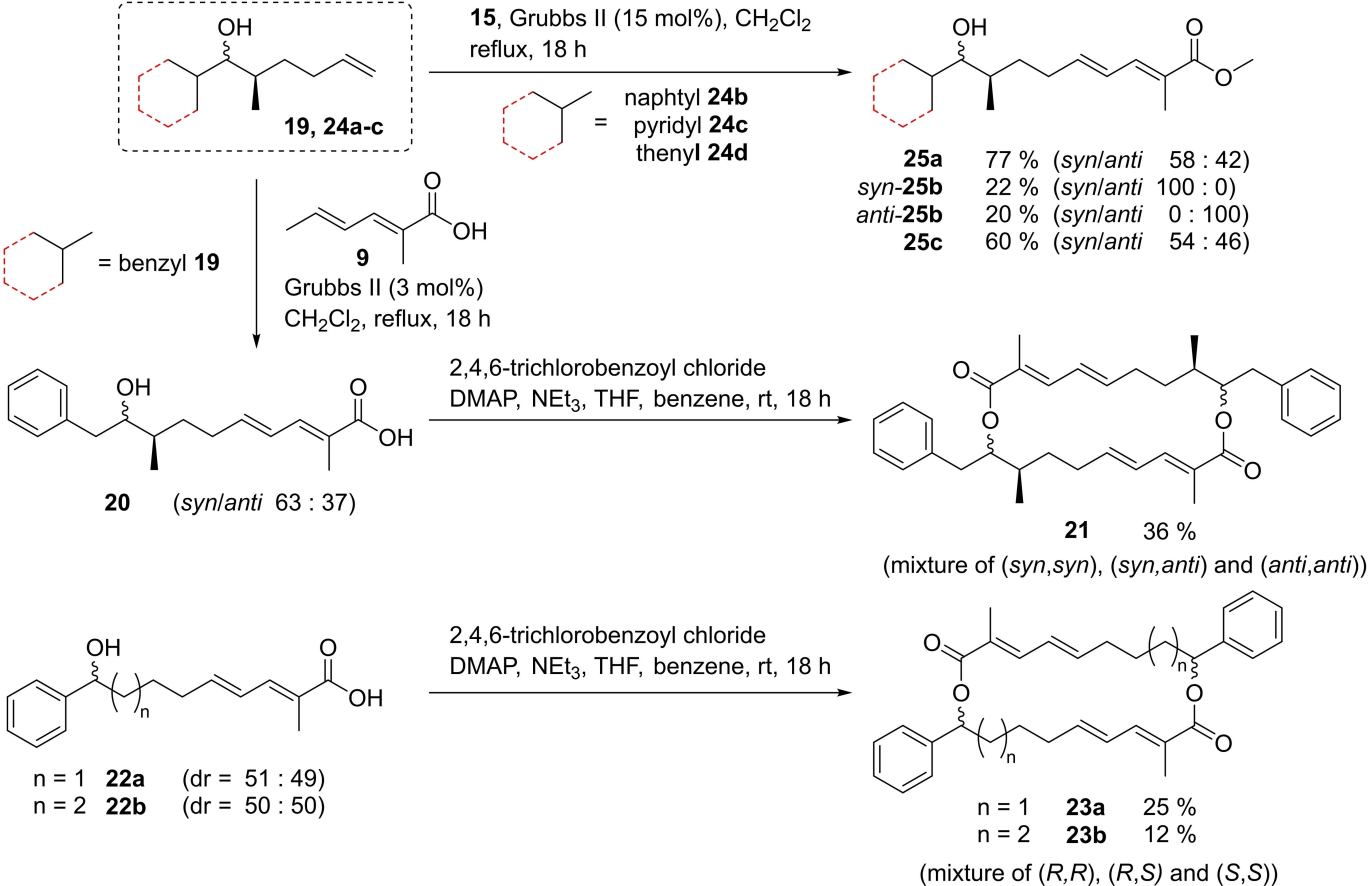
Synthesis of samroiyotmycin derivatives and heteroaryl analogues (for further details see Scheme S4, S5).

Furthermore, the synthetic route towards *syn*,*syn*‐Ts‐**4 a** and *anti*,*anti*‐Ts‐**4 a** was applied further to get access to samroiyotmycin derivatives carrying an aromatic or heteroaromatic unit instead of the oxazol unit. Scheme 3 shows selected examples such as the structurally related *seco*‐acids **20**, **22 a,b** and methyl esters **25**. Macrolactonization under the conditions discussed above provided the macrodiolides **21** and **23 a,b**. For details on the synthesis of the samroiyotmycin derivatives see Scheme S4.

### Biological Studies of Tosyl‐Samroiyotmycin and Several Precursors

With the samroiyotmycin derivatives *syn,syn*–Ts–**4 a**, *anti,anti*‐Ts‐**4 a**, precursors and analogues in hand, we aimed to get some insight into the structure‐activity relationships (SAR). Initial biological activity tests were carried out in three series. First cytotoxicity and antimicrobial activities were examined to rule out any cross reactivity, followed by antimalarial tests.

Cytotoxicity was examined using the L929 mouse fibroblast cell line and determining cell viability assay using resazurin reagent of the Alamar Blue reagent^[57]^. For details and a full listing of all tested compounds and biological experiments see Table S9). This initial screening resulted in heat maps (Figure S2), from which the most active candidates were identified and submitted to dose‐dependent tests giving IC_50_ values. In a similar manner, the antimicrobial activity was assessed against the TolC deletion mutant of the Gram‐negative bacterium *Escherichia coli* and the Gram‐positive bacterium *Staphylococcus aureus* via an adjusted broth microdilution method.^[58]^ Subsequent dose‐dependent experiments gave IC_50_ values. Only the tosyl‐protected oxazolylketone **14** showed weak cytotoxic and antimicrobial activity, whereas neither macrodiolides *syn,syn*‐Ts‐**4 a** and *anti,anti*‐Ts‐**4 a** nor the tosyl‐protected precursors and analogues showed any activity in these tests (for details see Table S9), which is in line with Pittayakhajonwuts's report.^[19]^

Antiplasmodial activity was studied by 3H‐hypoxanthine incorporation assay (Table 2).[^59^, ^60^] First, the antiplasmodial activity against the *Plasmodium falciparum* NF54 strain was examined, which revealed some surprising SAR results. Selected members of the series were employed in tests against the multi drug resistant *Plasmodium falciparum* K1 strain and compared with the reported data^[19]^ and a side‐by‐side assay of natural *syn,syn*‐**4 a** ^[61]^ and synthetic tosylated *syn,syn*‐Ts‐**4 a** and *anti,anti*‐Ts‐**4 a**.

**Table 2:** Antimalarial activity of samroiyotmycins **4 a** and precursors against *Plasmodium falciparum* NF54 (sensitive) and K1 (multi drug resistant).

entry	compound	antiplasmodial activity (*P. falciparum*)	
		IC_50_ [μM] (NF54)^[b]^	IC_50_ [μM] (K1)^[b]^
1	samroiyotmycin A *syn,syn*‐**4 a** ^[a]^	0.77	0.51^[d]^
2	*syn*‐Ts‐**8**	13.47	‐^[c]^
3	*anti*‐Ts‐**8**	17.31	‐^[c]^
4	*syn*‐Ts‐**16**	0.94	0.95
5	*anti*‐Ts‐**16**	2.44	‐^[c]^
6	**17**	2.85	‐^[c]^
7	*syn,syn*‐Ts‐**4 a**	0.17	0.15
8	*anti,anti*‐Ts‐**4 a**	0.26	0.25
9	chloroquine	0.0075	0.27
10	artesunate	0.0064	0.0095

[a] isolated natural product^[62]^; [b] tested at Swiss TPH using the 72 h [3H] hypoxanthine incorporation assay. The experiments were performed 2x (biological replicates) and the individual IC_50_ values varied by less than 50 %; [c] not determined; [d] reported data from Lit.^[19]^=6.98 μM determined by [3H] hypoxanthine incorporation assay^[19]^; for further details see Table S9.

The results in Table 2 revealed that tosyl‐protected *seco*‐acids *syn*‐Ts‐**8**, *anti*‐Ts‐**8** were 18 x and 23 x respectively less active than the natural product *syn,syn*‐**4 a** (entries 1–3). The antiplasmodial activity was significantly improved by conversion of the *seco*‐acids into the corresponding methylesters *syn*‐Ts‐**16**, *anti*‐Ts‐**16** (entries 4, 5). Compound *syn*‐Ts‐**16** already possessed a similar activity as compared to *syn,syn*‐**4 a** (entry 1). The keto methylester **17** carrying only the (*R*)‐methyl group at C‐8 gave a similar IC_50_ value as compared to the methylester *anti*‐Ts‐**16** with both alcohol and methyl stereogenic centers present (entries 5, 6). Much to our surprise the tosyl‐protected samroiyotmycin A diastereomers displayed significantly lower IC_50_ values (*syn,syn*‐Ts‐**4 a** 0.17 μM (NF54), 0.15 μM (K1); *anti,anti*‐Ts‐**4 a** 0.26 μM (NF54), 0.25 μM (K1); entries 6, 7). In other words, *syn,syn*‐Ts‐**4 a** was 3.4 x more active against the multi‐resistant strain *P. falciparum* K1 as compared to the unprotected natural product *syn,syn*‐**4 a** (*syn,syn*‐Ts‐**4 a** 0.15 μM (K1); *syn,syn*‐**4 a** 0.51 μM (K1)). Even the diastereomeric tosyl‐protected macrodiolide *anti,anti*‐Ts‐**4 a** showed a 2× higher antiplasmodial activity. It should be emphasized that macrodiolides *syn,syn*‐Ts‐**4 a**, *anti,anti*‐Ts‐**4 a** as well as methylester *syn*‐Ts‐**11** do not suffer from parasite resistance and remain equally active against both sensitive NF54 and multi‐resistant K1 strain. In addition, the relative configuration also contributed to the antiplasmodial activity. In general, the *syn*‐diastereomers always showed somewhat lower IC_50_‐values, than the *anti‐*diastereomers.

To further elaborate the role of the oxazole units, dienoate and stereogenic centers in samroiyotmycin A *syn,syn*‐**4 a** on the antimalarial activity, biological studies of samroiyotmycin derivatives, precursors and heteroaryl analogues (of Scheme 3) were performed (for details and a full listing of all tested compounds and biological experiments see Table S9). Neither analogues carrying phenyl, benzyl or other heteroaryl unit instead of oxazole nor the corresponding *seco*‐acids showed any cytotoxic or antimicrobial activity. The antiplasmodial tests of these derivatives against NF54 strain revealed only moderate activity with IC_50_ values in the range of 6–40 μM. Macrodiolides **21**, **23** with lateral benzyl or phenyl ring were much less active (IC_50_ >10 μM) as compared to *syn,syn*‐Ts‐**4 a**, supporting the importance of the tosyloxazole unit for the antiplasmodial activity. Within the series of acyclic precursors **20**, **22**, **25** the 2‐thienyl‐substituted methylester **25 c** showed the lowest IC_50_ value (3.50 μM), whereas the IC_50_ values of the other acyclic derivatives were up to one order of magnitude higher. The improved activity of the *syn*‐ over the *anti*‐diastereomer was also observed for 2‐pyridyl‐substituted methylesters *syn*‐**25 b** (IC_50_ 14.65 μM) vs *anti*‐**25 b** (IC_50_ 24.13 μM) (Table S9).

The antiplasmodial results of samroiyotmycin derivatives and analogues were further compared with the natural product (−)‐borrelidin, a macrolide with known antimalarial activity, and previously synthesized borrelidin fragments (for details see Table S10). Although (−)‐borrelidin as well as several fragments carrying a (halo)cyanodiene unit showed promising antiplasmodial activity, these compounds possessed additional competing cytotoxicity and/or antimicrobial activity, which limits their medicinal applications.

In order to rationalize the unexpected increase of the antiplasmodial activity of *syn,syn*‐Ts‐**4 a** as compared to the “tosyl‐free” samroiyotmycin A *syn,syn*‐**4 a** qualitative molecular docking studies with hemozoin (*β*–hematin) dimer using YASARA Structure (Version 24.4.10)[^63^, ^64^, ^65^] were performed. The known X‐ray crystal structure data of *syn,syn*‐**4 a** ^[19]^ and hemozoin (*β*–hematin)^[66]^ were downloaded from the CCSC data base and used as input structures (for details see Figures S3, S4). Comparison of *syn,syn*‐**4 a**/hemozoin dimer complex with *syn,syn*‐Ts‐**4 a**/hemozoin dimer complex revealed, that in the former case the oxazole unit of the samroiyotmycin A was positioned parallel over one pyrrol ring of the heme unit enabling π‐π‐interactions. In contrast, in the *syn,syn*‐Ts‐**4 a**/hemozoin dimer complex the tosyl moiety is stacked on top of the pyrrole ring instead, thus resulting in improved donor‐acceptor interactions. Although these considerations have to be treated with great care and other modes of action might occur as well, such a working hypothesis might be helpful for developing novel lead structures.

## Conclusions

A total synthesis of the enantiopure *syn,syn*‐tosyl‐samroiyotmycin A *syn,syn*‐Ts‐**4 a** and it's diastereomer *anti,anti*‐Ts‐**4 a** was accomplished in 7 steps and 3 % overall yield. Key steps were a diastereoselective methylation of SuperQuat oxazolidinone auxiliary, cross metathesis and Yamaguchi esterification/macrolactonization. The synthetic strategy was also amenable to related macrodiolides **21**, **23 a**, **23 b** carrying phenyl or benzyl rings instead of the oxazole moiety and different ring sizes as well as acyclic *seco*‐acids and precursors. The observed reluctance of the C‐tosyl group to undergo reductive deprotection turned into a fortune, as the biological investigations revealed a remarkable antiplasmodial activity of the final macrodiolides *syn,syn*‐Ts‐**4 a** and *anti,anti*‐Ts‐**4 a**, which are 3.4 x and 2 x more active than the “tosyl‐free” natural product samroiyotmycin A *syn,syn*‐**4 a**. SAR studies with samroiyotmycin derivatives, precursors and heteroaryl analogues showed that the *syn*‐configuration led to lower IC_50_ values as compared to *anti*‐configurated diastereomers. Qualitative docking studies suggested hemozoin intercalation by the samroiyotmycin derivatives. In a more general context, our results might serve as a hint, that “semifinal”, i. e. protected precursors of natural products obtained during total synthesis might be valuable pharmaceutical lead structures.

## Author Contributions

B.K., F.S., J.W., R.P.R. and B.W. synthesized and characterized the samroiyotmycin derivatives, precursours and heteroaryl analogues. A.B., A.Z. and B.K. checked the data and wrote the manuscript. L.A. and A.S. performed the cytotoxcity and antimicrobial assays. U.B. supervised the cytotoxicity and antimicrobial assays. F.S., S.S., S.W. and D.T. performed the antiplasmodial assays. S.W. and D.T. supervised the antiplasmodial assays. R.M. provided his expertise on medicinal chemistry. S.L. wrote the manuscript, supervised and coordinated the research. All authors proofread the manuscript and agreed to the final version.

## Conflict of Interests

The authors declare no competing financial interest.

1

## Supporting information

As a service to our authors and readers, this journal provides supporting information supplied by the authors. Such materials are peer reviewed and may be re‐organized for online delivery, but are not copy‐edited or typeset. Technical support issues arising from supporting information (other than missing files) should be addressed to the authors.

Supporting Information

## Data Availability

The data that support the findings of this study are available in the supplementary material of this article.
