# Supplementary material for: Total Synthesis of Tosyl‐Samroiyotmycin A and Its Biological Profiling
Source: Chemistry. 2024 Nov 12;30(71):e202403408. doi: 10.1002/chem.202403408 (PMC11653242; doi:10.1002/chem.202403408)

Chemistry–A European Journal

Supporting Information

Total Synthesis of Tosyl-Samroiyotmycin A and Its Biological Profiling

Benedikt Kolb, Fabian Schmid, Jessica Weng, Luca Altevogt, Ruben Pereira Rebelo, Bianca Wank, Angelika Baro, Anna Zens,* Aditya Shekhar, Ursula Bilitewski,* Sibylle Sax, Sergio Wittlin,* Dale Taylor, Rudolf Müller,* and Sabine Laschat*

Supporting Information

Total Synthesis of Tosyl-Samroiyotmycin A and Its Biological Profiling

Benedikt Kolb,†[a] Fabian Schmid,†[a] Jessica Weng,[a] Luca Altevogt,[a] Ruben Pereira Rebelo,[a] Bianca Wank,[a] Angelika Baro,[a] Anna Zens,[a]* Aditya Shekhar,[b] Ursula Bilitewski,[b]* Sibylle Sax,[e] Sergio Wittlin,[e]* Dale Taylor,[c] Rudolf Müller,[c]* and Sabine Laschat[a]*

[a] B. Kolb, Dr. F. Schmid, J. Weng, L. Altevogt, R. P. Rebelo, B. Wank, Dr. A. Baro, Dr. A. Zens, Prof. S. Laschat

Institut für Organische Chemie, Universität Stuttgart, Pfaffenwaldring 55, D-70569 Stuttgart, Germany
E-Mail: sabine.laschat@oc.uni-stuttgart.de

[b] Dr. A. Shekhar, Prof. U. Bilitewski

AG Compound Profiling and Screening, Helmholtz Zentrum für Infektionsforschung, Inhoffenstr. 7, D-38124 Braunschweig, Germany
E-Mail: ursula.bilitewski@helmholtz-hzi.de

[c] Dr. D. Taylor, Dr. R. Müller

Drug Discovery and Development Centre (H3D), Department of Chemistry, University of Cape Town, Rondebosch 7700, South Africa
E-Mail: rudolfmueller@yahoo.com

[d] Dr. D. Taylor

Drug Discovery and Development Centre (H3D), Division of Clinical Pharmacology, Department of Medicine, University of Cape Town, Observatory. 7925, South Africa
E-Mail: dale.taylor@uct.ac.za

[e] Sibylle Sax, PD Dr. Sergio Wittlin

Parasite Chemotherapy, Swiss Tropical and Public Health Institute,
Kreuzstr. 2, CH-4123 Allschwil, Switzerland, University of Basel, Basel, Switzerland
E-Mail: sergio.wittlin@swisstph.ch

Table of Contents

1 Optimization of the addition reaction of oxazolidinon **12** and oxazole **13** S4

2 Investigation of the stereoselective synthesis of *sec*-alcohol *syn*-Ts-**10** S4

3 Determination of the configuration of Ts-**10** S5

4 Optimization of the cross coupling reaction S6

5 Detosylation reactions S6

5.1 Investigation on detosylation of tosyl-oxazole Ts-**S5** S6

5.2 Investigation on detosylation of ester protected tosyl-oxazoles S7

5.3 Investigations on detosylation of tosyl-oxazole Ts-**10** S7

5.4 Investigations on detosylation of Ts-**8** and Ts-**16** S8

6 Macrolactonization of *seco*-acids towards the macrodiolides S9

6.1 Stereochemical details S9

6.2 Optimization of the Macrolactonization S10

7 Alternative Approach towards samroiyotmycin A **4a** S11

8 Synthesis of aromatic or heteroaromatic samroiyotmycin-derivatives S12

9 Further investigations on macrolactonization towards **4a** S14

10 ^{1}H NMR stacked plots S16

11 Biological investigations S17

11.1 Antimalarial in vitro activity against asexual blood stages of *Plasmodium falciparum* NF54 and K1 at Swiss TPH S17

11.2 Antimalarial in vitro activity against asexual blood stages of *Plasmodium falciparum* NF54 at H3D S17

11.3 Antibacterial and cytotoxic activity at HZI S18

11.3.1 Test for antibacterial activity S18

11.3.2 Test for Cytotoxicity S18

11.3.3 Summary of antibacterial and cytotoxic activity S19

11.4 Summary of the biological results S20

12 Molecular Docking with Yasara Structure S30

13 Experimental Details S32

13.1 General Information S32

13.2 General Procedures S32

13.3 Synthesis of tosylated oxazole precursors S34

13.4 Synthesis of samroiyotmycin derivatives S51

13.5 Synthesis of Superquat-Auxiliary **11** S66
13.6 Synthesis of sorbic acid **9** S68
13.7 Compounds for optimization reactions S69
13.8 Synthesis of Mosher esters S74
14 Literature S77
15 NMR and MS spectra S79

1 Optimization of the addition reaction of oxazolidinon (12) and oxazole (13)

Table S1: Optimization of the oxazole coupling to ketone **14**.

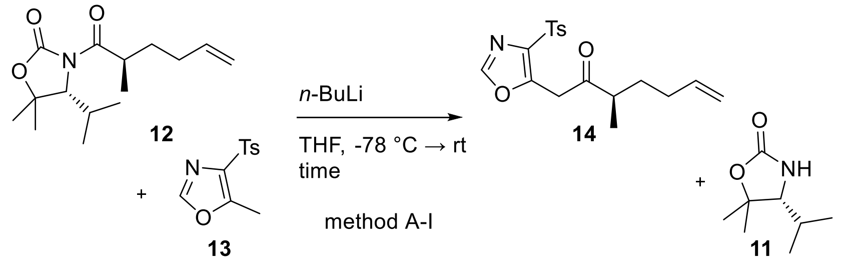

method	time at -78 °C [h]	time at rt [h]	yield **14** [%]	yield **11** [%]	yield **12**[c] [%]
A	1	2	8	60	0
B	1	0.1	23	55	29
C	1	0.5	24	-[a]	0
D	1	18	18	73	0
E	2	0.5	27	42	20
F	2	0.75	35	41	54
G	2	1	57	58	36
H	2	1.5	70	65	27
I	2	1.5[b]	80	79	15

[a] not determined; [b] warm up over night; [c] reisolated.

2 Investigation of the stereoselective synthesis of *sec*-alcohol (*syn*-Ts-10)

Table S2: Investigation of the stereoselective synthesis of *syn*-Ts-**10** starting from **14**.

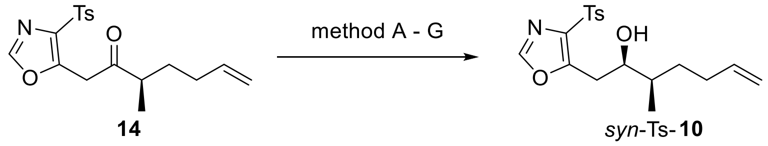

method	conditions	yield [%]
A	L-selectride, THF, -78 °C, 2 h	0[a]
B	(*R*)-Me-CBS (10 mol%), BH_3, THF, 25 °C, 30 min	0[a]
C	RuCl[(*S*,*S*-TsDPEN)](*p*-cymene) (10 mol%), HCO_2Na, H_2O, DMF, 40 °C → 50 °C, 90 h	0[b]
D	1) RuCl[(*S*,*S*-TsDPEN)](*p*-cymene) (1.5 mol%), KOH, H_2O, CH_2Cl_2, rt, 5 min<br>2) HCOOH, NEt_3, CH_2Cl_2, 40 °C, 96 h	0[b]
E	1) RuCl[(*S*,*S*-TsDPEN)](*p*-cymene) (1.5 mol%), KOH, H_2O, CH_2Cl_2, rt, 5 min<br>2) $NaHCO_2$, H_2O, CH_2Cl_2, 40 °C, 90 h	0[b]
F	1) RuCl[(*S*,*S*-TsDPEN)](*p*-cymene) (0.5 mol%), KOH, H_2O, CH_2Cl_2, rt, 5 min<br>2) 2-propanol, 30 °C, 168 h	0[b]
G	1) (1*R*)-(+)-α-pinene, 9-Borabicyclo[3.3.1]nonae, THF, 65 °C, 6 h	0

[a] decomposition of the starting material; [b] reisolation of the starting material

Furthermore, an enzymatic approach to separate the diastereomeric mixture *syn*-Ts-**10**, *anti*-Ts-**10** was tested. Unfortunately, the lipase catalysed stereoselective transesterification with various solvents ant different reaction times failed.

3 Determination of the configuration of (Ts-10)

Esterification of diastereomers Ts-**10** with Mosher acid chlorides MTPACl yielded esters (*R*)-**S1** and (*S*)-**S1.** The synthesis of the respective MTPACls was proceeded beforehand according to Hoye[1] starting from (*R*)- and (*S*)-mosher acids (Table S3).

Table S3: Determination of the absolute configuration of *anti*-Ts-**10** by Mosher's method.

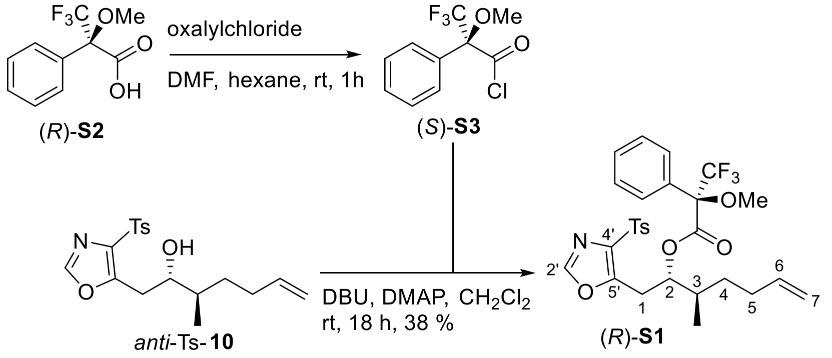

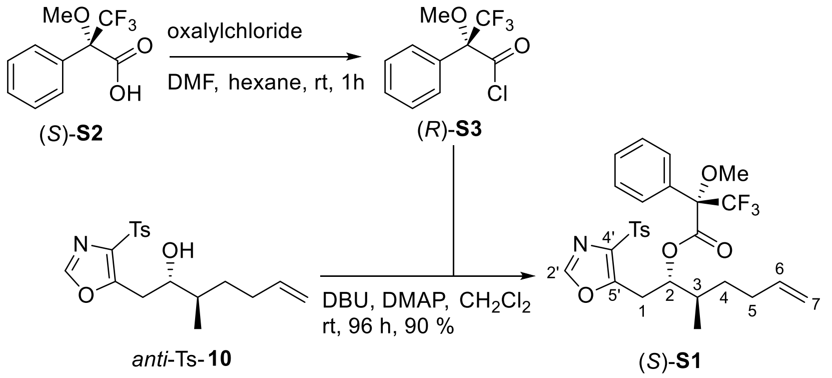

proton	*δ* ((*S*)-**S1**) [ppm]	*δ* ((*R*)-**S1**) [ppm]	Δ*δ* = *δ*(*S*)-*δ*(*R*) [ppm]	Hz [500 MHz]
2'-H	7.73	7.61	-0.24	-120
1-HA	3.29	3.34	-0.14	-70
1-HB	3.45	3.45	0.00	0
2-H	5.48	5.48	0.00	0
3-H	2.00	1.96	0.04	20
3Me-H	1.03	0.93	0.10	50
4-HA	1.33	1.28	0.05	25
4-HB	1.64	1.59	0.05	25
5-HA	2.11	2.07	0.04	20
5-HB	2.22	2.19	0.03	15
6-H	5.80	5.77	0.03	15
7-HA	5.00	4.99	0.01	5
7-HB	5.06	5.04	0.02	10

4 Optimization of the cross coupling reaction

Table S4: Investigation of the Grubbs II-catalyzed cross coupling.

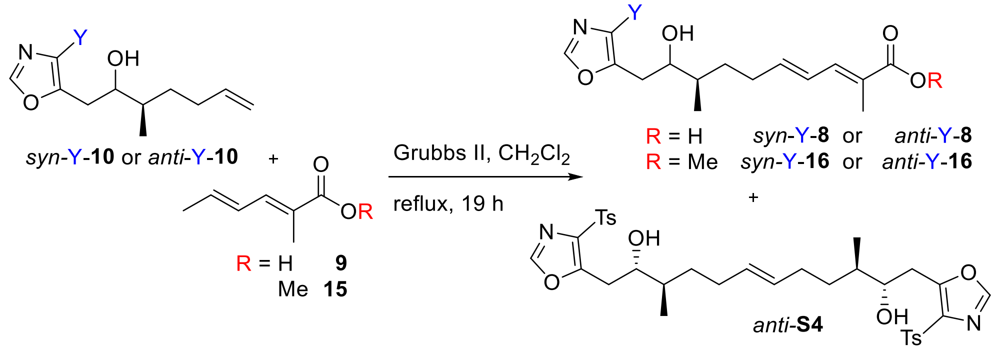

method	R	Y	catalyst [mol%]	**9** or **15** [eq]	yield *syn*-Y-**16** or *anti*-Y-**16** [%]	yield *syn*-Y-**8** or *anti*-Y-**8** [%]	yield dimer *anti*-**S4** [%]
A	H	Ts	1	1	0	0	0
B	H	H	5	2	0	0	0
C	H	Ts	1	2	0	0	0
D	H	Ts	3	2	0	0	0
E	H	Ts	15	2	0	5	3
f	Me	Ts	15	2	73	62	0

5 Detosylation reactions

5.1 Investigation on detosylation of tosyl-oxazole (Ts-S5)

Table S5: First investigation of the detosylation reaction with simplified tosyl derivative Ts-**S5**.

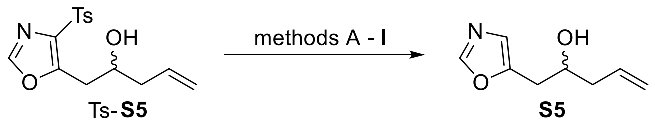

method	reactants (eq.)	solvent	temperature	time [h]	yield [%]	conversion [%]
A	Mg (5)	MeOH	0 °C	1	0	0
B	Mg (5)	MeOH	10 °C	1	0	0
C	Mg (15)	MeOH	0 °C→rt	18	<10[a]	100
D	Mg (15)	MeOH	50 °C	0.5	18	100
E	KO*t*Bu (5)	MeOH	rt	18	0	100
F	$LiNHC_4H_9$ (10)[b]	THF	-78 °C→rt	4	0	100
G	Mg (15)	MeOH	rt	1	33	100
H	Mg (10)	MeOH	rt	1	30	100
I	Mg (5+2.5)	MeOH	rt	4	38	100

[a] according to ^1H NMR; [b] synthesized from *n*-BuLi and *n*-butylamine.

5.2 Investigation on detosylation of ester protected tosyl-oxazoles

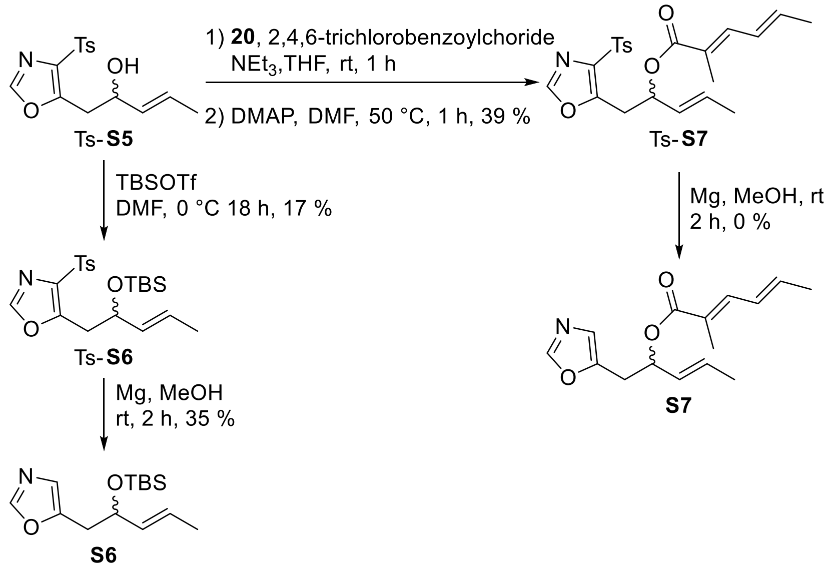

Scheme S1. Detosylation of ester protected tosyl-oxazoles Ts-**S6** or Ts-**S7**.

5.3 Investigations on detosylation of tosyl-oxazole (Ts-10)

Table S6: Optimization of the detosylation reaction with tosyl-oxazole alcohol Ts-**10**.

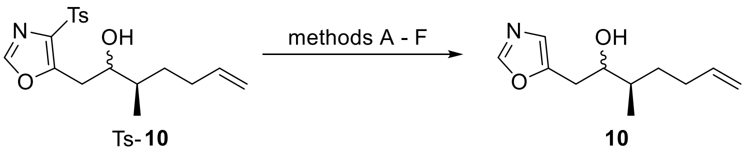

method	reactants (eq.)	solvent	temperature	time [h]	yield [%]	conversion [%]
A	Na, naphthalene	THF	-78 °C	4	0	100
B	Na, Na_2HPO_4	MeOH	0 °C	5	14	100
C	Mg (15)	MeOH	rt	1	25	100
D	Mg (5)	MeOH	rt	1	33	100
E	Mg (5)	MeOH	rt	2	35	100
F[a]	NaHg, Na_2HPO_4	THF/EtOH	rt	4	83	100

[a] ultrasound was used

5.4 Investigations on detosylation of Ts-(8) and Ts-(16)

Table S7: Optimization of detosylation reaction of *syn*-Ts-**8**, *anti*-Ts-**8**, *syn*-Ts-**16**, *anti*-Ts-**16**.

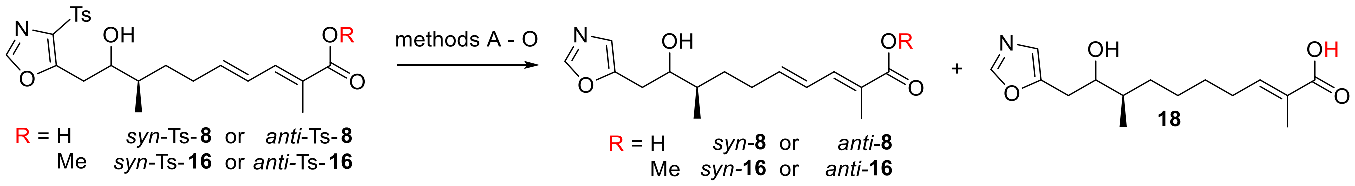

method	starting material (R)	reactants (eq)	solvent	temperature [°C]	time [h]	conversion [%]	yield [%]	yield **18** [%]
A	*syn*-Ts-**8** (H)	Mg	MeOH[a]	r. t. →reflux	24	-	0[c]	0
B	*syn*-Ts-**16** (Me)	Mg	MeOH[a]	rt	4	36	0	0
C	*syn*-Ts-**8** (H)	Na / naphthalene	THF[a]	-78 °C → rt	2	0	0[d]	0
D	*syn*-Ts-**16** (Me)	Na / naphthalene	THF[a]	-78 °C → rt	2	0	0[d]	0
E	*syn*-Ts-**16** (Me)	SmI_2 (3)	THF/ MeOH	-78 °C → rt	2	0	0[c]	0
F	*syn*-Ts-**16** (Me)	SmI_2 (2.2)	THF/ MeOH	78 °C → rt	2	0	0[d]	0
G	*anti*-Ts-**16** (Me)	SmI_2 (2.2)	THF	78 °C → rt	2	0	0[d]	0
H	*syn*-Ts-**8** (H)	SmI_2 (2.2)	THF/ MeOH	78 °C → rt	2	0	0[c]	0
I	*anti*-Ts-**16** (Me)	NaHg, Na_2HPO_4	EtOH/ THF[b]	rt	4	0[c]	0	0
J	*syn*-Ts-**8** (H)	NaHg, Na_2HPO_4	MeOH/THF[a]	rt	24	71	0	34 (crude)
K	*anti*-Ts-**16** (Me)	NaHg, Na_2HPO_4	MeOH/THF[a]	rt	48	0	0	0
L	*syn*-Ts-**8** (H)	NaHg, Na_2HPO_4	EtOH/ THF[b]	rt	4	100	20	0
M	*anti*-Ts-**8** (H)	NaHg, Na_2HPO_4	MeOH/THF[a]	rt	4	100	29	0
N	*syn*-Ts-**8** (H)	NaHg, Na_2HPO_4	MeOH/THF[a]	rt	24	100	-[e]	-[e]
O	*anti*-Ts-**8** (H)	NaHg, Na_2HPO_4	MeOH/THF[a]	rt	48	100	-[e]	-[e]

[a] stirring as mixing method; [b] ultrasound as mixing methode; [c] decomposition of the starting material; [d] reisolation of the starting material; [e] mixture of product **8** and **18**

6 Macrolactonization of *seco*-acids towards the macrodiolides

6.1 Stereochemical details

The macrolactonization was performed with the pure *seco*-acids *syn*-**8**, *anti*-**8** and *syn*-Ts-**8**, *anti*-Ts-**8** as well as with a (1 : 1) mixtures of **8** (*syn* / *anti* 56 : 44). In case of the pure *seco*-acids the respective pure macrolactones (*syn*,*syn*-**4a** or *anti*,*anti*-**4a** or *syn*,*syn*-Ts-**4a** or *anti*,*anti*-Ts-**4a)** are formed. However, in case of a (1 : 1) diastereomeric mixtures of **8** the formation of four macrolactones (*syn*,*syn*-**4a**, *anti*,*anti*-**4a**, *syn*,*anti*-**4a** , *anti*,*syn*-**4a**) are theoretically expected with the same mathematical probability: 25 : 25 : 25 : 25. But as outlined in Scheme S2, *syn*,*anti*-**4a**, *anti*,*syn*-**4a** are identical. This results in the following statistical diastereomeric distribution: *syn*,*syn*-**4a** : *syn*,*anti*-**4a**/*syn*,*anti*-**4a** : *anti*,*anti*-**4a** = 25 : 50 : 25. The same applies for Ts-**8**.

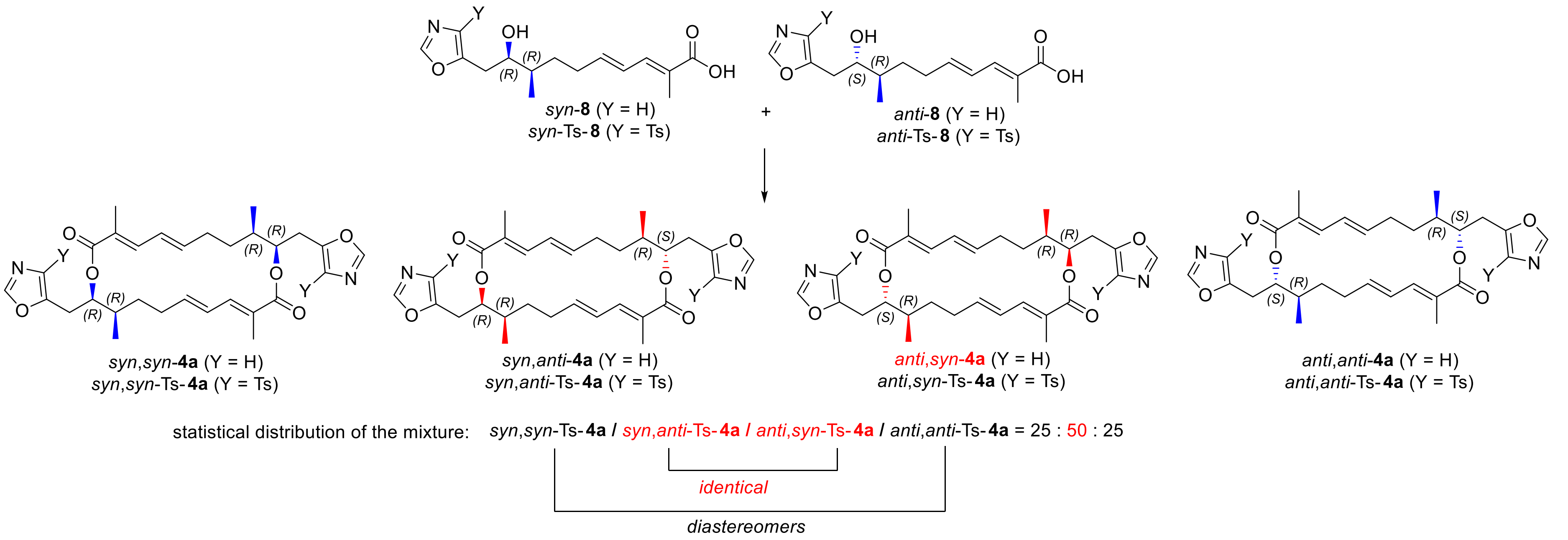

Scheme S2: Yamaguchi macrolactonization of the mixtures of 1 : 1 mixture of *syn*-**8**, *anti*-**8** and *syn*-Ts-**8**, *anti*-Ts-**8** to the macrodiolides.

6.2 Optimization of the Macrolactonization

Table S8 shows several attempts on the macrolactonization of a (1 : 1)-mixture of *syn*-Ts-**8**, *anti*-Ts-**8** *via* different approaches.

Table S8: Investigation of the macrolactonization of *seco*-acid Ts-**8**.

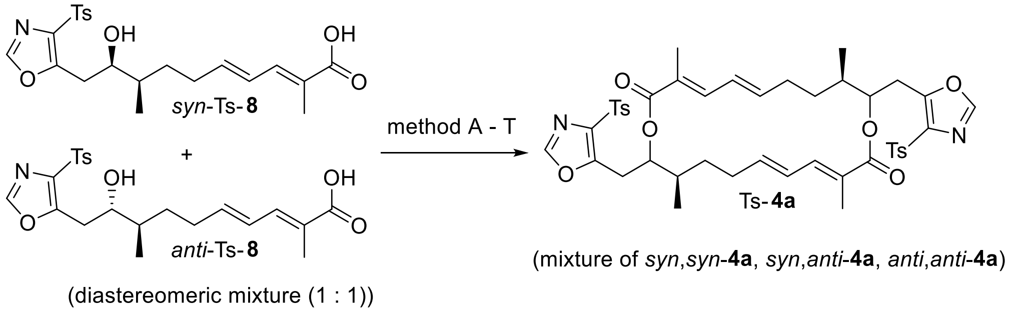

method	reagent	catalyst [mol%]	solvent	temperature [°C]	time [h]	yield [%]
A	Dy(OTf)$_3$	10	toluene	110	24	0
B	Dy(OTf)$_3$	10	dioxane	100	24	0
C	Hf(OTf)$_3$	10	dioxane	100	24	0
D	Hf(OTf)$_3$	10	DMF	110	72	0[a]
E	Hf(OTf)$_3$	10	DMSO	110	18	0
F	Hf(OTf)$_3$	10	benzene	80	24	0
G	Hf(OTf)$_3$	10	toluene	110	18	0[a]
H	Hf(OTf)$_3$	10	toluene	110	6	0[a]
I	Hf(OTf)$_3$	10	toluene / THF (20 : 1)	80	72	0[a]
J	Hf(OTf)$_3$	10	toluene / THF (4 : 1)	80	18	10[a]
K	Hf(OTf)$_3$	10	toluene / THF (4 : 1)	80	72	20[a]
L	Hf(OTf)$_3$	10	toluene / THF (1 : 1)	80	100	0
M	PPh_3, DEAD	-	toluene / THF (5 : 1)	-25	22	0
N	PBu_3, DIAD	-	toluene, THF	0	2	0
O	DCC, DMAP	-	CH_2Cl_2	rt	3	0[a]
P	TsCl	-	pyridine	0	18	0[b]
Q	$(PhCO)_2$, DMAP	-	CH_2Cl_2	rt	18	0[b]
R	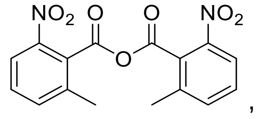 , DMAP	-	CH_2Cl_2	rt	18	<5
S	2,4,6-trichlorobenzoyl chloride, NEt_3, DMAP	-	THF toluene	110 °C	20	0
T	2,4,6-trichlorobenzoyl chloride, DMAP, mole sieve	-	CH_2Cl_2	rt	18	0

[a] reisolation of the starting material; [b] decomposition of the starting material (^{1}H NMR).

7 Alternative Approach towards samroiyotmycin A (4a)

As outlined in Scheme S3, we alternatively tested a RCM / CM approach towards dienoate **S8**. SuperQuat auxiliary **11** and 5-hexenoic acid were converted to *N*-acyloxazolidinone, followed by asymmetric methylation[2] providing derivative **12** in 98 % yield (over 2 steps) as a single diastereomer. Upon reductive cleavage of auxiliary **11** (recovered in 80 %)[2] the resulting α-branched alcohol was submitted to Ley oxidation[3] to give aldehyde **S9** in 76 % (over 2 steps).

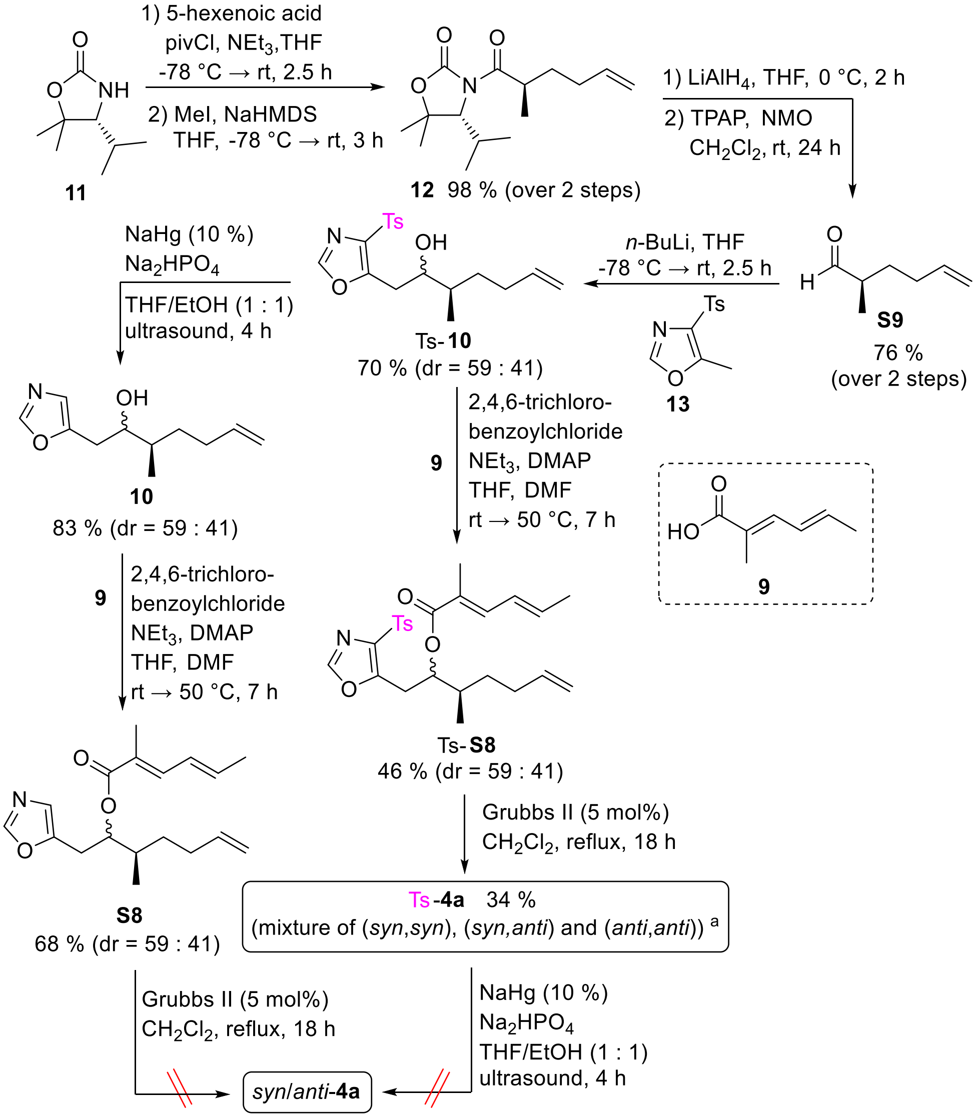

Scheme S3. Synthetic routes to esters **S8,** Ts-**4a** and attempts towards samroiyotmycin **4a**; [a] Due to overlapping ^{1}H-NMR signals dr could not be determined.

Subsequent C–C coupling with the lithiated 5-methyl-4-tosyloxazole **13**[4] gave the diastereomeric tosyloxazolyl alcohols Ts-**10** (*syn* / *anti* 59 : 41) in 70 % yield. Removal of the tosyl group with NaHg in the presence of Na_2HPO_4 in EtOH, THF under ultrasonification[4] gave oxazolyl alcohols **10** (*syn* / *anti* 59 : 41) in 83 % yield. Yamaguchi esterification[5] of **10** with 2-methylsorbic acid **9** yielded building block **S8** (*syn* / *anti* 59 : 41) in 68 %. Subsequent treatment with 5 mol% of Grubbs 2nd generation catalyst (Grubbs II)[6,7] in CH_2Cl_2 under

reflux,[8] however, did not give any trace of the desired macrocycle **4a**.This failure of **S8** to undergo sequential CM / RCM was rather surprising, because previous work from the literature revealed that oxazole precursors carrying methyl, ethylcarboxylate or an alkyl (alkenyl) chain at the 2-position of the oxazole did not compromise CM[9–12] or RCM,[13,14] and Cossy[11,12] demonstrated a broad scope for CM of vinyloxazoles. We surmised that in our case oxazoles **S8** with unsubstituted 2-position might either act as N-donor or NHC-like ligand at the Ru center, interfering with productive metathesis, and therefore C-tosyl protection seemed to be a precondition for successful metathesis. Thus, tosyloxazolyl alcohol Ts-**10** was esterified under Yamaguchi conditions to ester Ts-**S8** (*syn* / *anti* 59 : 41) in 46 % yield, which was subsequently treated with 5 mol% of Grubbs II. Macrodiolide Ts-**4a** could be isolated in 34 % yield (mixture of *syn*,*syn*-Ts-**4a**, *syn*,*anti*-Ts-**4a** and *anti*,*anti*-Ts-**4a** (see Scheme S2) revealing the suitability of the tosyl-protected oxazoles towards sequential CM / RCM. However, all attempts at reductive detosylation with NaHg to provide **4a** failed. The alternative cross metathesis described in table S4 of oxazolyl alcohol **10** (*syn* / *anti* 52 : 48) with 2-methylsorbic acid **9** in the presence of 5 mol% of Grubbs II to the desired *seco*-acid *syn*/*anti*-**8** completely failed, which is in good agreement with the above discussed reluctance of derivatives such as **S8**, carrying a free oxazole unit to undergo CM or RCM.

8 Synthesis of aromatic or heteroaromatic samroiyotmycin-derivatives

As we aimed to get some insight into the structure-activity relationship (SAR) of samroiyotmycin derivatives, analogues of *seco*-acids *syn*-Ts-**8**, *anti*-Ts-**8** carrying an aromatic or heteroaromatic unit instead of the oxazole moiety were prepared (Scheme S4). Starting from oxazolidinone **12**, reductive cleavage of auxiliary **11** (recovered in 80 %)[2] and subsequent Ley oxidation[3] of the resulting α-branched alcohol gave aldehyde **S8** in 76 % (over 2 steps). Treating **S9** with benzylmagnesium chloride in Et_2O at 40°C yielded the secondary alcohol **19** in 36 % as a diastereomeric mixture (*syn* / *anti* 67 : 33). When aldehyde **S9** was reacted with 2-naphthyl bromide in the presence of *n*-BuLi in THF at -78°C gave **24a** in 49 % (*syn* / *anti* 58 : 42) yield. Under similar conditions, the 2-pyridyl alcohol **24b** was isolated in 30 % (*syn* / *anti* 61 : 39) and 2-thienyl alcohol **24c** in 71 % (*syn* / *anti* 56 : 44) yield. In case of **24b**, *syn*- and *anti*-diastereomers could be separated by HPLC (for alternative routes see Scheme S5).

The corresponding alcohols **S10a**,**b** (Scheme 4b), devoid of the stereogenic methyl group, were obtained from Grignard reaction of benzaldehyde **S11** and 1-pentenylbromide **S12a** or 1-hexenylbromide **S12b** in the presence of Mg in 72 % (dr = 51 : 49, n = 1) and 59 % (dr = 50 : 50, n = 2) respectively. In case of benzyl- and phenyl-substituted alcohols **19**, **S10a,b**, CM with 2-methylsorbic acid **9** yielded the desired *seco*-acids **20** in 77 % (dr = 63 : 37, Bn) (Scheme S4a) and **22a**,**b** in 90 % (dr = 51 : 49, Ph, n = 1) as well as 98 % (dr = 50 : 50, Ph, n = 2) (Scheme S4b) respectively.

However, attempted CM of aryl-substituted alcohols **24a**,**b**,**c** with 2-methylsorbic acid **9** failed, whereas the corresponding reactions with 2-methylsorbic acid methylester **15** proceeded uneventfully with yields of 77 % (**25a**, dr = 58 : 42, Ar = 2-naphthyl) and 60 % (**25c**, dr = 54 : 46, Ar = 2-thienyl) (Scheme S4a). It should be emphasized that *syn*- and *anti*-

pyridylalcohols *syn*-**24b**, *anti*-**24b** could be separately converted to the *seco*-esters *syn*-**25b** and *anti*-**25b** in 22 % (*syn* / *anti* 100 : 0) and 20 % (*syn* / *anti* 0 : 100) respectively. Subsequent saponification of the methylesters **25** to the *seco*-acids **S13** was only possible with 2-naphthyl- and 2-thienyl-*seco*-esters **25a**, **25c** in 70 % (**S13a**, *syn* / *anti* 69 : 31) and 45 % (**S13b**, *syn* / *anti* 55 : 45) respectively. Surprisingly, *seco*-esters *syn*-**25b** and *anti*-**25b** both reacted to pyridyl ketone **S15**[15] in 19 % (see Scheme S5).

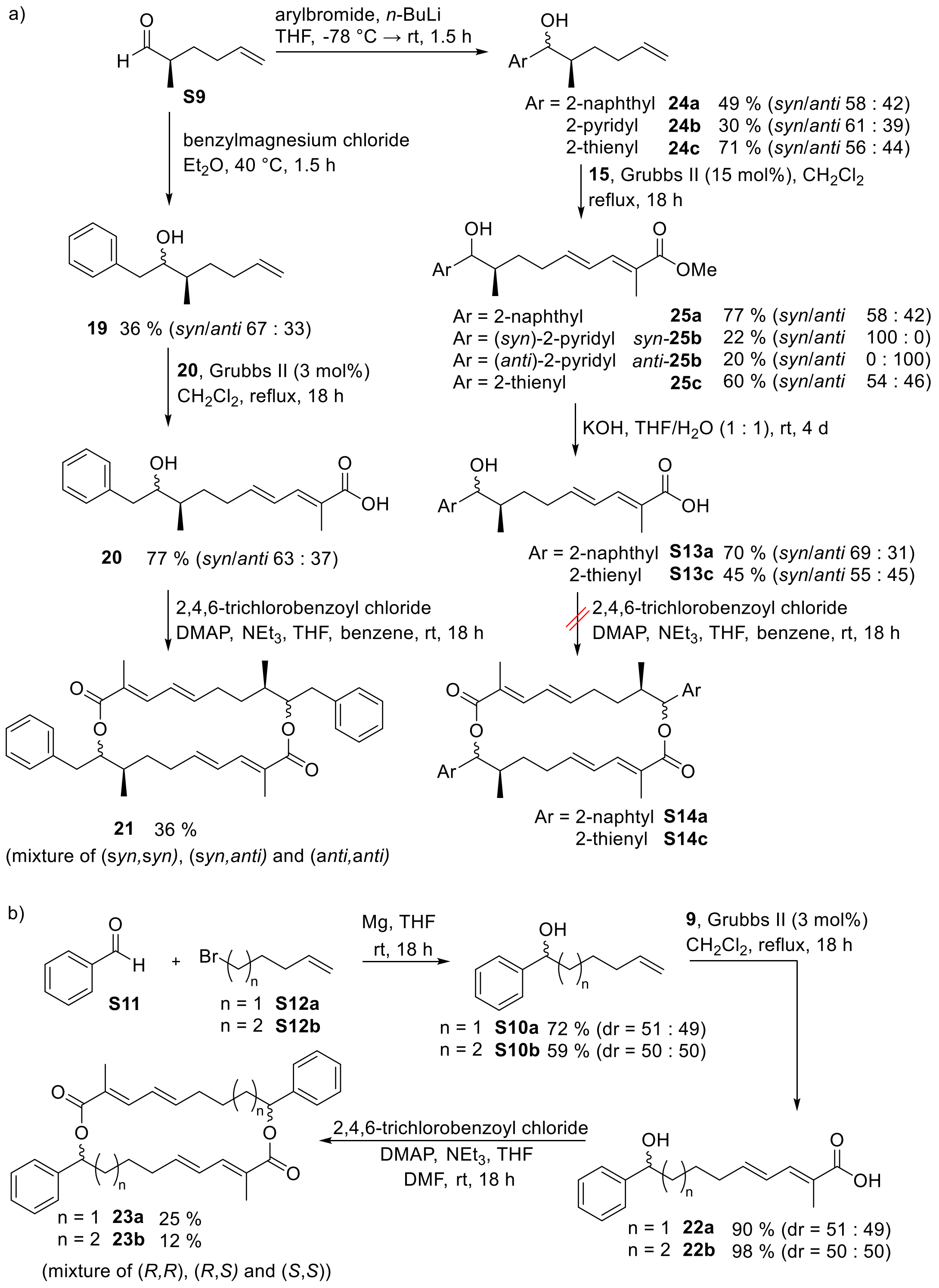

Scheme S4: Synthetic routes to samroiyotmycin derivatives and heteroaryl analogues.

Macrolactonization of 2-naphthyl- and 2-thienyl-*seco*-acids **S13a**,**c** failed, probably due to electronical and/or stereochemical effects. Under the same conditions, the macrolactonization of the benzyl- and phenyl-substituted *seco*-acids **20**, **22a**,**b** successfully yielded the desired samroiyotmycin-derivatives **21** in 36 % (Bn, Scheme S4a; product mixtures of *syn*,*syn*-**21**, *syn*,*anti*-**21**, *anti*,*anti*-**21** in analogy to Scheme S2) and **23a**,**b** in 25 % (Ph, n = 1) and 12 % (Ph, n = 2) (Scheme S4b; product mixtures of (*R*,*R*)-**23**, (*R*,*S*)-**23**, (*S*,*S*)-**23** in analogy to Scheme S2) respectively

Alternatively, secondary alcohols **19,24a-c**, were synthesized via Grignard reaction (Scheme S5). Scheme S5 also shows the formation of pyridyl ketone **S15** over 2 steps from **24b**

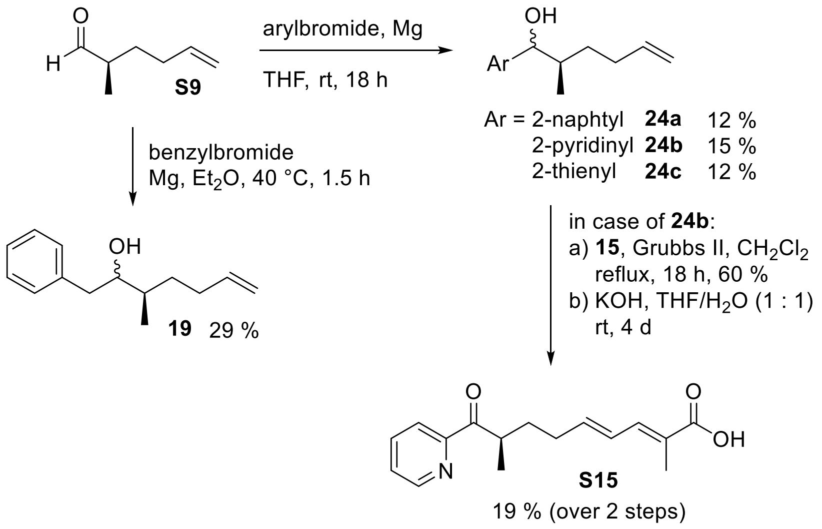

Scheme S5. Alternative synthesis of derivative **S15**.

9 Further investigations on macrolactonization towards (4a).

Mixtures of *syn*-*seco*-acids *syn*-**8** and the partially reduced acids *syn*-**18** (1 : 1) gave only traces of the macrodiolides *syn*,*syn*-**4a** and *syn*,*syn*-**S16** (Scheme S6) according to ESI-MS and ^{1}H NMR spectra. When the corresponding (1 : 1) mixture of *anti*-*seco*-acids *anti*-**8** and the partially reduced acids *anti*-**18** were treated under similar conditions, only the latter formed traces of the macrodiolide *anti*,*anti*-**S16** (according to ^{1}H-NMR), presumably resulting from conformational bias (Scheme S6).

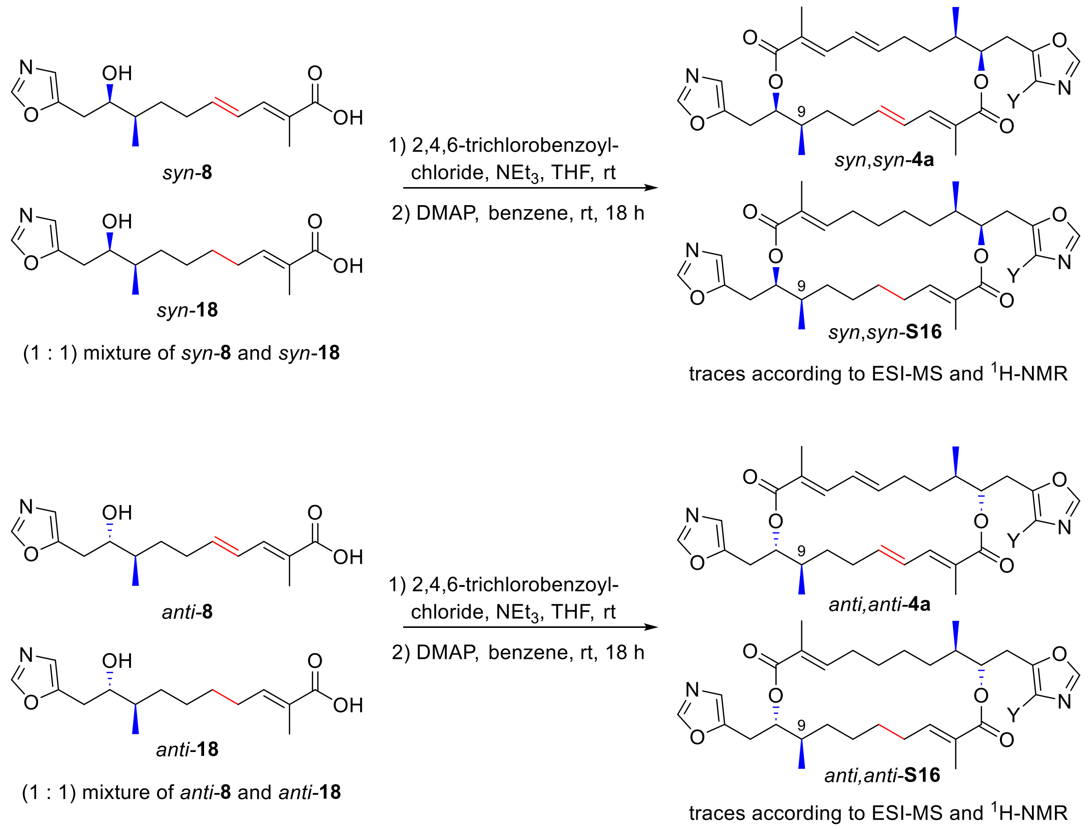

Scheme S6: Investigations on the macrolactonization of the mixtures of *syn*-**8** and the partially reduced acids *syn*-**18** (1 : 1) as well as *anti*-**8** and *anti*-**18** (1 : 1).

10 ^{1}H NMR stacked plots

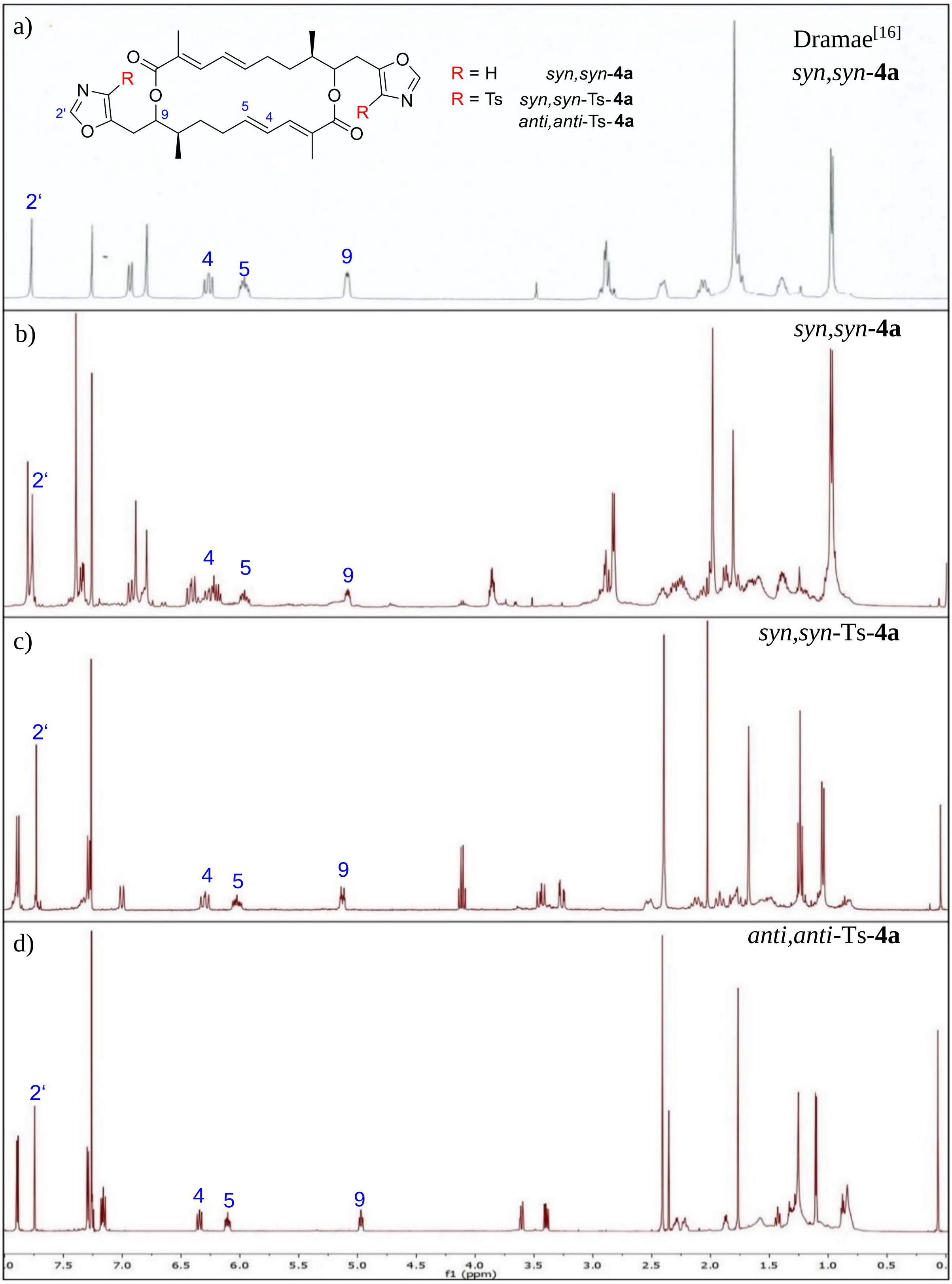

Figure S1: ^{1}H-NMR of a) samroiyotmycin A (*syn,syn*-**4a**) from the literature[16] in comparison to b) our synthesized *syn,syn*-**4a** (only isolated in traces and despite multiple purification steps not in pure form) from *syn*-**8** (*syn* : *anti* 100 : 1), c) *syn,syn*-Ts-**4a** and d) *anti,anti*-Ts-**4a**.

11 Biological investigations

11.1 Antimalarial in vitro activity against asexual blood stages of *Plasmodium falciparum* NF54 and K1 at Swiss TPH

Compounds were tested at SwissTPH against multidrug resistant (K1) and sensitive (NF54) strains of *P. falciparum* in vitro using the at Swiss TPH in developed [^{3}H]-hypoxanthine incorporation assay, as previously reported.[17] Parasites were cultured using a medium containing RPMI 1640 (10.44 g/L) (no hypoxanthine) supplemented with HEPES (5.94 g/L), NaHCO3 (2.1 g/L), neomycin (100 μg/mL), and Albumax II (5 g/L). An uninfected red blood cell solution (1 mL) was prepared (2.5% hematocrit) containing 50 μL of washed human erythrocytes (50% hematocrit) and mixed with 950 μL of screening medium. The screening medium (100 μL) was then added to each well of the Falcon 96-well microtiter plate. Serial drug dilutions were then prepared across the plate using an additional 100 μL of the screening medium. Infected blood (100 μL) (parasitemia of 0.3%, 2.5% hematocrit) was added to all wells in the plate with the exception of control wells. The plates were incubated at 37 °C in an atmosphere of 93% N_2, 4% CO_2, and 3% O_2. After 48 h, 50 μL of [3H]-hypoxanthine (0.25 μCi) solution was added to each well of the plate, and plates were incubated for another 24 h. The plates were then harvested with a Betaplate cell harvester (Wallac, Zurich, Switzerland) and, after collection by filtration dilution with scintillation fluid, were counted in a Betaplate liquid scintillation counter (Wallac, Zurich, Switzerland). The results were recorded as counts per minute (cpm) per well at each drug concentration. Data were transferred into the MS Excel software and expressed as a percentage of the untreated controls. The 50 % inhibitory concentration (IC_{50}) value was evaluated by logit regression analysis.

11.2 Antimalarial in vitro activity against asexual blood stages of *Plasmodium falciparum* NF54 at H3D

The test samples were tested in triplicate over 72 hours on two occasions against the wild-type chloroquine sensitive (CQS) strain of *Plasmodium falciparum* (NF54). Continuous *in vitro* cultures of asexual erythrocyte stages of *P. falciparum* were maintained using a modified version of the method of Trager and Jensen[18]. Parasites were maintained in complete medium supplemented with 0.5% (m/v) of Albumax-II (Gibco). Cultures were kept at 2% haematocrit and parasitemia lowered to 1% during the trophozoite stage via the addition of fresh erythrocytes (Rh-type O+). Cultures were kept in a specialised atmosphere of 4% CO_2 and 3% O_2 in nitrogen and housed at 37°C.

Quantitative assessment of antiplasmodial activity *in vitro* was determined via the parasite lactate dehydrogenase assay using the method described by Makler[19]. The test samples were prepared to a 10mM stock solution in 100% DMSO. Samples were tested as a suspension if not completely dissolved. Stock solutions were stored at room temperature. Further dilutions were prepared on the day of the experiment. The antimalarial drugs Chloroquine (CQ) and Artesunate (AS) were used as the reference drug in all experiments. A full dose-response was performed for all compounds in a 96-well plate to determine the concentration inhibiting 50% of parasite

growth (IC_{50}–value). Test samples were tested at a starting concentration of 10 mM, which was then serially diluted 2-fold in complete medium to give 10 concentrations; with the lowest concentration being 0.0195µM. The same dilution technique was used for all samples. CQ and AS were also tested from a starting concentration of 1 µg/mL. The highest concentration of solvent to which the parasites were exposed to had no measurable effect on the parasite viability (data not shown). The IC_{50}-values were obtained using a non-linear dose-response curve fitting analysis via Dotmatics software.

11.3 Antibacterial and cytotoxic activity at HZI

The biological investigations were carried out in the working group of Prof. Dr. Ursula Bilitewski at the Helmholtz Centre for Infection Research (HZI) in Braunschweig. The experiments were designed to examine the inhibitory potential of the compounds towards the growth of selected bacterial strains as well as the cell viability of the cell line L929 (mouse fibroblasts). The Gram-positive bacterium *Staphylococcus aureus* (*S. aureus*, strain SH1000 or USA300) and the Gram-negative bacterium *Escherichia coli* K12 (*E. coli* K12) and its deletion mutant *E. coli* Δ*tolC* were used as test organisms. The protein TolC is a component of an efflux pump, which transports non-cellular substances out of the cell, which is lacking in the deletion mutant. As a result, the pump becomes inactive and substances foreign to the cell cannot be transported further out of the cell.
To investigate the biological activity, 10 mM stock solutions of the substances were prepared in DMSO. In initial tests, the compounds were analysed at single concentration of 100 µM and subsequently in a dose-dependent manner were required.

11.3.1 Test for antibacterial activity

The minimum inhibitory concentrations (MICs) of the tested compounds were determined using the broth microdilution method, according to guidelines outlined by the Clinical and Laboratory Standards Institute (CLSI).[20] Bacterial cultures were initiated by growing *S. aureus* in tryptic soy broth or *E. coli* in lysogeny broth for 16 hours at 37 °C in baffled Erlenmeyer flasks while shaking at 150 rpm. Optical density at 600 nm (OD_{600}) was measured and new cultures were started at $OD_{600} = 0.1$ and strains were further grown until $OD_{600} \sim 0.5$ to achieve a growth in the logarithmic phase. Bacterial cultures were thereafter diluted to $OD_{600} = 0.01$ and transferred to a 96-well half area plate mixed with test compounds at single concentrations or in multiple concentrations in dose-dependent manner. Plates were incubated for 20-24 hours at 37 °C and OD_{600} was measured to determine growth. DMSO and/or water was used as vehicle controls. The final concentration of DMSO in each well did not exceed 1%. Wells without bacteria was used as background measurement. IC_{50} values were calculated by using the non-linear regression, four parameter equaltion of GraphPad Prism. The effect of compound treatment on bacterial growth was determined as percentage growth in relation to the no-compound controls.

11.3.2 Test for Cytotoxicity

The compounds were screened for their cytotoxicity using the *AlamarBlue*™ reagent [ThermoFischer Scientific], a resazurin based solution to measure cell viability. L929 cells were cultured in Dulbecco's Modified Eagle Medium (DMEM) supplemented with 10% fetal bovine

serum (FBS) and 10^3 cells/well was seeded in 96-well in half area cell culture plates. The plates with cells were incubated for 24 h at 37 °C and 10 % CO_2. Subsequently, test compound solutions were added per well at single a single concentration or in multiple concentrations in a dose-dependent manner. The plate was placed on a plate shaker for 15 seconds at 300 rpm to ensure optimal mixing and incubated at37°C and 10 % CO_2. After 72 h of incubation 5 µl of *AlamarBlue*™ reagent was added to each well. The fluorescence intensity of each well was determined after 2.5 h of incubation at 37°C and 10 % CO_2, using an extinction wavelength of (λmax, abs= 540 nm) and an emission wavelength of 600 nm (λmax, em= 600 nm). DMSO and/or water was used as vehicle controls. The final concentration of DMSO in each well did not exceed 1%**.** IC_{50} values were calculated by using the non-linear regression, four parameter equaltion of GraphPad Prism. The effect of compound treatment on cell viability was determined as percentage viability in relation to the no-compound controls.

11.3.3 Summary of antibacterial and cytotoxic activity

A

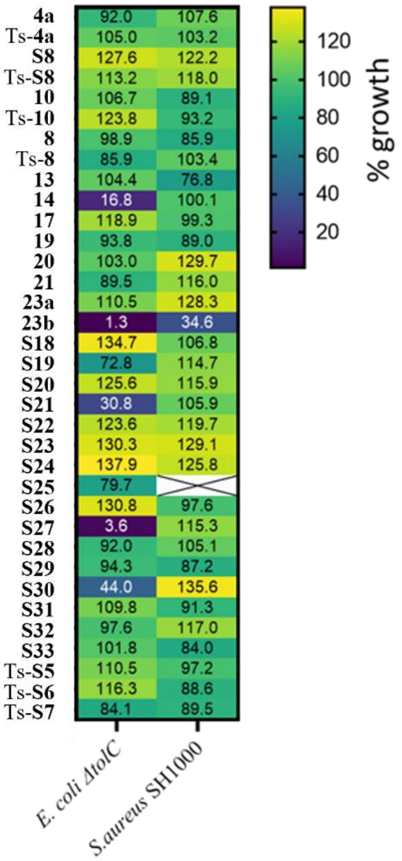

B

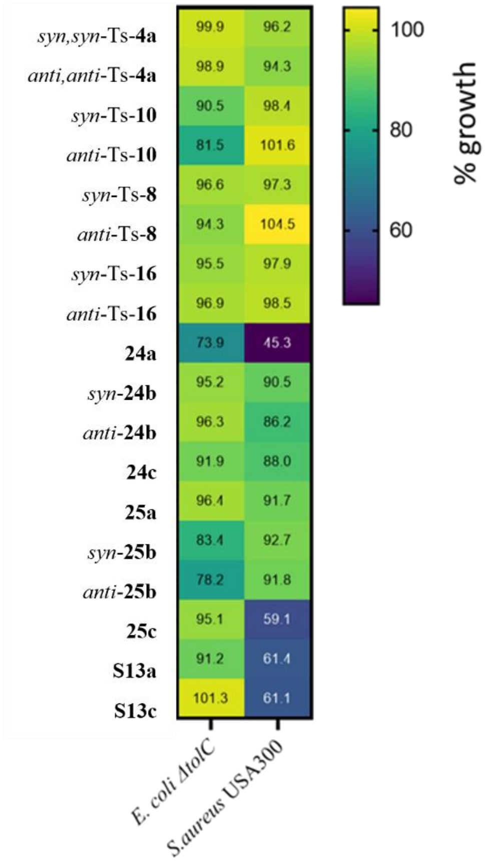

C D

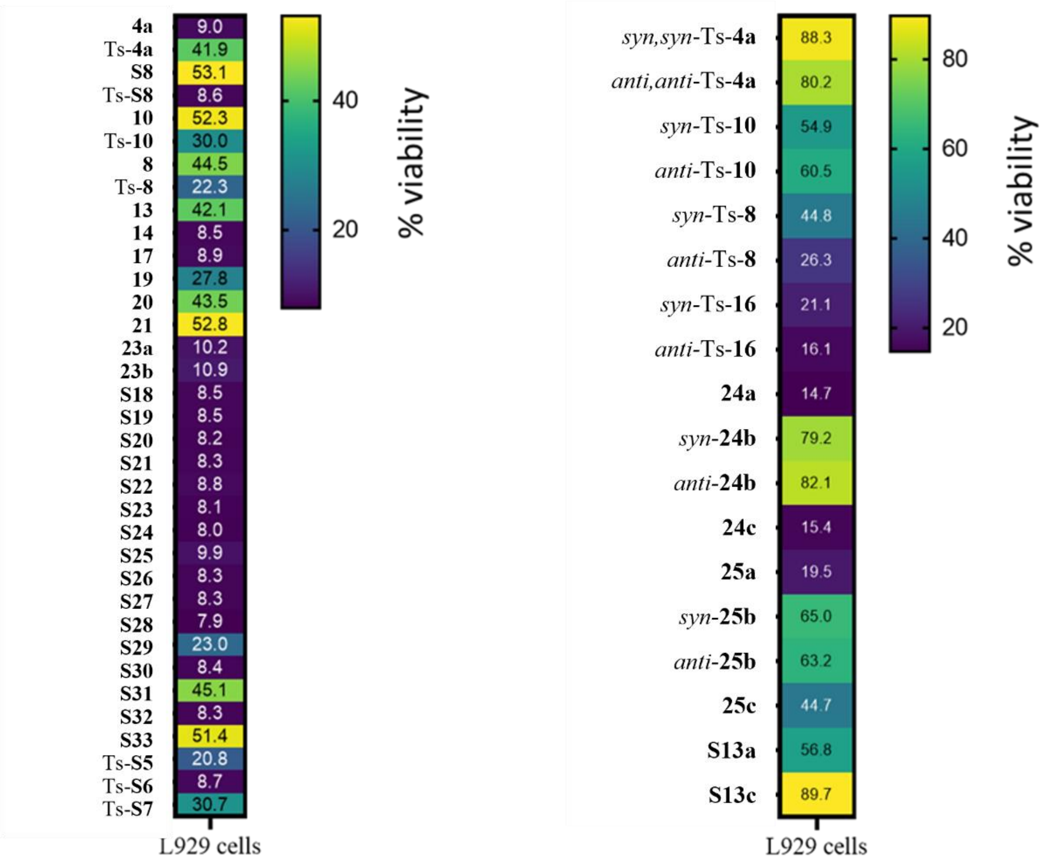

Figure S2: Heatmaps summarizing the antibacterial and cytotoxic properties of compounds at 100 µM. (A-B) Activity of compounds in the antibacterial growth inhibition assay against *E. coli ΔtolC* and *S. aureus* represented as % growth. (C-D) Cytotoxicity of compounds against L929 mouse fibroblast cells based on the alamarBlue assay represented as % viability of cells. Each data represents mean of two replicates.

11.4 Summary of the biological results

Table S9 shows the biological activity of samroiyotmycin **4a** in comparison with its analogues and some derivatives. In addition, all investigated borrelidin precursors are summarized in Table S10.

Table S9: Samroiyotmycin A *syn,syn*-4a **4a** in comparison to its analogues and some derivatives.

Entry		Compound	dr (*syn* : *anti*) [j]	Antiplasmodial activity (*P. falciparum*)		Cytotoxicity	Antimicrobial activity	
				IC_{50} [μM] (NF54)	IC_{50} [μM] (K1)	IC_{50} [μM] (L929)[e]	IC_{50} [μM] (*E. coli TolC*)[e]	IC_{50} [μM] (*S. aureus*)[e]
1	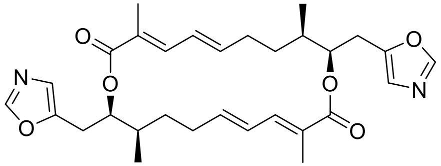	samroiyotmycin A *syn,syn*-**4a** [a]	100 : 0 : 0 [h]	0.77 [d]	0.51 [d]	- [b]	- [b]	- [b]
2	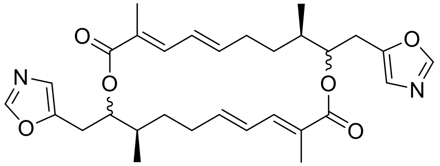	**4a** [g]	- [b]	>6[c]	- [b]	>100	>100	>100
3	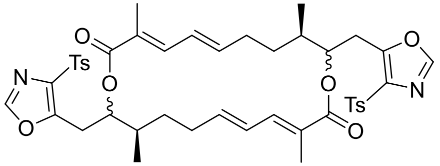	Ts-**4a** [g]	- [b]	4.38 [c] 1.61 [d]	- [b]	>100	>100	>100
4	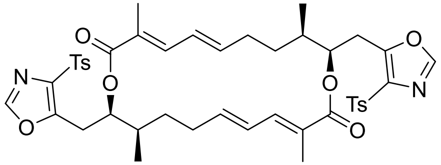	*syn,syn*-Ts-**4a**	100 : 0 : 0 [h]	0.17 [d]	0.15 [d]	>100	>100	>100
5	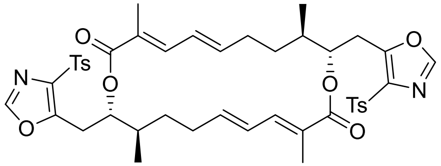	*anti,anti*-Ts-**4a**	0 : 0 : 100 [i]	0.26 [d]	0.25 [d]	>100	>100	>100

6	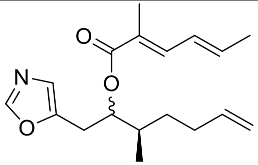	**S8**	59 : 41	>10 [c]	- [b]	>100	>100	>100
7	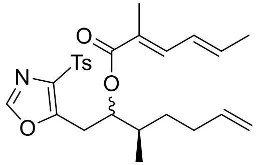	Ts-**S8**	59 : 41	>6 [c]	- [b]	>100	>100	>100
8	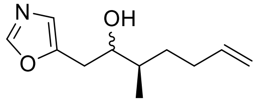	**10**	59 : 41	>10 [c]	- [b]	>100	>100	>100
9	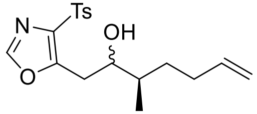	Ts-**10**	59 : 41	>6 [c]	- [b]	>100	>100	>100
10	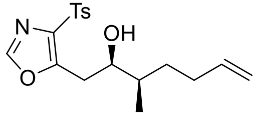	*syn*-Ts-**10**	100 : 0	>10 [d]	- [b]	>100	>100	>100
11	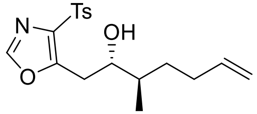	*anti*-Ts-**10**	0 : 100	>10 [d]	- [b]	>100	>100	>100
12	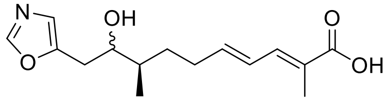	**8**	59 : 41	>6 [c]	- [b]	>100	>100	>100
13	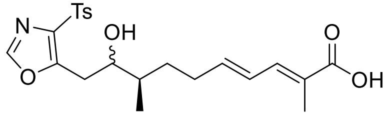	Ts-**8** [f]	59 : 41	>6 [c]<br>>10 [d]	- [b]	>100	>100	>100
14	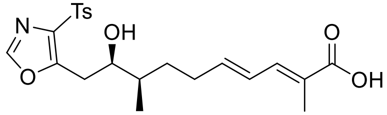	*syn*-Ts-**8**	100 : 0	13.47 [d]	- [b]	>100	>100	>100

15	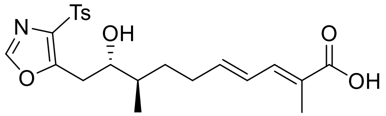	*anti*-Ts-**8**	0 : 100	17.31 [d]	- [b]	>100	>100	>100
16	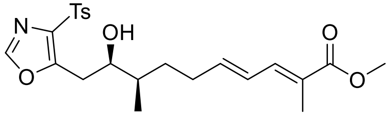	*syn*-Ts-**16**	100 : 0	0.94 [d]	0.95 [d]	>100	>100	>100
17	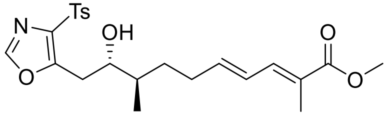	*anti*-Ts-**16**	0 : 100	2.44 [c]	- [b]	>100	>100	>100
18	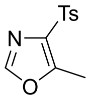	**13**	-	>6 [c]	- [b]	>100	>100	>100
19	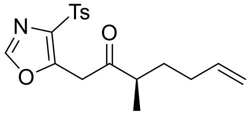	**14** [f]	-	>6 [c]<br>>10 [d]	- [b]	23.7 ± 1.65	~31	>100
20	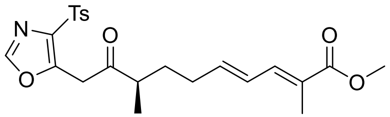	**17** [f]	-	3.32 [c]<br>2.85 [d]	- [b]	>100	>100	>100
21	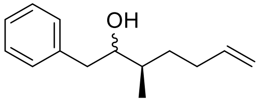	**19**	67 : 33	>10 [c]	- [b]	>100	>100	>100
22	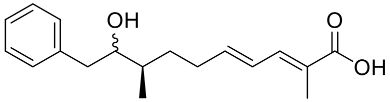	**20**	63 : 37	>6 [c]	- [b]	>100	>100	>100
23	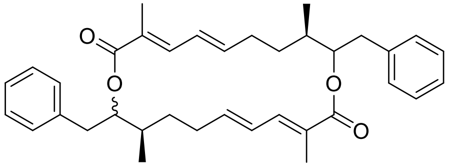	**21**	52 : 48	>10 [c]	- [b]	>100	>100	>100
24	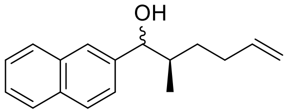	**24a**	58 : 42	>10 [d]	- [b]	>100	>100	>100

25	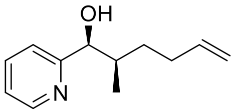	*syn*-**24b**	100 : 0	>10 [d]	- [b]	>100	>100	>100
26	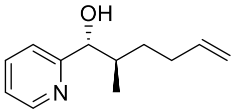	*anti*-**24b**	0 : 100	>10 [d]	- [b]	>100	>100	>100
27	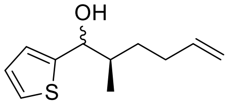	**24c**	56 : 44	>10 [d]	- [b]	>100	>100	>100
28	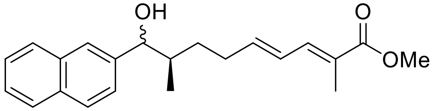	**25a**	58 : 42	14.64 [d]	- [b]	>100	>100	>100
29	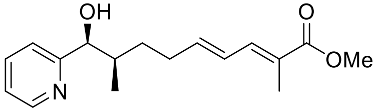	*syn*-**25b**	100 : 0	14.65 [d]	- [b]	>100	>100	>100
30	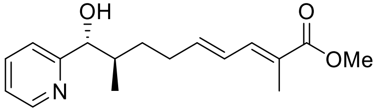	*anti*-**25b**	0 : 100	24.13 [d]	- [b]	>100	>100	>100
31	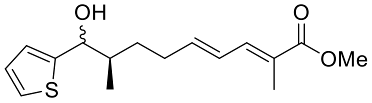	**25c**	54 : 46	3.50 [d]	- [b]	>100	>100	>100
32	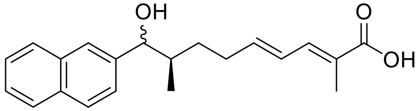	**S13a**	69 : 31	>50 [d]	- [b]	>100	>100	>100
33	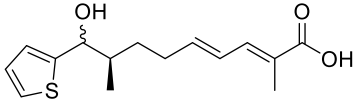	**S13c**	55 : 45	39.77 [d]	- [b]	>100	>100	>100

34	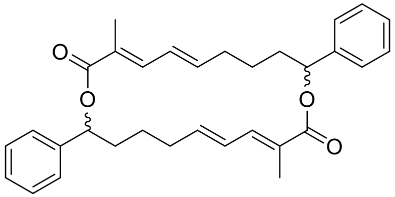	**23a**	-	>10 [c]	- [b]	>10	>100	>100
35	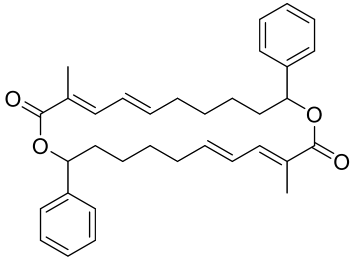	**23b**	-	>6 [c]	- [b]	>6	>100	>100
52	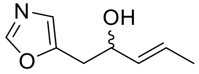	**S5**	50 : 50	>6 [c]	>100	>100	>100	52
53	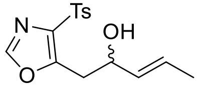	Ts-**S5**	50 : 50	>6 [c]	- [b]	>100	>100	>100
54	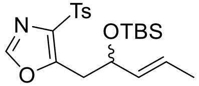	Ts-**S6**	50 : 50	>10 [c]	- [b]	>100	>100	>100
56	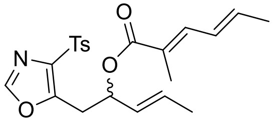	Ts-**S7**	62 : 38	>10 [c]	- [b]	>100	>100	>100
57	-	chloroquine [f]	-	0.0073 [c]<br>0.0075 [d]	0.27 [d]	- [b]	- [b]	- [b]
58	-	artesunate [f]	-	0.0046 [c]<br>0.0064 [d]	0.0095 [d]	- [b]	- [b]	- [b]

[a] samroiyotmycin A *syn,syn*-**4a** isolated from natural source[16,21]; reported data from Lit.[16] = 6.98 µM determined by [3H] hypoxanthine incorporation assay[16]; The ~10x increase in potency observed at Swiss TPH compared to the data described in ref.[16] can likely be explained by the difference in assay duration. It is well described in the literature that antibiotics, including azithromycin, doxycycline, and clindamycin, kill the progeny of the treated parasites rather than the treated parasites themselves.[22] This phenomenon, referred to as delayed death, means that the antibiotics exhibit increased potency after two intraerythrocytic cycles (96 h) compared to after just one cycle (48 h). Hence, the potency can be substantially increased when the assay duration in an antimalarial assay is increased. In the present work, samroiyotmycin A *syn,syn*-**4a** was for 72 h in contact with the parasites (1,5 intraerythrocytic cycle). In the study by Dramae[16] the assay duration was 42 h (less than one intraerythrocytic cycle). [b] not determined; [c] tested

at H3D using parasite lactate dehydrogenase assay; [d] tested at Swiss TPH using the 72 h [3H] hypoxanthine incorporation assay. The experiments were performed 2x (biological replicates) and the individual IC50 values varied by less than 50%; [e] tested at HZI; [f] for reasons of comparability of the activity tests performed at H3D and Swiss TPH; [g] mixture of *syn*,*syn*- / *syn*,*anti*- / *anti*,*anti*-products (see Scheme S2). Due to overlapping ^{1}H NMR signals, determination of dr not possible; [h] dr (*syn*,*syn* / *syn*,*anti* / *anti*,*anti*) = 100 : 0 : 0 (according to ^{1}H NMR, no traces of the minor diastereomer(s) were visible); [i] dr (*syn*,*syn* / *syn*,*anti* / *anti*,*anti*) = 0 : 0 : 100 (according to ^{1}H NMR, no traces of the minor diastereomer(s) were visible); [j] determined via ^{1}H NMR.

Table S10: Biological activity of borrelidin **S17** and some derivatives.

Entry	Compound		Antiplasmodial activity (*P. falciparum*)	Cytotoxicity		Antimicrobial activity
			IC_{50} [μM] (NF54)	IC_{50} [μM] (L929)[e]	IC_{50} [μM] (*E. coli TolC*)[e]	IC_{50} [μM] (*S. aureus*)[e]
36	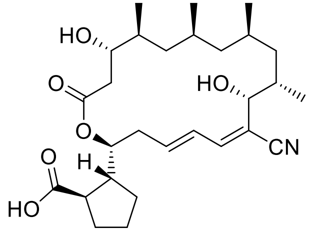	**S17** [d]	<0.0195 [b]<br>0.0032 [c]	0.49 ± 0.19	-[a]	-[a]
37	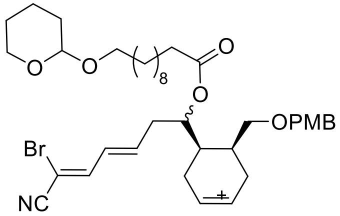	**S18**	4.02 [b]	>100	>100	>100
38	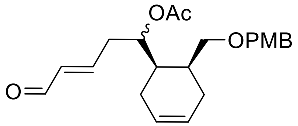	**S19**	>6 [b]	>100	>100	>100
39	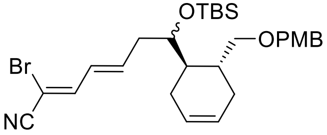	**S20**	2.74 [b]	>100	>100	>100
40	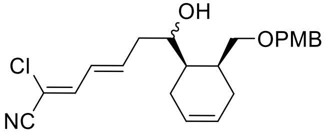	**S21**	>6 [b]	9.5 ± 0.46<br>23.3 ± 7.90	38.5 ± 3.19	>100

41	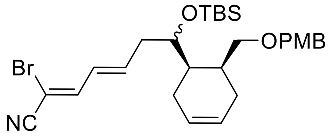	**S22**	1.66 [b]	>100	>100	>100
42	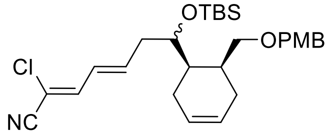	**S23**	0.72 [b]	>100	>100	>100
43	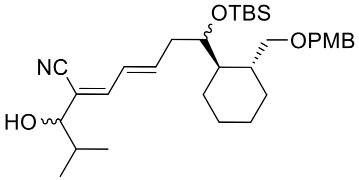	**S24**	7.21 [b]	14.5 ± 4.40	>100	>100
44	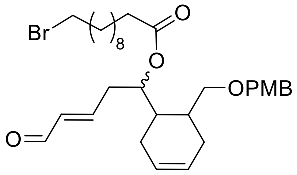	**S25**	>10 [b]	>100	>100	>100
45	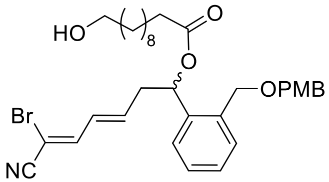	**S26**	1.20 [b]	22.3 ± 9.80	>100	>100
46	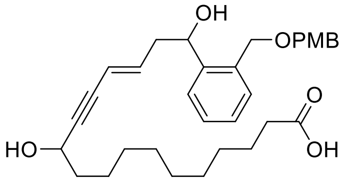	**S27**	>10 [b]	26.5 ± 1.06	34.9 ± 6.12	>100
47	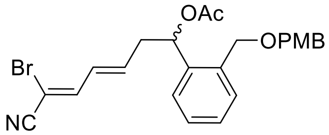	**S28**	>10 [b]	>100	>100	>100

48	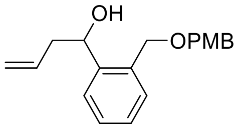	**S29**	>6 [b]	>100	>100	>100
49	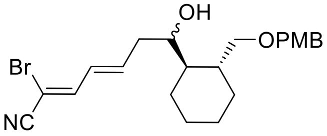	**S30**	7.78 [b]	5.3 ± 0.14<br>16.9±3.4	67.5 ± 6.12	>100
50	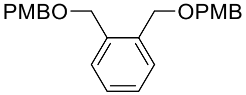	**S31**	3.28 [b]	>100	>100	>100
51	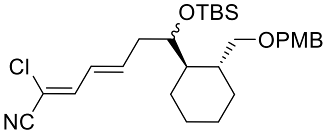	**S32**	>10 [b]	>100	>100	>100
57	-	chloroquine [d]	0.0073 [b]<br>0.0075 [c]	- [a]	- [a]	- [a]
58	-	artesunate [d]	0.0046 [b]<br>0.0064 [c]	- [a]	- [a]	- [a]

[a] not determined; [b] tested at H3D using parasite lactate dehydrogenase assay; [c] tested at Swiss TPH using the 72 h [3H] hypoxanthine incorporation assay. The experiments were performed 2x (biological replicates) and the individual IC50 values varied by less than 50%; [d] for reasons of comparability of the activity tests performed at H3D and Swiss TPH; [e] tested at HZI.

12 Molecular Docking with Yasara Structure

Molecular docking of *syn*,*syn*-**4a** with haemozoin (*β*-hematin) were performed using Yasara Structure (Version 24.4.10).[23–25] The known X-ray crystal structure data of *syn*,*syn*-**4a**[16] and hemozoin (*β*-hematin)[26] were downloaded from the CCSC data base under the codes WIMKIO and XETXUP03, respectively and used as input structures. Energy minimization of the crystal structure data was performed *via* the aYasara structure tool before docking.

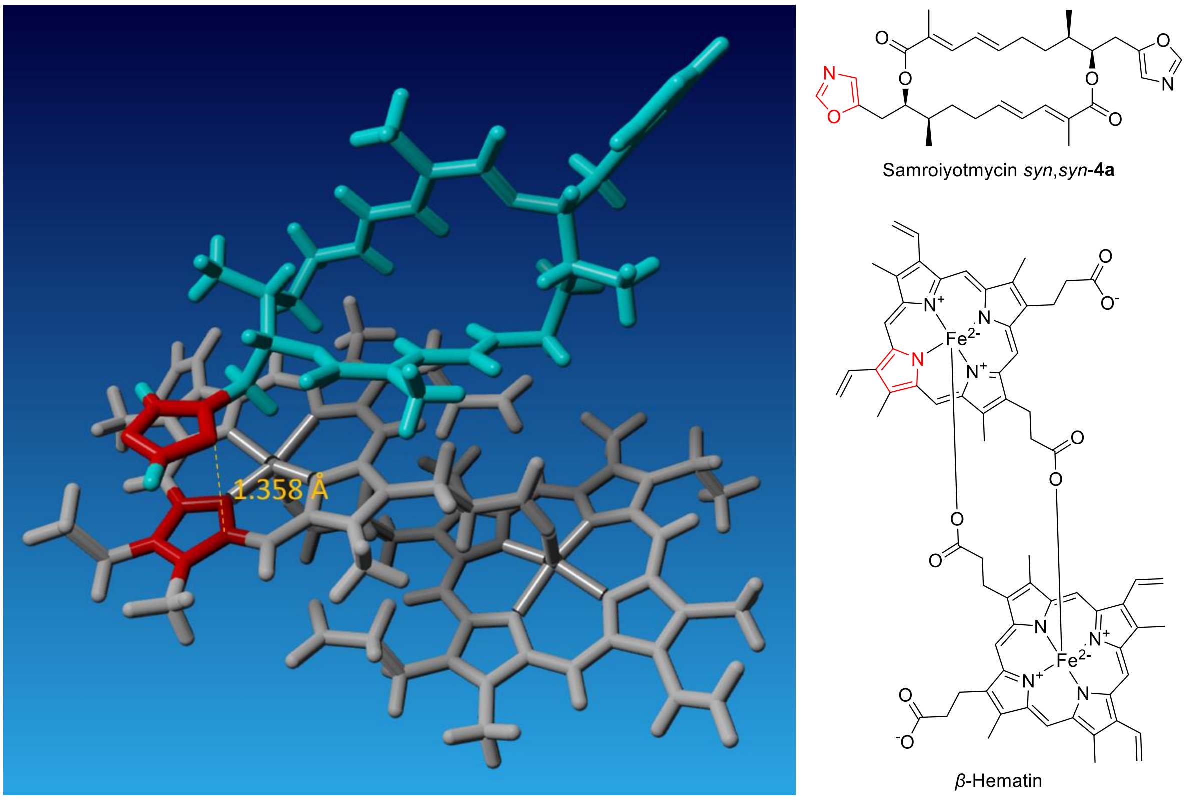

Figure S3: Docking of *syn*,*syn*-**4a** (blue) with haemozoin (*β*-hematin, grey) showing π-π-interactions of *β*-hematin's pyrrol residue and the oxazol unit of *syn*,*syn*-**4a** (both marked red) in a distance of 1.358 Å.

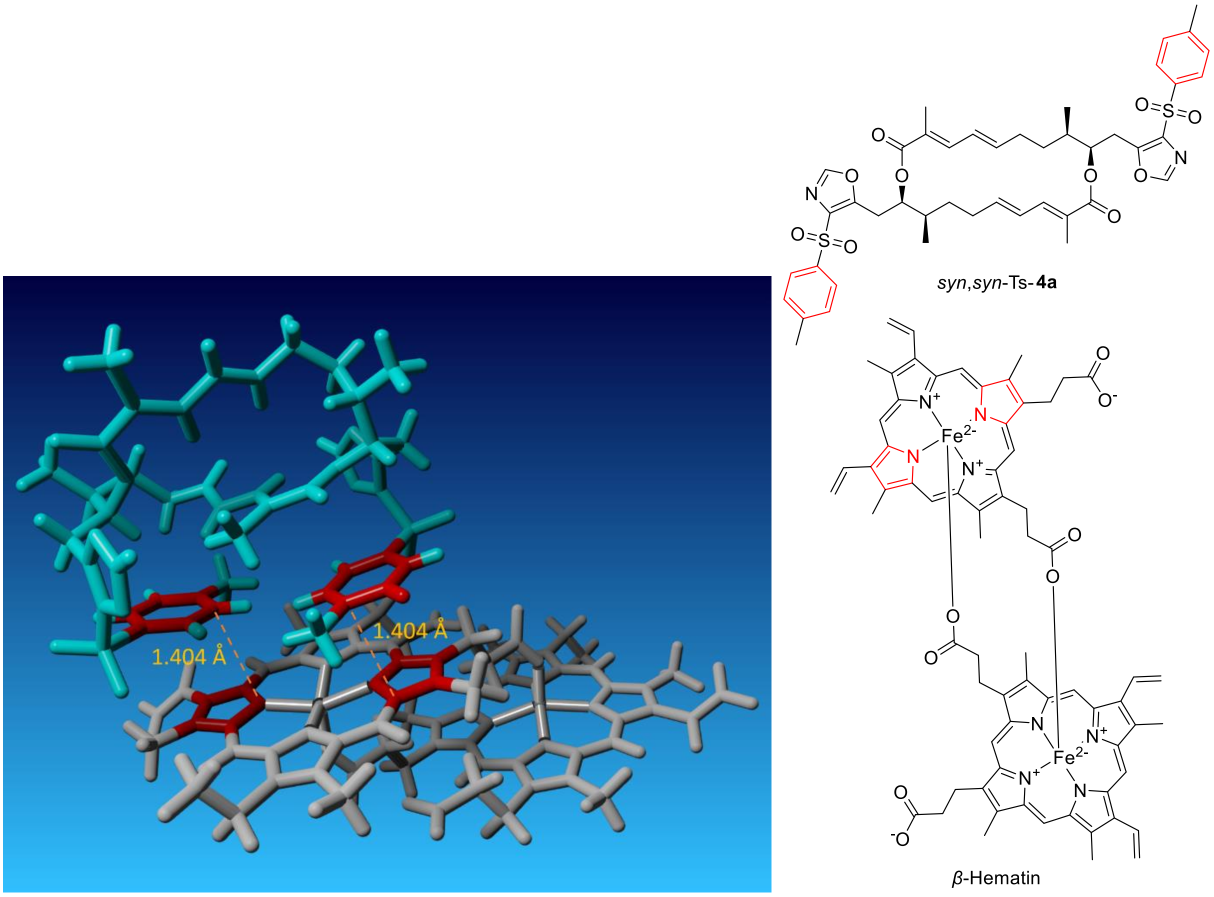

Figure S4: Docking of *syn*,*syn*-Ts-**4a** (blue) with haemozoin (*β*-hematin, grey) showing π-π-interactions of *β*-hematin's pyrrol residues and the tosyl groups of *syn*,*syn*-Ts-**4a** (both marked red) in a distance of 1.404 Å.

13 Experimental Details

13.1 General Information

Unless otherwise stated, reagents and solvents were used without further purification and all reactions were carried out under air. Commercially available chemicals were purchased at Sigma-Aldrich, Alfa Aesar or TCI. Hexane, CH_2Cl_2 and ethyl acetate (EtOAc) for chromatography were distilled prior to use. Air and moisture sensitive reactions are explicitly stated and were conducted under an atmosphere of nitrogen in anhydrous solvents using Schlenk technique. Anhydrous CH_2Cl_2 and triethylamine were obtained by distillation over CaH_2, whereas toluene was distilled over sodium. Cooling baths were prepared using acetone and liquid N_2. Thin-layer chromatography was conducted on DC Fertigfolien ALUGRAM® Xtra SIL G/UV254 from Machery-Nagel and R_f-values were rounded to 0.05. Compounds were detected by fluorescence (UV-light λ = 254 nm) and molybdatophosphoric acid stain (prepared from molybdatophosphoric acid (12.0 g) in EtOH (250 mL)). Flash chromatography was performed on Fluka silica gel 60 (40 - 63 μm) using the indicated solvents. NMR spectra were recorded on Bruker Avance 300, Ascend 400, Avance 500 and Avance 700 spectrometers in $CDCl_3$. The respective ^{1}H (^{13}C)-resonance frequencies were 300 MHz (75 MHz), 400 MHz (101 MHz), 500 MHz (126 MHz) and 700 MHz (176 MHz). Chemical shifts are given in ppm and $CDCl_3$ solvent peaks were used as reference for ^{1}H and ^{13}C spectra as internal standard (^{1}H NMR: $\delta(CDCl_3)$ = 7.26 ppm, ^{13}C NMR: $\delta(CDCl_3)$ = 77.1 ppm). Proton and carbon signal assignment as well as stereochemical elucidation was conducted with 2D NMR techniques (COSY, HSQC, HMBC, NOESY). The shown numbers are freely chosen for NMR-assignment and do not match the IUPAC nomenclature. No assignment is given for previously reported compounds. For FT-IR measurements a Bruker Vektor 22 with a MKII Golden Gate Single Reflection Diamant ATR for reduced total reflection was used. Mass spectra were obtained on Bruker Daltonics micro-TOF-Q (ESI) and Varian MAT 711 (EI) devices.

13.2 General Procedures

Oxazole detosylation with sodium amalgam (GP1)

Under an inert atmosphere (N_2), a tosylated oxazole (140 mg, 0.40 mmol) was dissolved in dry THF (5 mL) and dry EtOH or dry MeOH (5 mL) at rt, Na_2HPO_4 (226 mg, 1.60 mmol) was added and the reaction mixture was stirred or treated in an ultrasound bath for 10 min. In a separate flask, mercury (414 mg, 2.07 mmol) and sodium (46 mg, 2.00 mmol) were brought to reaction and the resulting sodium amalgam (460 mg, 2.00 mmol, 10 %) was added to the reaction mixture and stirred or treated in an ultrasound bath for 2 h. Additional sodium amalgam (460 mg, 2.00 mmol, 10 %) was added and after 2 h the reaction mixture was treated with EtOAc (20 mL) and subsequently added to demin. water (80 mL). The organic phase was washed with sat. $NaHCO_3$-solution (2 x 10 mL) and sat. NaCl-solution (10 mL) and dried over $MgSO_4$. Column chromatography yielded the desired product.

In case of carboxylic acids as products, an alkaline work up is not possible. Therefore, the reaction mixture was directly poured onto water (80 mL) and extracted with EtOAc (3 x

20 mL). The aqueous phase was acidified with NH_4Cl-solution (20 mL), extracted with EtOAc (3 x 20 mL) and dried over $MgSO_4$. Removing the solvent under reduced pressure yielded the desired product.

Cross metathesis of terminal alkenes (GP2)

Under an inert atmosphere (N_2), a terminal alkene (538 mg, 1.55 mmol) and (2*E*,4*E)*-2-methy2-2,4-hexadienoate **15** or (2*E*,4*E*)-2-methylhexa-2,4-dienoic acid **9** (260 mg, 1.86 mmol) were dissolved in dichloromethane (40 mL) and Grubbs-II catalyst (65.8 mg, 0.08 mmol) was added and refluxed for 18 h. The reaction mixture was filtered over a pad of silica and washed with EtOAc (100 mL). Column chromatography yielded the desired product.

Saponification of methyl esters (GP3)

A methyl ester (140 mg, 0.31 mmol) was dissolved in demin. water (5 mL) and THF (5 mL) and potassium hydroxide (246 mg, 4.38 mmol) was added in portions at 0 °C and stirred for 3 d. The reaction mixture was acidified with conc. HCl (pH = 1) and extracted with EtOAc (3 x 50 mL). The organic phase was dried over $MgSO_4$ and the solvent was removed over reduced pressure. The crude product was purified *via* preparative HPLC on a C-18 reversed phase column.

Yamaguchi macrolactonization (GP4)

Under an inert atmosphere (N_2), a seco acid (50.0 mg, 0.12 mmol) was dissolved in dry THF (10 mL) and freshly distilled NEt_3 (20.0 µL, 14.0 mg, 0.14 mmol) was added dropwise at rt. After stirring for 15 min, 2,4,6-trichlorobenzoyl chloride (27.1 µL, 42.2 mg, 0.17 mmol) was added dropwise and the reaction mixture was stirred for an additional 18 h overnight. The precipitate was filtered over celite and washed with dry THF (20 mL). The solvent was removed under reduced pressure and the formed anhydride was dissolved in dry benzene (80 mL) or DMF (40 mL) and 4-dimethylaminopyridine (84.5 mg, 0.69 mmol) was added at once. After stirring for 18 h the reaction mixture was filtered over a pad of silica and column chromatography of the crude product yielded the desired product.

Coupling of arylbromides with aldehyde (S5) (GP5)

Under inert atmosphere (N_2), an arylbromide (291 mg, 1.78 mmol) was dissolved in dry THF (20 mL) and *n*-butyllithium (126 mg, 1.96 mmol, 2.5 M in hexane) was added dropwise at -78 °C. After stirring for 30 min, (*R*)-2-methylhex-5-enal **S9** (200 mg, 1.78 mmol), dissolved in dry THF (20 mL), was added dropwise at the same temperature, warmed to rt and stirred for an additional hour. The reaction mixture was poured onto sat. NH_4Cl-solution (50 mL) and extracted with EtOAc (3 x 50 mL). Column chromatography yielded the desired product.

13.3 Synthesis of tosylated oxazole precursors

Hex-5-enoic acid (S33)

Cyclohexanone (41.1 mL, 39.0 g, 0.42 mol) was dissolved in MeOH (40 mL) at 15 °C and H_2O_2 (89.3 mL, 99.1 g, 0.87 mol, 30 %) was added slowly. After completion and stirring for 30 min, the reaction mixture was added dropwise to a solution of $FeSO_4 \cdot 7\,H_2O$ (110 g, 0.40 mol) and $CuSO_4 \cdot 5\,H_2O$ (99.2 g, 0.40 mol) in water (750 mL), stirred for 3 h and the resulting dark brown mixture was extracted with diethyl ether (3 x 150 mL). The combined organic layer was extracted with NaOH-solution (3 x 75 mL, 6M) and the alkaline extract was brought to pH = 1 with 20% H_2SO_4 (ca. 150 mL) and again extracted with diethyl ether (3 x 75 mL). The organic phase was washed with demin. water (100 mL) and sat. NaCl-solution (100 mL), dried over $MgSO_4$. The solution was concentrated and then the residue was distilled in vacuo (110 °C / 21 mbar) yielding the desired product Hex-5-enoic acid **S33** (6.95 g, 60.9 mmol, 16 %) as colourless oil.

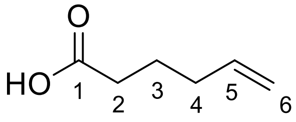

S33

$C_6H_{10}O_2$

114.14

R_f = 0.44 (petroleum ether / EtOAc = 5 : 1, molybdatophosphoric acid stain).

^{1}H NMR (400 MHz, $CDCl_3$): δ = 1.72 – 1.81 (m, 2H, 3-H), 2.10 – 2.18 (m, 2H, 4-H), 2.39 (t, J = 7.5 Hz, 2H, 2-H), 4.98 – 5.12 (m, 2H, 6-H), 5.74 – 5.87 (m, 1H, 5-H), 11.40 (br s, 1H, COOH) ppm.

^{13}C NMR (100 MHz, $CDCl_3$): δ = 23.7 (C-3), 32.9 (C-4), 33.3 (C-2), 115.5 (C-6), 137.5 (C-5), 180.0 (C-1) ppm.

The spectroscopic data is in agreement with the literature[27].

(*R*)-3-(Hex-5-enoyl)-4-isopropyl-5,5-dimethyloxazolidin-2-one (S34)

Under an inert atmosphere (N_2), (*R*)-4-Isopropyl-5,5-dimethyloxazolidin-2-one **11** (8.50 g, 54.1 mmol) was dissolved in dry THF (120 mL) cooled to -78 °C and *n*-butyllithium (26.0 mL, 8.47 g, 64.9 mmol, 2.5 M in hexane) was added dropwise. In another flask and under an inert atmosphere (N_2), Hex-5-enoic acid **S33** (7.41 g, 64.9 mmol) was dissolved in dry THF (80 mL) and pivaloyl chloride (8.65 mL, 8.47 g, 64.9 mmol) and NEt_3 (12.8 mL, 9.30 g, 54.1 mmol) were added dropwise at 0° C. The reaction mixture was stirred for 30 min and then added dropwise to the first prepared mixture. After stirring for 30 min at 0 °C and for 90 min at rt, the reaction mixture was added to sat. NH_4Cl-solution (200 mL) and extracted with EtOAc (3 x 150 mL). The combined organic phases were washed with sat. $NaHCO_3$-solution (150 mL), sat. NH_4Cl-solution (150 mL) and sat. NaCl-solution (150 mL), dried over $MgSO_4$ and the solvent removed under reduced pressure. Column chromatography on silica (petroleum ether / EtOAc 15 : 1) yielded the desired product (*R*)-3-(Hex-5-enoyl)-4-isopropyl-5,5-dimethyloxazolidin-2-one **S34** (12.2 g, 48.1 mmol, 93 %) as colourless solid.

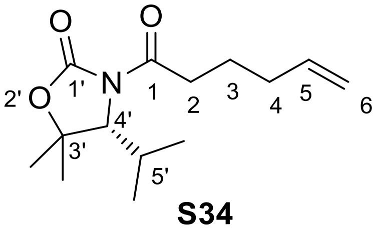

S34

R_f = 0.31 (petroleum ether / EtOAc = 10 : 1, molybdatophosphoric acid stain).

^{1}H NMR (400 MHz, $CDCl_3$): δ = 0.94 (d, J = 6.8 Hz, 3H, 5'-CH_3), 1.02 (d, J = 7.0 Hz, 3H, 5'-CH_3), 1.37 (s, 3H, 3'-CH_3), 1.50 (s, 3H, 3'-CH_3), 1.70 – 1.88 (m, 2H, 3-H), 2.06 – 2.19 (m, 3H, 4-H, 5'-H), 2.83 – 3.08 (m, 2H, 2-H), 4.15 (d, J = 3.4 Hz, 1H, 4'-H), 4.93 – 5.11 (m, 2H, 6-H), 5.73 – 5.89 (m, 1H, 5-H) ppm.

^{13}C NMR (100 MHz, $CDCl_3$): δ = 17.1 (5'-CH_3), 21.4 (5'-CH_3), 21.5 (3'-CH_3), 23.8 (C-3), 28.8 (3'-CH_3), 29.6 (C-5'), 33.1 (C-4), 34.8 (C-2), 66.3 (C-4'), 82.7 (C-3’), 115.3 (C-6), 137.9 (C-5), 153.6 (C-1'), 173.7 (C-1) ppm.

The spectroscopic data is in agreement with the literature[28].

(*R*)-4-Isopropyl-5,5-dimethyl-3-((*R*)-2-methylhex-5-enoyl)oxazolidin-2-one (12)

Under an inert atmosphere (N_2), (*R*)-3-(Hex-5-enoyl)-4-isopropyl-5,5-dimethyloxazolidin-2-one **S34** (9.25 g, 36.5 mmol) was dissolved in dry THF and sodium bis(trimethylsilyl)amide (36.5 mL, 6.70 g, 36.5 mmol, 1M in dry THF) was added dropwise at -78 °C. After stirring for 1 h, iodomethane (11.4 mL, 25.9 g, 183 mmol) was added. The reaction mixture was stirred for 1 h at -78 °C and 1 h at rt before adding to sat. NH_4Cl-solution (100 mL) and extracting with EtOAc (3 x 100 mL). The combined organic phases were washed with sat. $NaHCO_3$-solution (100 mL), sat. NH_4Cl-solution (100 mL) and sat. NaCl-solution (100 mL), dried over $MgSO_4$ and the solvent removed under reduced pressure. Column chromatography on silica (petroleum ether / EtOAc 30 : 1 → 10 : 1) yielded the desired product (*R*)-4-isopropyl-5,5-dimethyl-3-((*R*)-2-methylhex-5-enoyl)oxazolidin-2-one **12** (5.20 g, 19.5 mmol, 53 %) as colourless solid.

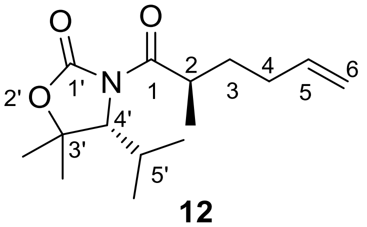

12

R_f = 0.29 (petroleum ether / EtOAc = 5 : 1, molybdatophosphoric acid stain).

^{1}H NMR (400 MHz, $CDCl_3$): δ = 0.95 (d, J = 6.8 Hz, 3H, 5'-CH_3), 1.01 (d, J = 7.0 Hz, 3H, 5'-CH_3), 1.26 (d, J = 6.9 Hz, 2H, 2-CH_3), 1.37 (s, 6H, 3'-CH_3), 1.39 – 1.48 (m, 1H, 3-H_A), 1.40 – 1.50 (s, 3H, 3'-CH_3), 1.72 – 1.95 (m, 1H, 3-H_B), 1.99 - 2.10 (m, 2H, 4-H), 2.12 – 2.20 (m, 1H, 5'-H), 3.71 – 3.85 (m, 1H, 2-H), 4.18 (d, J = 3.3 Hz, 1H, 4'-H), 4.89 – 5.05 (m, 2H, 6-H), 5.69 – 5.86 (m, 1H, 5-H) ppm.

^{13}C NMR (100 MHz, $CDCl_3$): δ = 17.0 (5'-CH_3), 18.4 (2-CH_3), 21.4 (5'-CH_3), 21.5 (3'-CH_3), 28.7 (3'-CH_3), 29.6 (C-5'), 31.6 (C-4), 32.2 (C-3), 37.3 (C-2), 66.1 (C-4'), 82.5 (C-3'), 114.9 (C-6), 138.1 (C-5), 153.2 (C-1'), 177.5 (C-1) ppm.

The spectroscopic data is in agreement with the literature[28].

(*R*)-2-Methylhex-5-en-1-ol (S35)

Under an inert atmosphere (N_2), (*R*)-4-Isopropyl-5,5-dimethyl-3-((*R*)-2-methylhex-5-enoyl)-oxazolidin-2-one **12** (1.70 g, 6.36 mmol) was dissolved in dry THF (55 mL) and $LiAlH_4$ (1.09 g, 28.1 mmol) was added in portion at 0 °C. After stirring for 2 h, demin. water (2.2 mL) and NaOH-solution (2.2 mL, 2M) was added dropwise. The reaction mixture was filtered over celite and the solvent removed under reduced pressure. Column chromatography on silica (petroleum ether / EtOAc 10 : 1) yielded the desired product (*R*)-2-methylhex-5-en-1-ol **S35** (500 mg, 4.38 mmol, 69 %) as colourless oil.

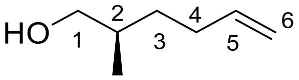

S35

$C_7H_{14}O$

114.19

R_f = 0.23 (petroleum ether / EtOAc = 10 : 1, molybdatophosphoric acid stain)

^1H NMR (300 MHz, $CDCl_3$): δ = 0.93 (d, *J* = 6.7 Hz, 3H, 2-CH_3), 1.12 – 1.35 (m, 2H, 3-H_A, OH), 1.46 – 1.59 (m, 1H, 3-H_B), 1.59 – 1.72 (m, 1H, 2-H), 1.98 – 2.21 (m, 2H, 4-H), 3.41-3.48 (m, 1H, 1-H_A), 3.49 - 3.55 (m, 1H, 1-H_B), 4.92 – 5.07 (m, 2H, 6-H), 5.75 – 5.88 (m, 1H, 5-H) ppm.

^{13}C NMR (100 MHz, $CDCl_3$): δ = 16.4 (2-CH_3), 31.2 (C-3), 32.3 (C-4), 35.2 (C-2), 68.2 (C-1), 114.4 (C-6), 138.9 (C-5) ppm.

The spectroscopic data is in agreement with the literature[28].

(*R*)-2-Methylhex-5-enal (S9)

Under an inert atmosphere (N_2), mole sieve (4.00 g, 3 Å) and *N*-methylmorpholine-*N*-oxide (472 mg, 4.03 mmol) were dissolved in dichloromethane (20 mL) and (*R*)-2-Methylhex-5-en-1-ol **S35** (230 mg, 1.93 mmol) was added at room temperature. After stirring for 30 min tetrapropylammonium perruthenate (42.5 mg, 0.12 mmol) was added, stirred for another 24 h, filtered over a pad of silica and washed with dichloromethane (100 ml). Removing the solvent under reduced pressure yielded the desired product (*R*)-2-methylhex-5-enal **S9** (216 mg, 1.93 mmol, quant.) als colourless oil. Please note, removing dichloromethane under minimal pressure leads to yield loss due to the high volatility of **S5**. Therefore, only use reduced pressure of >200 mbar.

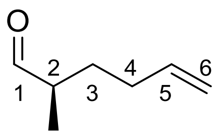

S9

$C_7H_{12}O$

112.17

^{1}H NMR (400 MHz, $CDCl_3$): δ = 1.11 (d, J = 7.0 Hz, 3H, 2-CH_3), 1.39 – 1.51 (m, 1H, 3-H_A), 1.77 – 1.89 (m, 1H, 3-H_B), 2.01 – 2.18 (m, 2H, 4-H), 2.30 – 2.44 (m, 1H, 2-H), 4.92 – 5.10 (m, 2H, 6-H), 5.71 – 5.85 (m, 1H, 5-H), 9.63 (d, J = 2.0 Hz, 1H, 1-H) ppm.

^{13}C NMR (100 MHz, $CDCl_3$): δ = 13.2 (2-CH_3), 29.6 (C-3), 31.0 (C-4), 45.6 (C-2), 115.4 (C-6), 137.7 (C-5), 205.0 (C-1) ppm.

The spectroscopic data is in agreement with the literature[29].

5-methyl-4-tosyl oxazole (13)

Under an inert atmosphere (N_2), *p*-toluenesulfonylmethyl isocyanide (2.26 g, 11.6 mmol) was dissolved in dry THF (40 mL) and at -78 °C *n*-butyllithium (815 mg, 12.7 mmol, 2.5 M in hexane) was added and stirred for 15 min. Acetic anhydride (1.20 mL, 1.30 mg, 12.7 mmol) was added and stirred for 1 h at -78 °C and 2 h at rt. The reaction mixture was added to demin. water (100 mL), extracted with EtOAc (3 x 50 mL) and the combined organic layers were dried over $MgSO_4$. After removal of the solvent under reduced pressure, the resulting solid was dissolved in dichloromethane (50 mL), filtered over a pad of siliva and washed with EtOAc (100 mL). Concentrating the solvent under reduced pressure yielded 5-methyl-4-tosyl oxazols **13** (2.634 mg, 11.1 mmol, 96 %) as colourless solid.

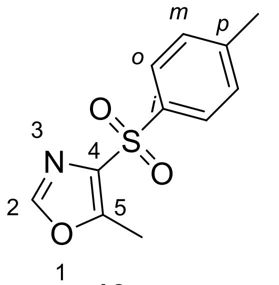

13

$C_{11}H_{11}NO_3S$

237.27

R_f = 0.43 (Petroleum ether / EtOAc 3 : 1)

^{1}H NMR (400 MHz, $CDcCl_3$): δ = 2.42 (s, 3H, *p*-CH_3), 2.70 (s, 3H, 5-CH_3), 7.34 (d, J = 8.0 Hz, 2H, *m*-H), 7.70 (s, 1H, 2-H), 7.91 (d, J = 8.0 Hz, 2H, *o*-H) ppm.

^{13}C NMR (101 MHz, $CDCl_3$): δ = 11.4 (5-CH_3), 21.7 (*p*-CH_3), 128.0 (C-*m*), 129.9 (C-*o*), 135.7 (C-5), 137.4 (C-*i*), 144.9 (C-*p*), 149.4 (C-2), 153.5 (C-4) ppm.

The spectroscopic data is in agreement with the literature[30].

(*R*)-3-Methyl-1-(4-tosyloxazol-5-yl)hept-6-en-2-one (14)

Under an inert atmosphere (N_2), 5-Methyl-4-tosyloxazole **13** (7.94 g, 33.5 mmol) was dissolved in dry THF (300 mL) and *n*-butyllithium (4.29 g, 66.9 mmol, 2.5 M in hexane) was added dropwise at -78 °C. After stirring for 30 min, (*R*)-4-isopropyl-5,5-dimethyl-3-((*R*)-2-methylhex-5-enoyl)oxazolidin-2-one **12** (7.16 mg, 26.8 mmol), dissolved in abs. THF (80 mL), was added and the cooling bath was covered with aluminium foil to allow the reaction mixture to warm up slowly to rt over night. After a reaction time of 20 h, the mixture was added to sat. $NaNH_4$-solution (300 mL), extracted with EtOAc (3 x 100 mL) and dried over $MgSO_4$. Column

chromatography on silica (petroleum ether / EtOAc 10 : 1) yielded (*R*)-3-methyl-1-(4-tosyloxazol-5-yl)hept-6-en-2-one **14** (5.20 g, 15.0 mmol, 56 %) as yellow oil.

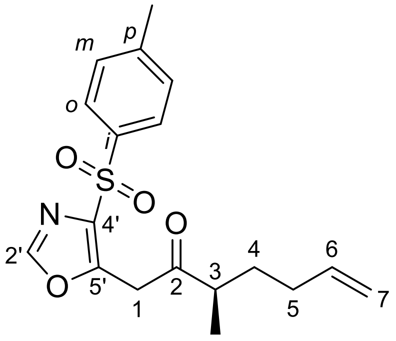

14

$C_{18}H_{21}NO_4S$

347.43

R_f = 0.43 (petroleum ether / EtOAc 3 : 1)

^{1}H NMR (400 MHz, $CDCl_3$): δ = 1.21 (d, J = 7.0 Hz, 3H, 3-CH_3), 1.45 – 1.59 (m, 1H, 4-H_A), 1.81 – 1.98 (m, 1H, 4-H_B), 2.01 – 2.19 (m, 2H, 5-H), 2.42 (s, 3H, *p*-CH_3), 2.64 – 2.80 (m, 1H, 3-H), 4.23 – 4.42 (m, 2H, 1-H), 4.96 – 5.11 (m, 2H, 7-H), 5.79 (m, 1H, 6-H), 7.34 (d, J = 8.0 Hz, 2H, *m*-H), 7.78 (s, 1H, 2'-H), 7.84 – 7.95 (m, 2H, *o*-H) ppm.

^{13}C NMR (101 MHz, $CDCl_3$): δ = 16.2 (3-CH_3), 21.7 (*p*-CH_3), 31.2 (C-5), 31.7 (C-4), 38.1 (C-1), 45.6 (C-3), 115.6 (C-7), 128.2 (C-*o*), 129.9 (C-*m*), 136.7 (C-*i*), 137.6 (C-5'), 137.7 (C-6), 145.1 (C-*p*), 150.2 (C-4'), 150.4 (C-2'), 206.6 (C-2) ppm.

FT-IR (ATR): $\tilde{\nu}$ = 3136 (w), 2932 (w), 2178 (w), 1720 (m), 1596 (m), 1514 (w), 1457 (w), 1400 (w), 1326 (s), 1305 (m), 1246 (w), 1148 (vs), 1087 (w), 917 (w), 815 (w), 798 (m), 658 (m), 631 (w), 600 (s), 537 (m) cm^{-1}.

MS (ESI): *m / z* for $C_{18}H_{21}NO_4S$ calc.: 370.1 $[M+Na]^+$, found: 370.1.

HRMS (ESI): *m / z* for $C_{18}H_{21}NO_4S$ calc.: 370.1083 $[M+Na]^+$, found: 370.1090.

(3*R*)-3-Methyl-1-(4-tosyloxazol-5-yl)hept-6-en-2-ol (Ts-10)

Under an inert atmosphere (N_2), (*R*)-3-Methyl-1-(4-tosyloxazol-5-yl)hept-6-en-2-one **14** (5.20 g, 15.0 mmol) was dissolved in dry THF (40 mL) and $NaBH_4$ (1.14 g, 30.0 mmol) was added at 0 °C. After 30 min the reaction mixture was warmed to room temperature and further stirred for 20 h, before adding HCl-solution (60 mL, 1 M). Extraction with dichloromethane (3 x 30 mL), drying over $MgSO_4$ and subsequent column chromatography on silica (petroleum ether / EtOAc 3 : 1) yielded the diastereomeric mixture Ts-**10** (4.00 g, 11.5 mmol, 77 %, *syn/anti* 46 : 54 (according to ^{1}H NMR)) as colourless oil.

The separation of the diastereomeric mixture Ts-**10** (*syn / anti* 46 : 54) was performed *via* preparative HPLC on a Refill-column Orbit 100 Sil 5 µm (250 x 20 mm) with a flow rate of 10 $mLmin^{-1}$ and a solvent gradient of petroleum ether / EtOAc 70 : 30 to 60 : 40. The diastereomers *syn*-Ts-**4a** and *anti*-Ts-**4a** were received in 35 % (1.84 g, 5.2 mmol) and 42 % (2.16 g, 6.18 mmol) respectively.

(2*R*,3*R*)-3-Methyl-1-(4-tosyloxazol-5-yl)hept-6-en-2-ol (*syn*-Ts-10)

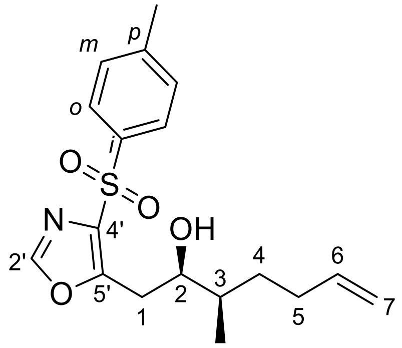

syn-Ts-**10**

$C_{18}H_{23}NO_4S$

349.44

R_f = 0.69 (petroleum ether / EtOAc 1 : 1).

R_t = 37.4 min.

^{1}H NMR (400 MHz, $CDCl_3$): δ = 1.02 (d, J = 6.6 Hz, 3H, 3-CH_3), 1.29 – 1.43 (m, 1H, 4-H_A), 1.42 – 1.70 (m, 2H, 3-H, 4-H_B), 2.02 – 2.14 (m, 1H. 5-H_A), 2.17 (p, J = 6.4 HZ, 1H. 5-H_B), 2.43 (s, 3H, *p*-CH_3), 3.18 (dd, J = 14.8 Hz, 3.6 Hz, 1H, 1-H_A), 3.32 (dd, J = 14.8 Hz, 9.5 Hz, 1H, 1-H_B), 3.92 (d, J = 10.1 HZ, 1H, 2-H), 4.91 – 5.10 (m, 2H, 7-H), 5.72 – 5.92 (m, 1H, 6-H), 7.34 (d, J = 8.1 Hz, 2H, *m*-H), 7.75 (s, 1H, 2'-H), 7.87 – 7.97 (m, 2H, *o*-H) ppm.

^{13}C NMR (101 MHz, $CDCl_3$) δ = 13.5 (3-CH_3), 21.7 (*p*-CH_3), 31.0 (C-1), 31.3 (C-5), 32.1 (C-4), 38.1 (C-3), 73.6 (C-2), 114.8 (C-7), 128.2 (C-*o*), 129.9 (C-*m*), 137.0 (C-5'), 138.5 (C-6), 145.0 (C-*p*), 149.8 (C-2'), 155.0 (C-4') ppm.

FT-IR (ATR): $\tilde{\nu}$ = 3531 (w), 3130 (w), 3071 (w), 2920 (w), 1736 (w), 1640 (w), 1594 (m), 1517 (m), 1495 (w), 1459 (w), 1380 (w), 1322 (s), 1305 (m), 1245 (m), 1215 (w), 1145 (vs), 1085 (m), 1061 (m), 1017 (m), 997 (m), 910 (m), 861 (w), 814 (m), 733 (w), 705 (m), 696 (m), 660 (vs), 598 (vs), 539 (vs) cm^{-1}.

MS (ESI): *m / z* for $C_{18}H_{23}NO_4S$ calc.: 372.1 $[M+Na]^+$, found: 372.1.

HRMS (ESI): *m / z* for $C_{18}H_{23}NO_4S$ calc.: 372.1240 $[M+Na]^+$, found: 372.1248.

(2*S*,3*R*)-3-Methyl-1-(4-tosyloxazol-5-yl)hept-6-en-2-ol (*anti*-Ts-10)

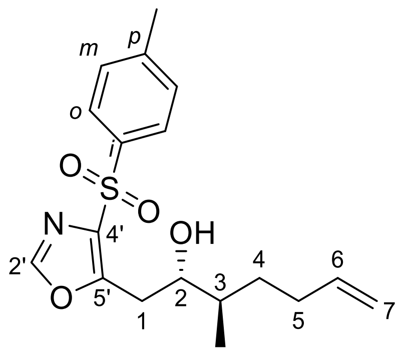

anti-Ts-**10**

$C_{18}H_{23}NO_4S$

349.44

R_f = 0.69 (petroleum ether / EtOAc 1 : 1).

R_t = 40.5 min.

^{1}H NMR (400 MHz, $CDCl_3$): δ = 1.03 (d, J = 6.6 Hz, 3H, 3-CH_3), 1.27 – 1.38 (m, 1H, 4-H_A), 1.60 – 1.76 (m, 2H, 3-H, 4-H_B), 2.00 – 2.13 (m, 1H, 5-H_A), 2.13 – 2.29 (m, 1H, 5-H_B), 2.43 (s, 3H, *p*-CH_3), 3.15 – 3.32 (m, 2H, 1-H), 3.77 – 3.89 (m, 1H, 2-H), 4.86 – 5.13 (m, 2H, 7-H), 5.83 (ddt, J = 16.9 Hz, 10.2 Hz, 6.6 Hz, 1H, 6-H), 7.34 (d, J = 8.1 Hz, 2H *m*-H), 7.75 (s, 1H, 2'-H), 7.93 (d, J = 8.2 Hz, 2H, *o*-H) ppm.

^{13}C NMR (101 MHz, $CDCl_3$): δ = 14.9 (3-CH_3), 21.7 (*p*-CH_3), 30.2 (C-1), 31.1 (C-5), 31.2 (C-4), 38.6 (C-3), 74.4 (C-2), 114.8 (C-7), 128.2 (C-*o*), 129.9 (C-*m*), 136.8 (C-*i*), 137.0 (C-5'), 138.6 (C-6), 145.0 (C-*p*), 149.8 (C-2'), 155.1 (C-1) ppm.

FT-IR (ATR): $\tilde{\nu}$ = 3534 (w), 3133 (w), 3072 (w), 2924 (w), 1640 (w), 1594 (m), 1517 (m), 1495 (w), 1456 (w), 1402 (w), 1381 (w), 1322 (s), 1305 (m), 1245 (w), 1218 (w), 1146 (vs), 1085 (m), 1063 (m), 1017 (w), 996 (w), 911 (w), 860 (w), 814 (m), 743 (w), 706 (m), 697 (m), 662 (s), 600 (vs), 539 (s) cm^{-1}.

MS (ESI): *m / z* for $C_{18}H_{23}NO_4S$ calc.: 372.1 $[M+Na]^+$, found: 372.1.

HRMS (ESI): *m / z* for $C_{18}H_{23}NO_4S$ calc.: 372.1240 $[M+Na]^+$, found: 372.1250.

The determination of the absolute configuration of the alcohols was performed via Mosher's method[1].

(3*R*)-3-Methyl-1-(oxazol-5-yl)hept-6-en-2-ol (10)

According to GP1, (3*R*)-3-Methyl-1-(4-tosyloxazol-5-yl)hept-6-en-2-ol Ts-**10** (140 mg, 0.40 mmol, dr (*syn*/*anti*) = 46 : 54), Na_2HPO_4 (226 mg, 1.60 mmol) and sodium amalgam (2 x 460 mg, 2.00 mmol, 10 %), dissolved in THF (5 mL) and EtOH (5 mL), were treated in an ultrasound bath. Column chromatography on silica (petroleum ether / EtOAc 5 : 1) yielded the desired product (3*R*)-3-Methyl-1-(oxazol-5-yl)hept-6-en-2-ol **10 (**64 mg, 0.33 mmol, 83 %, dr (*syn*/*anti*) = 59 : 41 (according to ^{1}H NMR)) as colourless oil.

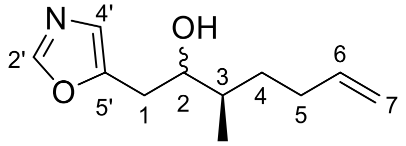

$C_{11}H_{17}NO_2$

195.26

10

R_f = 0.40 (petroleum ether / EtOAc = 1 : 1, molybdatophosphoric acid stain).

$[\alpha]_D^{20}$ = + 3.2° (c = 1.0, $CHCl_3$).

^{1}H NMR (400 MHz, $CDCl_3$): δ = 1.02 (d, J = 7.0 Hz, 3H, 3-CH_3), 1.20-1.38 (m, 1H, 4-H_a), 1.55-1.69 (m, 2H, 3-H, 4-H_b), 1.97-2.24 (m, 2H, 5-H), 2.74-2.91 (m, 2H, 1-H), 3.74-3.89 (m, 1H, 2-H), 4.93-5.06 (m, 2H, 7-H), 5.74-5.87 (m, 1H, 6-H), 6.89 (s, 2H, 4'-H), 7.08 (s, 1H, 2'-H) ppm.

^{13}C NMR (100 MHz, $CDCl_3$): δ = 13.4 (3-CH_3), 30.9 (C-1), 31.3 (C-5), 32.3 (C-4), 37.3 (C-3), 73.0 (C-2), 114.7 (C-7), 123.6 (C-4'), 129.6 (C-5'), 138.5 (C-6), 150.5 (C-2') ppm.

FT-IR (ATR): $\tilde{\nu}$ = 3379 (m), 3134 (w), 3077 (w), 2962 (s), 2923 (s), 2160 (w), 2004 (w), 1970 (w), 1722 (w), 1640 (m), 1601 (m), 1511 (vs), 1460 (m), 1436 (m), 1379 (m), 1323 (m), 1260 (m), 1213 (m), 1149 (s), 1105 (s), 1086 (s), 1052 (s), 994 (s), 964 (vs), 910 (vs), 821 (s), 647 (vs), 587 (m), 515 (w) cm^{-1}.

MS (ESI): *m/z* (%) = 685.4 (4), 631.1 (11), 537.2 (63), 459.2 (31), 440.2 (100), 370.1 (14), 328.1 (77), 285.1 (45), 218.1 (37), 201.0 (15), 139.0 (8), 122.0 (5).

HRMS (ESI): calc. für $[C_{11}H_{17}NO_2Na]^+$ 218.1151, gef. 218.1143 $[M + Na]^+$.

MS (ESI): *m / z* for $C_{11}H_{17}NO_2$ calc.: 218.1 $[M+Na]^+$, found: 218.1.

HRMS (ESI): *m / z* for $C_{11}H_{17}NO_2$ calc.: 218.1151 $[M+Na]^+$, found: 218.1143.

(3*R*)-3-Methyl-1-(oxazol-5-yl)hept-6-en-2-yl-(2*E*,4*E*)-2-methylhexa-2,4dienoat (S8)

Under an inert atmosphere (N_2), (2*E*,4*E*)-2-methylhexa-2,4-dienoic acid **9** (45.0 mg, 0.36 mmol) was dissolved in dry THF (10 mL) and NEt_3 (50.0 µL, 36.0 mg, 0.36 mmol) was added dropwise. After 10 min, 2,4,6-trichlorobenzoyl chloride (60.0 µL, 88.0 mg, 0.36 mmol) was added dropwise and the reaction mixture was stirred for additional 6 h, filtered over celite, washed with dry THF (10 mL) and concentrated under reduced pressure. The formed anhydride was dissolved in dry DMF (10 mL) and (3*R*)-3-methyl-1-(oxazol-5-yl)hept-6en-2-ol **10** (70.0 mg, 0.36 mmol, dr (*syn*/*anti*) = 46 : 54), dissolved in DMF (10 mL), and 4-dimethylaminopyridine (88.0 mg, 0.72 mmol) were added and stirred for 1 h at 50 °C. The solvent was removed under reduced pressure and the crude product was dissolved in dichloromethane (10 mL), washed with HCl-solution (10 mL, 1 M), sat. $NaHCO_3$-solution (10 mL) und water (10 mL) and dried over $MgSO_4$. Column chromatography on silica (petroleum ether / EtOAc 5 : 1) yielded the desired product (3*R*)-3-methyl-1-(oxazol-5-yl)hept-6-en-2-yl-(2*E*,4*E*)-2-methylhexa-2,4-dienoat **S8 (**74.0 mg, 0.24 mmol, 68 %, dr (*syn* / *anti*) = 59 : 41 (according to ^1H NMR)) as colourless oil.

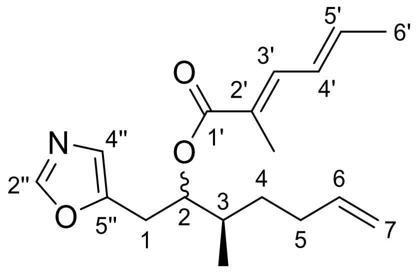

$C_{18}H_{25}NO_3$

303.40

S8

R_f = 0.39 (petroleum ether / EtOAc = 5 : 1, molybdatophosphoric acid stain).

^1H NMR (500 MHz, $CDCl_3$): δ = 0.96-1.00 (m, 3H, 3-CH_3), 1.20-1.31 (m, 1H, 4-H_a), 1.49-1.62 (m, 1H, 4-H_b), 1.71-1.84 (m, 1H, 3-H), 1.86-1.88 (m, 3H, 6'-H), 1.88-1.91 (m, 3H, 2'-CH_3), 1.97-2.22 (m, 2H, 5-H), 2.91-3.05 (m, 2H, 1-H), 4.92-5.04 (m, 2H, 7-H), 5.09-5.20 (m, 1H, 2-H), 5.71-5.83 (m, 1H, 6-H), 6.05-6.14 (m, 1H, 5'-H), 6.28-6.39 (m, 1H, 4'-H), 6.78-6.83 (m, 1H, 4"-H), 7.09-7.14 (m, 1H, 3'-H), 7.74-7.78 (m, 1H, 2"-H) ppm.

^{13}C NMR (125 MHz, $CDCl_3$): δ = 12.5 (2'-CH_3), 14.2 (3-CH_3), 18.9 (C-6'), 27.9 (C-1), 31.2 (C-5), 32.1 (C-4), 35.4 (C-3), 75.1 (C-2), 114.8 (C-7), 123.7 (C-4''), 124.6 (C-2'), 127.3 (C-4'), 138.1 (C-5'), 138.3 (C-6), 138.9 (C-3'), 149.3 (C-5''), 150.4 (C-2''), 168.1 (C-1') ppm.

FT-IR (ATR): $\tilde{\nu}$ = 2932 (m), 2163 (w), 1702 (vs), 1641 (m), 1609 (w), 1510 (m), 1438 (w), 1386 (w), 1289 (m), 1232 (vs), 1166 (m), 1100 (vs), 970 (m), 914 (m), 825 (w), 743 (w), 647 (w) cm^{-1}.

MS (ESI): *m / z* for $C_{18}H_{25}NO_3$ calc.: 326.2 $[M+Na]^+$, found: 326.2.

HRMS (ESI): *m / z* for $C_{18}H_{25}NO_3$ calc.: 326.1727 $[M+Na]^+$, found: 326.1721.

(3*R*)-3-Methyl-1-(4-tosyloxazol-5-yl)hept-6-en-2-yl (2*E*,4*E*)-2-methylhexa2,4-dienoate (Ts-S8)

Under an inert atmosphere (N_2), (2*E*,4*E*)-2-methylhexa-2,4-dienoic acid **9** (610 mg, 4.81 mmol) was dissolved in dry THF (20 mL) and NEt_3 (0.67 mL, 487 mg, 4.81 mmol) was added dropwise. After 10 min, 2,4,6-trichlorobenzoyl chloride (0.75 mL, 1.17 g, 4.81 mmol) was added dropwise and the reaction mixture was stirred for additional 6 h, filtered over celite, washed with dry THF (20 mL) and concentrated under reduced pressure. The formed anhydride was dissolved in dry DMF (10 mL) and (3*R*)-3-methyl-1-(4-tosyloxazol-5-yl)hept-6en-2-ol Ts-**10** (1.40 g, 4.00 mmol, dr (*syn*/*anti*) = 46 : 54), dissolved in DMF (20 mL), and 4-dimethylaminopyridine (0.98 g, 8.00 mmol) were added and stirred for 1 h at 50 °C. The solvent was removed under reduced pressure and the crude product was dissolved in dichloromethane (10 mL), washed with HCl-solution (20 mL, 1 M), sat. $NaHCO_3$-solution (20 mL) und water (20 mL) and dried over $MgSO_4$. Column chromatography on silica (petroleum ether / EtOAc 5 : 1) yielded the desired product (3*R*)-3-methyl-1-(4-tosyloxazol-5-yl)hept-6-en-2-yl-(2*E*,4*E*)-2-methylhexa-2,4-dienoat Ts-**S8** (823 mg, 1.84 mmol, 46 %, dr (*syn*/*anti*) = 59 : 41 (according to ^1H NMR)) as colourless oil.

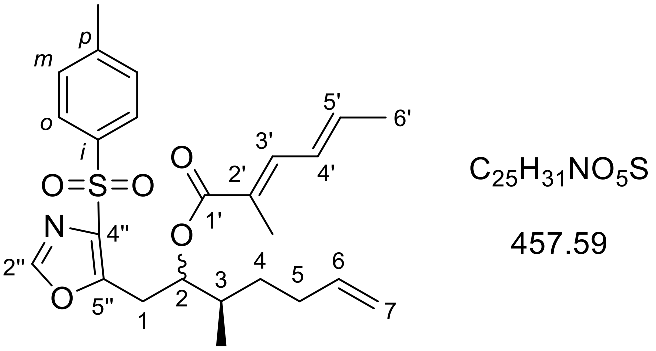

Ts-**S8**

R_f = 0.33 (petroleum ether / EtOAc = 5 : 1, molybdatophosphoric acid stain)

^1H NMR (700 MHz, $CDCl_3$): δ = 1.03-1.08 (m, 3H, 3-CH_3), 1.27-1.36 (m, 1H, 3-H), 1.76-1.94 (m, 2H, 4-H), 1.85 (d, *J* = 10.5 Hz, 3H, 6'-H), 1.86-1.89 (m, 3H, 2'-CH_3), 2.02-2.24 (m, 2H, 5-H), 2.41 (s, 3H, *p*-CH_3), 3.15-3.51 (m, 2H, 1-H), 4.92-5.06 (m, 2H, 7-H), 5.22-5.31 (m, 1H, 2-H), 5.72-5.84 (m, 1H, 6-H), 6.07-6.14 (m, 1H, 5'-H), 6.31-6.38 (m, 1H, 4'-H), 7.09-7.13 (m, 1H, 3'-H) 7.29-7.36 (m, 2H, *m*-H), 7.70-7.73 (m, 1H, 2''-H), 7.87-7.93 (m, 1H, *o*-H) ppm.

^{13}C NMR (175 MHz, $CDCl_3$): δ = 12.5 (2’-CH_3), 14.3 (3-CH_3), 18.9 (C-6’), 21.7 (*p*-CH_3), 27.0 (C-1), 31.2 (C-5), 32.0 (C-3), 36.0 (C-4), 74.2 (C-2), 114.9 (C-7), 124.4 (C-2’), 127.3 (C-4’), 128.1 (C-*o*), 129.8 (C-*m*), 136.7 (C-*i*), 137.0 (C-5”), 138.3 (C-6), 138.4 (C-5’), 139.1 (C-3’), 144.8 (C-*p*), 150.0 (C-2”), 153.5 (C-4”), 167.7 (C-1’) ppm.

FT-IR (ATR): $\tilde{\nu}$ = 3132 (w), 2971 (w), 2298 (w), 1700 (s), 1640 (m), 1595 (m), 1516 (w), 1494 (w), 1445 (w), 1386 (w), 1328 (s), 1305 (m), 1289 (s), 1227 (vs), 1147 (vs), 1087 (s), 1048 (m), 1018 (m), 996 (m), 970 (m), 930 (m), 913 (m), 841 (w), 814 (m), 744 (w), 705 (m), 661 (vs), 599 (vs), 539 (s) cm^{-1}.

MS (ESI): *m / z* for $C_{25}H_{31}O_5NS$ calc.: 458.2 $[M+H]^+$, found: 458.2.

HRMS (ESI): *m / z* for $C_{25}H_{31}O_5NS$ calc.: 458.1996 $[M+H]^+$, found: 458.1962.

Methyl (*R*,2*E*,4*E*)-2,8-dimethyl-9-oxo-10-(4-tosyloxazol-5-yl)deca-2,4-dienoate (17)

According to GP2, (*R*)-3-Methyl-1-(4-tosyloxazol-5-yl)hept-6-en2-one **14** (538 mg, 1.55 mmol) was treated with (2*E*,4*E)*-2-methy2-2,4-hexadienoate **15** (260 mg, 1.86 mmol) and Grubbs-II catalyst (65.8 mg, 0.08 mmol) in dichloromethane (40 mL). Column chromatography on silica (petroleum ether / EtOAc 5 : 1) yielded methyl-(*R*,2*E*,4*E*)-2,8-dimethyl-9-oxo-10-(4-tosyloxazol-5-yl)deca-2,4-dienoate 1**7** **(**420 mg , 0.94 mmol, 61 %) as colourless oil.

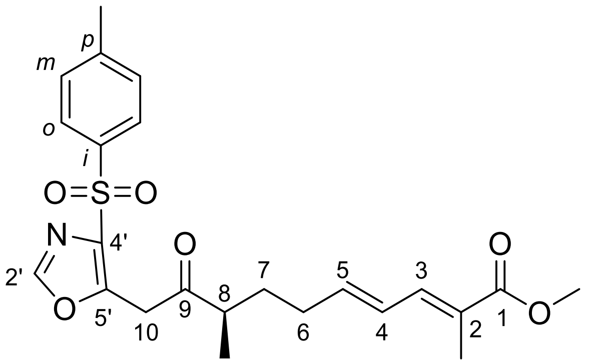

$C_{23}H_{27}NO_6S$

445.53

17

R_f = 0.22 (petroleum ether / EtOAc = 3 : 1, molybdatophosphoric acid stain)

^{1}H NMR (700 MHz, $CDCl_3$): δ = 1.24 (d, *J* = 7.0 Hz, 3H, 8-CH_3), 1.93 (s, 3H, 2-CH_3), 1.52-1.60 (m, 1H, 7-H_a), 1.94-1.98 (m, 1H, 7-H_b), 2.20-2.28 (m, 2H, 6-H), 2.43 (s, 3H, p-CH_3), 2.74 (q, *J* = 7.0 Hz, 1H, 8-H), 3.75 (s, 3H, O-CH_3), 4.28 (d, *J* = 17.5 Hz, 1H, 10-H_b), 4.37 (d, *J* = 17.5 Hz, 1H, 10-H_b), 6.03 (dt, *J* = 15.0 Hz, 7.3 Hz, 1H, 5-H), 6.39 (dd, *J* = 15.0 Hz, 11.3 Hz, 1H, 4-H), 7.16 (d, *J* = 11.3 Hz, 1H, 3-H), 7.35 (d, *J* = 8.0 Hz, 2H, *m*-H), 7.78 (s, 1H, 2’-H), 7.88 (d, *J* = 8.0 Hz, 2H, *o*-H) ppm.

^{13}C NMR (100 MHz, $CDCl_3$): δ = 12.6 (2-CH_3), 16.3 (8-Me), 21.7 (*p*-CH_3), 30.7 (C-6), 31.7 (C-7), 38.1 (C-10), 45.7 (C-8), 51.8 (OCH_3), 125.6 (C-2), 126.9 (C-4), 128.2 (C-*o*), 129.9 (C-*m*), 136.6 (C-*i*), 137.6 (C-5’), 138.3 (C-3), 141.2 (C-5), 145.2 (C-*p*), 150.0 (C-4’), 150.5 (C-2’), 169.0 (C-1), 206.4 (C-9) ppm.

FT-IR (ATR): $\tilde{\nu}$ = 3131 (w), 2931 (w), 1704 (s), 1638 (w), 1596 (m), 1513 (m), 1435 (m), 1323 (s), 1305 (m), 1245 (s), 1195 (w), 1145 (vs), 1107 (m), 1039 (m), 1018 (m), 977 (w), 934 (w), 647 (w), 815 (m), 751 (w), 697 (m), 660 (s), 601 (vs), 537 (s) cm^{-1}.

MS (ESI): *m / z* for $C_{24}H_{25}NO_6S$ calc.: 456.1 $[M+Na]^+$, found: 456.1.

HRMS (ESI): *m / z* for $C_{24}H_{25}NO_6S$ calc.: 456.1475 $[M+Na]^+$, found: 456.1440.

Methyl-(2*E*,4*E*,8*R*,9*R*)-9-hydroxy-2,8-dimethyl-10-(4-tosyloxazol-5-yl)deca-2,4-dienoate (*syn*-Ts-16)

According to GP2, (2*R*,3*R*)-3-Methyl-1-(4-tosyloxazol-5-yl)hept-6-en-2-ol *syn*-Ts-**10** (165 mg, 472 mmol) was treated with (2*E*,4*E)*-2-methy2-2,4-hexadienoate **15** (132 mg, 0.94 mmol) and Grubbs-II catalyst (60.1 mg, 70.8 µmol) in dichloromethane (15 mL). Column chromatography on silica (petroleum ether / EtOAc 3 : 1) yielded methyl-(2*E*,4*E*,8*R*,9*R*)-9-hydroxy-2,8-dimethyl-10-(4-tosyloxazol-5-yl)deca-2,4-dienoate *syn*-Ts-**16** (155 mg, 0.35 mmol, 73 %, dr (*syn*/*anti)*= 100 : 0 (according to ^{1}H NMR)) as colourless oil.

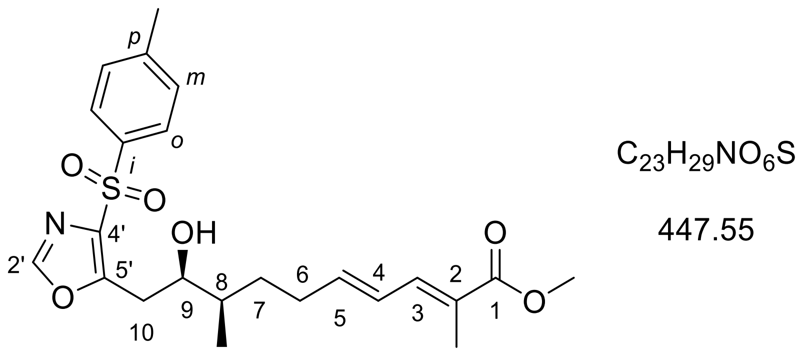

syn-Ts-**16**

$[\alpha]_D^{20} = +18.3°$ (c = 1.0, CH_2Cl_2).

R_f = 0.5 (petroleum ether / EtOAc 1 : 1).

^{1}H NMR (700 MHz, $CDCl_3$) δ = 1.03 (t, *J* = 5.5 Hz, 3H, 8-CH_3), 1.35 – 1.43 (m, 1H, 7-H_A), 1.58 – 1.72 (m, 2H, 7-H_B), 1.92 (s, 3H, 2-CH_3), 2.16 – 2.25 (m, 1H, 6-H_A), 2.27 – 2.35 (m, 1H, 6-H_B), 2.42 (s, 3H, *p*-CH_3), 3.14 – 3.20 (m, 1H, 10-H_A), 3.27 – 3.36 (m, 1H, 10-H_B), 3.73 – 3.75 (m, 3H, OCH_3), 3.86 – 3.96 (m, 1H, 9-H), 6.02 – 6.10 (m, 1H, 5-H), 6.34 – 6.40 (m, 1H, 4-H), 7.16 (d, *J* = 11.5 Hz, 1H, 3-H), 7.34 (d, *J* = 7.9 Hz, 2H, *m*-H), 7.75 (d, *J* = 3.1 Hz, 1H, 2'-H), 7.91 (d, *J* = 7.8 Hz, 2H, *o*-H) ppm.

^{13}C NMR (176 MHz, $CDCl_3$): δ = 12.6 (2-CH_3), 13.5 (8-CH_3), 21.7 (*p*-CH_3), 30.9 (C-10), 31.0 (C-6), 32.1 (C-7), 38.2 (C-8), 51.8 (OCH_3), 73.5 (C-9), 125.2 (C-2), 126.4 (C-4), 128.2 (C-*o*), 130.0 (C-*m*), 136.7 (C-*i*), 136.9 (C-5'), 138.7 (C-3), 142.4 (C-5), 145.1 (C-*p*), 149.9 (C-2'), 154.9 (C-4'), 169.1 (C-4), 171.2 (C-1) ppm.

FT-IR (ATR): $\tilde{\nu}$ = 3486 (br), 2930 (br), 1704 (s), 1638 (w), 1595 (w), 1516 (w), 1435 (w), 1325 (m), 1246 (m), 1147 (vs), 1106 (m), 976 (w), 814 (w), 751 (w), 697 (w), 662 (m), 600 (s), 540 (m) cm^{-1}.

MS (ESI): *m / z* for $C_{23}H_{29}NO_6S$ calc.: 470.16 [M+Na], found: 470.16.

HRMS (ESI): *m / z* for $C_{23}H_{29}NO_6S$ calc.: 470.1608 [M+Na], found: 470.1607.

Methyl-(2*E*,4*E*,8*R*,9*S*)-9-hydroxy-2,8-dimethyl-10-(4-tosyloxazol-5-yl)deca-2,4-dienoate (*anti*-Ts-16)

According to GP2, (2*S*,3*R*)-3-Methyl-1-(4-tosyloxazol-5-yl)hept-6-en-2-ol *anti*-Ts-**10** (165 mg, 472 mmol) was treated with (2*E*,4*E)*-2-methy2-2,4-hexadienoate **15** (132 mg, 0.94 mmol) and Grubbs-II catalyst (60.1 mg, 70.8 µmol) in dichloromethane (15 mL). Column chromatography on silica (petroleum ether / EtOAc 3 : 1) yielded methyl (2*E*,4*E*,8*R*,9*S*)-9-hydroxy-2,8-dimethyl-10-(4-tosyloxazol-5-yl)deca-2,4-dienoate *anti*-Ts-**16** **(**130 mg, 0.29 mmol, 62 %, dr (*syn*/*anti)*= 0 : 100 (according to ^{1}H NMR)) as colourless oil.

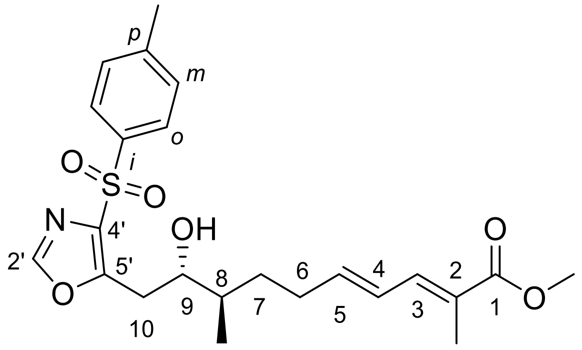

$C_{23}H_{29}NO_6S$

447.55

anti-Ts-**16**

$[\alpha]_D^{20} = -22.0°$ (c = 1.0, CH_2Cl_2).

R_f = 0.5 (petroleum ether / EtOAc 1 : 1).

^{1}H NMR (700 MHz, $CDCl_3$): δ 1.04 (d, *J* = 6.7 Hz, 3H, 8-CH_3), 1.30 – 1.44 (m, 1H, 7-H_A), 1.62 – 1.82 (m, 1H, 7-H_B), 1.93 (d, *J* = 1.5 Hz, 3H, 2-CH_3), 2.09 – 2.28 (m, 1H, 6-H_A), 2.28 – 2.43 (m, 1H. 6-H_B), 2.42 (s, 3H, *p*-CH_3), 3.25 (d, *J* = 6.1 Hz, 2H, 10-H), 3.75 (d, *J* = 1.9 Hz, 3H, OCH_3), 3.78 – 3.88 (m, 1H, 9-Me), 6.03 – 6.13 (m, 1H, 5-H), 6.33 – 6.44 (m, 1H, 4-H), 7.12 – 7.19 (m, 1H, 3-H), 7.34 (d, *J* = 8.0 Hz, 2H, *m*-H), 7.75 (s, 1H, 2'-H), 7.90 – 7.94 (m, 2H, *o*-H) ppm.

^{13}C NMR (176 MHz, $CDCl_3$): δ = 12.6 (2-CH_3), 15.0 (8-CH_3), 21.7 (*p*-CH_3), 30.3 (C-10), 30.8 (C-6), 31.2 (C-7), 38.7 (C-8), 51.8 (OCH_3), 74.4 (C-9), 125.1 (C-2), 126.3 (C-4), 128.3 (C-*o*), 130.0 (C-*m*), 136.8 (C-5'), 138.7 (C-3), 142.5 (C-5), 145.1 (C-*p*), 149.9 (C-2'), 154.9 (C-4'), 171.2 (C-1) ppm.

FT-IR (ATR): $\tilde{\nu}$ = 3510 (br), 3130 (w), 2925 (m), 2855 (w), 1704 (m), 1637 (w), 1595 (w), 1515 (w), 1435 (w), 1387 (w), 1325 (m), 1304 (m), 1248 (s), 1147 (vs), 1106 (m), 1085 (m), 1064 (m), 1017 (w), 976 (w), 933 (w), 845 (w), 815 (w), 751 (w), 706 (w), 696 (w), 663 (m), 601 (s), 539 (m), 498 (w), 458 (w), 420 (w) cm^{-1}.

MS (ESI): *m* / *z* for $C_{23}H_{29}NO_6S$ calc.: 470.16 $[M+Na]^+$, found: 470.16.

HRMS (ESI): *m* / *z* for $C_{23}H_{29}NO_6S$ calc.: 470.1608 $[M+Na]^+$, found: 470.1609.

(2*E*,4*E*,8*R*,9*R*)-9-Hydroxy-2,8-dimethyl-10-(4-tosyloxazol-5-yl)deca-2,4-dienoic acid (*syn*-Ts-8)

According to GP3, methyl-(2*E*,4*E*,8*R*,9*R*)-9-hydroxy-2,8-dimethyl-10-(4-tosyloxazol-5-yl)deca-2,4-dienoate *syn*-Ts-**16** (140 mg, 0.31 mmol) was treated with potassium hydroxide

(246 mg, 4.38 mmol) in demin. water (5 mL) and THF (5 mL). Purification via preparative HPLC on a reversed phase column Orbit 100 C18 5 µm (250 x 20 mm) with a flow rate of 10 mLmin^{-1} and a solvent gradient of MeOH / H_2O of 75 : 25 to 95 : 5 yielded the desired product (2*E*,4*E*,8*R*,9*R*)-9-Hydroxy-2,8-dimethyl-10-(4-tosyloxazol-5-yl)deca-2,4-dienoic acid *syn*-Ts-**8** (134 mg, 0.31 mmol, 99 %, dr (*syn*/*anti)*= 100 : 0 (according to ^{1}H NMR)) as colourless foam.

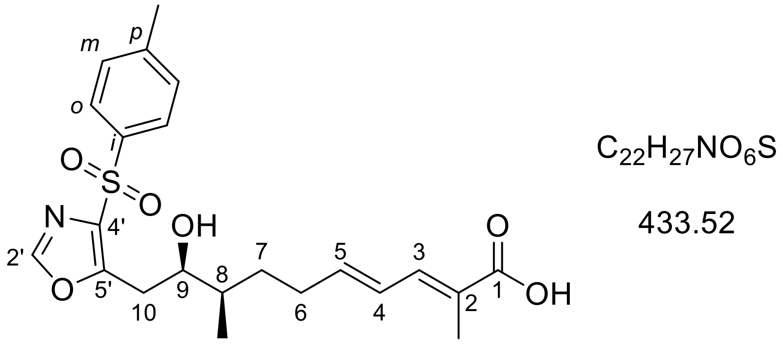

syn-Ts **-8**

^{1}H NMR (700 MHz, $CDCl_3$): δ = 1.03 (d, *J* = 6.6 Hz, 2H, 8-CH_3), 1.37 – 1.45 (m, 1H, 7-H_A), 1.50 – 1.74 (m, 2H, 7-H_B, 8-H), 1.92 (s, 3H, 2-CH_3), 2.19 – 2.27 (m, 1H, 6-H_A), 2.28 – 2.37 (m, 1H, 6-H_B), 2.42 (s, 3H, *p*-CH_3), 3.11 – 3.23 (m, 1H, 10-H_A), 3.27 – 3.37 (m, 1H, 10-H_B), 3.84 – 3.99 (m, 1H, 9-H), 6.04 – 6.18 (m, 1H, 5-H), 6.30 – 6.46 (m, 1H, 4-H), 7.26 (m, 1H, 3-H), 7.34 (d, *J* = 7.9 Hz, 2H, *m*-H), 7.76 (d, *J* = 3.4 Hz, 1H, 2'H), 7.92 (d, *J* = 8.0 Hz, 2H, *o*-H) ppm.

^{13}C NMR (176 MHz, $CDCl_3$): δ = 12.3 (2-CH_3), 13.6 (8-CH_3), 21.7 (*p*-CH_3), 30.9 (C-10), 31.0 (C-6), 32.0 (C-7), 38.1 (C-8), 73.5 (C-9), 124.5 (C-2), 126.4 (C-4), 128.1 (C-*o*), 130.0 (C-*m*), 136.6 (C-*i*), 136.9 (C-5'), 140.7 (C-3), 143.7 (C-5), 145.1 (C-*p*), 150.0 (C-2'), 154.9 (C-4'), 173.3 (C-1) ppm.

FT-IR (ATR): $\tilde{\nu}$ = 2966 (w), 2925 (m), 2250 (w), 1676 (s), 1637 (m), 1595 (m), 1517 (w), 1495 (w), 1421 (m), 1383 (w), 1322 (s), 1304 (m), 1291 (m), 1245 (m), 1145 (vs), 1108 (m), 1085 (m), 1059 (m), 1017 (m), 977 (m), 911 (m), 863 (w), 814 (m), 731 (s), 705 (s), 697 (s), 661 (vs), 600 (vs), 539 (s) cm^{-1}.

MS (ESI): *m / z* for $C_{23}H_{29}NO_6S$ calc.: 432.15 [M-H]-, found: 432.15.

HRMS (ESI): *m / z* for $C_{23}H_{29}NO_6S$ calc.: 432.1475 [M-H]-, found: 432.1492.

(2*E*,4*E*,8*R*,9*S*)- 9-Hydroxy-2,8-dimethyl-10-(4-tosyloxazol-5-yl)deca-2,4-dienoic acid (*anti*-Ts-8)

According to GP3, methyl-(2*E*,4*E*,8*R*,9*S*)-9-hydroxy-2,8-dimethyl-10-(4-tosyloxazol-5-yl)deca-2,4-dienoate *anti*-Ts-**16** (115 mg, 0.26 mmol) was treated with potassium hydroxide (201 mg, 3.60 mmol) in demin. water (5 mL) and THF (5 mL). Purification via preparative HPLC on a reversed phase column Orbit 100 C18 5 µm (250 x 20 mm) with a flow rate of 10 mLmin^{-1} and a solvent gradient of MeOH / H_2O of 75 : 25 to 95 : 5 yielded the desired product (2*E*,4*E*,8*R*,9*S*)-9-Hydroxy-2,8-dimethyl-10-(4-tosyloxazol-5-yl)deca-2,4-dienoic

acid *anti*-Ts-**8** (108 mg, 0.25 mmol, 97 %, dr (*syn*/*anti)*= 0 : 100 (according to ^{1}H NMR)) as colourless foam.

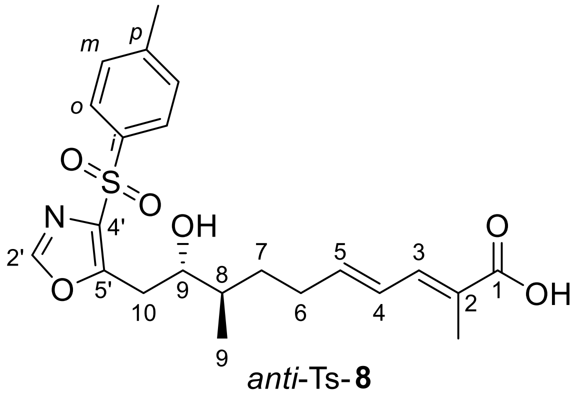

$C_{22}H_{27}NO_6S$

433.52

anti-Ts-**8**

^{1}H NMR (700 MHz, $CDCl_3$): δ = 1.04 (d, *J* = 6.8 Hz, 3H, 8-CH_3), 1.34 – 1.41 (m, 1H, 7-H_A), 1.65 – 1.72 (m, 1H, 7-H_B), 1.72 – 1.80 (m, 1-H, 8-H), 1.92 (s, 3H, 2-CH_3), 2.18 – 2.28 (m, 1H, 6-H_A), 2.30 – 2.38 (m, 1H, 6-H_B), 2.42 (s, 3H, *p*-CH_3), 3.24 – 3.27 (m, 10-H), 3.81 – 3.87 (m, 1H, 9-H), 6.14 (dt, *J* = 14.6 Hz, 7.0 Hz, 1H, 5-H), 6.37 – 6.44 (m, 1H), 4-H), 7.17 (d, *J* = 7.8 Hz, 1H, 3-H), 7.34 (d, *J* = 8.2 Hz, 2H, *m*-H), 7.76 (s, 1H, 2'-H), 7.92 (d, *J* = 8.1 Hz, 2H, *o*-H) ppm.

^{13}C NMR (176 MHz, $CDCl_3$): δ = 12.6 (2-CH_3), 15.3 (8-CH_3), 22.0 (*p*-CH_3), 30.6 (C.10), 31.1 (C-6), 31.4 (C-7), 39.0 (C-8), 74.7 (C-9), 124.7 (C-2), 126.7 (C-4), 128.5 (C-*o*), 130.3 (C-*m*), 137.2 (C-*i*), 141.0(C-3), 144.1 (C-5), 145.4 (C-*p*), 150.2 (C-2'), 155.2 (C-4'), 173.4 (C-1) ppm.

FT-IR (ATR): $\tilde{\nu}$ = 2925 (w), 2256 (w), 1677 (s), 1636 (m), 1595 (m), 1517 (w), 1495 (m), 1421 (w), 1321 (m), 1304 (m), 1291 (m), 1245 (m), 1145 (vs), 1109 (m), 1085 (m), 1060 (m), 1017 (m), 977 (m), 909 (s), 856 (w), 813 (m), 729 (s), 705 (s), 696 (s), 660 (vs), 599 (vs), 539 (vs) cm^{-1}.

MS (ESI): *m / z* for $C_{23}H_{29}NO_6S$ calc.: 456.2 $[M+Na]^+$, found: 456.2.

HRMS (ESI): *m / z* for $C_{22}H_{27}NO_6S$ calc.: 456.1451 $[M+Na]^+$, found: 456.1457.

(2*E*,4*E*,8*R*,9*R*)-9-Hydroxy-2,8-dimethyl-10-(oxazol-5-yl)deca-2,4-dienoic acid (*syn*-8)

According to GP1, (2*E*,4*E*,8*R*,9*R*)-9-Hydroxy-2,8-dimethyl-10-(4-tosyloxazol-5-yl)deca-2,4-dienoic acid *syn*-Ts-**8** (590 mg, 1.37 mmol), Na_2HPO_4 (658 mg, 5.48 mmol) and sodium amalgam (2 x 1.58 g, 2 x 6.85 mmol, 10 %), dissolved in THF (5 mL) and EtOH (5 mL), were treated in an ultrasound bath. Work up and purification were performed according to the procedure for carboxylic acids. The desired product (2*E*,4*E*,8*R*,9*R*)-9-hydroxy-2,8-dimethyl-10-(oxazol-5-yl)deca-2,4-dienoic acid *syn*-**8** **(**106 mg, 0.38 mmol, 29 %, dr (*syn*/*anti)*= 100 : 0 (according to ^{1}H NMR)) was formed as colourless oil.

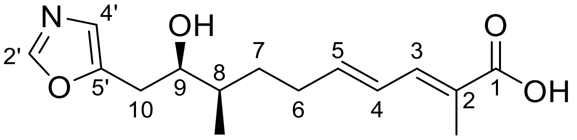

$C_{15}H_{21}NO_4$

279.34

syn-**8**

R_f = 0.55 (CH_2Cl_2 / MeOH = 10 : 1, molybdatophosphoric acid stain).

$[\alpha]_D^{20}$ = + 17.0° (c = 1.0, $CHCl_3$).

^{1}H NMR (500 MHz, $CDCl_3$): δ = 0.98 (d, J = 6.8 Hz, 3H, 8-CH_3), 1.35-1.43 (m, 1H, 7-H_a), 1.57-1.69 (m, 2H, 7-H_b, 8-H), 1.93 (s, 3H, 2-CH_3), 2.17-2.26 (m, 1H, 6-H_a), 2.27-2.35 (m, 1H, 6-H_b), 2.81-2.85 (m, 2H, 10-H), 3.85-3.89 (m, 1H, 9-H), 6.11 (ddd, J = 15.0 Hz, 14.5, 14.5 Hz, 1H, 5-H), 6.38 (dd, J = 15.0 Hz, 12.0 Hz, 1H, 4-H), 6.90 (s, 1H, 4'-H), 7.26 (d, J =12.0 Hz, 1H, 3-H), 7.82 (s, 1H, 2'-H) ppm.

^{13}C NMR (100 MHz, $CDCl_3$): δ = 12.3 (2-CH_3), 13.4 (8-Me), 30.9 (C-10), 31.0 (C-6), 32.2 (C-7), 37.4 (C-8), 72.9 (C-9), 123.6 (C-4'), 124.3 (C-2), 126.3 (C-4), 140.6 (C-3), 143.7 (C-5), 150.3 (C-5'), 150.6 (C-2'), 173.2 (C-1) ppm.

FT-IR (ATR): $\tilde{\nu}$ = 3133 (m), 2925 (s), 2618 (m), 2183 (w), 1681 (vs), 1639 (s), 1607 (m), 1513 (s), 1424 (m), 1384 (m), 1246 (s), 1165 (m), 1119 (m), 1090 (m), 1041 (m), 975 (s), 934 (m), 824 (m), 754 (w), 646 (m), 569 (w), 502 (w) cm^{-1}.

MS (ESI): m / z for $C_{15}H_{21}NO_4$ calc.: 278.1 [M-H]-, found: 278.1.

HRMS (ESI): m / z for $C_{15}H_{21}NO_4$ calc.: 278.1387 [M-H]-, found: 278.1416.

(2*E*,4*E*,8*R*,9*S*)- 9-Hydroxy-2,8-dimethyl-10-(oxazol-5-yl)deca-2,4-dienoic acid (*anti*-8)

According to GP1, (2*E*,4*E*,8*R*,9*S*)-9-Hydroxy-2,8-dimethyl-10-(4-tosyloxazol-5-yl)deca-2,4-dienoic acid *anti*-Ts-**8** (250 mg, 0.58 mmol), Na_2HPO_4 (277 mg, 2.31 mmol) and sodium amalgam (2 x 664 mg, 2 x 2.89 mmol, 10 %), dissolved in THF (5 mL) and EtOH (5 mL), were treated in an ultrasound bath. Work up and purification were performed according to the procedure for carboxylic acids. The desired product (2*E*,4*E*,8*R*,9*S*)-9-hydroxy-2,8-dimethyl-10-(oxazol-5-yl)deca-2,4-dienoic acid *anti*-**8** **(**110 mg, 0.39 mmol, 68 %, dr (*syn*/*anti)*= 0 : 100 (according to ^{1}H NMR)) was formed as colourless oil.

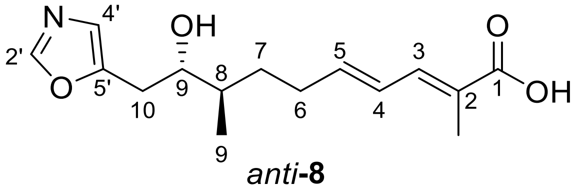

anti-**8**

$C_{15}H_{21}NO_4$

279.34

R_f = 0.55 (CH_2Cl_2 / MeOH = 10 : 1, molybdatophosphoric acid stain).

$[\alpha]_D^{20}$ = - 10.8° (c = 1.0, $CHCl_3$).

^{1}H NMR (500 MHz, $CDCl_3$): δ = 1.00 (d, J = 6.7 Hz, 3H, 8-CH_3), 1.30-1.39 (m, 1H, 7-H_a), 1.60-1.66 (m, 1H, 8-H), 1.68-1.76 (m, 1H, 7-H_b), 1.93 (s, 3H, 2-CH_3), 2.14-2.23 (m, 1H, 6-H_a), 2.30-2.40 (m, 1H, 6-H_b), 2.79 (dd, J = 15.0 Hz, 9.0 Hz, 1H, 10-H_a), 2.89 (dd, J = 15.0 Hz, 3.1 Hz, 1H, 10-H_b), 3.74-3.79 (m, 1H, 9-H), 6.12 (ddd, J = 15.0 Hz, 14.5 Hz, 14.5 Hz, 1H, 5-H), 6.38 (dd, J = 15.0, 11.5 Hz, 1H, 4-H), 6.91 (s, 1H, 4'-H), 7.26 (d, J = 11.5 Hz, 1H, 3-H), 7.84 (s, 1H, 2'-H) ppm.

^{13}C NMR (125 MHz, $CDCl_3$): δ = 12.3 (2-CH_3), 15.2 (8-CH_3), 30.3 (C-10), 30.8 (C-6), 31.1 (C-7), 38.0 (C-8), 73.8 (C-9), 123.7 (C-4'), 124.5 (C-2), 126.3 (C-4), 140.6 (C-3), 143.7 (C-5), 150.3 (C-5'), 150.6 (C-2'), 173.2 (C-1) ppm.

FT-IR (ATR): $\tilde{\nu}$ = 3137 (m), 2926 (s), 2605 (w), 2209 (w), 2175 (w), 2149 (w), 2054 (w), 2033 (w), 1997 (w), 1974 (w), 1680 (vs), 1639 (s), 1607 (m), 1513 (m), 1423 (m), 1245 (s), 1120 (m), 1090 (m), 1047 (m), 975 (s), 825 (m), 753 (w), 646 (m), 567 (w), 491 (w), 437 (w), 420 (w) cm^{-1}.

MS (ESI): *m / z* for $C_{15}H_{21}NO_4$ calc.: 278.1 [M-H]-, found: 278.1.

HRMS (ESI): *m / z* for $C_{15}H_{21}NO_4$ calc.: 278.1387 [M-H]-, found: 278.1400.

(3*E*,5*E*,9*R*,10*R*,13*E*,15*E*,19*R*,20*R*)-3,9,13,19-tetramethyl-10,20-bis((4-tosyloxazol-5-yl)methyl)-1,11-dioxacycloicosa-3,5,13,15-tetraene-2,12-dione (*syn*,*syn*-Ts-4a)

According to GP4, (2*E*,4*E*,8*R*,9*R*)-9-Hydroxy-2,8-dimethyl-10-(4-tosyloxazol-5-yl)deca-2,4-dienoic acid *syn*-Ts-**8** (75.0 mg, 0.17 mmol) was treated with NEt_3 (0.21 mL, 28.9 µg, 0.12 mmol), 2,4,6-trichlorobenzoyl chloride (41.0 µL, 63.3mg, 0.26 mmol) and 4-dimethylaminopyridine (126 mg, 1.04 mmol) in dry THF (20 mL) and dry benzene (80 mL). Column chromatography on silica (petroleum ether / EtOAc 3 : 1) and further purification with preparative HPLC on a reversed-phase column Orbit 100 C18 5 µm (250 x 20 mm) with a flow rate of 10 $mLmin^{-1}$ and a solvent gradient of MeOH / water of 75 : 25 to 95 : 5 yielded the desired product *syn*,*syn*-Ts-**4a** (14.0 mg, 16.9 mmol, 20 %, dr (*syn*,*syn* / *syn*,*anti* / *anti*,*anti*) = 100 : 0 : 0 (according to ^{1}H NMR)) as colourless oil.

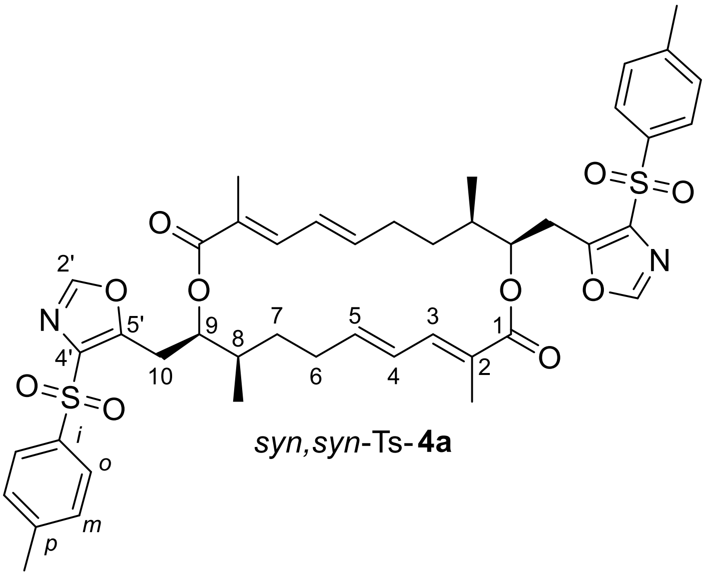

syn,syn-Ts-**4a**

$C_{44}H_{50}N_2O_{10}S_2$

831.01

R_f = 0.54 (petroleum ether / EtOAc 1 : 1).

$[\alpha]_D^{20} = 27.3°$ (c = 1.0, CH_2Cl_2).

^{1}H NMR (400 MHz, $CDCl_3$): δ = 1.06 (d, *J* = 7.0 Hz, 6H, 8-CH_3), 1.45 – 1.56 (m, 2H, 7-H_A), 1.69 (s, 6H, 2-CH_3), 1.78 – 1.86 (m, 2H, 7-H_B), 1.87 – 2.00 (m, 2H, 6-H_A), 2.07 – 2.21 (m, 1H, 6-H_B), 2.41 (s, 3H, *p*-CH_3), 2.48 – 2.59 (m, 1H, 8-H), 3.26 – 3.33 (m, 2H, 10-H_A), 3.40 – 3.50 (m, 2H, 10-H_B), 5.07 – 5.18 (m, 2H, 9-H), 5.84 – 6.14 (m, 2H, 5-H), 6.30 (dd, *J* = 14.9 Hz,

11.2 Hz, 2H, 4-H), 7.00 (d, J = 11.1 Hz, 2H, 3-H), 7.28 (d, J = 8.1 Hz, 4H, *m*-H), 7.72 (s, 2H, 2'-H), 7.80 – 7.96 (m, 4H, *o*-H) ppm.

^{13}C NMR (176 MHz, $CDCl_3$): δ = 13.1 (2-CH_3), 16.7 (8-CH_3), 22.2 (*p*-CH_3), 30.0 (C-10), 30.2 (C-6), 30.5 (C-7), 35.1 (C-8), 76.0 (C-9), 126.0 (C-4), 128.1 (C-*o*), 128.6 (C-2), 130.3 (C-*m*), 137.0 (C-*i*), 137.5 (C-5'), 138.9 (C-3), 143.4 (C-5), 145.3 (C-*p*), 150.4 (C-2'), 154.3 (C-4'), 167.9 (C-1) ppm.

FT-IR (ATR): $\tilde{\nu}$ = 3131(w), 2959 (w), 2924 (m), 2855 (w), 1698 (s), 1640 (w), 1595 (m), 1515 (w), 1495 (w), 1482 (w), 1458 (w), 1364 (w), 1324 (m), 1304 (w), 1288 (w), 1244 (s), 1185 (w), 1146 (vs), 1100 (s), 1085 (s), 1052 (s), 1016 (s), 973 (m), 908 (s), 799 (s), 730 (vs), 700 (s), 660 (vs), 599 (vs), 536 (vs) cm^{-1}

MS (ESI): *m / z* for $C_{44}H_{50}N_2O_{10}S_2$ calc.: 848.33 $[M+NH_4]^+$, found: 848.32

HRMS (ESI): *m / z* for $C_{44}H_{50}N_2O_{10}S_2$ calc.: 848.3250 $[M+NH_4]^+$, found: 848.3245.

(3*E*,5*E*,9*R*,10*R*,13*E*,15*E*,19*R*,20*R*)-3,9,13,19-3,9,13,19-tetramethyl-10,20-bis((4-tosyloxazol-5-yl)methyl)-1,11-dioxacycloicosa-3,5,13,15-tetraene-2,12-dione (*anti,anti*-Ts-4a)

According to GP4, (2*E*,4*E*,8*R*,9*s*)-9-Hydroxy-2,8-dimethyl-10-(4-tosyloxazol-5-yl)deca-2,4-dienoic acid *anti*-Ts-**4a** (50.0 mg, 0.12 mmol) was treated with NEt_3 (20.0 µL, 14.0 mg, 0.14 mmol), 2,4,6-trichlorobenzoyl chloride (27.1 µL, 42.2 mg, 0.17 mmol) and 4-dimethylaminopyridine (84.5 mg, 0.69 mmol) in dry THF (20 mL) and dry benzene (80 mL). Column chromatography on silica (petroleum ether / EtOAc 3 : 1) and further purification with preparative HPLC on a reversed-phase column Orbit 100 C18 5 µm (250 x 20 mm) with a flow rate of 10 mLmin^{-1} and a solvent gradient of MeOH / water of 75 : 25 to 95 : 5 yielded the desired product *anti,anti*-Ts-**4a** (11.0 mg, 13.2 µmol, 23 %, dr (*syn,syn* / *syn,anti* / *anti,anti*) = 0 : 0 : 100 (according to ^{1}H NMR)) as colourless oil.

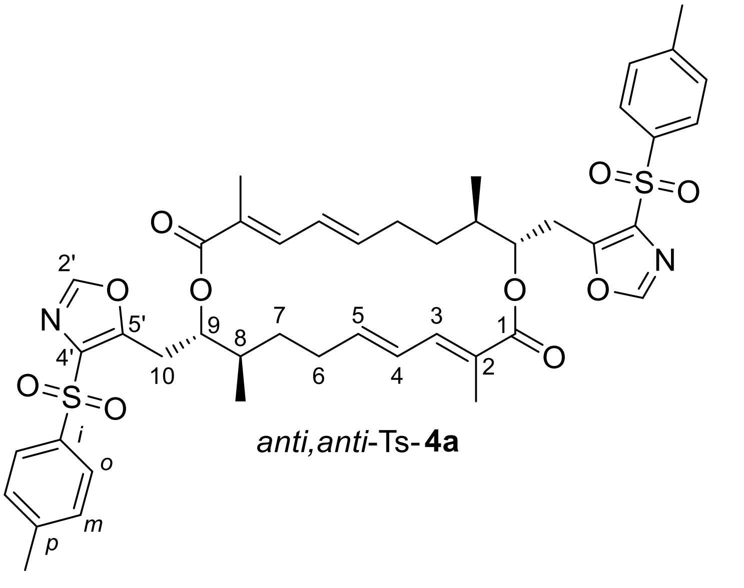

anti,anti-Ts-**4a**

$C_{44}H_{50}N_2O_{10}S_2$

831.01

R_f = 0.76 (petroleum ether / EtOAc 1 : 1).

$[\alpha]_D^{20} = -33.5°$ (c = 1.0, CH_2Cl_2).

^{1}H NMR (700 MHz, $CDCl_3$): δ = 1.10 (d, J = 6.7 Hz, 6H, 8-CH_3), 1.28 – 1.35 (m, 2H, 7-H_A), 1.39 – 1.46 (m, 2H, 7-H_B), 1.77 (s, 6H, 2-CH_3), 1.82 – 1.91 (m, 2H, 8-H), 2.18 – 2.26 (m, 2H, 6-H_A), 2.26 – 2.33 (m, 2H, 6-H_B), 2.41 (s, 6H, *p*-CH_3), 3.40 (dd, J = 15.1 Hz, 8.4 Hz, 2H, 10-H_A), 3.61 (dd, J = 15.1 Hz, 3.3 Hz, 2H, 10-H_B), 4.94 – 5.00 (m, 2H, 9-H), 6.05 – 6.14 (m, 2H, 5-H), 6.30 – 6.40 (m, 2H, 4-H), 7.11 – 7.20 (m, 2H, 3-H), 7.29 (d, J = 8.0 Hz, 4H, *m*-H), 7.74 (s, 2H, 2‘-H), 7.89 (d, J = 8.1 Hz, 4H, *o*-H) ppm.

^{13}C NMR (176 MHz, $CDCl_3$): δ = 12.6 (2-CH_3), 16.3 (8-CH_3), 21.7 (*p*-CH_3), 29.0 (C-10), 29.7 (C-6), 32.9 (C-7), 36.3 (C-8), 75.4 (C-9), 126.8 (C-4), 128.3 (C-*o*), 129.1 (C-2), 129.8 (C-*m*), 136.9 (C-*i*), 137.0 (C-5’), 138.9 (C-3), 142.8 (C-5), 144.8 (C-*p*), 150.1 (C-2’), 153.2 (C-4’), 167.4 (C-1) ppm.

FT-IR (ATR): $\tilde{\nu}$ = 3129 (w), 2925 (m), 1771 (m), 1697 (s), 1639 (m), 1594 (m), 1548 (w), 1515 (m), 1494 (w), 1458 (m), 1317 (m), 1326 (s), 1305 (m), 1283 (m), 11241 (s), 1147 (vs), 1085 (s), 1037 (m), 1018 (m), 975 (m), 913 (m), 859 (w), 814 (m), 732 (s), 706 (m), 696 (m), 663 (s), 599 (vs), 537 (s) cm^{-1}.

MS (ESI): *m / z* for $C_{44}H_{50}N_2O_{10}S_2$ calc.: 831.30 [M+H], found: 831.30.

HRMS (ESI): *m / z* for $C_{44}H_{50}N_2O_{10}S_2$ calc.: 831.2980 [M+H], found: 831.2988.

13.4 Synthesis of samroiyotmycin derivatives

1-Phenylhex-5-en-1-ol (S10a)

Under an inert atmosphere (N_2), magnesium turnings (310 mg, 12.7 mmol) were dissolved in dry THF (20 mL) and 5-bromopent-1-en **S12a** (1.50 mL, 1.89 mg, 12.7 mmol) was added dropwise at rt. In a separate flask, benzaldehyde **S11** (1.28 mL, 1.34 g, 12.7 mmol) was dissolved in dry THF (20 mL) at 0 °C and the before prepared Grignard reagent was added dropwise. After stirring for 18 h at rt, the reaction mixture was poured onto sat. NH_4Cl-solution (20 mL), extracted with EtOAc (3 x 10 mL) and the combined organic phases were washed with sat. NaCl-solution (3 x 10 mL) and dried over $MgSO_4$. Column chromatography on silica (petroleum ether / EtOAc 20 : 1) yielded the desired product 1-phenylhex-5-en-1-ol **S10a** (1.62 g, 9.16 mmol, 72 %, dr (*syn*/*anti*) = 51 : 49) as colourless oil.

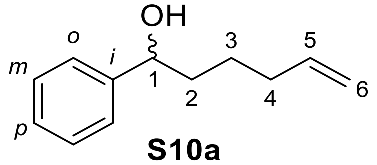

S10a

$C_{12}H_{16}O$

176.26

R_f = 0.33 (petroleumm ether / EtOAc = 10 : 1, molybdatophosphoric acid stain).

^{1}H NMR (500 MHz, $CDCl_3$): δ = 1.32-1.59 (m, 2H, 3-H), 1.67-1.86 (m, 2H, 2-H), 2.08 (td, J = 7.3 Hz, 6.9 Hz, 2H, 4-H), 4.67 (t, J = 7.3 Hz, 1H, 1-H), 4.92-5.02 (m, 2H, 6-H), 5.78 (ddt, J =17.0 Hz, 10.2 Hz, 6.9 Hz, 1H, 5-H), 7.26-7.30 (m, 1H, *p*-H), 7.32-7.35 (m, 4H, *o*-H, *m*-H) ppm.

^{13}C NMR (125 MHz, $CDCl_3$): δ = 25.1 (C-3), 33.6 (C-4), 38.5 (C-2), 74.5 (C-1), 114.7 (C-6), 125.9 (C-*o* / C-*m*), 127.5 (C-*p*), 128.5 (C-*m* / C-*o*), 138.5 (C-5), 144.8 (C-*i*) ppm.

The spectroscopic data is in agreement with the literature[31].

1-Phenylhept-6-en-1-ol (S10b)

Under an inert atmosphere (N_2), magnesium turnings (109 mg, 4.49 mmol) were dissolved in dry THF (20 mL) and 6-bromohex-1-en **S12b** (0.60 mL, 0.73 mg, 4.49 mmol) was added dropwise at rt. In a separate flask, benzaldehyde **S11** (0.45 mL, 477 mg, 4.49 mmol) was dissolved in dry THF (20 mL) at 0 °C and the before prepared Grignard reagent was added dropwise. After stirring for 18 h at rt, the reaction mixture poured onto sat. NH_4Cl-solution (20 mL), extracted with EtOAc (3 x 10 mL) and the combined organic phases were washed with sat. NaCl-solution (3 x 10 mL) and dried over $MgSO_4$. Column chromatography on silica (petroleum ether / EtOAc 20 : 1) yielded the desired product 1-phenylhept-6-en-1-ol **S10b** (500 mg, 2.63 mmol, 59 %, dr (*syn*/*anti*) = 50 : 50) as colourless oil.

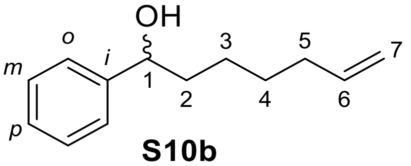

S10b

$C_{13}H_{18}O$

190.29

R_f = 0.30 (petroleum ether / EtOAc = 10 : 1, molybdatophosphoric acid stain).

^{1}H NMR (400 MHz, $CDCl_3$): δ = 1.24-1.46 (m, 4H, 3-H, 4-H), 1.67-1.84 (m, 2H, 2-H), 2.04 (d, J = 6.7 Hz, 2H, 5-H), 4.66 (t, J = 6.3 Hz, 1H, 1-H), 4.90-5.01 (m, 2H, 7-H), 5.73-5.84 (m, 1H, 6-H), 7.24-7.30 (m, 1H, *p*-H), 7.32-7.36 (m, 4H, *o*-H, *m*-H) ppm.

^{13}C NMR (100 MHz, $CDCl_3$): δ = 25.3 (C-3), 28.8 (C-4), 33.7 (C-5), 38.9 (C-2), 74.5 (C-1), 114.7 (C-7), 125.9 (C-*o* / C-*m*), 127.5 (C-*p*), 128.5 (C-*m* / C-*o*), 138.8 (C-6), 144.8 (C-*i*) ppm.

The spectroscopic data is in agreement with the literature[31].

(3*R*)-3-Methyl-1-phenylhept-6-en-2-ol (19)

Under inert atmosphere (N_2), magnesium turnings (243 mg, 10.0 mmol) were dissolved in diethyl ether (20 mL) and benzylbromide (1.19 mL, 1.71 g, 10.0 mmol) was added dropwise at rt. In a separate flask, (*R*)-2-methylhex-5-enal **S9 (**561 mg, 5.00 mmol) was dissolved in dry diethyl ether (10 mL) and after stirring for 1 h the before prepared Grignard reagent was added dropwise at 0 °C, warmed to rt and stirred for 1.5 h. The reaction mixture was added dropwise to sat. NH_4Cl-solution (20 mL) and was extracted with EtOAc (3 x 50 mL). The combined organic phases were dried over $MgSO_4$ and column chromatography on silica (petroleum ether / EtOAc 10 : 1) yielded the desired product (3*R*)-3-methyl-1-phenylhept-6-en-2-ol **19** (370 mg, 1.81 mmol, 36 %, dr (*syn*/*anti*) = 67 : 33) as colourless oil.

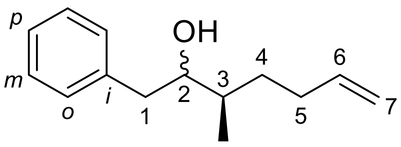

19

$C_{14}H_{20}O$

204.31

R_f = 0.45 (petroleum ether / EtOAc = 10 : 1, molybdatophosphoric acid stain).

^{1}H NMR (500 MHz, $CDCl_3$): δ = 0.99 (d, J = 6.8 Hz, 3H, 3-CH_3), 1.28-1.39 (m, 1H, 3-H), 1.57-1.73 (m, 2H, 4-H), 2.00-2.23 (m, 2H, 5-H), 2.54-2.70 (m, 1H, 1-Ha), 2.77-2.88 (m, 1H, 1-H_b), 3.64-3.78 (m, 1H, 2-H), 4.93-5.07 (m, 2H, 7-H), 5.76-5.88 (m, 1H, 6-H), 7.20-7.25 (m, 3H, *o*-H, *p*-H), 7.29-7.35 (m, 2H, *m*-H) ppm.

^{13}C NMR (125 MHz, $CDCl_3$): δ = 13.8 (3-CH_3), 31.5 (C-5), 32.5 (C-4), 37.3 (C-3), 41.0 (C-1), 75.9 (C-2), 114.5 (C-7), 126.4 (C-*p*), 128.6 (C-*m*), 129.3 (C-*o*), 138.9 (C-5), 139.2 (C-*i*) ppm.

FT-IR (ATR): $\tilde{\nu}$ = 3566 (w), 3436 (w), 3063 (w), 3027 (w), 2962 (m), 2922 (s), 2876 (m), 2190 (w), 2164 (w), 2153 (w), 2124 (w), 2041 (w), 1987 (w), 1951 (w), 1825 (w), 1708 (w), 1639 (m), 1602 (w), 1495 (m), 1454 (m), 1415 (w), 1378 (w), 1262 (w), 1179 (w), 1074 (m), 1030 (m), 993 (s), 909 (s), 865 (w), 742 (s), 699 (vs), 637 (w), 538 (w), 461 (w), 412 (w) cm^{-1}.

MS (EI): m/z (%) = 43.0 (10), 55.1 (9), 65.0 (5), 77.0 (4), 92.1 (100), 103.1 (7), 121.1 (6), 204.2 $[M]^+$ (5).

HRMS (EI): *m / z* for $C_{14}H_{20}O$ calc.: 204.1514 $[M]^+$, found: 204.1512.

(2*R*)-2-Methyl-1-(naphthalen-2-yl)hex-5-en-1-ol (24a)

According to GP5, 2-bromo naphataline (369 mg, 1.78 mmol) was treated with *n*-butyllithium (126 mg, 1.96 mL, 2.5 M in hexane) and (*R*)-2-methylhex-5-enal **S9** (200 mg, 1.78 mmol) in dry THF (20 mL). Column chromatography on silica (petroleum ether / EtOAc 10 : 1) yielded (2*R*)-2-methyl-1-(naphthalen-2-yl)hex-5-en-1-ol **24a** (210 mg, 0.87 mmol, 49 %, dr (*syn*/*anti*) = 58 : 42) as colourless oil.

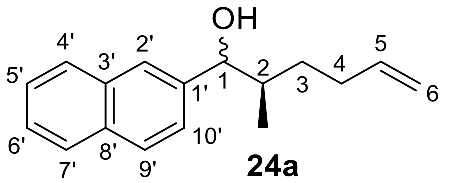

24a

$C_{17}H_{20}O$

240.35

R_f = 0.40 (petroleum ether / EtOAc 10 : 1).

^{1}H NMR (700 MHz, $CDCl_3$) δ = 0.81 (d, J = 6.8, 3H, Me*), 0.96 (d, J = 6.8, 3H, Me), 1.19 – 1.33 (m, 2H, 3-H_A, 3-H_A*), 1.47 – 1.55 (m, 1H, 3-H_B), 1.79 – 1.85 (m, 1H, 3-H_B*), 1.90 – 2.00 (m, 2H, 2-H, 2-H*), 2.00 – 2.07 (m, 1H, 4-H), 2.12 – 2.25 (m, 1H, 4-H*), 4.62 (d, J = 7.0 Hz, 1H, 1-H*), 4.73 (d, J = 5.7 Hz, 1H, 1-H), 4.90 – 5.05 (m, 2H, 6-H, 6-H*), 5.69 – 5.86 (m, 2H, 5-H, 5-H*), 7.41 – 7.52 (m, 6H, 4'-H, 7'-H, 9'-H, 4'-H*, 7'-H*, 9'-H*), 7.76 (d, J = 9.9 Hz, 2H, 2'-H, 2'-H*), 7.83 (t, J = 7.5 Hz, 6H, 5'-H, 6'-H, 10'-H, 5'-H*, 6'-H*, 10'-H)* ppm.

^{13}C NMR (176 MHz, $CDCl_3$) δ = 14.2 (Me), 15.7 (Me*), 31.3 (C-3*), 31.4 (C-3), 31.4 (C-4*) 32.4 (C-4), 39.4 (C-2), 39.5 (C-2*), 78.1 (C-1), 79.2 (C-1*), 114.5 (C-6*), 114.6 (C-6), 124.5 (C-4'), 124.7 (C-4'*), 125.1 (C-2'), 125.6 (C-2'*), 125.8 (C-7'), 125.8 (C-7'*), 126.1 (C-9'), 126.1 (C-9'*), 127.7 (C-6'), 128.0 (C-6'*), 128.0 (C-5'), 128.1 (C-5'*), 132.9 (C-10'), 133.0 (C-10'*), 133.0 (C-3'), 133.2 (C-3'*), 133.2 (C-8'), 133.2 (C-8'*), 138.8 (C-5), 139.0 (C-5*), 141.0 (C-1'), 141.2 (C-1'*) ppm.

FT-IR (ATR): $\tilde{\nu}$ = 3404 (br), 3057 (m), 2971 (s), 2929 (s), 2875 (s), 1639 (m), 1601 (w), 1507 (m), 1460 (m), 1437 (m), 1414 (w), 1375 (m), 1297 (w), 1270 (m), 1165 (w), 1124 (m), 1018

(s), 996 (s), 948 (w), 908 (vs), 858 (s), 819 (vs), 774 (w), 747 (s), 620 (w), 550 (w), 478 (s), 411 (w) cm^{-1}.

MS (ESI): *m / z* for $C_{17}H_{20}O$ calc.: 263.14 $[M+Na]^+$, found: 263.14.

HRMS (ESI): *m / z* for $C_{17}H_{20}O$ calc.: 263.1406 $[M+Na]^+$, found: 263.1399.

(2*R*)-2-Methyl-1-(pyridin-2-yl)hex-5-en-1-ol (24b)

According to GP5, 2-bromo pyridine (259 mg, 1.78 mmol) was treated with *n*-butyllithium (126 mg, 1.96 mmol, 2.5 M in hexane) and (*R*)-2-methylhex-5-enal **S9** (200 mg, 1.78 mmol) in dry THF (20 mL). Column chromatography on silica (petroleum ether / EtOAc 8 : 1) yielded (2*R*)-2-methyl-1-(pyridin-2-yl)hex-5-en-1-ol **24b** (102 mg, 0.53 mmol, 30 %, dr (*syn*/*anti*) = 61 : 39) as colourless oil.

The seperation of the diastereomeric mixture *syn*-**24b** und *anti*-**24b** was performed via column chromatography on silica (petroleum ether / EtOAc 8 : 1). The determination of the absolute configuration of the alcohols was performed through optical rotation and retention times and in comparison with the optical rotation of *syn*-Ts-**10** and *anti*-Ts-**10.**

(1*S*,2*R*)-2-Methyl-1-(pyridin-2-yl)hex-5-en-1-ol (*syn*-24b)

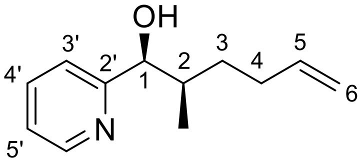

syn-**24b**

$C_{12}H_{17}NO$

191.27

R_f = 0.48 (petroleum ether / EtOAc 3 : 1).

$[\alpha]_D^{20} = 15.4°$ (c = 1.0, CH_2Cl_2).

^1H NMR (700 MHz, $CDCl_3$): δ = 0.97 (d, J = 6.9 Hz, 3H, 2-CH_3), 1.21 – 1.30 (m, 1H, 3-H_A), 1.35 – 1.43 (m, 1H, 3-H_B), 1.85 – 1.95 (m, 1H, 2-H), 2.06 – 2.14 (m, 2H, 4-H), 4.60 (d, J = 3.2 Hz, 1H, 1-H), 4.86 – 4.97 (m, 2H, 6-H), 5.65 – 5.75 (m, 1H, 5-H), 7.18 – 7.24 (m, 2H, 5'-H, 4'-H), 7.64 – 7.71 (m, 1H, 3'-H), 8.53 – 8.58 (m, 1H, 6'-H) ppm.

^{13}C NMR (176 MHz, $CDCl_3$) δ = 16.2 (CH_3), 29.7 (C-4), 31.4 (C-3), 39.5 (C-2), 76.9 (C-1), 114.4 (C-6), 121.3 (C-4'), 122.3 (C-5'), 136.6 (C-3'), 138.9 (C-5), 147.9 (C-6'), 161.0 (C-2') ppm.

FT-IR (ATR): $\tilde{\nu}$ = 3380 (br). 3078 (w), 2966 (m), 2930 (m), 2876 (m), 2856 (m), 1639 (m), 1594 (s), 1571 (m), 1473 (m), 1436 (s), 1406 (m), 1378 (m), 1309 (w), 1261 (w), 1149 (m), 1122 (w), 1085 (w), 1048 (m), 996 (vs), 909 (s), 833 (w), 754 (vs), 674 (w), 627 (m), 555 (w), 530 (w), 424 (w) cm^{-1}.

MS (ESI): *m / z* for $C_{12}H_{17}NO$ calc.: 214.12 [M+Na], found: 214.12.

HRMS (ESI): *m / z* for $C_{12}H_{17}NO$ calc.: 214.1203 [M+Na], found: 214.1203.

(1*S*,2*S*)-2-methyl-1-(pyridin-2-yl)hex-5-en-1-ol (*anti*-24b)

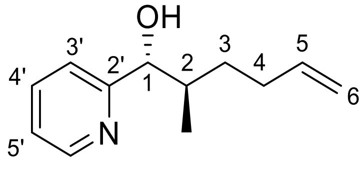

anti-**24b**

$C_{12}H_{17}NO$

191.27

R_f = 0.40 (petroleum ether / EtOAc 3 : 1).

$[\alpha]_D^{20} = -22.8°$ (c = 1.0, CH_2Cl_2).

^{1}H NMR (500 MHz, $CDCl_3$): δ = 0.89 (d, *J* = 6.9 Hz, 3H, 2-CH_3), 1.11 – 1.23 (m, 1H, 3-H_A), 1.29 – 1.38 (m, 1H, 3-H_B), 1.77 – 1.88 (m, 2H, 4-H), 1.98 – 2.08 (m, 1H, 2-H), 4.52 (d, *J* = 4.6 Hz, 1H, 1-H), 4.76 – 4.89 (m, 2H, 6-H), 5.63 (m, 1H, 5-H), 7.08 – 7.17 (m, 2H, 5'-H, 4'-H), 7.60 (m, 1H, 3'-H), 8.47 (m, 1H, 6'-H) ppm.

^{13}C NMR (176 MHz, $CDCl_3$): δ = 12.7 (CH_3), 31.6 (C-4), 32.9 (C-3), 39.3 (C-2), 74.8 (C-1), 114.5 (C-6), 120.8 (C-4'), 122.2 (C-5'), 136.7 (C-3'), 138.9 (C-5), 147.6 (C-6'), 161.2 (C-2') ppm.

FT-IR (ATR): $\tilde{\nu}$ = 3380 (br), 3074 (w), 2965 (s), 2930 (s), 2876 (m), 2856 (m), 1639 (m), 1594 (s), 1571 (m), 1473 (m), 1435 (s), 1405 (m), 1377 (w), 1310 (w), 1261 (w), 1213 (w), 1148 (w), 1121 (w), 1085 (w), 1048 (m), 977 (vs), 909 (s), 833 (w), 754 (vs), 708 (w), 674 (w), 626 (w), 555 (w), 530 (w), 444 (w), 421 (w) cm^{-1}.

MS (ESI): *m / z* for $C_{12}H_{17}NO$ calc.: 214.12 [M+Na], found: 214.12.

HRMS (ESI): *m / z* for $C_{12}H_{17}NO$ calc.: 214.1203 [M+Na], found: 214.1204.

(2*R*)-2-Methyl-1-(thiophen-2-yl)hex-5-en-1-ol (24c)

According to GP5, 2-bromo thiophene (291 mg, 1.78 mmol) was treated with *n*-butyllithium (126 mg, 1.96 mmol, 2.5 M in hexane) and (*R*)-2-methylhex-5-enal **S9** (200 mg, 1.78 mmol) in dry THF (20 mL). Column chromatography on silica (petroleum ether / EtOAc 10 : 1) yielded (2*R*)-2-methyl-1-(thiophen-2-yl)hex-5-en-1-ol **24c** (250 mg, 1.27 mmol, 71 %, dr (*syn*/*anti*) = 56 : 44) as colourless oil.

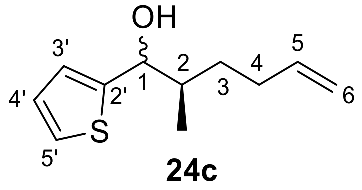

24c

$C_{11}H_{16}OS$

196.31

R_f = 0.60 (petroleum ether / EtOAc 5 : 1)

^{1}H NMR (700 MHz, $CDCl_3$) δ = 0.86 (d, *J* = 6.8 Hz, 3H, CH_3*), 1.00 (d, *J* = 6.8 Hz, 3H, CH_3), 1.18 – 1.30 (m, 2H, 3-H_A, 3-H_A*), 1.48 – 1.55 (m, 1H, 3-H_B), 1.74 – 1.80 (m, 1H, 3-H_B*), 1.85 – 1.93 (m, 2H, 2-H, 2-H*), 1.93 – 1.99 (m, 2H, OH, OH*), 2.01 – 2.06 (m, 2H, 4-H), 2.11 – 2.23 (m, 2H, 4-H*), 4.73 (d, *J* = 6.9 Hz, 1H, 1-H*), 4.79 (d, *J* = 5.9 Hz, 1H, 1-H), 4.90 – 5.07

(m, 4H, 6-H, 6-H*), 5.72 – 5.86 (m, 2H, 5-H, 5-H*), 6.93 – 6.98 (m, 4H, 3'-H, 4'-H, 3'-H*, 4'-H*), 7.23 – 7.25 (m, 2H, 3'-H, 3'-H*) ppm.

^{13}C NMR (176 MHz, CDCl$_3$) δ = 14.6 (CH$_3$), 15.5 (CH$_3$*), 31.2 (C-4*), 31.3 (C-4), 31.5 (C-3*), 32.1 (C-3), 40.1 (C-2), 40.1 (C-2*), 74.5 (C-1), 74.9 (C-1*), 114.5 (C-6*), 114.6 (C-6*), 124.1 (C-4'), 124.4 (C-5'), 124.4 (C-4'*), 124.6 (C-5'*), 126.5 (C-3'*), 126.6 (C-3'), 138.7 (C-5), 138.9 (C-5*), 147.4 (C-2'), 147.7 (C-2'*) ppm.

FT-IR (ATR): $\tilde{\nu}$ = 3385 (br), 3074 (w), 2971 (m), 2925 (m), 2876 (m), 2855 (m), 1639 (m), 1458 (m), 1438 (m), 1414 (w), 1376 (m), 1298 (w), 1230 (w), 1154 (w), 994 (s), 909 (s), 853 (m), 828 (m), 794 (w), 755 (w), 696 (vs), 641 (w), 553 (w), 535 (w) cm^{-1}.

MS (ESI): *m / z* for $C_{11}H_{16}OS$ calc.: 219.08 [M+Na], found: 219.08

HRMS (ESI): *m / z* for $C_{11}H_{16}OS$ calc.: 219.0814 [M+Na], found: 219.0812.

Methyl (2*E*,4*E*,8*R*)-9-hydroxy-2,8-dimethyl-9-(naphthalen-2-yl)nona-2,4-dienoate (25a)

According to GP2, (2*R*)-2-methyl-1-(naphtalen-2-yl)hex-5-en-1-ol **24a** (133 mg, 0.55 mmol) was treated with (2*E*,4*E)*-2-methy2-2,4-hexadienoate **15** (155 mg, 1.11 mmol) and Grubbs-II catalyst (70.5 mg, 0.08 mmol) in dichloromethane (20 mL). Column chromatography on silica (petroleum ether / EtOAc 10 : 1) yielded methyl (2*E*,4*E*,8*R*)-9-hydroxy-2,8-dimethyl-9-(naphtalen-2-yl)nona-2,4-dienoate **25a (**144 mg, 0.43 mmol, 77 %, dr (*syn*/*anti*) = 58 : 42) as colourless oil.

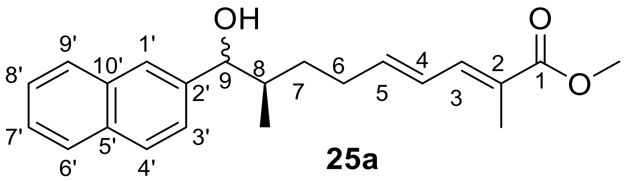

25a

$C_{22}H_{26}O_3$

338.45

^{1}H NMR (400 MHz, CDCl$_3$): δ = 0.83 (d, *J* = 6.8 Hz, 3H, 8-Me*), 0.98 (d, *J* = 6.8 Hz, 3H, 8-Me), 1.17 – 1.39 (m, 2H, 7-H$_A$, 7-H$_A$*), 1.49 – 1.61 (m, 2H, 7-H$_B$, 7-H$_B$*), 1.80 – 2.02 (m, 8H, 8H, 2-Me, 2-Me*, 8-H, 8-H*), 2.08 – 2.22 (m, 1H, 6-H$_A$, 6-H$_A$*), 2.22 – 2.40 (m, 1H, 6-H$_B$; 6-H$_B$*), 3.74 (d, *J* = 3.0 Hz, 6H, OMe, OMe*), 4.62 (d, *J* = 7.0 Hz, 1H, OH*), 4.71 (d, *J* = 5.7 Hz, 1H, OH), 6.02 (m, 2H, 4-H, 4-H*), 6.22 – 6.38 (m, 2H, 5-H, 5-H*), 7.14 (m, 2H, 3-H, 3-H*), 7.39 – 7.54 (m, 6H, 4'-H, 7'-H .9'-H, 4'-H*, 7'-H*, 9'-H*), 7.75 (t, *J* = 2.4 Hz, 2H, 2'-H, 2'-H*), 7.79 – 7.88 (m, 6H, 5'-H, 6'-H, 10'-H, 5'-H*, 6'-H*, 10'-H*) ppm.

^{13}C NMR (101 MHz, CDCl$_3$): δ = 12.6 (2-Me), 14.4 (8-Me), 15.9 (8-Me*), 30.8 (C-6), 30.9 (C-6*), 31.3 (C-7), 32.4 (C-7*), 39.6 (C-8), 39.6 (C-8*), 51.8 (OMe), 51.8 (OMe*), 78.2 (C-9), 79.1 (C-9*), 124.5 (C-4'), 124.6 (C-4'*), 124.9 (C-2'), 125.0 (C-2'*), 125.2 (C-5), 125.5 (C-5*), 125.8 (C-7'), 125.9 (C-7'*), 126.1 (C-9'), 126.2 (C-9'*), 127.7 (C-6'), 127.9 (C-6'*), 128.1 (C-5'), 128.1 (C-5'*), 128.1 (C-4), 128.1 (C-4*), 132.9 (C-10'), 132.9 (C-10'*), 133.2 (C-8'), 133.2 (C.-8'*), 138.7 (C-3'), 138.9 (C-3'*), 140.9 (C-1'), 141.0 (C-1'*), 142.8 (C-2), 142.8 (C-2*), 143.1 (C-3), 143.1 (C-3*), 169.1 (C-1) ppm.

FT-IR (ATR): $\tilde{\nu}$ = 3465 (br), 3054 (w), 2949 (m), 2929 (m), 2229 (w), 2169 (w), 2062 (w), 2028 (w), 1981 (w), 1927 (w), 1703 (vs), 1636 (m), 1603 (w), 1507 (w), 1435 (s), 1363 (w),

1243 (vs), 1193 (m), 1164 (m), 1107 (s), 1018 (m), 973 (m), 940 (w), 896 (w), 859 (m), 820 (m), 749 (s), 690 (w), 558 (w), 479 (m) cm^{-1}.

MS (ESI): *m / z* for $C_{22}H_{26}O_3$ calc.: 361.18 [M+Na], found: 361.18.

HRMS (ESI): *m / z* for $C_{22}H_{26}O_3$ calc.: 361.1774 [M+Na], found: 361.1775.

Methyl (2*E*,4*E*,8*R*,9*S*)-9-hydroxy-2,8-dimethyl-9-(pyridin-2-yl)nona-2,4-dienoate (*syn*-25b)

According to GP2, (1*S*,2*R*)-2-methyl-1-(pyridin-2-yl)hex-5-en-1-ol *syn*-**24b** (145 mg, 0.76 mmol) was treated with (2*E*,4*E)*-2-methy2-2,4-hexadienoate **15** (213 mg, 1.52 mmol) and Grubbs-II catalyst (128 mg, 0.15 mmol) in dichloromethane (20 mL). Column chromatography on silica (petroleum ether / EtOAc 3 : 1) yielded methyl (2*E*,4*E*,8*R*,9*S*)-9-hydroxy-2,8-dimethyl-9-(pyridin-2-yl)nona-2,4-dienoate *syn*-**25b (**49.2 mg, 0.17 mmol, 20 %, dr (*syn*/*anti)* = 100 : 0 (according to ^{1}H NMR)) as colourless oil.

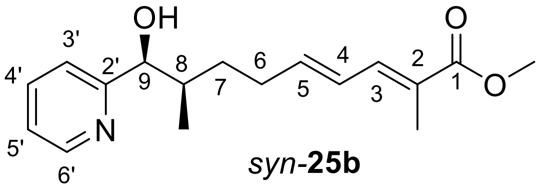

syn-**25b**

$C_{17}H_{23}NO_3$

289.38

R_f = 0.46 (petroleum ether / EtOAc 1 : 1)

$[\alpha]_D^{20} = 23.4°$ (c = 1.0, CH_2Cl_2)

^{1}H NMR (700 MHz, $CDCl_3$): δ = 0.67 (d, *J* = 7.0 Hz, 3H, 8-CH_3), 1.39 – 1.50 (m, 1H, 7-H_A), 1.70 – 1.78 (m, 1H, 7-H_B), 1.87 – 1.93 (m, 4H, 2-CH_3, 8-H), 2.24 – 2.47 (m, 2H, 6-H), 3.74 (s, 3H, OCH_3), 4.71 – 4.73 (m, 1H, 9-H), 6.03 – 6.13 (m, 1H, 5-H), 6.26 – 6.39 (m, 1H, 4-H), 7.14 – 7.23 (m, 3H, 3-H, 5’-H, 4’-H), 7.63 – 7.70 (m, 1H, 3’-H), 8.52 (d, *J* = 4.9 Hz, 1H, 6’-H) ppm.

^{13}C NMR (101 MHz, $CDCl_3$): δ = 12.6 (2-CH_3), 12.7 (8-CH_3), 31.1 (C-6), 32.9 (C-7), 39.4 (C-8), 51.7 (OCH_3), 74.8 (C-9), 120.7 (C-4’), 122.2 (C-5’), 124.9 (C-2’), 126.1 (C-4), 136.6 (C-3’), 138.8 (C-3), 143.0 (C-5), 147.8 (C-6’), 161.1(C-2’), 169.2 (C-1) ppm.

FT-IR (ATR): $\tilde{\nu}$ = 3403 (br), 3052 (w), 2949 (m), 2928 (m), 2875 (m), 2855 (m), 2182 (w), 2039 (w), 1999 (w), 1937 (w), 1702 (vs), 1636 (s), 1593 (s), 1570 (m), 1434 (vs), 1406 (m), 1242 (vs), 1192 (s), 1152 (s), 1105 (vs), 1046 (s), 996 (s), 974 (s), 939 (m), 831 (w), 750 (vs), 691 (w), 674 (w), 625 (w), 489 (w) cm^{-1}.

MS (ESI): *m / z* for $C_{21}H_{24}O_3$ calc.: 290.18 [M+H], found: 323.17.

HRMS (ESI): *m / z* for $C_{21}H_{24}O_3$ calc.: 290.1751 [M-H], found: 290.1753.

(2*E*,4*E*,8*R*,9*R*)-9-Hydroxy-2,8-dimethyl-9-(pyridin-2-yl)nona-2,4-dienoate (*anti*-25b)

According to GP2, (1*S*,2*S*)-2-methyl-1-(pyridin-2-yl)hex-5-en-1-ol *anti*-**24b** (165 mg, 0.86 mmol) was treated with (2*E*,4*E)*-2-methy2-2,4-hexadienoate **15** (242 mg, 1.73 mmol) and Grubbs-II catalyst (146 mg, 0.17 mmol) in dichloromethane (20 mL). Column chromatography on silica (petroleum ether / EtOAc 3 : 1) yielded methyl (2*E*,4*E*,8*R*,9*R*)-9-hydroxy-2,8-

dimethyl-9-(pyridin-2-yl)nona-2,4-dienoate *anti*-**25b** (49.9 mg, 0.17 mmol, 20 %, dr (*syn*/*anti)* = 0 : 100 (according to ^{1}H NMR)) as colourless oil.

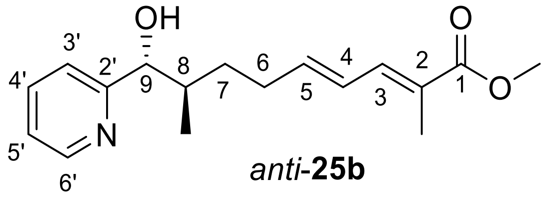

anti-**25b**

$C_{17}H_{23}NO_3$

289.38

R_f = 0.43 (petroleum ether / EtOAc 1 : 1).

$[\alpha]_D^{20} = -22.5°$ (c = 1.0, CH_2Cl_2).

^{1}H NMR (700 MHz, $CDCl_3$): δ = 0.93 – 1.00 (m, 3H, 8-CH_3), 1.25 – 1.33 (m, 1H, 7-H_A), 1.39 – 1.50 (m, 1H, 7-H_B), 1.88 – 1.93 (m, 4H, 2-CH_3, 8-H), 1.99 – 2.07 (m, 1H, 6-H_A), 2.14 – 2.30 (m, 1H, 6-H_B), 3.70 – 3.80 (m, 3H, OCH_3), 4.53 – 4.67 (m, 1H, 9-H), 5.88 – 6.02 (m, 1H, 5-H), 6.18 – 6.32 (m, 1H, 4-H), 7.10 (d, *J* = 11.2 Hz, 1H, 3-H), 7.17 – 7.23 (m, 2H, 4'-H, 5'-H), 7.60 – 7.75 (m, 1H, 3'-H), 8.48 – 8.62 (m, 1H, 6'-H) ppm.

^{13}C NMR (101 MHz, $CDCl_3$): δ = 12.6 (2-CH_3), 16.3 (8-CH_3), 29.8 (C-6), 30.9 (C-7), 39.6 (C-8), 51.7 (OCH_3), 76.9 (C-9), 121.3 (C-4'), 122.4 (C-5'), 124.8 (C-2'), 126.0 (C-4), 136.7 (C-3'), 138.8 (C-3), 143.0 (C-5), 148.0 (C-6'), 161.0 (C-2'), 169.2 (C-1) ppm.

FT-IR (ATR): $\tilde{\nu}$ = 3399 (br), 2951 (m), 2928 (m), 2874 (m), 2065 (w), 1939 (w), 1702 (vs), 1636 (s), 1593 (s), 1570 (m), 1434 (vs), 1388 (m), 1240 (vs), 1193 (s), 1159 (vs), 1105 (s), 1044 (s), 999 (s), 974 (s), 940 (m), 831 (w), 750 (vs), 691 (w), 674 (w), 623 (w), 552 (w), 489 (w), cm^{-1}.

MS (ESI): *m / z* for $C_{17}H_{23}N_1O_3$ calc.: 290.18 [M+H], found: 323.17.

HRMS (ESI): *m / z* for $C_{17}H_{23}N_1O_3$ calc.: 290.1751 [M+H], found: 290.1744.

Methyl (2*E*,4*E*,8*R*)-9-hydroxy-2,8-dimethyl-9-(thiophen-2-yl)nona-2,4-dienoate (25c)

According to GP2, (2*R*)-2-methyl-1-(thiopen-2-yl)hex-5-en-1-ol **24c** (190 mg, 0.97 mmol) was treated with (2*E*,4*E)*-2-methy2-2,4-hexadienoate **15** (271 mg, 1.94 mmol) and Grubbs-II catalyst (123 mg, 0.15 mmol) in dichloromethane (20 mL). Column chromatography on silica (petroleum ether / EtOAc 10 : 1) yielded methyl (2*E*,4*E*,8*R*)-9-hydroxy-2,8-dimethyl-9-(thiophen-2-yl)nona-2,4-dienoate **25c (**171 mg, 0.58 mmol, 44 %, dr (*syn*/*anti*) = 54 : 46) as colourless oil.

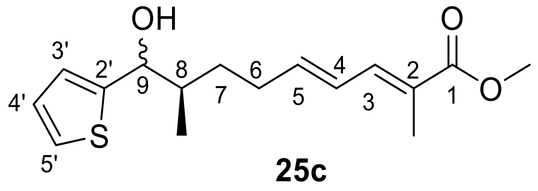

25c

$C_{16}H_{22}O_3S$

294,41

R_f = 0.34 (petroleum ether / EtOAc 5 : 1).

^{1}H NMR (400 MHz, $CDCl_3$) δ = 0.88 (d, *J* = 6.8 Hz, 3H,8-CH_3*), 1.01 (d, *J* = 6.8 Hz, 3H, 8-CH_3), 1.20 – 1.38 (m, 2H, 7-H_A, 7-H_A*), 1.51 – 1.62 (m, 1H, 7-H_B), 1.78 – 1.96 (m, 9H, 7-H_B*,

8-H, 8-H*, 2-CH_3, 2-CH_3 *), 2.10 – 2.23 (m, 1H, 6-H), 2.23 – 2.39 (m, 1H, 6-H*), 3.75 (s, 6H, OCH_3, OCH_3*), 4.73 (d, J = 6.8 Hz, 1H, 9-H*), 4.79 (d, J = 6.0 Hz, 1H, 9-H), 5.95 – 6.15 (m, 2H, 5-H, 5-H*), 6.24 – 6.45 (m, 2H, 4-H, 4-H*), 6.91 – 7.02 (m, 4H, 3'-H, 4'-H, 3'-H*, 4'-H*), 7.11 – 7.21 (m, 2H, 3-H, 3-H*), 7.21 – 7.31 (m, 2H, 5'-H, 5'-H*) ppm.

^{13}C NMR (101 MHz, $CDCl_3$) δ = 12.6 (2-CH_3), 14.7 (8-CH_3), 15.6 (8-CH_3*), 30.7 (C-6*), 30.9 (C-6), 31.5 (C-7*), 32.1 (C-7), 40.2 (C-8), 40.3 (C-8*), 51.8 (OCH_3), 74.4 (C-9), 74.9 (C-9*), 124.2 (C-4'), 124.4 (C-4'*), 124.5 (C-5), 124.6 (C-5*), 126.1 (C-4), 126.2 (C-4*), 126.5 (C-5'), 126.6 (C-5'*), 138.7 (C-3), 138.8 (C-3*), 142.7 (C-3'), 142.9 (C-3'*), 147.3 (C-2'), 147.5 (C-2'*), 169.1 (C-1), 169.2 (C-1*) ppm.

FT-IR (ATR): $\tilde{\nu}$ = 3470 (br), 2949 (m), 2928 (m), 2875 (m), 1702 (vs), 1637 (m), 1608 (m), 1435 (s), 1388 (m), 1289 (s), 1243 (vs), 1193 (m), 1163 (m), 1107 (s), 1018 (m), 973 (s), 939 (w), 852 (w), 829 (m), 752 (m), 698 (s), 530 (w) cm^{-1}.

MS (ESI): m / z for $C_{16}H_{22}O_3S$ calc.: 317.12 [M+Na], found: 317.12.

HRMS (ESI): m / z for $C_{16}H_{22}O_3S$ calc.: 317.1182 [M+Na], found: 317.1185.

(2*E*,4*E*)-9-Hydroxy-2-methyl-9-phenylnona-2,4-dienoic acid (22a)

According to GP2, 1-phenylhex-5-en-1-ol **S10a** (375 mg, 2.13 mmol) was treated with (2*E*,4*E*)-2-methylhexa-2,4-dienoic acid **9** (537 mg, 4.26 mmol) and Grubbs-II catalyst (45.0 mg, 0.05 mmol) in dichloromethane (40 mL). Column chromatography on silica (petroleum ether / EtOAc 5 : 1) yielded (2*E*,4*E*)-9-hydroxy-2-methyl-9-phenylnona-2,4-dienoic acid **22a (**500 mg, 1.92 mmol, 90 %, dr (*syn*/*anti*) = 51 : 49) as colourless oil.

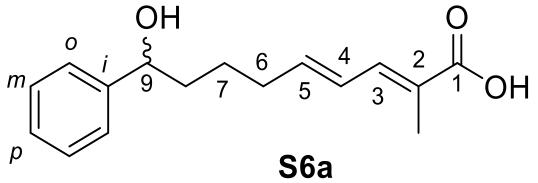

S6a

$C_{16}H_{20}O_3$

260.33

R_f = 0.16 (petroleum ether / EtOAc = 3 : 1, molybdatophosphoric acid stain).

^{1}H NMR (500 MHz, $CDCl_3$): δ = 1.40-1.65 (m, 2H, 7-H), 1.70-1.86 (m, 2H, 8-H), 1.91 (s, 3-H, 2-CH_3), 2.23 (td, J = 7.3, 7.3 Hz, 2H, 6-H), 4.69 (t, J = 6.8 Hz, 1H, 9-H), 6.09 (dt, J =15.2, 7.3 Hz, 1H, 5-H), 6.33 (dd, J =15.2, 11.3 Hz, 1H, 4-H), 7.25 (d, J = 11.3 Hz, 1H, 3-H), 7.27-7.30 (m, 1H, *p*-H), 7.32-7.36 (m, 4H, *o*-H, *m*-H) ppm.

^{13}C NMR (125 MHz, $CDCl_3$): δ = 12.2 (2-CH_3), 25.1 (C-7), 33.1 (C-6), 38.5 (C-8), 74.5 (C-9), 124.3 (C-2), 125.9 (C-*o* / C-*m*), 126.3 (C-4), 127.7 (C-*p*), 128.5 (C-*m* / C-*o*), 140.8 (C-3), 143.8 (C-5), 144.6 (C-*i*), 173.7 (C-1) ppm.

FT-IR (ATR): $\tilde{\nu}$ = 3029 (m), 2930 (m), 2860 (m), 2650 (w), 1673 (vs), 1637 (s), 1604 (m), 1494 (w), 1453 (w), 1420 (m), 1258 (s), 1115 (w), 1062 (w), 1025 (w), 974 (s), 912 (m), 815 (w), 760 (m), 700 (s), 675 (w), 577 (w), 553 (w) cm^{-1}.

MS (ESI): m / z for $C_{16}H_{20}O_3$ calc.: 259.1 [M-H]-, found: 259.1.

HRMS (ESI): m / z for $C_{16}H_{20}O_3$ calc.: 259.1329 [M-H]-, found: 259.1355.

(2*E*,4*E*)-10-Hydroxy-2-methyl-10-phenyldeca-2,4-dienoic acid (22b)

According to GP2, 1-phenylhept-6-en-1-ol **S10b** (480 mg, 2.52 mmol) was treated with (2*E*,4*E*)-2-methylhexa-2,4-dienoic acid **9** (636 mg, 5.04 mmol) and Grubbs-II catalyst (53.0 mg, 0.06 mmol) in dichloromethane (40 mL). Column chromatography on silica (petroleum ether / EtOAc 3 : 1) yielded (2*E*,4*E*)-10-hydroxy-2-methyl-10-phenyldeca-2,4-dienoic acid **22b (**700 mg, 2.46 mmol, 98 %, dr (*syn*/*anti*) = 50 : 50) as colourless oil.

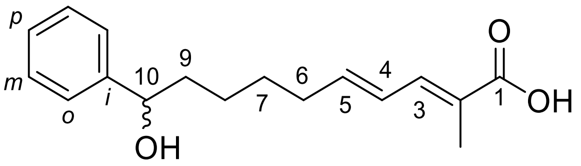

22b

$C_{17}H_{22}O_3$

274.36

R_f = 0.18 (petroleum ether / EtOAc = 3 : 1, molybdatophosphoric acid stain)

^{1}H NMR (500 MHz, $CDCl_3$): δ = 1.29-1.53 (m, 4H, 7-H, 8-H), 1.67-1.86 (m, 2H, 9-H), 1.92 (s, 3-H, 2-CH_3), 2.20 (d, *J* = 6.8 Hz, 2H, 6-H), 4.67 (t, *J* = 6.8 Hz, 1H, 10-H), 6.05-6.14 (m, 1H, 5-H), 6.29-6.37 (m, 1H, 4-H), 7.23-7.26 (m, 1H, 3-H), 7.26-7.30 (m, 1H, *p*-H), 7.32-7.36 (m, 4H, *o*-H, *m*-H) ppm.

^{13}C NMR (125 MHz, $CDCl_3$): δ = 12.2 (2-CH_3), 25.4 (C-8), 28.8 (C-7), 33.2 (C-6), 38.8 (C-9), 74.6 (C-10), 124.1 (C-2), 125.9 (C-*o* / C-*m*), 126.1 (C-4), 127.6 (C-*p*), 128.5 (C-*m* / C-*o*), 140.9 (C-3), 144.2 (C-5), 144.8 (C-*i*), 173.6 (C-1) ppm.

FT-IR (ATR): $\tilde{\nu}$ = 3400 (w), 3028 (w), 2933 (s), 2860 (m), 2168 (w), 1682 (vs), 1494 (w), 1453 (m), 1248 (s), 975 (s), 760 (m), 701 (s), 559 (w), 443 (w), 421 (w) cm^{-1}.

MS (ESI): *m / z* for $C_{17}H_{22}O_3$ calc.: 273.15 [M-H]-, found: 273.15.

HRMS (ESI): *m / z* for $C_{17}H_{22}O_3$ calc.: 273.1485 [M-H]-, found: 273.1524.

(2*E*,4*E*,8*R*)-9-hydroxy-2,8-dimethyl-10-phenyldeca-2,4-dienoic acid (20)

According to GP2, (3*R*)-3-methyl-1-phenylhept-6-en-2-ol **19** (350 mg, 1.71 mmol) was treated with (2*E*,4*E*)-2-methylhexa-2,4-dienoic acid **9** (432 mg, 3.43 mmol) and Grubbs-II catalyst (44.0 mg, 51.3µmol) in dichloromethane (30 mL). Column chromatography on silica (petroleum ether / EtOAc 5 : 1) yielded (2*E*,4*E*,8*R*)-9-hydroxy-2,8-dimethyl-10-phenyldeca-2,4-dienoic acid **20 (**380 mg, 1.32 mmol, 77 %, dr (*syn*/*anti*) = 63 : 37) as colourless oil.

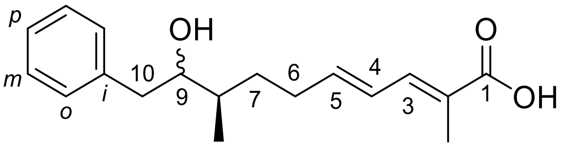

20

$C_{18}H_{24}O_3$

288.39

R_f = 0.25 (petroleum ether / EtOAc = 3 : 1, molybdatophosphoric acid stain)

^{1}H NMR (500 MHz, $CDCl_3$): δ = 1.00 (d, *J* = 6.9 Hz, 3H, 8-CH_3), 1.34-1.43 (m, 1H, 7-H_a), 1.59-1.72 (m, 2H, 7-H_b, 8-H), 1.93 (s, 3H, 2-CH_3), 2.15-2.38 (m, 2H, 6-H), 2.55-2.71 (m, 1H,

$10\text{-}H_a$), 2.77-2.89 (m, 1H, $10\text{-}H_b$), 3.63-3.79 (m, 1H, 9-H), 6.08-6.18 (m, 1H, 5-H), 6.32-6.43 (m, 1H, 4-H), 7.20-7.26 (m, 4H, *o*-H, *p*-H, 3-H), 7.29-7.34 (m, 2H, *m*-H) ppm.

^{13}C NMR (125 MHz, $CDCl_3$): δ = 12.2 (2-CH_3), 13.8 (3-CH_3), 31.1 (C-6), 32.4 (C-7), 37.3 (C-8), 41.0 (C-10), 75.8 (C-9), 124.2 (C-2), 126.1 (C-4), 126.5 (C-*p*), 128.7 (C-*m*), 129.3 (C-*o*), 139.0 (C-*i*), 140.9 (C-3), 144.1 (C-5), 173.6 (C-1) ppm.

FT-IR (ATR): $\tilde{\nu}$ = 3027 (m), 2925 (s), 2033 (w), 2011 (w), 1678 (vs), 1637 (s), 1604 (m), 1495 (m), 1453 (m), 1421 (m), 1254 (s), 1111 (m), 1030 (m), 976 (s), 743 (m), 700 (s), 33, 576 (w) cm^{-1}.

MS (ESI): *m / z* for $C_{18}H_{24}O_3$ calc.: 287.17 [M-H]-, found: 287.17.

HRMS (ESI): *m / z* for $C_{18}H_{24}O_3$ calc.: 287.1653 [M-H]-, found: 287.1656.

(2*E*,4*E*,8*R*)-9-Hydroxy-2,8-dimethyl-9-(naphthalen-2-yl)nona-2,4-dienoic acid (S13a)

According to GP3, methyl (2E,4E,8R)-9-hydroxy-2,8-dimethyl-9-(napthalen-2-yl)nona-2,4-dienoate **25a** (90 mg, 0.27 mmol) was treated with potassium hydroxide (209 mg, 3.72 mmol) in demin. water (20 mL) and THF (20 mL). Purification via preparative HPLC on a reversed phase column Orbit 100 C18 5 µm (250 x 20 mm) with a flow rate of 10 $mLmin^{-1}$ and a solvent gradient of MeOH / H_2O of 75 : 25 to 95 : 5 yielded the desired product (2*E*,4*E*,8*R*)-9-hydroxy-2,8-dimethyl-9-(naphtalen-2-yl)nona-2,4-dienoic acid **S13a** (60 mg, 0.19 mmol, 70 %, dr (*syn*/*anti*) = 69 : 31) as colourless foam.

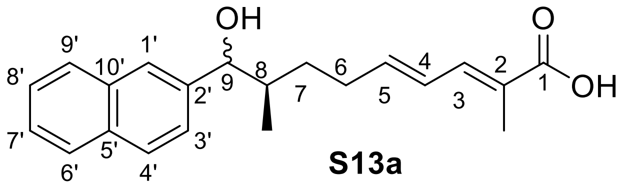

S13a

$C_{21}H_{24}O_3$

324.42

^{1}H NMR (700 MHz, $CDCl_3$): δ = 0.83 (d, *J* = 6.8 Hz, 3H, 8-Me*), 0.97 – 1.01 (m, 3H, 8-Me), 1.24 – 1.31 (m, 2H, 7-H_A, 7-H_A*), 1.50 – 1.59 (m, 2H, 7-H_B, 7-H_B*), 1.85 – 1.99 (m, 5H, 8-H, 8-H*, 2-Me), 2.13 – 2.22 (m, 3H, 2-Me*), 2.25 – 2.39 (m, 2H, 6-H_B; 6-H_B*), 4.57 – 4.64 (m, 1H, OH*), 4.67 – 4.74 (m, 1H, OH*), 6.00 – 6.15 (m, 2H, 4-H, 4-H*), 6.26 – 6.39 (m, 2H, 5-H, 5-H*), 7.19 – 7.31 (m, 2H, 3-H, 3-H*), 7.40 – 7.52 (m, 6H, 4'-H, 7'-H .9'-H, 4'-H*, 7'-H*, 9'-H*), 7.73 – 7.77 (m, 2H, 2'-H, 2'-H*),), 7.80 – 7.90 (m, 6H, 5'-H, 6'-H, 10'-H, 5'-H*, 6'-H*, 10'-H*) ppm.

^{13}C NMR (176 MHz, $CDCl_3$): δ = 11.2 (2-Me), 13.4 (2-Me*), 14.2 (8-Me), 14.8 (8-Me*), 29.8 (C-6), 30.0 (C-6*), 30.2 (C-7), 31.2 (C-7*), 38.5 (C-8), 64.9, 77.2 (C-9), 78.1 (C-9*), 123.1 (C-4'), 123.4 (C-4'*), 123.4 (C-2'), 123.5 (C-2'*), 123.6 (C-5), 124.2 (C-5*), 124.5 (C-7'), 124.5 (C-7'*), 124.8 (C-9'), 124.9 (C-9'*), 125.0 (C-6'), 125.1 (C-6'*), 125.2 (C-5'), 126.7 (C-5'*), 126.9 (C-4), 127.0 (C-4*), 127.1 (C-10'*), 127.1 (C-10'*), 131.9 (C-8'), 132.0 (C.-8'*), 132.1 (C-3'), 132.1 (C-3'*), 139.8 (C-1'), 139.9 (C-1'*), 139.9 (C-2), 140.0 (C-2*), 143.0 (C-3), 143.3 (C-3*), 172.3 (C-1) ppm.

FT-IR (ATR): $\tilde{\nu}$ = 3051 (br), 2962 (s), 2927 (s), 2646 (m), 2247 (w), 1673 (vs), 1635 (s), 1602 (m), 1508 (w), 1454 (w), 1419 (m), 1378 (m), 1258 (s), 1169 (w), 1121 (m), 1019 (m), 974 (s), 907 (s), 858 (m), 818 (s), 774 (w), 732 (s), 674 (w), 649 (w), 625 (w), 577 (w), 478 (m) cm^{-1}.

MS (ESI): *m / z* for $C_{21}H_{24}O_3$ calc.: 323.17 [M-H], found: 323.17.

HRMS (ESI): *m / z* for $C_{21}H_{24}O_3$ calc.: 323.1653 [M-H], found: 323.1653.

(2*E*,4*E*,8*R*)-9-Hydroxy-2,8-dimethyl-9-(thiophen-2-yl)nona-2,4-dienoic acid (S13c)

According to GP3, methyl (2E,4E,8R)-9-hydroxy-2,8-dimethyl-9-(thiophen-2-yl)nona-2,4-dienoate **25c** (70 mg, 0.24 mmol) was treated with potassium hydroxide (187 mg, 3.33 mmol) in demin. water (20 mL) and THF (20 mL). Purification via preparative HPLC on a reversed phase column Orbit 100 C18 5 µm (250 x 20 mm) with a flow rate of 10 $mLmin^{-1}$ and a solvent gradient of MeOH / H_2O of 75 : 25 to 95 : 5 yielded the desired product (2*E*,4*E*,8*R*)-9-hydroxy-2,8-dimethyl-9-(thiophen-2-yl)nona-2,4-dienoic acid **S13c** (30 mg, 0.11 mmol, 45 %, dr (*syn*/*anti*) = 55 : 45) as colourless foam.

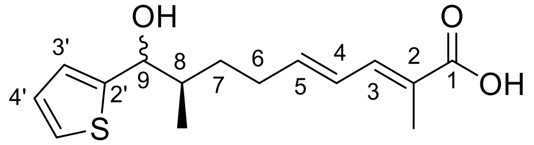

S13c

$C_{15}H_{20}O_3S$

280.38

^{1}H NMR (400 MHz, $CDCl_3$): δ = 0.88 (d, *J* = 6.6 Hz, 3H, 8-CH_3), 1.02 (d, *J* = 6.6 Hz, 3H, 8-CH_3*), 1.20 – 1.38 (m, 2H, 7-H_A, 7-H_A*), 1.52 – 1.65 (m, 1H, 7-H_B), 1.78 – 1.98 (m, 9H, 2-CH_3, 2-CH_3*, 7-H_B*, 8-H, 8-H*), 2.11 – 2.24 (m, 2H, 6-H_A, 6-H_A*), 2.24 – 2.43 (m, 2H, 6-H_B; 6-H_B*), 4.74 (d, *J* = 6.8 Hz, 1H, 9-H), 4.79 (d, *J* = 6.8, 1H, 9-H*), 6.02 – 6.18 (m, 2H. 5-H, 5-H*), 6.26 – 6.43 (m, 2H. 4-H, 4-H*), 6.93 – 7.01 (m, 6H, 3'-H, 4'-H, 5'-H, 3'-H*, 4'-H*, 5'-H*), 7.22 – 7.31 (m, 2H, 3-H, 3-H*) ppm.

^{13}C NMR (101 MHz, $CDCl_3$): δ = 12.2 (2-CH_3), 14.7 (8-CH_3), 15.6 (8-CH_3*), 30.9 (C-6*), 30.9 (C-6), 31.4 (C-7*), 32.0 (C-7), 40.2 (C-8), 40.2 (C-8*), 74.5 (C-9), 74.8 (C-9*), 124.2 (C-4'), 124.4 (C-4'*), 124.5 (C-5), 124.6 (C-5*), 126.1 (C-4), 126.2 (C-4*), 126.5 (C-5'), 126.6 (C-5'*), 140.8 (C-3), 140.9 (C-3*), 144.0 (C-3), 144.2 (C-3'*), 147.3 (C-2'), 147.3 (C-2'*), 173.8 (C-1), 173.8 (C-1*) ppm.

FT-IR (ATR): $\tilde{\nu}$ = 3069 (br), 3031 (br), 2962 (m), 2926 (m), 2646 (m), 1672 (vs), 1635 (s), 1603 (m), 1417 (m), 1380 (m), 1256 (s), 1169 (w), 1118 (w), 975 (s), 910 (w), 852 (w), 828 (m), 754 (w), 732 (w), 699 (s), 578 (w), 445 (w) cm^{-1}.

MS (ESI): *m / z* for $C_{15}H_{20}O_3S$ calc.: 279.11 [M-H], found: 279.11.

HRMS (ESI): *m / z* for $C_{15}H_{20}O_3S$ calc.: 279.1060 [M-H], found: 279.1064.

(*R*,2*E*,4*E*)-2,8-dimethyl-9-oxo-9-(pyridin-2-yl)nona-2,4-dienoic acid (S15)

According to GP3, methyl (2*E*,4*E*,8*R*,9*S*)-9-hydroxy-2,8-dimethyl-9-(pyridin-2-yl)nona-2,4-dienoate *anti*-**25b** (40 mg, 0.14 mmol) was treated with potassium hydroxide (109 mg, 1.94 mmol) in demin. water (20 mL) and THF (20 mL). Purification via preparative HPLC on a reversed phase column Orbit 100 C18 5 µm (250 x 20 mm) with a flow rate of 10 $mLmin^{-1}$

and a solvent gradient of MeOH / H_2O of 75 : 25 to 95 : 5 yielded (*R*,2*E*,4*E*)-2,8-dimethyl-9-oxo-9-(pyridin-2-yl)nona-2,4-dienoic acid **S15** (7.00 mg, 0.03 mmol, 19 %) as colourless foam.

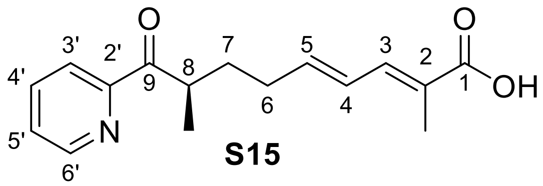

S15

$C_{16}H_{19}NO_3$

273.33

^{1}H NMR (700 MHz, $CDCl_3$): δ = 1.12 – 1.15 (m, 3H, 8-CH_3), 1.47 – 1.58 (m, 1H, 7-H_A), 1.78 – 1.82 (m, 3H, 2-CH_3), 1.89 – 1.97 (m, 1H, 7-H_B), 2.16 – 2.22 (m, 2H, 6-H), 4.02 – 4.11 (m, 1H, 8-H), 5.99 – 6.08 (m, 1H, 5-H), 6.16 – 6.27 (m, 1H, 4-H), 7.13 – 7.18 (m, 1H, 3-H), 7.36 – 7.44 (m, 1H, 5’-H), 7.73 – 7.80 (m, 1H, 4’-H), 7.94 – 8.00 (m, 1H, 3’-H), 8.57 – 8.66 (m, 1H, 6’-H) ppm.

^{13}C NMR (176 MHz, $CDCl_3$): δ = 12.2 (2-CH_3), 17.0 (8-CH_3), 31.2 (C-6), 32.2 (C-7), 38.6 (C-8), 122.5 (C-3’), 124.3 (C-2), 126.4 (C-4), 127.0 (C-5’), 137.0 (C-4’), 140.8 (C-3), 143.7 (C-5), 149.0 (C-6’), 153.0 (C-2’), 173.5 (C-9), 205.3 (C-1) ppm.

FT-IR (ATR): $\tilde{\nu}$ = 2925 (s), 2855 (s), 2167 (w), 2095 (w), 2026 (w), 1692 (vs), 1637 (s), 1604 (m), 1583 (m), 1568 (m), 1455 (m), 1423 (m), 1373 (m), 1260 (s), 1226 (s), 1114 (m), 995 (s), 978 (s), 808 (m), 745 (s), 701 (s), 674 (s), 618 (s), 577 (w), 531 (w), 423 (w) cm^{-1}.

MS (ESI): *m / z* for $C_{16}H_2N_1O_3$ calc.: 274.14 [M+H], found: 274.14.

HRMS (ESI): *m / z* for $C_{16}H_2N_1O_3$ calc.: 274.1426 [M+H], found: 274.1438.

(3*E*,5*E*,13*E*,15*E*)-3,13-Dimethyl-10,20-diphenyl-1,11-dioxacycloicosa-3,5,13,15-tetraene-2,12-dione (23a)

According to GP4, (2*E*,4*E*)-9-hydroxy-2-methyl-9-phenylnona-2,4-dienoic acid **22a** (150 mg, 0.58 mmol) was treated with NEt_3 (96.0µL, 69.8 mg, 0.69 mmol), 2,4,6-trichlorobenzoyl chloride (108µL, 168 mg, 0.69 mmol) and 4-dimethylaminopyridine (169 mg, 1.38 mmol) in dry THF (10 mL) and dry DMF (10 mL). Column chromatography on silica (petroleum ether / EtOAc 5 : 1) yielded the desired product (3*E*,5*E*,13*E*,15*E*)-3,13-dimethyl-10,20-diphenyl-1,11-dioxacycloicosa-3,5,13,15-tetraene-2,12-dione **23a** (35.0 mg, 72.2µmol, 25 % (mixture of *(R,R), (R,S), (S,S)*) as colourless oil.

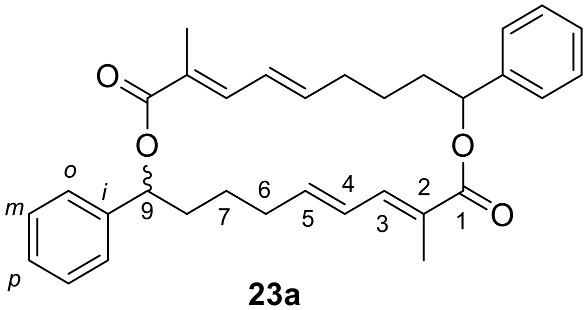

23a

$C_{32}H_{36}O_4$

484.64

R_f = 0.44 (petroleum ether / EtOAc = 3 : 1, molybdatophosphoric acid stain)

^{1}H NMR (500 MHz, $CDCl_3$): δ = 1.40-1.60 (m, 4H, 7-H), 1.78-1.99 (m, 4H, 8-H), 1.93 (s, 6-H, 2-CH_3), 2.10-2.34 (m, 4H, 6-H), 5.77-5.91 (m, 2H, 9-H), 6.00-6.12 (m, 2H, 5-H), 6.26-6.39 (m, 2H, 4-H), 7.14-7.25 (m, 2H, 3-H), 7.27-7.30 (m, 2H, *p*-H), 7.31-7.37 (m, 8H, *o*-H, *m*-H) ppm.

^{13}C NMR (125 MHz, $CDCl_3$): δ = 12.7 (2-CH_3), 25.2 (C-7), 33.1 (C-6), 36.2 (C-8), 75.7 (C-9), 125.5 (C-2), 126.2 (C-*o* / C-*m*), 126.8 (C-4), 127.8 (C-*p*), 128.5 (C-*m* / C-*o*), 138.4 (C-3), 141.1 (C-*i*), 142.4 (C-5), 167.7 (C-1) ppm.

FT-IR (ATR): $\tilde{\nu}$ = 3058 (m), 3025 (m), 2929 (m), 2867 (m), 2645 (w), 1759 (m), 1674 (vs), 1635 (s), 1600 (m), 1492 (m), 1419 (s), 1387 (m), 1387 (w), 1365 (w), 1262 (vs), 1117 (m), 1074 (m), 1047 (w), 1030 (w), 967 (s), 875 (m), 817 (m), 754 (s), 700 (vs), 672 (w), 573 (w), 527 (w), 493 (w) cm^{-1}.

MS (ESI): *m / z* for $C_{32}H_{36}O_4$ calc.: 483.3 [M-H]-, found: 483.3.

HRMS (ESI): *m / z* for $C_{32}H_{36}O_4$ calc.: 483.2530 [M-H]-, found: 483.2537.

(3*E*,5*E*,14*E*,16*E*)-3,14-Dimethyl-11,22-diphenyl-1,12-dioxacyclodocosa-3,5,14,16-tetraene-2,13-dione (23b)

According to GP4, (2*E*,4*E*)-10-hydroxy-2-methyl-10-phenyldeca-2,4-dienoic acid **22b** (100 mg, 0.58 mmol) was treated with NEt_3 (64.0µL, 47.0 mg, 0.46 mmol), 2,4,6-trichlorobenzoyl chloride (82.0µL, 128 mg, 0.53 mmol) and 4-dimethylaminopyridine (128 mg, 1.05 mmol) in dry THF (10 mL) and dry benzene (20 mL). Column chromatography on silica (petroleum ether / EtOAc 5 : 1) yielded the desired product (3*E*,5*E*,14*E*,16*E*)-3,14-dimethyl-11,22-diphenyl-1,12-dioxacyclodocosa-3,5,14,16-tetraene-2,13-dione **23b** (11 mg, 21.5µmol, 12 %, (mixture of *(R,R), (R,S), (S,S)*) as colourless oil.

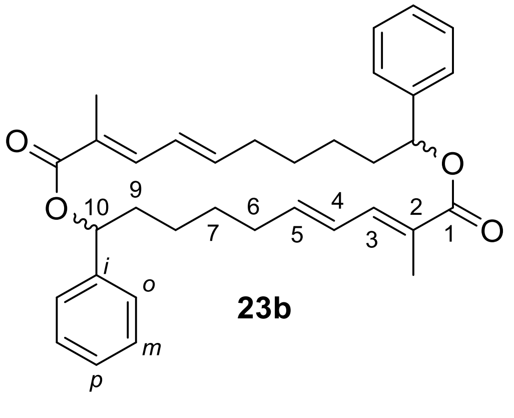

$C_{34}H_{40}O_4$

512.69

R_f = 0.44 (petroleum ether / EtOAc = 10 : 1, molybdatophosphoric acid stain).

^{1}H NMR (500 MHz, $CDCl_3$): δ = 1.22-1.53 (m, 8H, 7-H, 8-H), 1.76-1.96 (m, 4H, 9-H), 1.92 (s, 6H, 2-CH_3), 2.16-2.36 (m, 4H, 6-H), 5.85-6.02 (m, 2H, 10-H), 6.03-6.21 (m, 2H, 5-H), 6.33-6.40 (m, 2H, 4-H), 7.21-7.29 (m, 2H, 3-H), 7.31-7.36 (m, 8H, *o*-H, *m*-H), 7.39-7.42 (m, 2H, *p*-H) ppm.

^{13}C NMR (125 MHz, $CDCl_3$): δ = 12.7 (2-CH_3), 25.1 (C-8), 28.2 (C-7), 33.3 (C-6), 35.8 (C-9), 78.5 (C-10), 123.3 (C-2), 125.8 (C-*o* / C-*m*), 126.8 (C-4), 128.0 (C-*p*), 128.4 (C-*m* / C-*o*), 138.1 (C-3), 141.5 (C-*i*), 142.4 (C-5), 162.5 (C-1) ppm.

FT-IR (ATR): $\tilde{\nu}$ = 2928 (s), 2856 (m), 2860 (m), 2240 (w), 2018 (m), 1978 (m), 1739 (vs), 1579 (s), 1548 (s), 1455 (m), 1371 (s), 1270 (vs), 1210 (s), 1121 (s), 1083 (w), 988 (s), 857 (m), 822 (m), 760 (m), 700 (m), 589 (w), 546 (w), 471 (w), 429 (w) cm^{-1}.

MS (ESI): *m / z* for $C_{32}H_{36}O_4$ calc.: 513.3 $[M+H]^+$, found: 513.3.

HRMS (ESI): *m / z* for $C_{32}H_{36}O_4$ calc.: 513.2999 $[M+H]^+$, found: 513.2969.

(3*E*,5*E*,9*R*,13*E*,15*E*,19*R*)-10-benzyl-3,9,13,19-tetramethyl-20-phenyl-1,11-dioxacycloicosa-3,5,13,15-tetraene-2,12-dione (21)

According to GP4, (2*E*,4*E*,8*R*)-9-hydroxy-2,8-dimethyl-10-phenyldeca-2,4-dienoic acid **20** (150 mg, 0.52 mmol) was treated with NEt_3 (86.0µL, 63.1 mg, 0.62 mmol), 2,4,6-trichlorobenzoyl chloride (122µL, 190 mg, 0.78 mmol) and 4-dimethylaminopyridine (190 mg, 1.56 mmol) in dry THF (20 mL) and dry benzene (30 mL). Column chromatography on silica (petroleum ether / EtOAc 10 : 1) yielded the desired product (3*E*,5*E*,9*R*,13*E*,15*E*,19*R*)-10-benzyl-3,9,13,19-tetramethyl-20-phenyl-1,11-dioxacycloicosa-3,5,13,15-tetraene-2,12-dione **21** (50.0 mg, 92.5 µmol, 36 %, (mixture of *syn,syn- / syn,anti- / anti,anti*-**21**) as colourless oil.

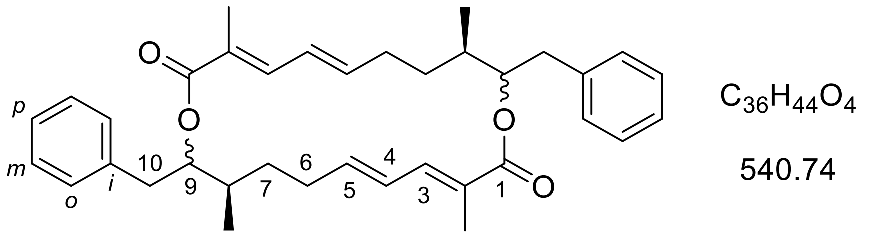

21

R_f = 0.63 (petroleum ether / EtOAc = 10 : 1, molybdatophosphoric acid stain)

^1H NMR (500 MHz, $CDCl_3$): δ = 0.95-1.07 (m, 6H, 8-CH_3), 1.19-1.39 (m, 2H, 7-H_a), 1.43-1.58 (m, 2H, 7-H_b), 1.63-1.75 (m, 2H, 8-H), 1.77-1.92 (m, 6H, 2-CH_3), 2.07-2.46 (m, 4H, 6-H), 2.76-2.98 (m, 4H, 10-H), 4.99-5.20 (m, 2H, 9-H), 5.88-6.11 (m, 2H, 5-H), 6.19-6.39 (m, 2H, 4-H), 7.14-7.29 (m, 12H, *o*-H, *m*-H, *p*-H, 3-H) ppm.

^{13}C NMR (125 MHz, $CDCl_3$): δ = 12.4 (2-CH_3), 16.6 (3-CH_3), 30.6 (C-6), 32.8 (C-7), 35.1 (C-8), 37.8 (C-10), 77.7 (C-9), 125.3 (C-2), 126.2 (C-4), 126.3 (C-*p*), 128.3 (C-*m*), 129.2 (C-*o*), 138.0 (C-3), 138.4 (C-*i*), 142.2 (C-5), 167.8 (C-1) ppm.

FT-IR (ATR): $\tilde{\nu}$ = 3029 (w), 2928 (w), 2252 (w), 1793 (w), 1731 (m), 1697 (s), 1637 (m), 1604 (w), 1578 (w), 1549 (w), 1495 (w), 1454 (m), 1383 (m), 1368 (m), 1237 (s), 1154 (m), 1099 (s), 1007 (s), 972 (s), 908 (s), 859 (m), 820 (w), 729 (vs), 648 (w), 589 (w), 542 (w), 520 (w), 431 (w) cm^{-1}.

MS (ESI): *m / z* for $C_{36}H_{44}O_4$ calc.: 563.3 $[M+Na]^+$, found: 563.3.

HRMS (ESI): *m / z* for $C_{36}H_{44}O_4$ calc.: 563.3132 $[M+Na]^+$, found: 563.3135.

13.5 Synthesis of Superquat-Auxiliary (11)

(*R*)-1-methoxy-3-methyl-1-oxobutan-2-aminium chloride (S36)

D-valine (20.2 g, 173 mmol) was suspended in methanol (400 mL) at 0 °C and thionyl chloride (18.8 mL, 30.8 g, 259 mmol) was added dropwise. Stirring for 24 h at rt and concentration under reduced pressure provided the desired product (*R*)-1-methoxy-3-methyl-1-oxobutan-2-aminium chloride **S36** (28.9 g, 172 mmol, 99 %) as colourless solid.

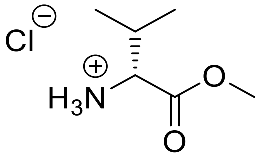

$C_6H_{14}ClNO_2$

167.63

S36

^{1}H NMR (400 MHz, $CDCl_3$): δ = 1.15 (t, J = 6.8 Hz, 6H, $(CH_3)_2$), 2.25 (m, 1H, C*H*$(CH_3)_2$), 3.83 (s, 3H, OCH_3), 3.89 – 3.97 (m, 1H, C*H*NH_3), 8.85 (br s, 3H, NH_3) ppm.

^{13}C NMR (100 MHz, $CDCl_3$): δ = 18.3 ($(CH_3)_2$), 29.9 (*C*$(CH_3)_2$), 53.0 (OCH_3), 58.6 (CNH_3), 168.8 (*C*$OOCH_3$) ppm.

The spectroscopic data is in agreement with the literature[32].

Methyl (*tert*-butoxycarbonyl)-D-valinate (S37)

(*R*)-1-Methoxy-3-methyl-1-oxobutan-2-aminium chloride **S36** (28.5 g, 170 mmol) was dissolved in ethanol (500 mL) at 0 °C and $NaHCO_3$ (42.9 g, 511 mmol) und Di-*tert*-butyldicarbonat (40.9 g, 187 mmol) were added in portions. The white suspension was stirred for 93 h, filtered over celite and washed with diethyl ether (100 mL). After concentration under reduced pressure the participate was again dissolved in diethyl ether (100 mL), filtered over celite and washed with diethyl ether (100 mL). Concentration under reduced pressure yielded the desired product methyl (*tert*-butoxycarbonyl)-D-valinate **S37** (26.6 g, 115 mmol) as colourless oil.

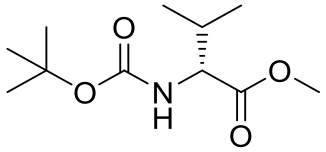

$C_{11}H_{21}NO_4$

231.29

S37

^{1}H NMR (400 MHz, $CDCl_3$): δ = 0.89 (d, J = 6.9 Hz, 3H, CHC*H*$_3$), 0.95 (d, J = 6.9 Hz, 3H, CHC*H*$_3$), 1.44 (s, 9H, $C(CH_3)_3$), 2.04 – 2.18 (m, 1H, C*H*$(CH_3)_2$), 3.73 (s, 3H, OCH_3), 4.15 – 4.27 (m, 1H, C*H*i*Pr), 5.01 (d, J = 8.8 Hz, 1H, NH) ppm.

^{13}C NMR (100 MHz, $CDCl_3$): δ = 16.6 (CH*C*H$_3$), 18.0 (CH*C*H$_3$), 27.4 (C(*C*H$_3$)$_3$), 30.3 (*C*H$(CH_3)_2$), 51.0 (OCH_3), 57.5 (C*i*Pr), 78.8 (*C*H$(CH_3)_2$), 154.7 (COO*t*Bu), 172.0 ($COOCH_3$) ppm.

The spectroscopic data is in agreement with the literature[32].

tert-Butyl (R)-(2-hydroxy-2,4-dimethylpentan-3-yl)carbamate (S38)

Under an inert atmosphere (N_2), magnesium (9.08 g, 374 mmol) was suspended in diethyl ether (120 mL) and iodomethane (23.3 mL, 52.0 g, 374 mmol) was added dropwise. After stirring for 30 min at rt, methyl (*tert*-butoxycarbonyl)-D-valinate **S37** (21.6 g, 93.4 mmol), dissolved in diethyl ether (100 mL), was added and the reaction mixture was stirred for 21 h at rt before adding the mixture to sat. NH_4Cl-solution (300 mL). The formed precipitate was dissolved in conc. HCl (50 mL), extracted with diethyl ether (3 x 150 mL), washed with sat. NaCl-solution and dried with $MgSO_4$. Removing the solvent under reduced pressure yielded the desired product *tert*-butyl (*R*)-(2-hydroxy-2,4-dimethylpentan-3-yl)carbamate **S38** (19.4 g, 83.9 mmol, 90 %) as colourless oil.

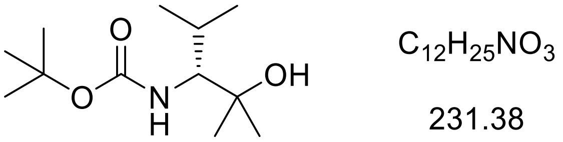

S38

R_f = 0.40 (petroleum ether / EtOAc = 5 : 1, molybdatophosphoric acid stain)

^{1}H NMR (400 MHz, $CDCl_3$): δ = 0.90 (d, J = 6.8 Hz, 3H, CHCH_3), 0.95 (d, J = 6.8 Hz, 3H, CHCH_3), 1.23 (d, J = 13.8 Hz, 6H, CO(CH_3)$_2$), 1.45 (s, 9H, C(CH_3)$_3$), 1.50 - 1.59 (br s, 1H, OH), 2.05 – 2.15 (m, 1H, CH(CH$_3$)$_2$), 3.39 (dd, J = 10.2 Hz, 2.7 Hz, 1H, CH*i*Pr), 4.81 (br d, J = 10.2 Hz, 1H, NH) ppm.

^{13}C NMR (100 MHz, $CDCl_3$): δ = 16.9 (CHCH$_3$), 22.3 (COHCH$_3$), 27.1 (COHCH$_3$), 28.2 (C(CH$_3$)$_2$$CH_3$), 28.3 (C(CH$_3$)$_2$$CH_3$), 28.4 (C(CH$_3$)$_2$$CH_3$), 29.1 ($C$(CH$_3$)$_2$), 61.7 ($C$(CH$_3$)$_2$OH), 73.8 ($C$C(CH$_3$)$_2$), 79.1 ($C$(CH$_3$)$_3$), 157.0 ($C$OO(CH$_3$)$_3$) ppm.

The spectroscopic data is in agreement with the literature[32].

(R)-4-Isopropyl-5,5-dimethyloxazolidin-2-one (11)

Under an inert atmosphere (N_2), *tert*-butyl (*R*)-(2-hydroxy-2,4-dimethylpentan-3-yl)carbamate **S38** (19.4 g, 83.7 mmol) was dissolved in dry THF (300 mL), potassium *tert*-butanolate (12.2 g, 198.9 mmol) was added in portions at 0 °C and stirred for 1 h. The solvent was removed under reduced pressure and the precipitate was dissolved in EtOAc, washed with sat. NaCl-soltion (2 x 150 mL) and dried over $MgSO_4$. The desired product (*R*)-4-Isopropyl-5,5-dimethyloxazolidin-2-one **11** (12.3 g, 78.0 mmol, 93 %) was formed as colourless solid.

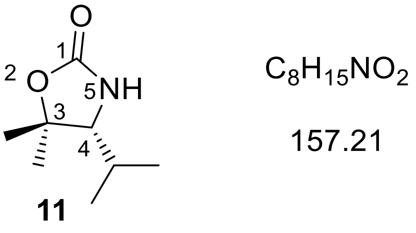

11

^{1}H NMR (400 MHz, $CDCl_3$): δ = 0.93 (d, J = 6.6 Hz, 3H, CHCH_3), 0.98 (d, J = 6.6 Hz, 3H, CHCH_3), 1.39 (s, 3H, 3-CH_3), 1.49 (s, 3H, 3-CH_3), 1.77 - 1.88 (m, 1H, 3-H), 3.18 (d, J = 8.6 Hz, 1H, 4-H), 5.51 (s, 1H, NH) ppm.

^{13}C NMR (100 MHz, $CDCl_3$): δ = 19.9 (CHCH_3), 20.0 (CHCH_3), 21.3 (3-CH_3), 28.5 (3-CH_3), 28.5 (C*H*Me$_2$), 68.3 (C-4), 83.9 (C-3), 158.5 (C-1) ppm.

The spectroscopic data is in agreement with the literature[32].

13.6 Synthesis of sorbic acid (9)

(*E*)-3-Hydroxy-2-methylhex-4-enoate (S39)

Under an inert atmosphere (N_2), zinc (2.25 g, 34.4 mmol, activated with HCl-solution (2 M), demin. water, aceton and diethyl ether) was suspended in dry benzene (5 mL), a mixture of *trans*-crotonaldehyde (2.60 mL 2.20 g, 31.3 mmol) and methyl-2-bromopropionate (3.34 mL, 5.00 g, 29.9 mmol) was added dropwise and refluxed for 4 h. The reaction mixture was added to H_2SO_4-solution (10 mL, 2 M) and the separated organic layer was washed with H_2SO_4-solution (10 mL, 2 M), sat. $NaHCO_3$-solution (10 mL) und demin. Wasser (10 mL). Drying over $MgSO_4$ and removing the solvent yielded the desired product methyl (*E*)-3-hydroxy-2-methylhex-4-enoate **S39** (2.72 g, 17.2 mmol, 58 %, dr (*syn*/*anti*) = 58 : 42 according to ^{1}H NMR) as colourless oil.

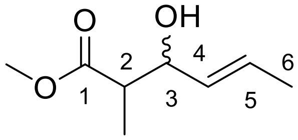

S39

$C_8H_{14}O_3$

158.20

^{1}H NMR (400 MHz, $CDCl_3$): δ = 1.14 (d, J = 7.2 Hz, 3H, 2-CH_3), 1.17 (d, J = 7.2 Hz, 3H, 2-CH_3*), 1.66 – 1.74 (m, 3H, 6-H), 2.42 – 2.66 (m, 2H, 2-H, OH), 3.66 – 3.73 (m, 3H, OCH_3), 4.14 (m, 1H, 3-H), 4.31 (m, 1H. 3-H*), 5.40 – 5.53 (m, 1H, 4-H / 5-H), 5.69 – 5.79 (m, 1H, 4-H / 5-H) ppm.

^{13}C NMR (100 MHz, $CDCl_3$): δ = 11.5 (2-CH_3), 14.1 (2-CH_3*), 17.7 (C-6), 17.7 (C-6*), 45.0 (C-2), 45.6 (C-2*), 51.7 (OCH_3), 51.8 (OCH_3*), 73.3 (C-3), 74.8 (C-3*), 128.4 (C-4 / C-5), 129.2 (C-4* / C-5*), 130.3 (C-4 / C-5), 131.1 (C-5* / C-4*), 175.8 (C-1), 176.1 (C-1*) ppm.

The spectroscopic data is in agreement with the literature[33].

(2*E*,4*E*)-2-Methylhexa-2,4-dienoic acid methyl ester (15)

Methyl (*E*)-3-hydroxy-2-methylhex-4-enoate **S39** (2.72 g, 17.2 mmol) was dissolved in dichloromethane (15 mL), Sicapent (3 g, phosphor pentoxide drying agent, Merck) was added and refluxed for 4 h. Filtration, washing with demin. water (100 mL) and drying over $MgSO_4$ yielded the product (2*E*,4*E)*-2-methy2-2,4-hexadienoate **15** (2.40 g, 17.1 mmol, quant.) as colourless oil.

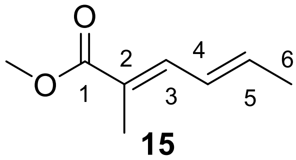

15

$C_8H_{12}O_2$

140.18

^{1}H NMR (400 MHz, $CDCl_3$): δ = 1.81 – 1.90 (m, 3H, 6-H), 1.90 – 1.97 (m, 3H. 2-CH_3), 3.62 – 3.78 (m, 3H, OCH_3), 5.99 - 6.22 (m, 1H, 5-H), 6.20 – 6.45 (m, 1H, 4-H), 7.16 (d, *J* = 11.3 Hz, 1H, 3-H) ppm.

^{13}C NMR (100 MHz, $CDCl_3$): δ = 12.5 (2-CH_3), 18.9 (C-6), 51.7 (OCH_3), 124.6 (C-2), 127.4 (C-4), 137.8 (C-5), 138.7 (C-3), 169.2 (C-1) ppm.

The spectroscopic data is in agreement with the literature[33].

(2*E*,4*E*)-2-methylhexa-2,4-dienoic acid (9)

(2*E*,4*E)*-2-methy2-2,4-hexadienoate **15** (2.40 g, 17.1 mmol) was dissolved in THF (20 mL) and demin. water (10 mL) and potassium hydroxide (6.72 g, 120 mmol) was added and stirred for 4 d at rt. The reaction mixture was acidified with conc. HCl (pH = 1) and extracted with EtOAc (3 x 50 mL). The organic phase was dried over $MgSO_4$ and the solvent was removed over reduced pressure. The desired product (2*E*,4*E*)-2-methylhexa-2,4-dienoic acid **9** (2.10 g, 16.7 mmol, 97 %) was formed as colourless oil.

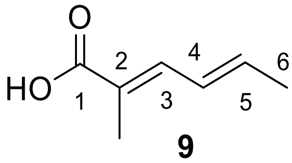

9

$C_7H_{10}O_2$

126.16

^{1}H NMR (400 MHz, $CDCl_3$): δ = 1.88 (d, *J* = 6.8 Hz, 3H, 6-H), 1.92 (s, 3H, 2-CH_3), 6.14 (dq, *J* = 13.9 Hz, 6.8 Hz, 1H, 5-H), 6.32 – 6.44 (m, 1H, 4-H), 7.29 (s, 1H, 3-H) ppm.

^{13}C NMR (100 MHz, $CDCl_3$): δ = 12.1 (2-CH_3), 18.9 (C-6), 124.0 (C-2), 127.4 (C-4), 139.2 (C-5), 140.9 (C-3), 174.4 (C-1) ppm.

The spectroscopic data is in agreement with the literature[33].

13.7 Compounds for optimization reactions

(*E*)-1-(4-Tosyloxazol-5-yl)pent-3-en-2-ol (Ts-S5)

Under an inert atmosphere (N_2), 5-Methyl-4-tosyloxazole **15** (570 mg, 2.40 mmol) was dissolved in dry THF (10 mL) and *n*-buthyllithium (2.11 mL, 5.29 mmol, 2.5M in hexane) was added dropwise at -78 °C. After stirring for 30 min, crotonaldehyde (0.21 mL, 177 mg, 2.52 mmol) was added at the same temperature, stirred for 1 h, poured onto water (20 mL) and extracted with EtOAc (3 x 20 mL). The combined organic layers were washed with sat. NaCl-solution (20 mL) and dried over $MgSO_4$. Column chromatography on silica (petroleum ether / EtOAc = 1 : 1) yielded the desired product (*E*)-1-(4-Tosyloxazol-5-yl)pent-3-en-2-ol Ts-**S5** (530 mg, 1.72 mmol, 72 %, dr (*syn*/*anti*) = 50 : 50) as colourless oil.

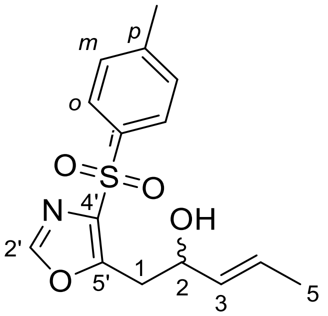

C15H17NO4Si

307.36

Ts-**S5**

R_f = 0.60 (petroleum ether / EtOAc = 1 : 1, molybdatophosphoric acid stain)

^{1}HNMR (400 MHz, $CDCl_3$): δ = 1.70 (d, J = 6.4 Hz, 3H, 5-H), 1.94 (br s, 1H, OH), 2.42 (s, 3H, p-CH_3), 3.29-3.39 (m, 2H, H-1), 4.44-4.51 (m, 1H, H-2), 5.58 (ddq, J = 15.2 Hz, 6.9 Hz, 1.3 Hz, 1H, 3-H), 5.71-5.78 (m, 1H, 4-H), 7.34 (d, J = 8.2 Hz, 2H, m-H), 7.70 (s, 1H, 2'-H), 7.92 (d, J = 8.2 Hz, 2H, o-H) ppm.

^{13}C NMR (100 MHz, $CDCl_3$): δ = 17.6 (C-5), 21.7 (p-CH_3), 33.6 (C-1), 71.2 (C-2), 128.2 (C-o), 128.4 (C-4), 129.9 (C-m), 132.2 (C-3), 136.9 (C-i), 137.1 (C-5'), 145.0 (C-p), 149.9 (C-4'), 153.5 (C-2') ppm.

FT-IR (ATR): $\tilde{\nu}$ = 3470 (w), 2920 (w), 2168 (w), 2038 (s), 1595 (m), 1516 (w), 1450 (w), 1325 (s), 1242 (w), 1148 (vs), 1085 (m), 1044 (m), 967 (m), 815 (w), 706 (m), 663 (s), 601 (s), 539 (m) cm^{-1}.

MS (ESI): m / z for $C_{15}H_{17}NO_4S$ calc.: 330.1 $[M+Na]^+$, found: 330.1.

HRMS (ESI): m / z for $C_{15}H_{17}NO_4S$ calc.: 330.0770 [M+Na], found: 330.0762.

(*E*)-1-(Oxazol-5-yl)pent-3-en-2-ol (S5)

According to GP1, (*E*)-1-(4-Tosyloxazol-5-yl)pent-3-en-2-ol Ts-**S5** (308 mg, 1.00 mmol), Na_2HPO_4 (566 mg, 4.00 mmol) and sodium amalgam (2 x 1.15 g, 5.00 mmol, 10 %), dissolved in THF (5 mL) and EtOH (5 mL), were treated in an ultrasound bath. Column chromatography on silica (petroleum ether / EtOAc 1 : 1) yielded the desired product (*E*)-1-(Oxazol-5-yl)pent-3-en-2-ol **S5 (**130 mg mg, 0.85 mmol, 85 %, dr (*syn*/*anti*) = 50 : 50) as colourless oil.

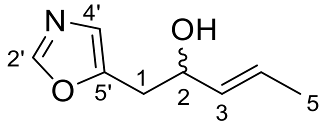

$C_8H_{11}NO_2S$

153.18

S5

R_f = 0.34 (petroleum ether / EtOAc = 1 : 1, molybdatophosphoric acid stain)

^{1}H NMR (400 MHz, $CDCl_3$): δ = 1.70 (dd, J = 6.5 Hz, 0.9 Hz, 3H, 5-H), 2.90 (dd, J = 6.5 Hz, 0.8 Hz, 2H, 1-H), 4.38 (dt, J = 6.5, 6.5 Hz, 1H, 2-H), 5.50-5.57 (m, 1H, 3-H), 5.68-5.78 (m, 1H, 4-H), 6.88 (s, 1H, 4'-H), 7.80 (s, 1H, 2'-H) ppm.

^{13}C NMR (100 MHz, $CDCl_3$): δ = 17.6 (C-5), 33.6 (C-1), 71.0 (C-2), 123.8 (C-4'), 128.2 (C-4), 132.4 (C-3), 149.5 (C-5'), 150.4 (C-2') ppm.

FT-IR (ATR): $\tilde{\nu}$ = 3357 (s), 3130 (m), 2918 (s), 2193 (w), 2072 (w), 2031 (w), 1729 (m), 1674 (m), 1604 (w), 1511 (vs), 1450 (m), 1379 (m), 1317 (m), 1117 (vs), 1087 (s), 1037 (s), 996 (vs), 825 (m), 756 (w), 702 (m), 648 (vs) cm^{-1}.

MS (ESI): *m / z* for $C_8H_{11}NO_2$ calc.: 176.1 [M+Na]$^+$, found: 176.1.

HRMS (ESI): *m / z* for $C_8H_{11}NO_2$ calc.: 176.0682 [M+Na], found: 176.0682.

(*E*)-5-(2-((*tert*-Butyldimethylsilyl)oxy)pent-3-en-1-yl)-4-tosyloxazole (Ts-S6)

Under an inert atmosphere (N_2), (*E*)-1-(4-tosyloxazole-5-yl)pent-3-en-2-ol Ts-**S5** (800 mg, 2.60 mmol) was dissolved in dry DMF (10 mL) at 0 °C, NEt_3 (0.38 mL, 0.52 g, 5.20 mmol) was added dropwise and after stirring for 10 min *tert*-butyldimethylsilyl trifluoromethanesulfonate (0.77 mL, 0.89 g, 3.38 mmol) was added and stirred for 18 h at rt. The reaction mixture was added to sat. $NaHCO_3$-solution (10 mL), extracted with dichloromethane (4 x 10 mL) and the combined organic layers were dried with $MgSO_4$. Column chromatography on silica (petroleum ether / EtOAc 3 : 1) yielded (*E*)-5-(2-((tert-Butyldimethylsilyl)oxy)pent-3-en-1-yl)-4-tosyloxazole Ts-**S6** (760 mg, 1.80 mmol, 70 %, dr (*syn*/*anti*) = 50 : 50) as colourless oil.

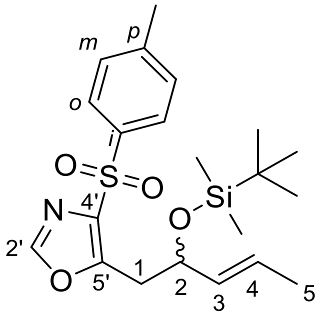

Ts-**S6**

$C_{21}H_{31}NO_4SSi$

421.63

R_f = 0.41 (petroleum ether / EtOAc = 5 : 1, molybdatophosphoric acid stain)

^{1}H NMR (500 MHz, $CDCl_3$): δ = -0.13 (s, 3H, Si-(C*H*$_3$)$_a$), -0.03 (s, 3H, Si-(C*H*$_3$)$_b$), 0.78 (s, 9H, C(C*H*$_3$)$_3$), 1.65 (d, 6.3 Hz, 3H, 5-H), 2.43 (s, 3H, *p*-CH_3), 3.24-3.27 (m, 2H, 1-H), 4.46-4.52 (m, 1H, 2-H), 5.45-5.51 (m, 1H, 3-H), 5.56-5.64 (m, 1H, 4-H), 7.33 (d, *J* = 8.0 Hz, 2H, *m*-H), 7.72 (s, 1H, 2'-H), 7.92 (d, *J* = 8.0 Hz, 2H, *o*-H) ppm.

^{13}C NMR (125 MHz, $CDCl_3$): δ = -5.2 (Si-(C*H*$_3$)$_a$), -4.4 (Si-(C*H*$_3$)$_b$), 17.5 (C-5), 17.9 (*C*(CH_3)$_3$), 21.7 (*p*-CH_3), 25.6 (C(*C*H$_3$)$_3$), 34.8 (C-1), 72.4 (C-2), 126.9 (C-4), 128.1 (C-*o*), 129.8 (C-*m*), 133.1 (C-3), 136.4 (C-5'), 137.4 (C-*i*), 144.7 (C-*p*), 149.5 (C-2'), 154.7 (C-4') ppm.

FT-IR (ATR): $\tilde{\nu}$ = 3134 (w), 2956 (m), 2930 (m), 2891 (w), 2857 (m), 2064 (w), 2025 (w), 1596 (m), 1516 (w), 1472 (w), 1329 (s), 1305 (w), 1253 (m), 1149 (vs), 1086 (s), 967 (m), 934 (m), 837 (s), 813 (m), 778 (m), 706 (m), 661 (s), 600 (s), 540 (m) cm^{-1}.

MS (ESI): *m / z* for $C_{21}H_{31}NO_4SSi$ calc.: 444.16 [M+Na]$^+$, found: 444.16.

HRMS (ESI): *m / z* for $C_{21}H_{31}NO_4SSi$ calc.: 444.1635 [M+Na]$^+$, found: 444.1635.

(*E*)-5-(2-((tert-Butyldimethylsilyl)oxy)pent-3-en-1-yl)oxazol (S6)

Under an inert atmosphere (N_2), (*E*)-5-(2-((tert-Butyldimethylsilyl)oxy)pent-3-en-1-yl)-4-tosyloxazol Ts-**S6** (570 mg, 1.35 mmol) dissolved in methanol (20 mL) and magnesium turnings (237 mg, 9.75 mmol) were added at rt and stirred for 1 h. The reaction mixture was added to HCl-solution (10 mL, 3 M), extracted with dichloromethane (3 x 10 mL) and the combined organic layers were dried over $MgSO_4$. Column chromatography on silica (petroleum ether / EtOAc 3 : 1) yielded (*E*)-5-(2-((tertButyldimethylsilyl)oxy)pent-3-en-1-yl)oxazol **S6** (125 mg, 0.47 mmol, 35 %, dr (*syn*/*anti*) = 50 : 50) as colourless oil.

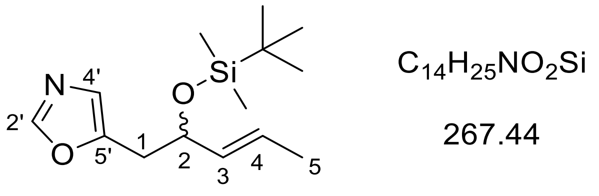

S6

R_f = 0.50 (petroleum ether / EtOAc = 5 : 1, molybdatophosphoric acid stain)

^{1}H NMR (500 MHz, $CDCl_3$): δ = -0.09 (s, 3H, Si-(CH_3)$_a$), -0.04 (s, 3H, Si-(CH_3)$_b$), 0.83 (s, 9H, C(CH_3)$_3$), 1.66 (d, 6.5 Hz, 3H, 5-H), 2.76-2.86 (m, 2H, 1-H), 4.30-4.35 (m, 1H, 2-H), 5.44 (dqd, *J* = 15.2 Hz, 6.5 Hz, 1.5 Hz, 1H, 3-H), 5.55-5.63 (m, 1H, 4-H), 6.81 (s, 1H, 4'-H), 7.77 (s, 1H, 2'-H) ppm.

^{13}C NMR (125 MHz, $CDCl_3$): δ = -5.2 (Si-(CH_3)$_a$), -4.5 (Si-(CH_3)$_b$), 17.5 (C-5), 18.0 (*C*(CH_3)$_3$), 25.7 (C(*C*H_3)$_3$), 34.9 (C-1), 71.8 (C-2), 123.7 (C-4'), 126.3 (C-4), 133.4 (C-3), 150.0 (C-5'), 150.1 (C-2') ppm.

FT-IR (ATR): $\tilde{\nu}$ = 3365 (w), 2954 (m), 2929 (m), 2885 (w), 2856 (m), 2184 (w), 2103 (w), 1674 (w), 1603 (w), 1510 (m), 1472 (m), 1463 (w), 1389 (w), 1361 (w), 1253 (s), 1212 (w), 1121 (m), 1093 (s), 1070 (s), 1006 (m), 966 (m), 935 (m), 884 (m), 832 (vs), 774 (vs), 665 (m), 647 (m), 574 (w), 536 (w), 444 (w) cm^{-1}.

MS (EI): *m/z* (%) = 267.2 (3), 252.1 (5), 226.1 (1), 210.1 (100), 185.1 (87), 168.0 (3), 140.0 (3), 128.1 (6), 113.0 (6), 99.1 (3), 73.0 (62), 59.0 (5), 45.0 (3), 29.0 (1).

HRMS (ESI): *m / z* for $C_{14}H_{25}NO_2Si$ calc.: 290.1547 $[M+Na]^+$, found: 290.1541.

(*S*,*E*)-1-(4-tosyloxazol-5-yl)pent-3-en-2-yl (2E,4E)-2-methylhexa-2,4-dienoate (Ts-S7)

Under an inert atmosphere (N_2), (2*E*,4*E*)-2-methylhexa-2,4-dienoic acid **9** (126 mg, 1.00 mmol) was dissolved in dry THF (10 mL) and NEt_3 (0.14 mL, 101 mg, 1.00 mmol) was added dropwise. After 10 min, 2,4,6-trichlorobenzoyl chloride (0.16 mL, 244 mg, 1.00 mmol) was added dropwise and the reaction mixture was stirred for additional 6 h, filtered over celite, washed with dry THF (10 mL) and concentrated under reduced pressure. The formed anhydride was dissolved in dry DMF (10 mL) and (*E*)-1-(4-tosyloxazol-5-yl)pent-3-en-2-ol Ts-**S6** (307 mg, 1.00 mmol), dissolved in DMF (10 mL), and 4-Dimethylaminopyridine (245 mg, 2.00 mmol) were added and stirred for 1 h at 50 °C. The solvent was removed under reduced pressure and the crude product was dissolved in dichloromethane (10 mL), washed with HCl-solution (10 mL, 1 M), sat. $NaHCO_3$-solution (10 mL) and water (10 mL), and dried over

$MgSO_4$. Column chromatography on silica (petroleum ether / EtOAc 5 : 1) yielded the desired product (*S*,*E*)-1-(4-tosyloxazol-5-yl)pent-3-en-2-yl (2*E*,4*E*)-2-methylhexa-2,4-dienoate Ts-**S7** **(**166 mg, 0.40 mmol, 39 %) as colourless oil. Starting material (*E*)-1-(4-tosyloxazol-5-yl)pent-3-en-2-ol Ts-**S5** **(**85 mg, 0.28 mmol, 28 %, dr (*syn*/*anti*) = 62 : 38) was reisolated.

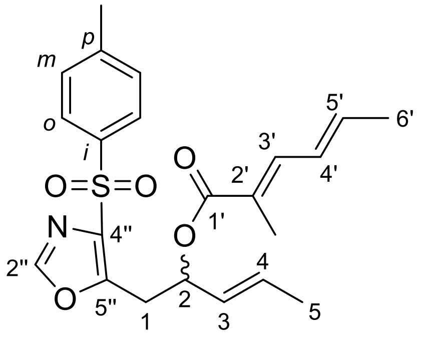

$C_{22}H_{25}NO_5S$

415.50

Ts-**S7**

R_f = 0.36 (petroleum ether / EtOAc = 3 : 1, molybdatophosphoric acid stain).

^{1}H NMR (400 MHz, $CDCl_3$): δ = 1.66-1.69 (m, 3H, 5-H), 1.87 (s, 3H, 6'-H), 1.88 (s, 3H, 2'-CH_3), 2.42 (s, 3H, *p*-CH_3), 3.45-3.60 (m, 2H, 1-H), 5.50-5.58 (m, 1H, 4-H), 5.61-5.68 (m, 1H, 2-H), 5.73-5.83 (m, 1H, 3-H), 6.05-6.15 (m, 1H, 5'-H), 6.28-6.39 (m, 1H, 4'-H), 7.15 (d, J = 10.9 Hz, 1H, 3'-H), 7.32 (d, J = 8.0 Hz, 2H, *m*-H), 7.73 (s, 1H, 2"-H), 7.91 (d, J = 8.0 Hz, 1H, *o*-H) ppm.

^{13}C NMR (100 MHz, $CDCl_3$): δ = 12.4 (2'-CH_3), 17.7 (C-5), 18.9 (C-6'), 21.7 (*p*-CH_3), 31.0 (C-1), 72.0 (C-2), 124.4 (C-2'), 127.4 (C-4'), 128.0 (C-4), 128.2 (C-*o*), 129.86 (C-*m*), 129.93 (C-3), 130.6 (C-5"), 137.1 (C-*i*), 138.3 (C-5'), 139.2 (C-3'), 144.9 (C-*p*), 150.0 (C-2"), 152.8 (C-4"), 167.5 (C-1') ppm.

FT-IR (ATR): $\tilde{\nu}$ = 3137 (w), 3034 (w), 2923 (w), 2856 (w), 2256 (w), 1701 (s), 1641 (m), 1596 (m), 1516 (w), 1446 (w), 1329 (m), 1305 (m), 1290 (m), 1229 (s), 1148 (vs), 1089 (s), 1048 (m), 1048 (m), 1018 (m), 968 (m), 910 (s), 814 (m), 730 (vs), 706 (m), 661 (s), 600 (vs), 538 (s) cm^{-1}.

MS (ESI): m/z for $C_{22}H_{25}NO_5S$ calc.: 415.1 $[M+Na]^+$, found: 415.1.

HRMS (ESI): m/z for $C_{22}H_{25}NO_5S$ calc.: 415.1453 $[M+Na]^+$, found: 415.1449.

(2*S*,3*R*,10*R*,11*S*,*E*)-3,10-dimethyl-1,12-bis(4-tosyloxazol-5-yl)dodec-6-ene-2,11-diol (*anti*-S4)

According to GP2, (2*S*,3*R*)-3-Methyl-1-(4-tosyloxazol-5-yl)hept-6-en-2-ol *anti*-Ts-**10** (400 mg, 1.15 mmol) was treated with (2*E*,4*E)*-2-methy2-2,4-hexadienoate **15** (434 mg, 1.15 mmol) and Grubbs-II catalyst (14.6 mg, 17.3µmol) in dichloromethane (30 mL). Column chromatography on silica (petroleum ether / EtOAc 1 : 1) yielded (2*S*,3*R*,10*R*,11*S*,*E*)-3,10-dimethyl-1,12-bis(4-tosyloxazol-5-yl)dodec-6-ene-2,11-diol *anti*-**S4** **(**118 mg, 0.27 mmol, 47 %, dr (*syn*/*anti)*= 0 : 100 (according to ^{1}H NMR)) as colourless solid.

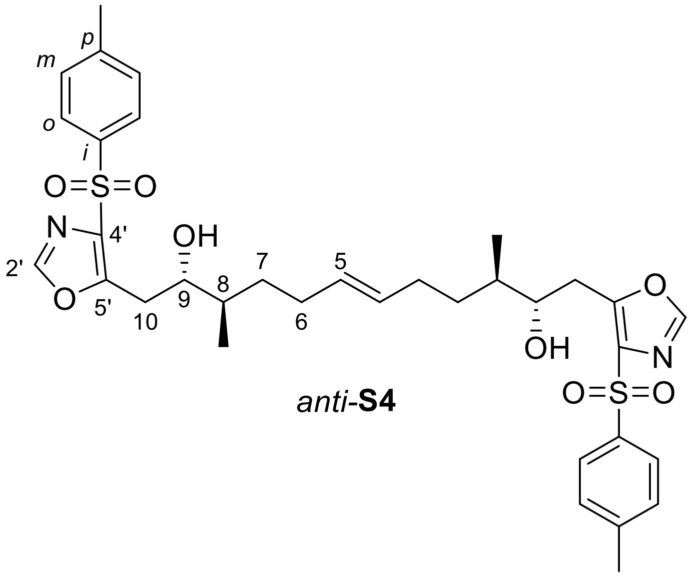

$C_{34}H_{42}N_2O_8S_2$

670.84

R_f = 0.24 (petroleum ether / EtOAc = 1 : 1, molybdatophosphoric acid stain).

$[\alpha]_D^{20}$ = - 43.8° (c = 1.0, $CHCl_3$)

^1H NMR (500 MHz, $CDCl_3$): δ = 1.01 (d, J = 6.7 Hz, 3H, 3-CH_3), 1.23-1.32 (m, 1H, 4-H_a), 1.60-1.73 (m, 2H, 3-H, 4-H_b), 1.96-2.25 (m, 2H, 5-H), 2.43 (s, 3H, *p*-CH_3), 3.21-3.27 (m, 2H, 1-H), 3.81-3.87 (m, 1H, 2-H), 5.38-5.47 (m, 1H, 6-H), 7.33 (d, J = 8.2 Hz, 2H, *m*-H), 7.75 (s, 1H, 2'-H), 7.91 (d, J = 8.2 Hz, 2H, *o*-H) ppm.

^{13}C NMR (125 MHz, $CDCl_3$): δ = 14.7 (3-CH_3), 21.7 (*p*-CH_3), 29.9 (C-5), 30.0 (C-1), 31.8 (C-4), 38.4 (C-3), 74.4 (C-2), 128.1 (C-*o*), 129.9 (C-*m*), 130.4 (C-6), 136.6 (C-*i*), 136.9 (C-5'), 145.1 (C-p), 149.8 (C-2'), 155.0 (C-4') ppm.

FT-IR (ATR): $\tilde{\nu}$ = 3524 (w), 3134 (w), 2923 (w), 2253 (w), 1682 (w), 1637 (w), 1595 (m), 1517 (m), 1495 (w), 1456 (w), 1402 (w), 1381 (w), 1321 (s), 1305 (m), 1291 (m), 1244 (m), 1218 (w), 1145 (vs), 1085 (m), 1060 (m), 1017 (w), 975 (m), 911 (m), 853 (w), 814 (m), 731 (s), 705 (m), 697 (m), 661 (vs), 600 (vs), 539 (s) cm^{-1}.

MS (ESI): *m / z* for $C_{34}H_{42}N_2O_8S_2$ calc.: 693.2 $[M+Na]^+$, found: 693.2.

HRMS (ESI): *m / z* for $C_{34}H_{42}N_2O_8S_2$ calc.: 693.2275 $[M+Na]^+$, found: 693.2265.

13.8 Synthesis of Mosher esters

(2*S*,3*R*)-3-Methyl-1-(4-tosyloxazol-5-yl)hept-6-en-2-yl-(*R*)-3,3,3-trifluoro-2methoxy-2-phenylpropanoat ((*R*)-S1)

Under an inert atmosphere (N_2), (*R*)-(+)-α-Methoxy-α-trifluoromethylphenylacetic acid (*R*)-**S2** (40.0 mg, 0.17 mmol) was dissolved in dry THF (10 mL) at rt, oxalylchloride (73.0 µL, 108 mg, 0.85 mmol) and DMF (13.0 µL, 12.5 mg, 0.17 mmol) were added dropwise and the reaction mixture was stirred for 4 h. Evaporation of the solvent under reduced pressure gave the respective (*S*)-Mosheracid chloride (*R*)-**S3**, which was subsequently dissolved in dry dichloromethane (5 mL) at rt and under an inert atmosphere and dimethylaminopyridine

(20.9 mg, 0.17 mmol) was added. In a separate flask, (2*S*,3*R*)-3-Methyl-1-(4-tosyloxazol-5-yl)hept-6-en-2-ol *anti*-Ts-**10** (44.0 mg, 0.13 mmol), dissolved in dry dichloromethane (3 mL), was treated diazabicycloundecene (20.8µL, 21.3 mg, 0.14 mmol), stirred for 10 min at rt and then added dropwise to the (*S*)-Mosheracid chloride reaction mixture. After stirring for 24 h, the reaction mixture was added to water (10 mL), extracted with dichloromethane (3 x 20 mL) and the combined organic phases were dried over $MgSO_4$. Column chromatography (petroleum ether / EtOAc 3 : 1) yielded the desired product (2*S*,3*R*)-3-Methyl-1-(4-tosyloxazol-5-yl)hept-6-en-2-yl-(*R*)-3,3,3-trifluoro2-methoxy-2-phenylpropanoat (*R*)-**S1** (27.0 mg, 4.77 µmol, 38 %, dr = 100 : 0 (according to ^{1}H NMR)) as colourless oil.

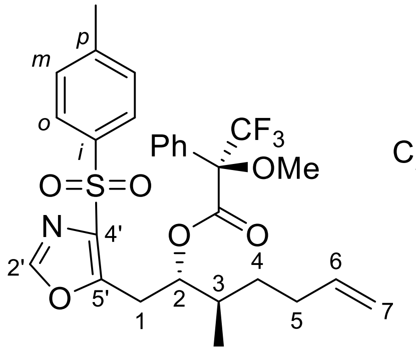

$C_{28}H_{30}F_3NO_6S$

565.60

(*R*)-**S1**

R_f = 0.45 (petroleum ether / EtOAc = 3 : 1, molybdatophosphoric acid stain).

^{1}H NMR (500 MHz, $CDCl_3$): δ = 0.93 (d, J = 7.0 Hz, 3H, 3-Me), 1.26-1.34 (m, 1H, 4-H_a), 1.56-1.63 (m, 1H, 4-H_b), 1.92-1.99 (m, 1H, 3-H), 2.03-2.24 (m, 2H, 5-H), 2.40 (s, 3H, *p*-CH_3), 3.37 (s, 3H, OCH_3), 3.44 (d, J = 6.6 Hz, 2H, 1-H), 4.98 (d, J = 10.3 Hz, 1H, 7-H_a), 5.04 (dd, J = 17.0 Hz, 1.5 Hz, 1H, 7-H_b), 5.48-5.53 (m, 1H, 2-H), 5.77 (ddt, J = 17.0 Hz, 10.3 Hz, 6.8 Hz, 1H, 6-H), 7.22-7.29 (m, 5H, Ph-H), 7.34 (d, J = 8.2 Hz, 2H, *m*-H), 7.61 (s, 1H, 2’-H), 7.90 (d, J = 8.2 Hz, 2H, *o*-H) ppm.

^{13}C NMR (125 MHz, $CDCl_3$): δ = 14.1 (3-Me), 21.7 (*p*-CH_3), 26.2 (C-1), 31.1 (C-5), 31.2 (C-4), 35.7 (C-3), 55.2 (O*C*H_3), 77.8 (C-2), 84.5 (q, J = 27.8 Hz, *C*OCH_3), 115.2 (C-7), 123.2 (q, J = 288.0 Hz, CF_3), 127.0 (CPh*p*), 128.2 (C-*o*), 128.4 (C-Ph*o*), 129.5 (C-Ph*m*), 129.9 (C-*m*), 131.8 (C-Ph*i*), 136.7 (C-*i*), 137.0 (C-5’), 137.9 (C-6), 145.1 (C-p), 149.9 (C-2’), 152.8 (C-4’), 165.9 (COO) ppm.

FT-IR (ATR): $\tilde{\nu}$ = 3131 (w), 3066 (w), 2927 (w), 1746 (s), 1641 (w), 1596 (m), 1515 (w), 1495 (w), 1452 (w), 1329 (m), 1291 (m), 1249 (s), 1169 (s), 1149 (vs), 1121 (m), 1084 (m), 1018 (m), 996 (m), 917 (w), 815 (w), 766 (w), 720 (m), 697 (m), 663 (m), 601 (s), 539 (m) cm^{-1}.

MS (ESI): *m / z* for $C_{28}H_{30}F_3NO_4S$ calc.: 588.2 $[M+Na]^+$, found: 588.2.

HRMS (ESI): *m / z* for $C_{28}H_{30}F_3NO_4S$ calc.: 588.1638 $[M+Na]^+$, found: 588.1623.

(2*S*,3*R*)-3-Methyl-1-(4-tosyloxazol-5-yl)hept-6-en-2-yl-(*S*)-3,3,3-trifluoro-2methoxy-2-phenylpropanoat ((*S*)-S1)

Under an inert atmosphere (N_2), (*S*)-(+)-*α*-Methoxy-*α*-trifluoromethylphenylacetic acid (*S*)-**S2** (40.0 mg, 0.17 mmol) was dissolved in dry THF (10 mL) at rt, oxalylchloride (73.0 µL, 108 mg, 0.85 mmol) and DMF (13.0 µL, 12.5 mg, 0.17 mmol) were added dropwise and the reaction mixture was stirred for 4 h. Evaporation of the solvent under reduced pressure gave the respective (*R*)-Mosheracid chloride (*S*)-**S3**, which was subsequently dissolved in dry dichloromethane (5 mL) at rt and under an inert atmosphere and dimethylaminopyridine (20.9 mg, 0.17 mmol) was added. In a separate flask, (2*S*,3*R*)-3-Methyl-1-(4-tosyloxazol-5-yl)hept-6-en-2-ol *anti*-Ts-**10** (44.0 mg, 0.13 mmol), dissolved in dry dichloromethane (3 mL), was treated diazabicycloundecene (20.8µL, 21.3 mg, 0.14 mmol), stirred for 10 min at rt and then added dropwise to the (*S*)-Mosheracid chloride reaction mixture. After stirring for 24 h, the reaction mixture was added to water (10 mL), extracted with dichloromethane (3 x 20 mL) and the combined organic phases were dried over $MgSO_4$. Column chromatography (petroleum ether / EtOAc 3 : 1) yielded the desired product (2*S*,3*R*)-3-Methyl-1-(4-tosyloxazol-5-yl)hept-6-en-2-yl-(*S*)-3,3,3-trifluoro2-methoxy-2-phenylpropanoat (*S*)-**S1** (65.0 mg, 11.5µmol, 90 %, dr = 0 : 100 (according to ^{1}H NMR)) as colourless oil.

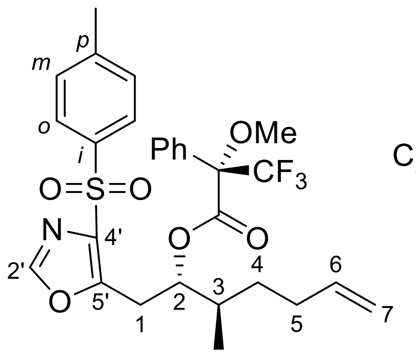

$C_{28}H_{30}F_3NO_6S$

565.60

(*S*)-**S1**

R_f = 0.45 (petroleum ether / EtOAc = 3 : 1, molybdatophosphoric acid stain).

^{1}H NMR (500 MHz, $CDCl_3$): δ = 1.03 (d, *J* = 7.0 Hz, 3H, 3-Me), 1.29-1.37 (m, 1H, 4-H_a), 1.59-1.68 (m, 1H, 4-H_b), 1.96-2.03 (m, 1H, 3-H), 2.07-2.27 (m, 2H, 5-H), 2.43 (s, 3H, *p*-CH_3), 3.29 (dd, *J* = 15.3 Hz, 10.2 Hz, 1H, 1-H_a), 3.44 (dd, *J* = 15.3 Hz, 3.5 Hz, 1H, 1-H_b), 3.46 (s, 3H, OCH_3), 5.00 (d, *J* = 10.3 Hz, 1H, 7-H_a), 5.06 (dd, *J* = 17.0 Hz, 1.8 Hz, 1H, 7-H_b), 5.48 (dt, *J* = 10.0 Hz, 3.8 Hz, 1H, 2-H), 5.80 (tt, *J* = 10.2, 6.7 Hz, 1H, 6-H), 7.23-7.34 (m, 5H, Ph-H), 7.36 (d, *J* = 8.2 Hz, 2H, *m*-H), 7.37 (s, 1H, 2'-H), 7.91 (d, *J* = 8.2 Hz, 2H, *o*-H) ppm.

^{13}C NMR (125 MHz, $CDCl_3$): δ = 14.4 (3-Me), 21.7 (*p*-CH_3), 26.1 (C-1), 31.1 (C-5), 31.3 (C-4), 35.9 (C-3), 55.4 (O*C*H_3), 77.9 (C-2), 84.4 (q, *J* = 27.7 Hz, *C*OCH_3), 115.3 (C-7), 123.1 (q, *J* = 288.0 Hz, CF_3), 127.1 (CPh*p*), 128.2 (C-*o*), 128.5 (C-Ph*o*), 129.5 (C-Ph*m*), 129.9 (C-*m*), 131.9 (C-Ph*i*), 136.6 (C-*i*), 136.8 (C-5'), 137.9 (C-6), 145.1 (C-p), 149.9 (C-2'), 152.7 (C-4'), 165.9 (COO) ppm.

FT-IR (ATR): $\tilde{\nu}$ = 2928 (w), 1746 (s), 1641 (w), 1595 (w), 1516 (w), 1494 (w), 1452 (w), 1329 (m), 1254 (s), 1169 (s), 1149 (vs), 1122 (m), 1084 (m), 1018 (m), 997 (m), 917 (w), 815 (w), 766 (w), 719 (m), 697 (m), 663 (m), 601 (s), 539 (m) cm^{-1}.

MS (ESI): *m / z* for $C_{28}H_{30}F_3NO_4S$ calc.: 588.2 $[M+Na]^+$, found: 588.2.

HRMS (ESI): *m / z* for $C_{28}H_{30}F_3NO_4S$ calc.: 588.1638 $[M+Na]^+$, found: 588.1638.

14 Literature

[1] T. R. Hoye, C. S. Jeffrey, F. Shao, *Nat. Protoc.* **2007**, *2*, 2451–2458.

[2] M. J. Corr, R. A. Cormanich, C. N. von Hahmann, M. Bühl, D. B. Cordes, A. M. Z. Slawin, D. O'Hagan, *Org. Biomol. Chem.* **2016**, *14*, 211–219.

[3] T. Nagamitsu, D. Takano, K. Marumoto, T. Fukuda, K. Furuya, K. Otoguro, K. Takeda, I. Kuwajima, Y. Harigaya, S. Ōmura, *J. Org. Chem.* **2007**, *72*, 2744–2756.

[4] M. S. Addie, R. J. K. Taylor, *J. Chem. Soc. Perkin 1* **2000**, 527–531.

[5] J. Inanaga, K. Hirata, H. Saeki, T. Katsuki, M. Yamaguchi, *Bull. Chem. Soc. Jpn.* **1979**, *52*, 1989–1993.

[6] M. Scholl, S. Ding, C. W. Lee, R. H. Grubbs, *Org. Lett.* **1999**, *1*, 953–956.

[7] M. S. Sanford, J. A. Love, R. H. Grubbs, *J. Am. Chem. Soc.* **2001**, *123*, 6543–6554.

[8] A. V. R. Madduri, A. J. Minnaard, *Chem. – Eur. J.* **2010**, *16*, 11726–11731.

[9] L. Song, J. Liu, H. Gui, C. Hui, J. Zhou, Y. Guo, P. Zhang, Z. Xu, T. Ye, *Chem. – Asian J.* **2013**, *8*, 2955–2959.

[10] M. Kita, H. Watanabe, T. Ishitsuka, Y. Mogi, H. Kigoshi, *Tetrahedron Lett.* **2010**, *51*, 4882–4885.

[11] T. J. Hoffman, A. Kolleth, J. H. Rigby, S. Arseniyadis, J. Cossy, *Org. Lett.* **2010**, *12*, 3348–3351.

[12] T. J. Hoffman, J. H. Rigby, S. Arseniyadis, J. Cossy, *J. Org. Chem.* **2008**, *73*, 2400–2403.

[13] M. Kita, H. Oka, A. Usui, T. Ishitsuka, Y. Mogi, H. Watanabe, H. Kigoshi, *Tetrahedron* **2012**, *68*, 8753–8760.

[14] K. C. Nicolaou, H. Vallberg, N. P. King, F. Roschangar, Y. He, D. Vourloumis, C. G. Nicolaou, *Chem. – Eur. J.* **1997**, *3*, 1957–1970.

[15] *Saponification of Seco-Esters Syn-25b, Anti-25b Gave in Both Cases Pyridyl Ketone 33,* **n.d.**

[16] A. Dramae, S. Nithithanasilp, W. Choowong, P. Rachtawee, S. Prabpai, P. Kongsaeree, P. Pittayakhajonwut, *Tetrahedron* **2013**, *69*, 8205–8208.

[17] C. Snyder, J. Chollet, J. Santo-Tomas, C. Scheurer, S. Wittlin, *Exp. Parasitol.* **2007**, *115*, 296–300.

[18] W. Trager, J. B. Jensen, *Science* **1976**, *193*, 673–675.

[19] M. T. Makler, J. M. Ries, J. A. Williams, J. E. Bancroft, R. C. Piper, B. L. Gibbins, D. J. Hinrichs, *Am. J. Trop. Med. Hyg.* **1993**, *48*, 739–741.

[20] "Broth Dilution Method for MIC Determination • Microbe Online," can be found under https://microbeonline.com/minimum-inhibitory-concentration-mic-broth-dilution-method-procedure-interpretation/, **n.d.**

[21] A Sample of Samroiyotmycin A Was Isolated from the Natural Source by Pittayakhajonwut from BIOTEC, Thailand and Sent to Us for Our Biological Studies., **n.d.**

[22] S. Wittlin, E. Ekland, J. C. Craft, J. Lotharius, I. Bathurst, D. A. Fidock, P. Fernandes, *Antimicrob. Agents Chemother.* **2012**, *56*, 703–707.

[23] K. Ozvoldik, T. Stockner, E. Krieger, *J. Chem. Inf. Model.* **2023**, *63*, 6177–6182.

[24] E. L. Dodd, D. S. Bohle, *Chem. Commun.* **2014**, *50*, 13765–13768.

[25] S. Kapishnikov, E. Hempelmann, M. Elbaum, J. Als-Nielsen, L. Leiserowitz, *ChemMedChem* **2021**, *16*, 1515–1532.

[26] D. S. Bohle, E. L. Dodd, P. W. Stephens, *Chem. Biodivers.* **2012**, *9*, 1891–1902.

[27] M. Michalak, J. Wicha, *Org. Biomol. Chem.* **2011**, *9*, 3439–3446.

[28] M. J. Corr, R. A. Cormanich, C. N. von Hahmann, M. Bühl, D. B. Cordes, A. M. Z. Slawin, D. O'Hagan, *Org. Biomol. Chem.* **2015**, *14*, 211–219.

[29] R. K. Jr. Boeckman, A. B. Charette, T. Asberom, B. H. Johnston, *J. Am. Chem. Soc.* **1991**, *113*, 5337–5353.

[30] M. S. Addie, R. J. K. Taylor, *J. Chem. Soc. Perkin 1* **2000**, *0*, 527–531.

[31] G. H. Lonca, D. Y. Ong, T. M. H. Tran, C. Tejo, S. Chiba, F. Gagosz, *Angew. Chem. Int. Ed.* **2017**, *56*, 11440–11444.

[32] S. D. Bull, S. G. Davies, S. Jones, H. J. Sanganee, *J. Chem. Soc. Perkin 1* **1999**, *0*, 387–398.

[33] M. Ceroni, U. Séquin, *Helv. Chim. Acta* **1982**, *65*, 302–316.

15 NMR and MS spectra

^{1}H NMR (400 MHz, $CDCl_3$) of **14**

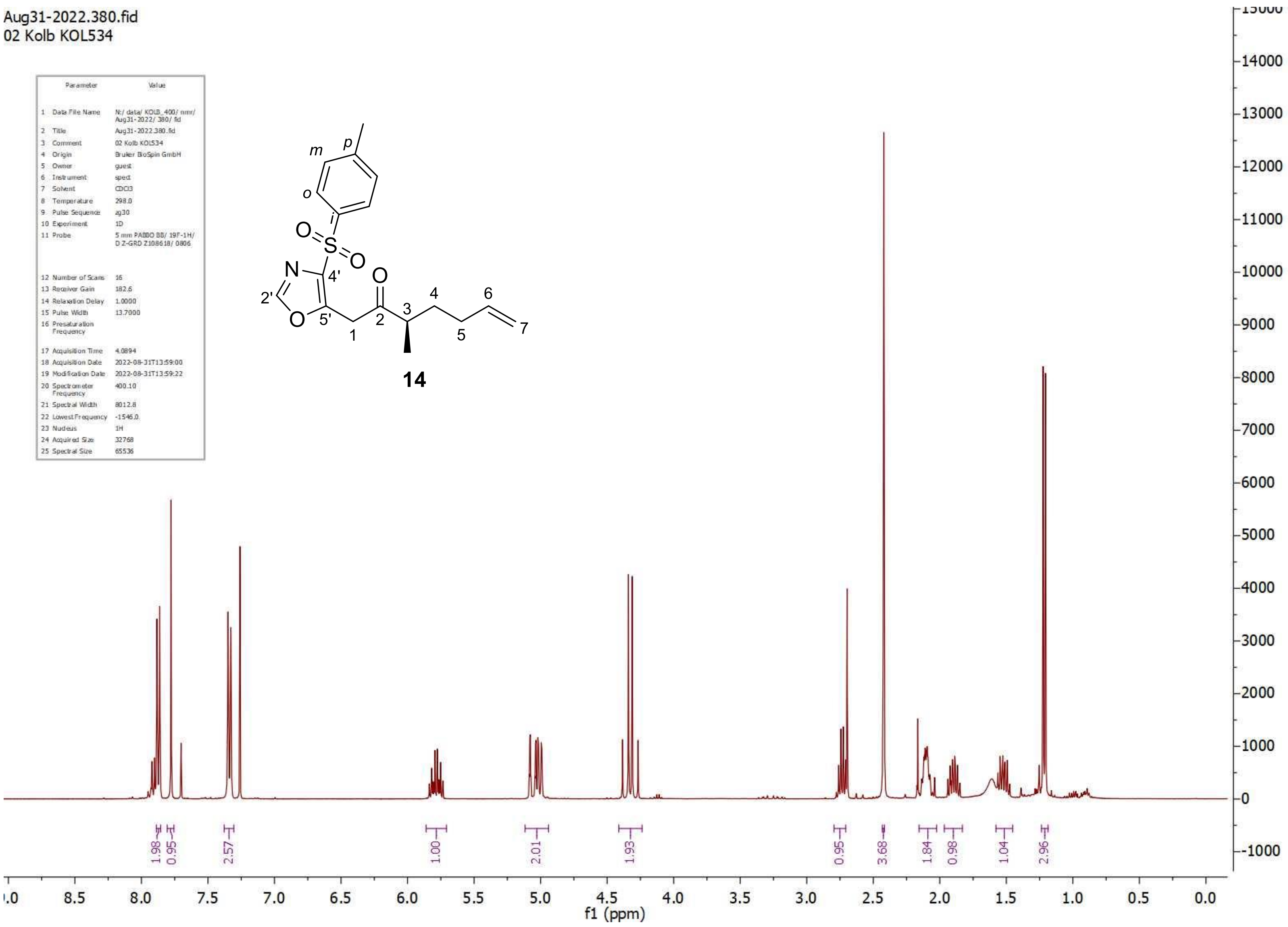

^{13}C NMR (100 MHz, $CDCl_3$) of **14**

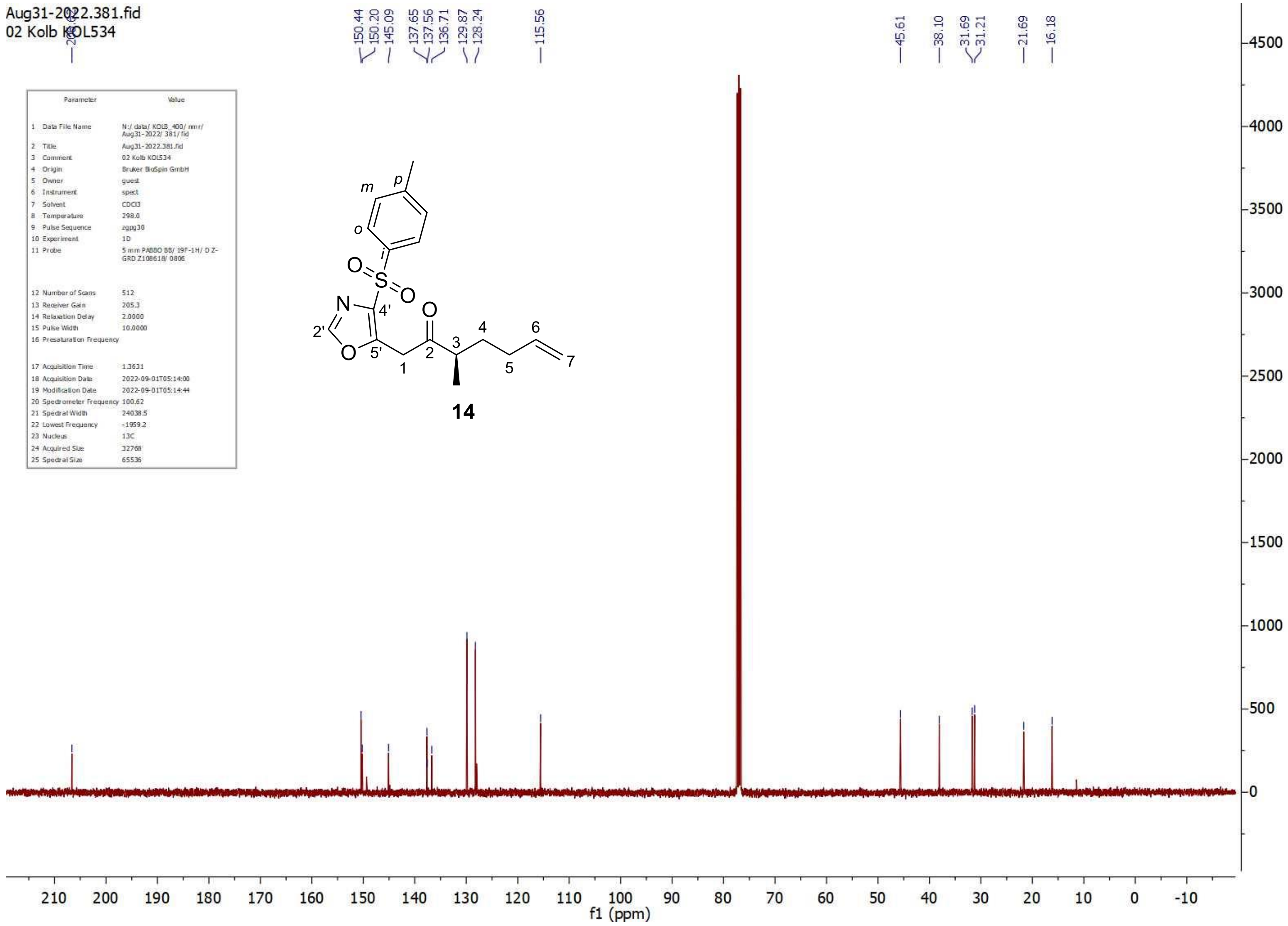

^{1}H NMR (400 MHz, $CDCl_3$) of *syn*-**Ts-10**

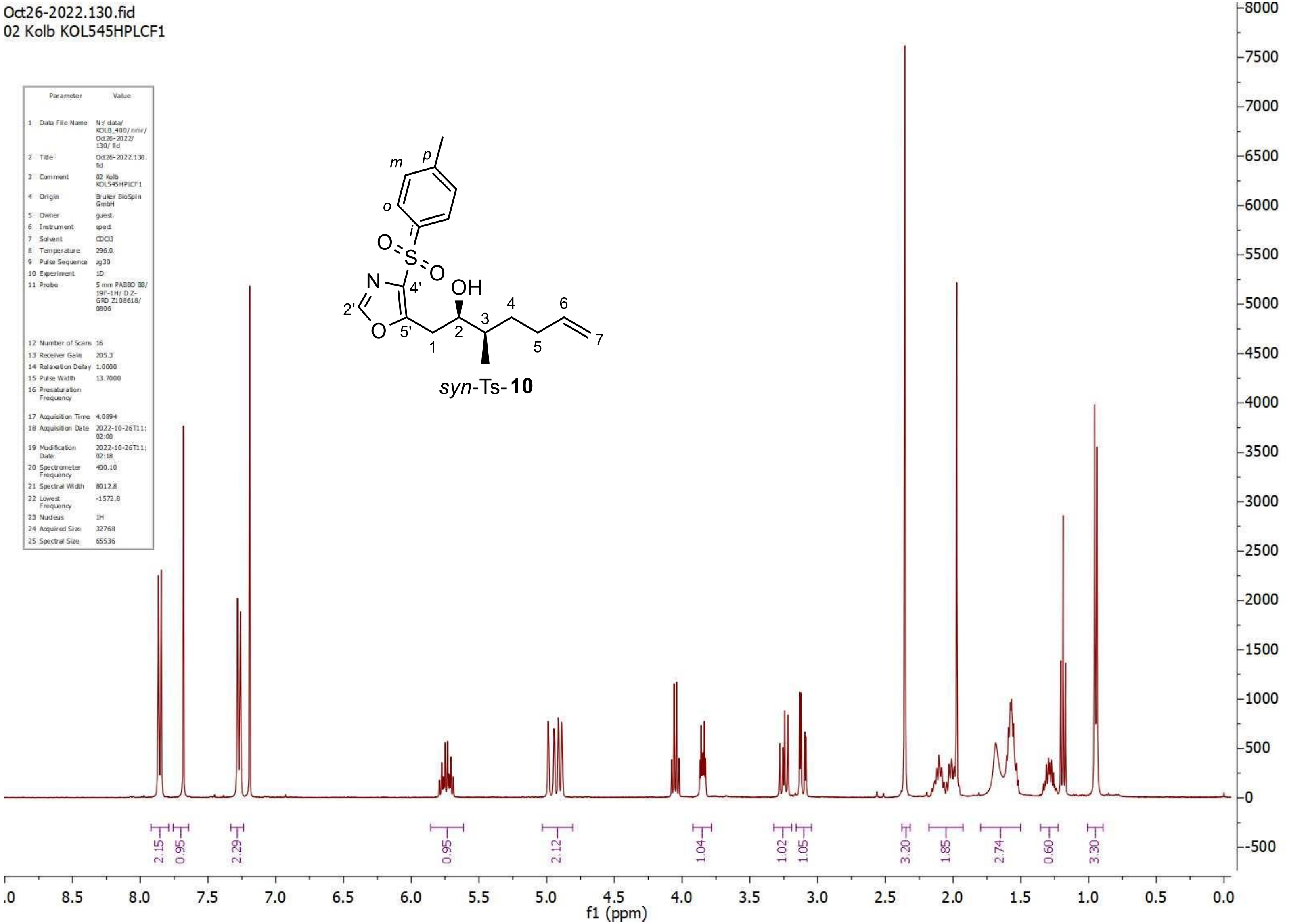

^{13}C NMR (100 MHz, $CDCl_3$) of *syn*-**Ts-10**

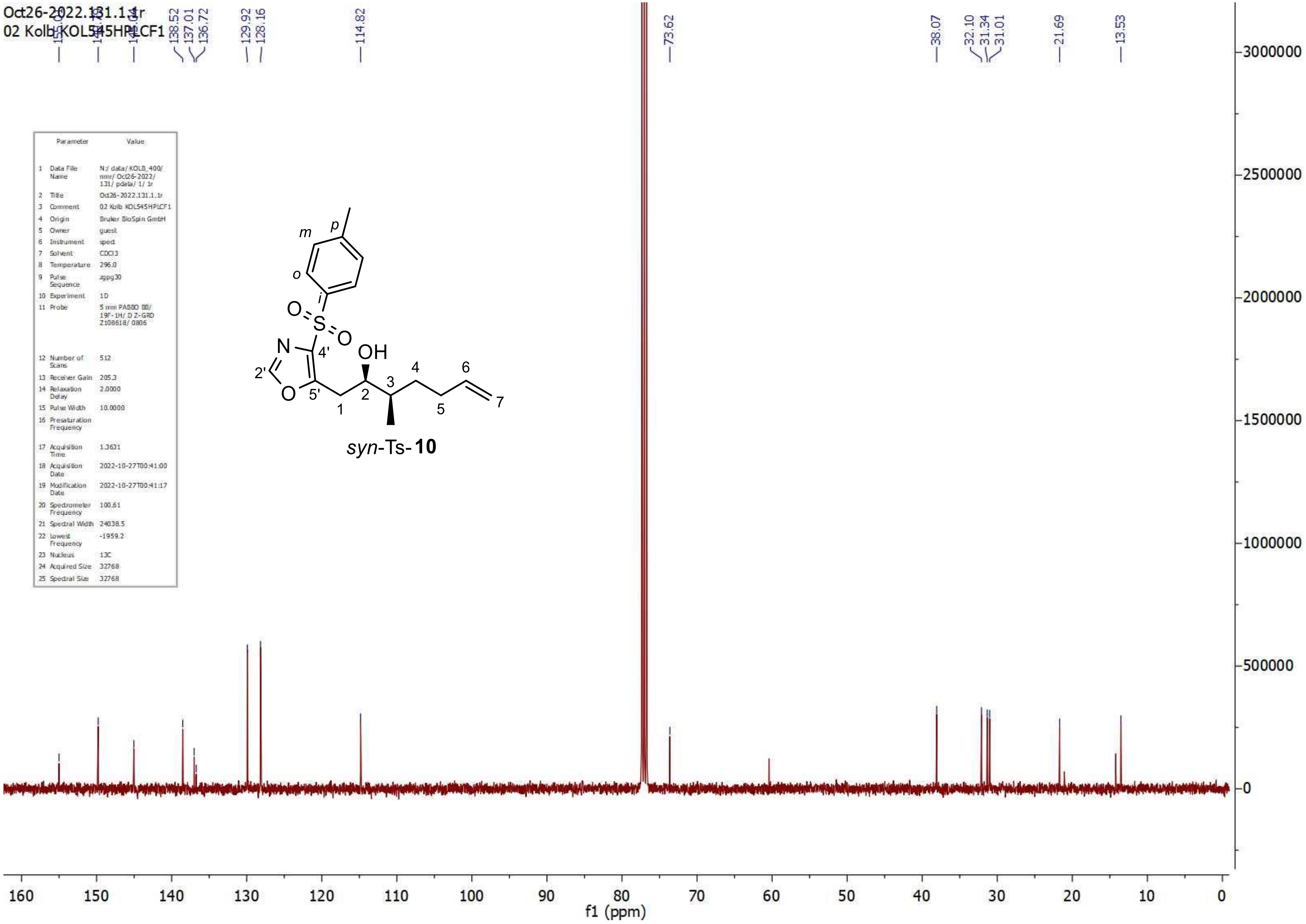

^{1}H NMR (400 MHz, $CDCl_3$) of *anti*-Ts-**10**

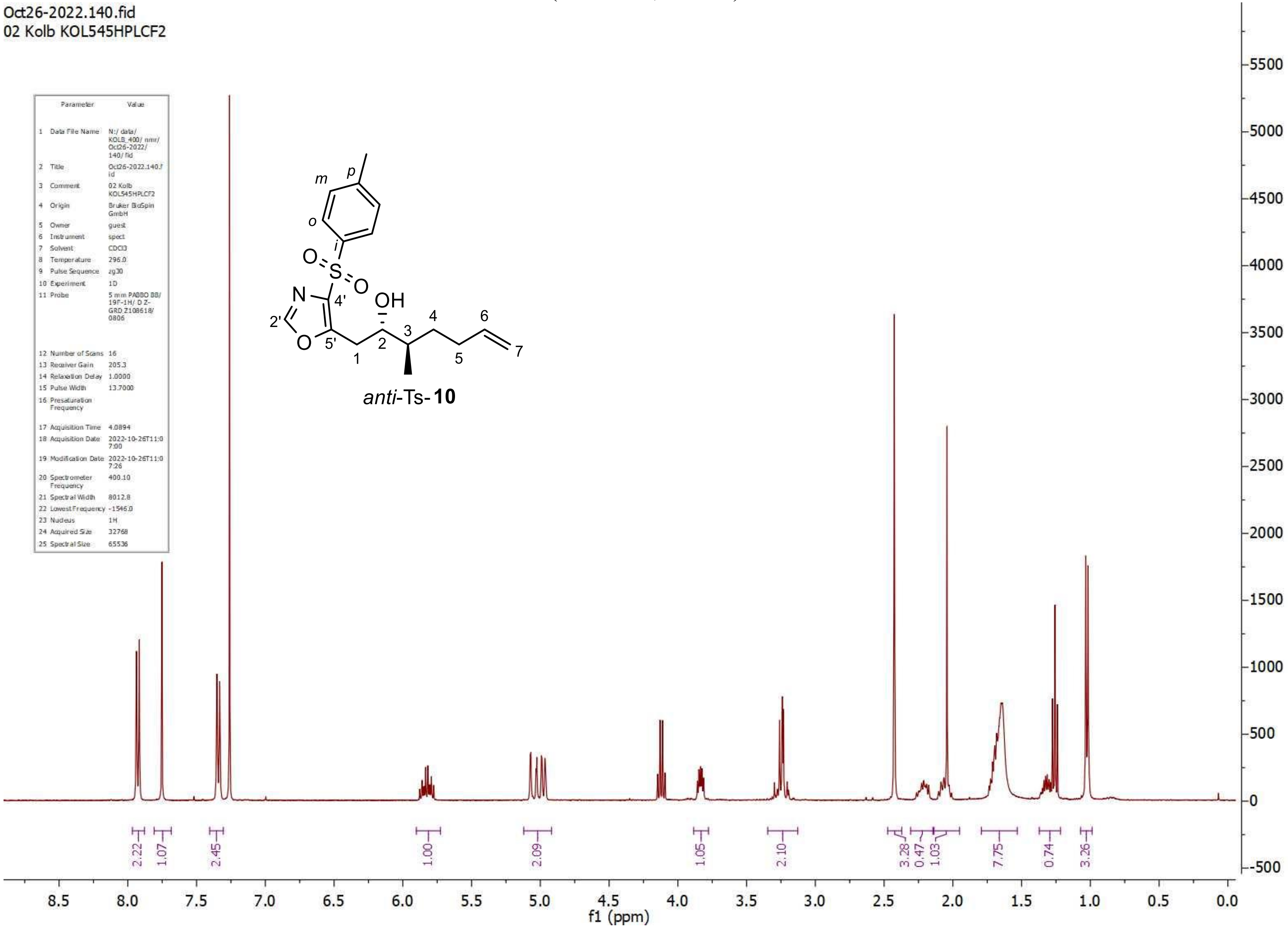

^{13}C NMR (100 MHz, $CDCl_3$) of *anti*-Ts-**10**

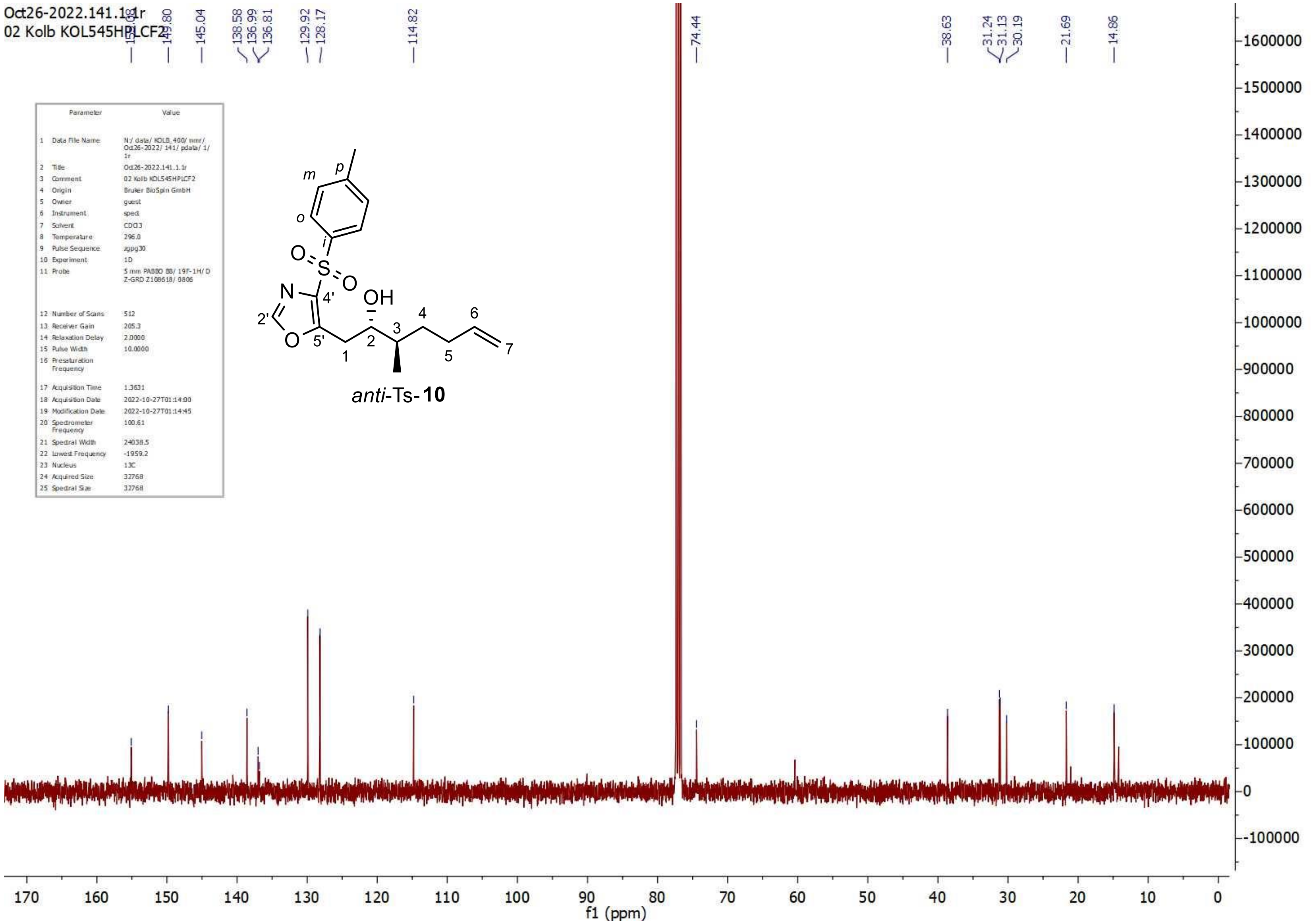

^{1}H NMR (400 MHz, $CDCl_3$) of **10**

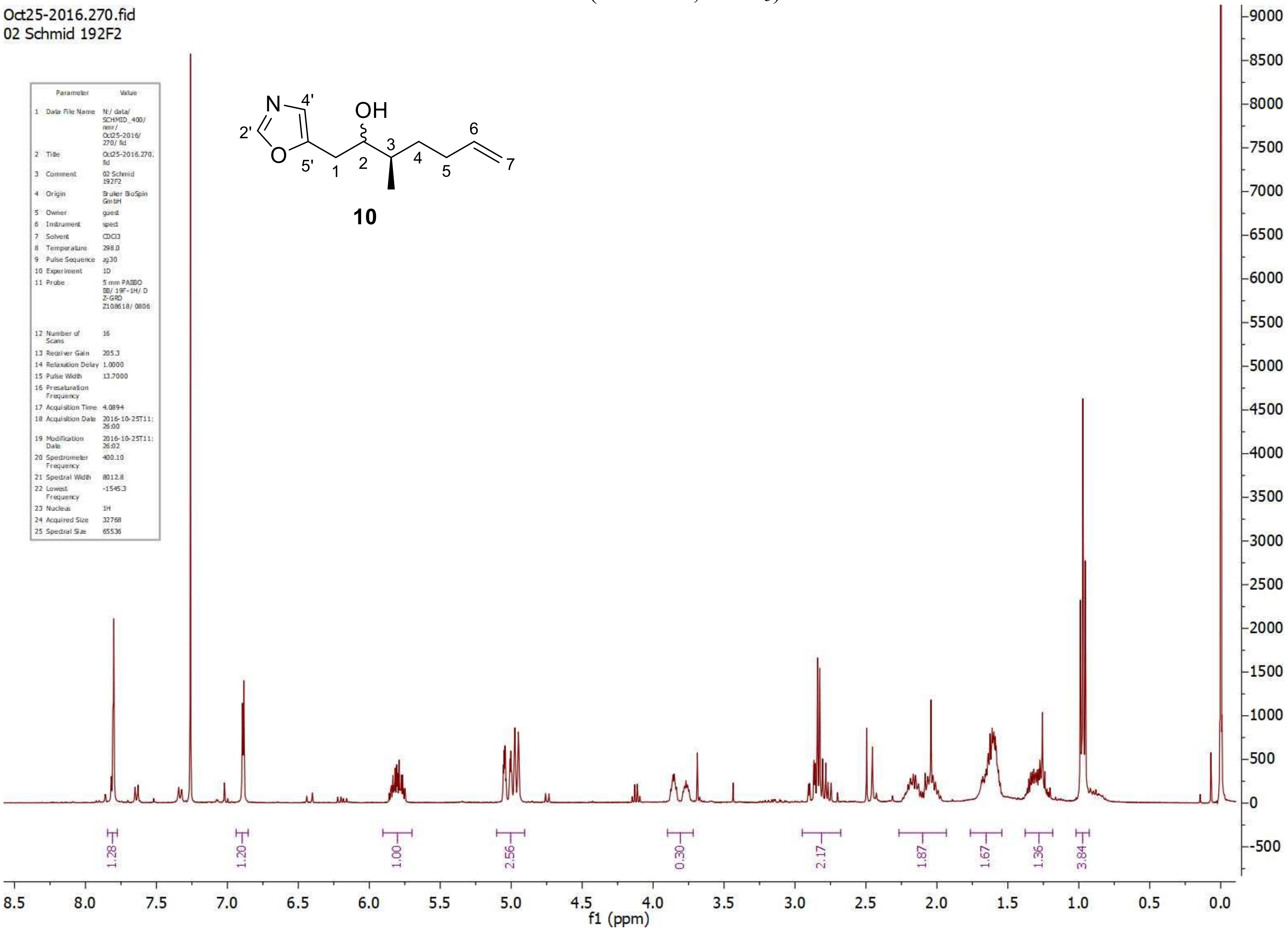

^{13}C NMR (100 MHz, $CDCl_3$) of **10**

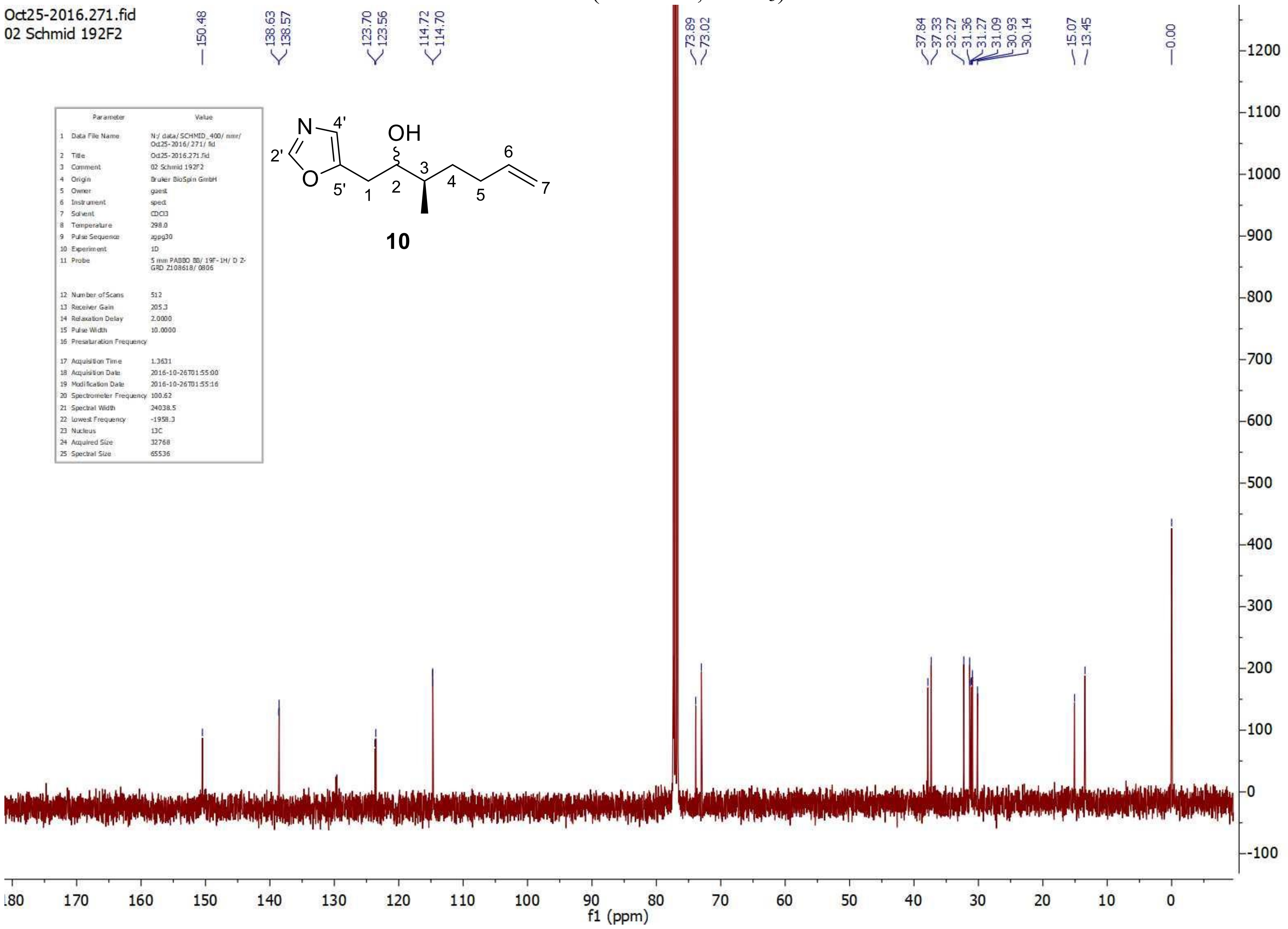

^{1}H NMR (700 MHz, $CDCl_3$) of Ts-**S8**

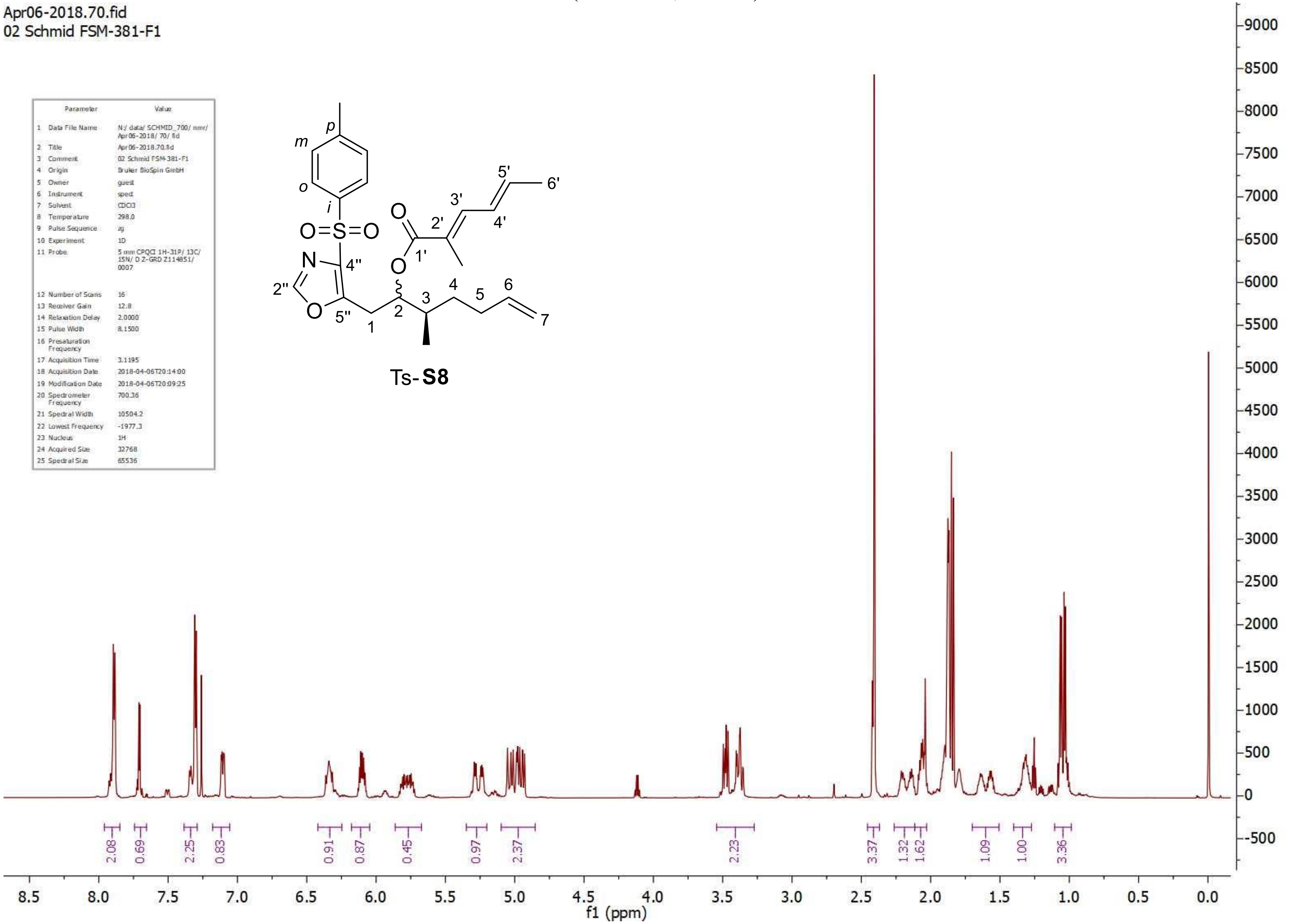

^{13}**C NMR** (176 MHz, $CDCl_3$) of Ts-**S8**

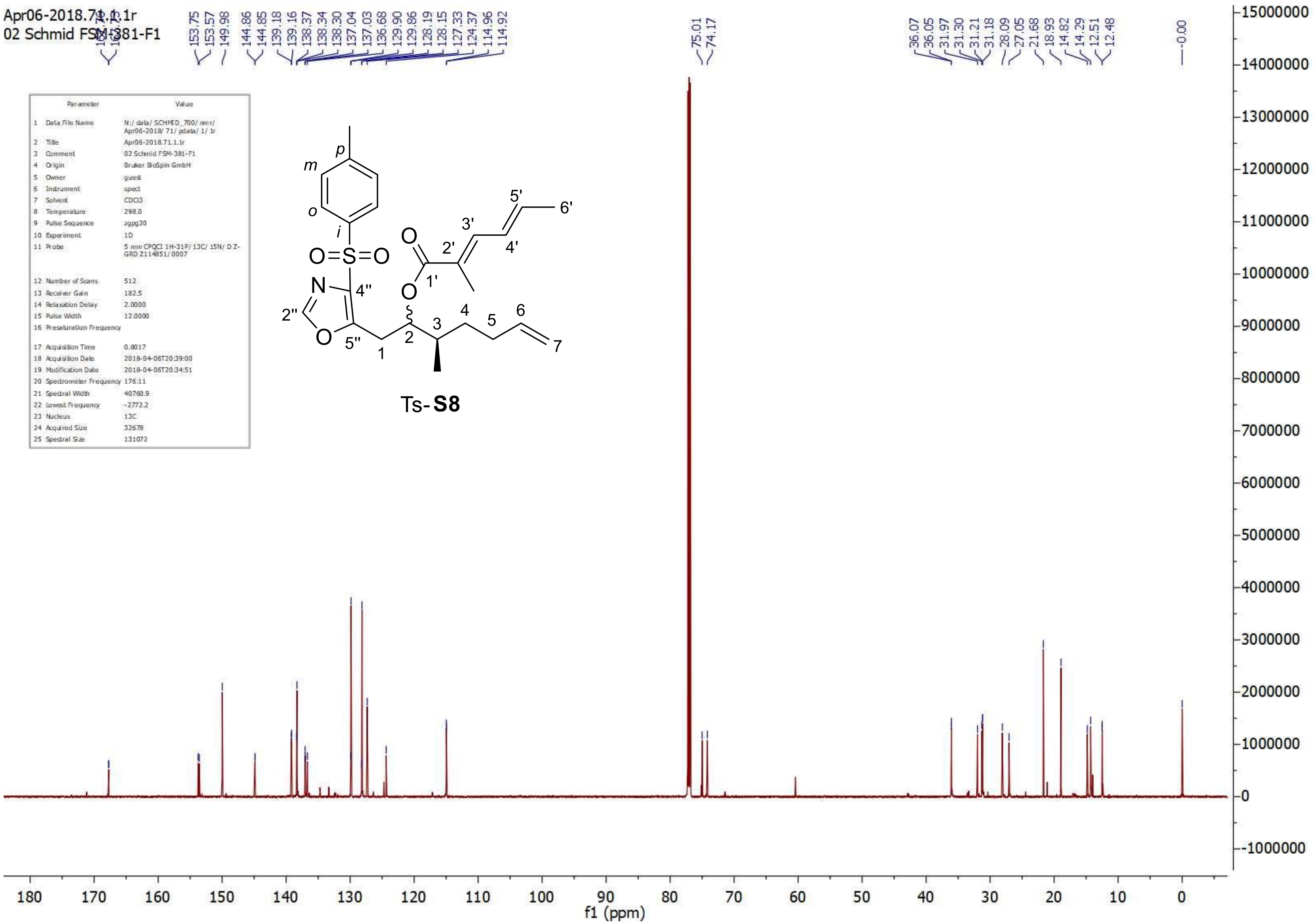

^{1}H NMR (500 MHz, $CDCl_3$) of **S8**

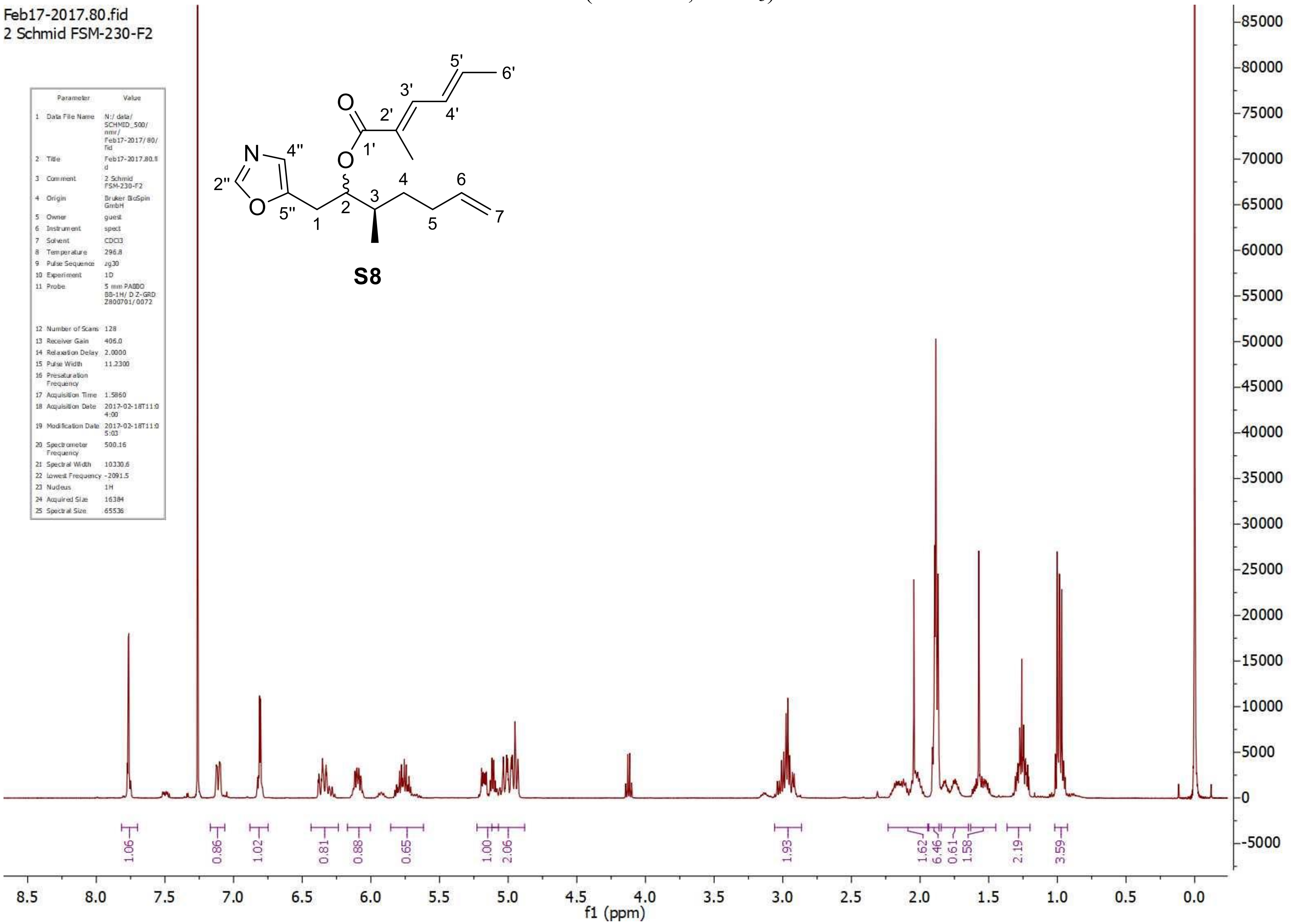

^{13}C NMR (125 MHz, $CDCl_3$) of **S8**

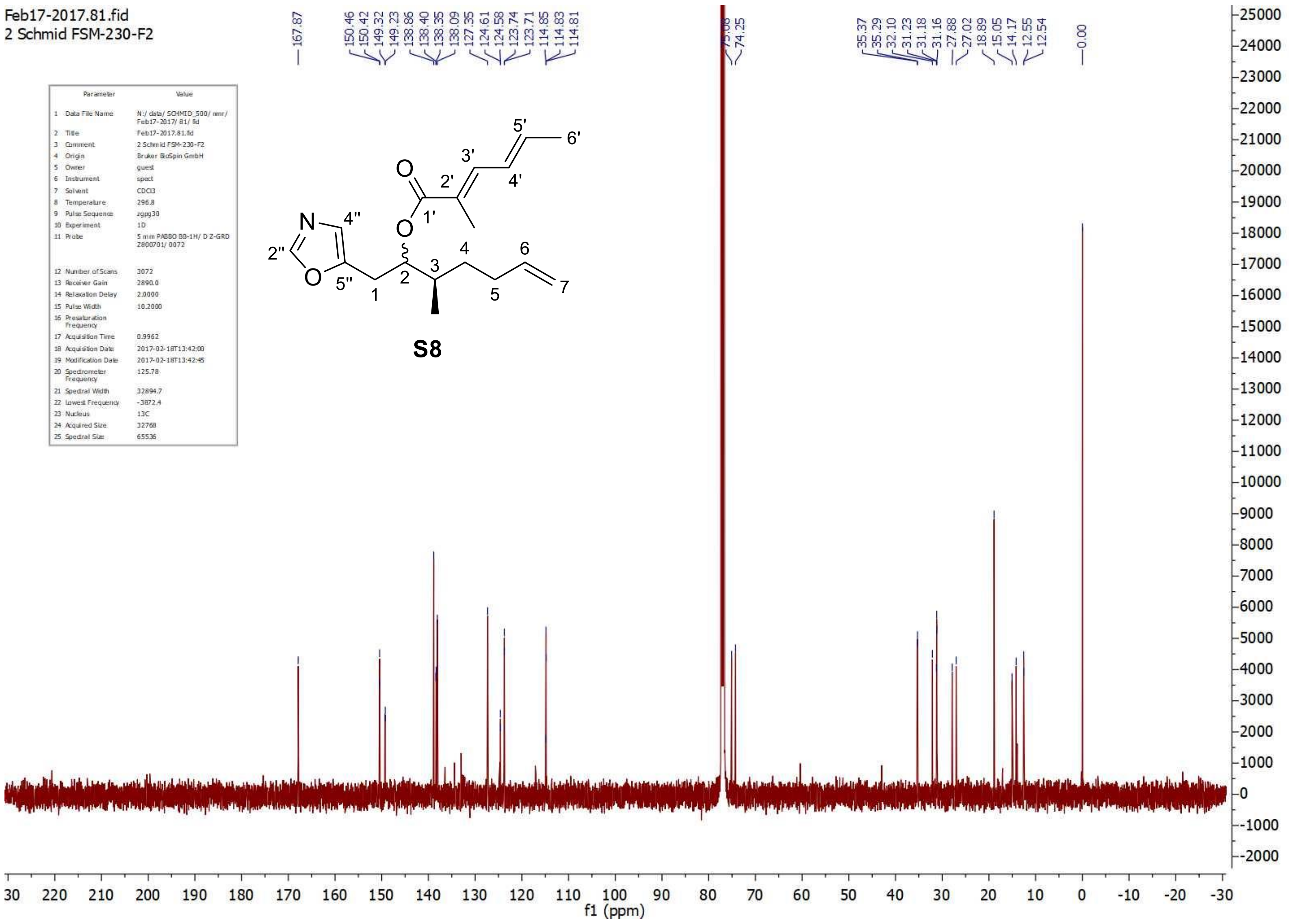

^{1}H NMR (500 MHz, $CDCl_3$) of **17**

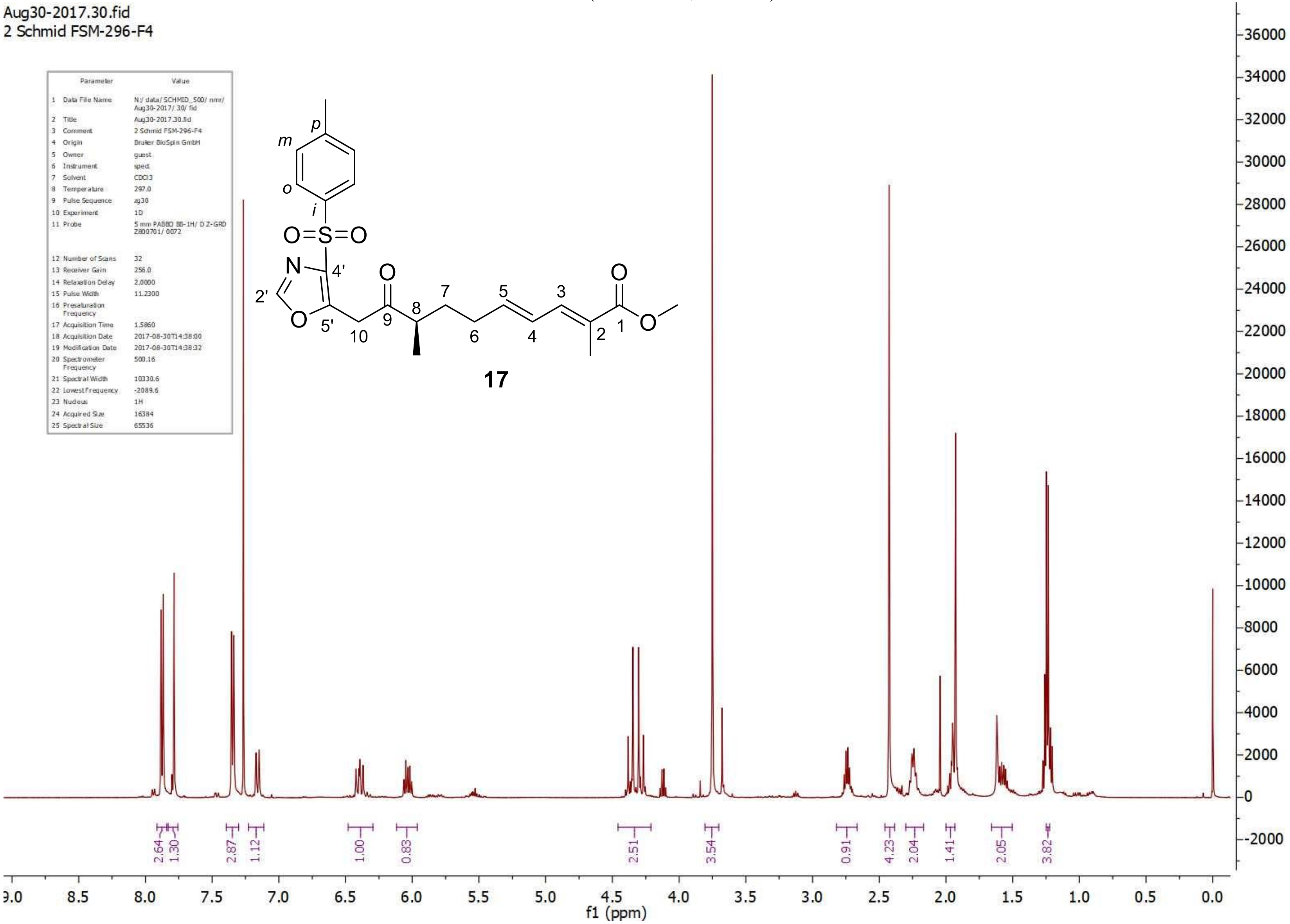

^{13}**C NMR** (125 MHz, $CDCl_3$) of **17**

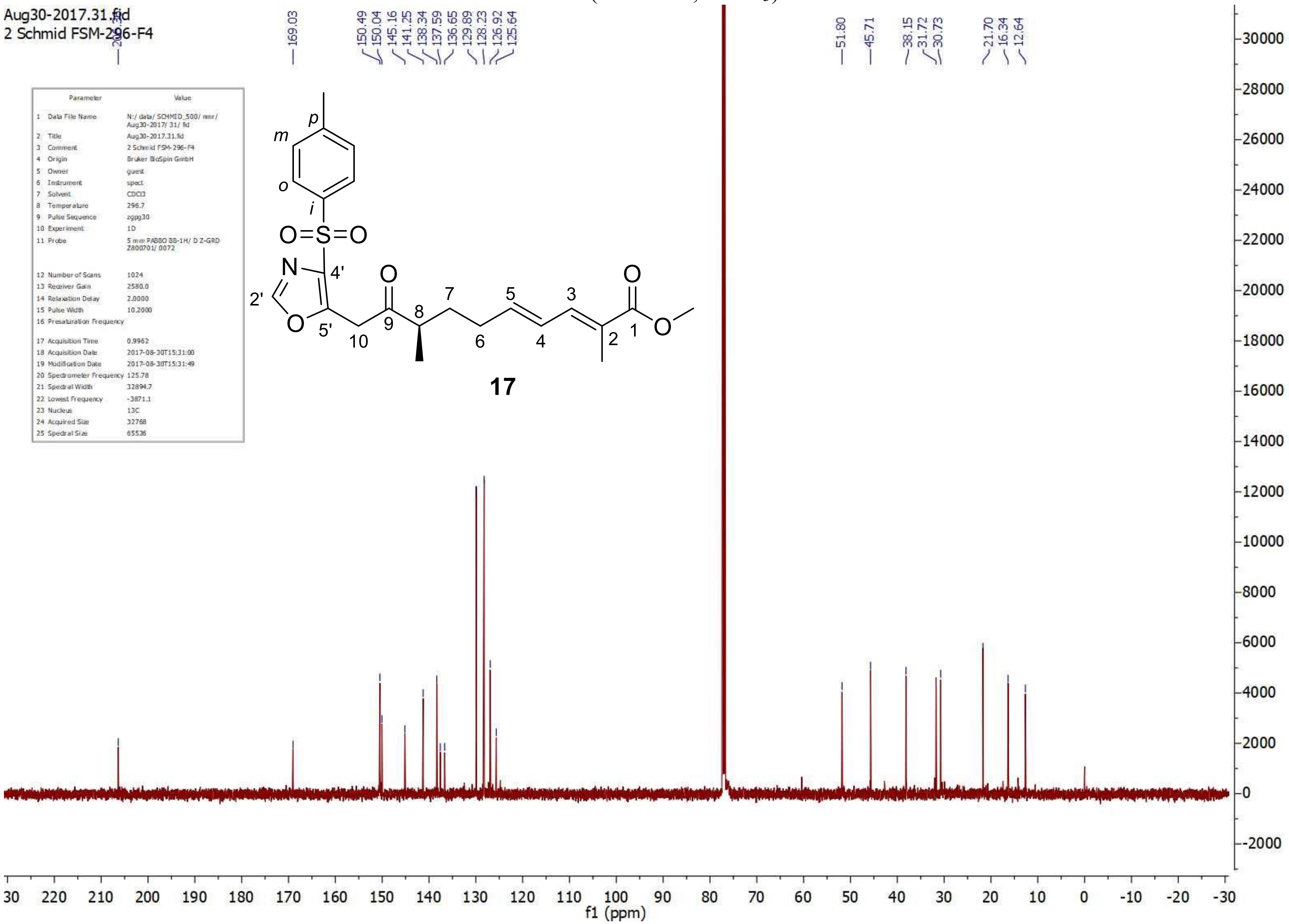

^{1}H NMR (400 MHz, $CDCl_3$) of *syn*-Ts-**16**

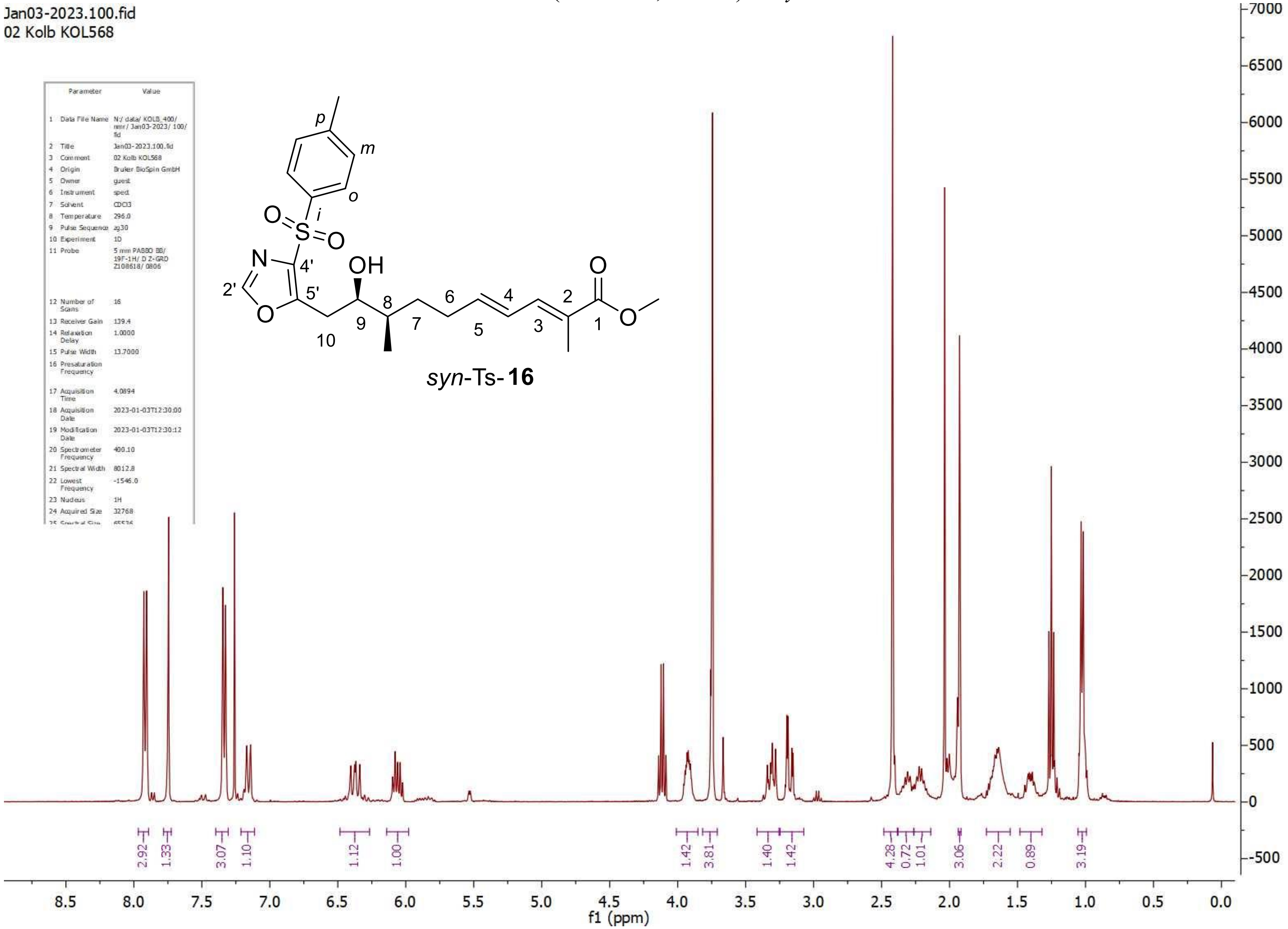

^{13}C NMR (100 MHz, $CDCl_3$) of *syn*-Ts-**16**

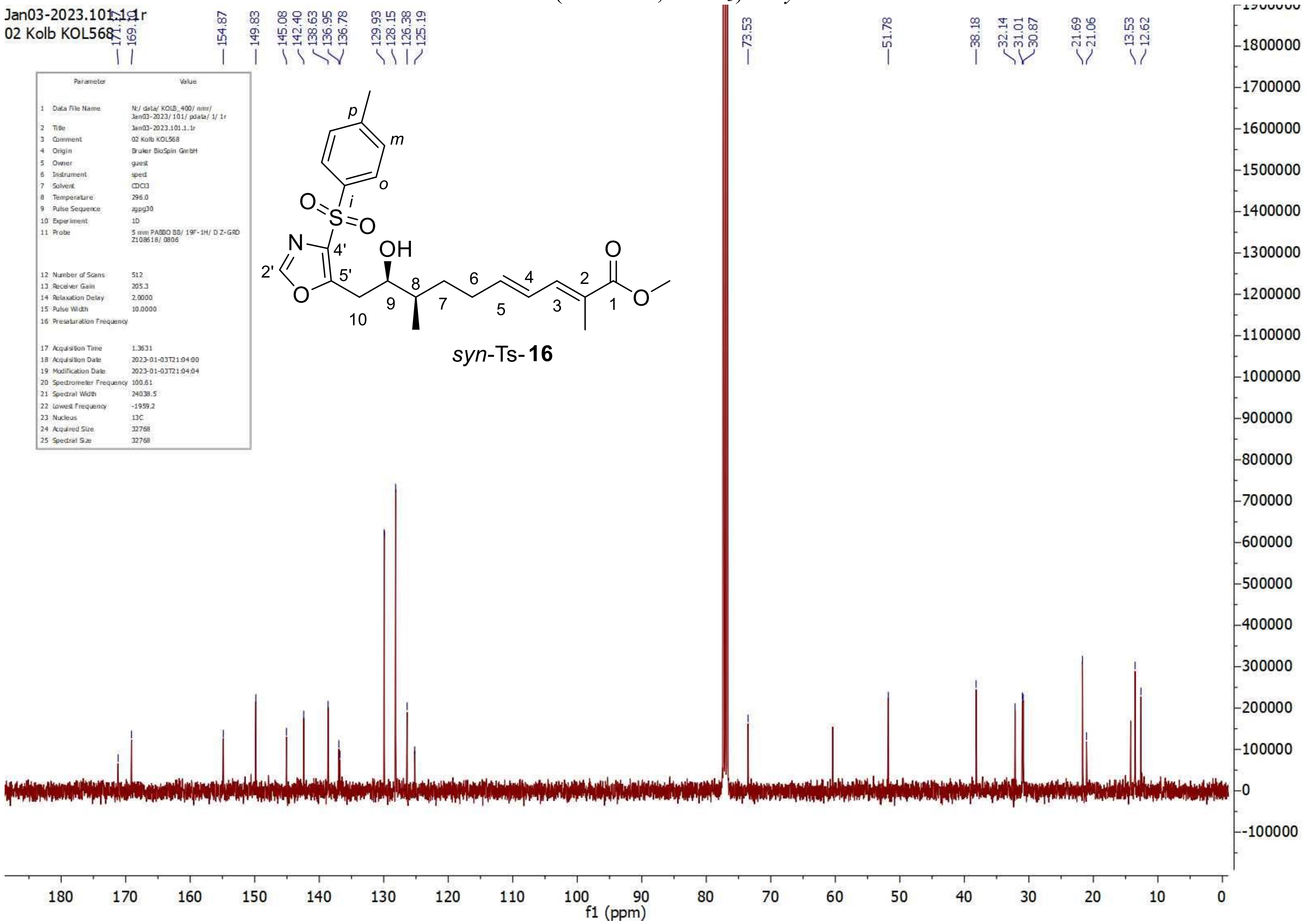

^{1}H NMR (400 MHz, $CDCl_3$) of *anti*-Ts-16

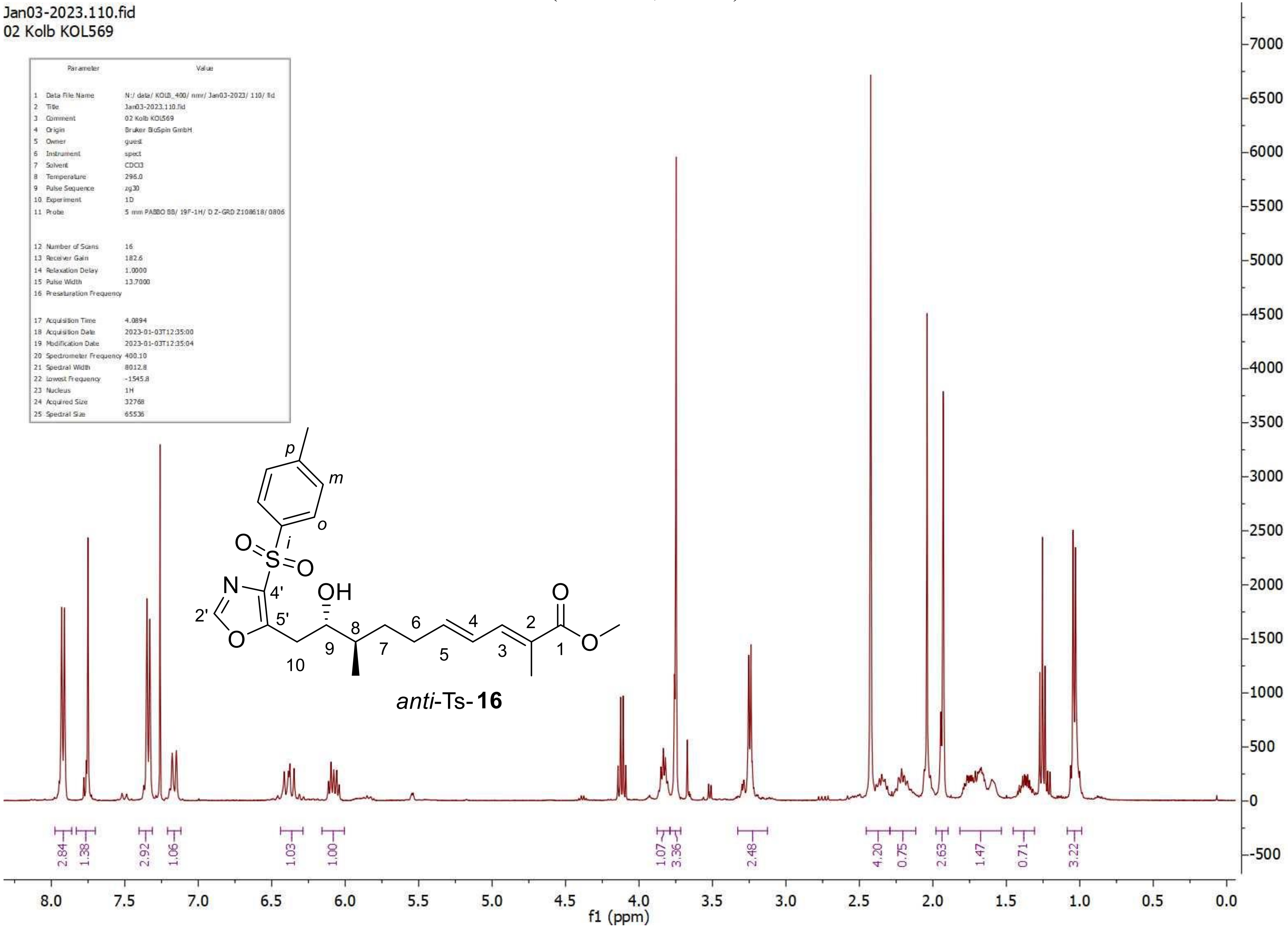

^{13}C NMR (100 MHz, CDCl$_3$) of *anti*-Ts-**16**

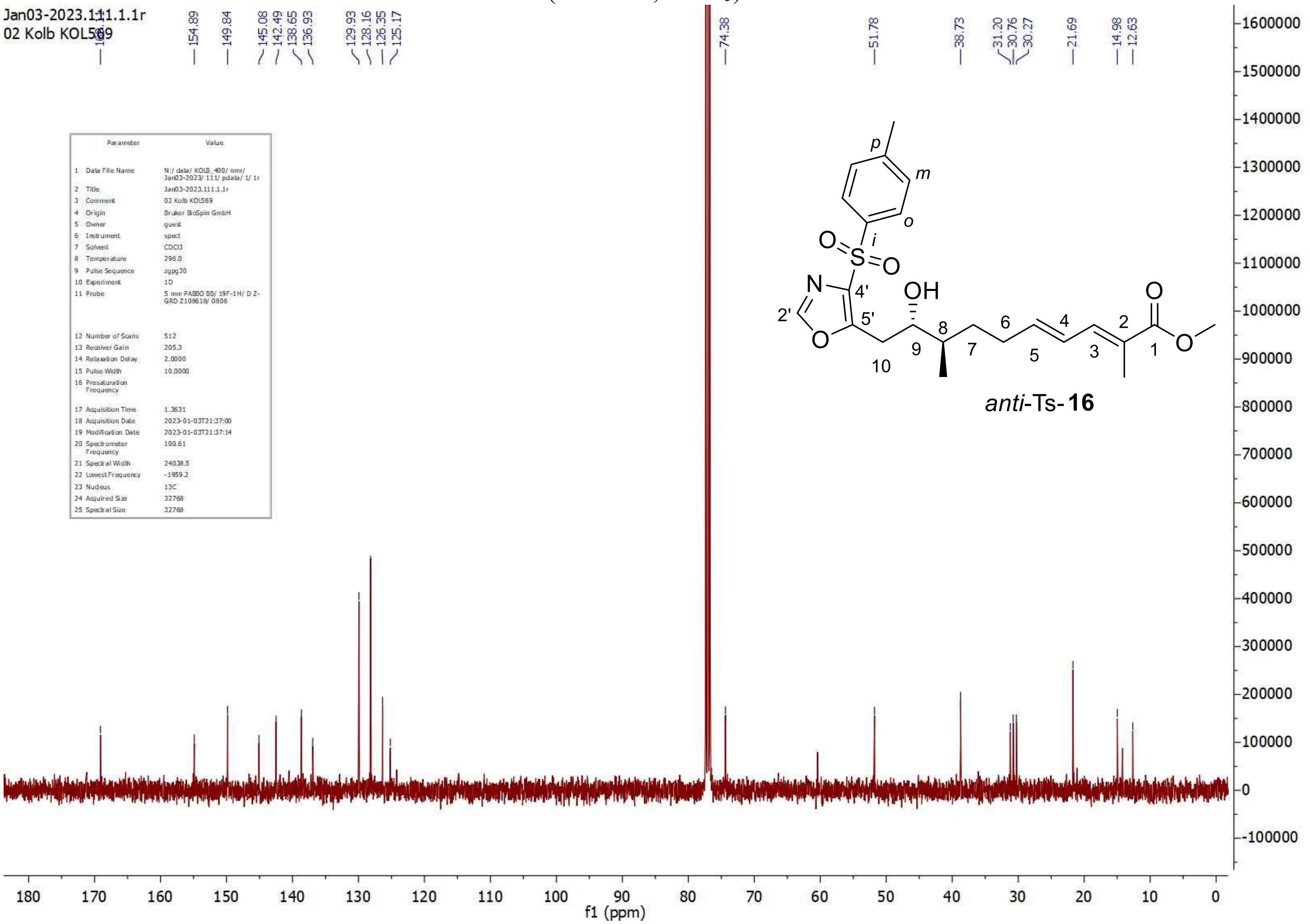

^{1}H NMR (700 MHz, $CDCl_3$) of *syn*-Ts-**8**

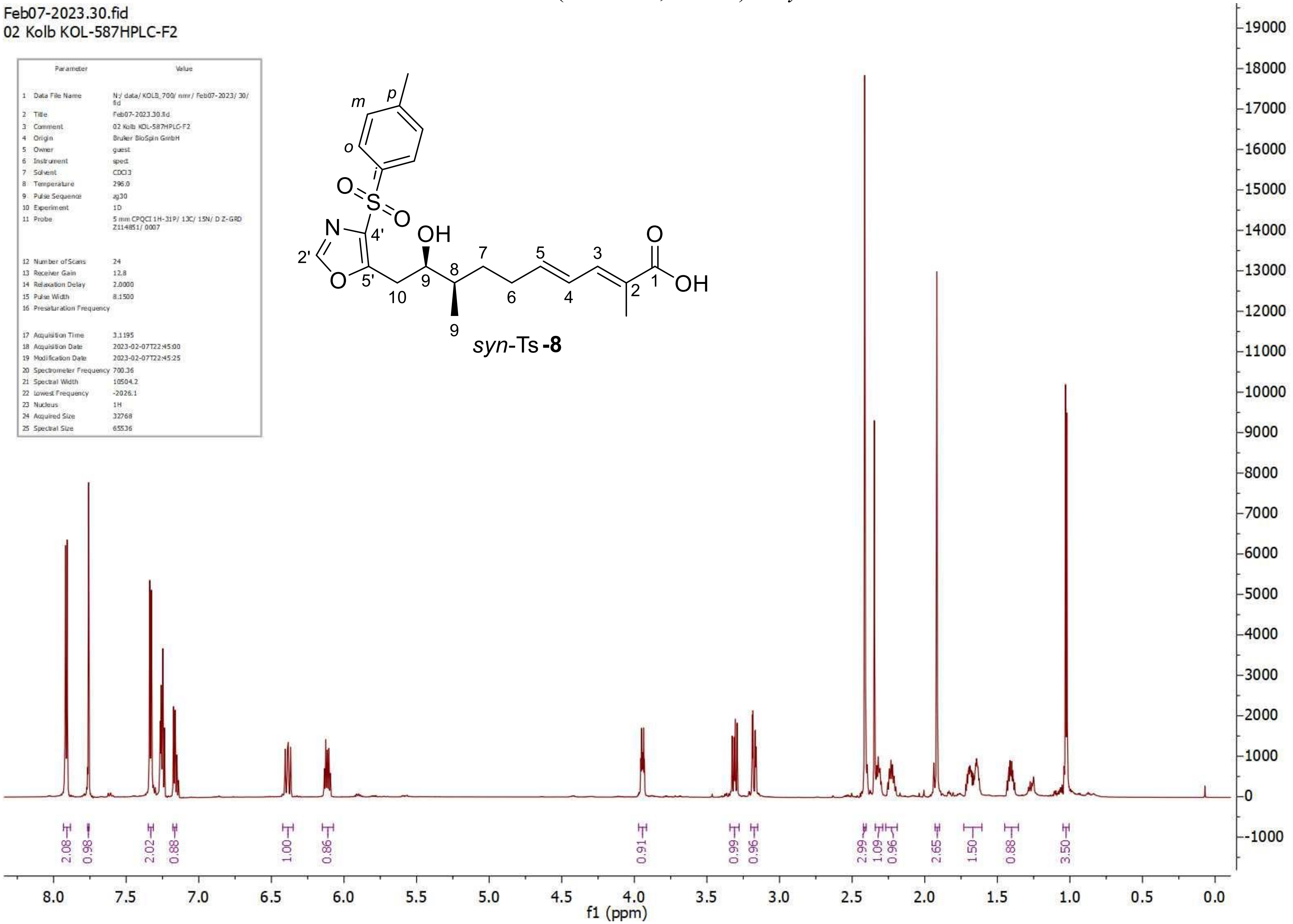

^{13}C NMR (176 MHz, $CDCl_3$) of *syn*-Ts-**8**

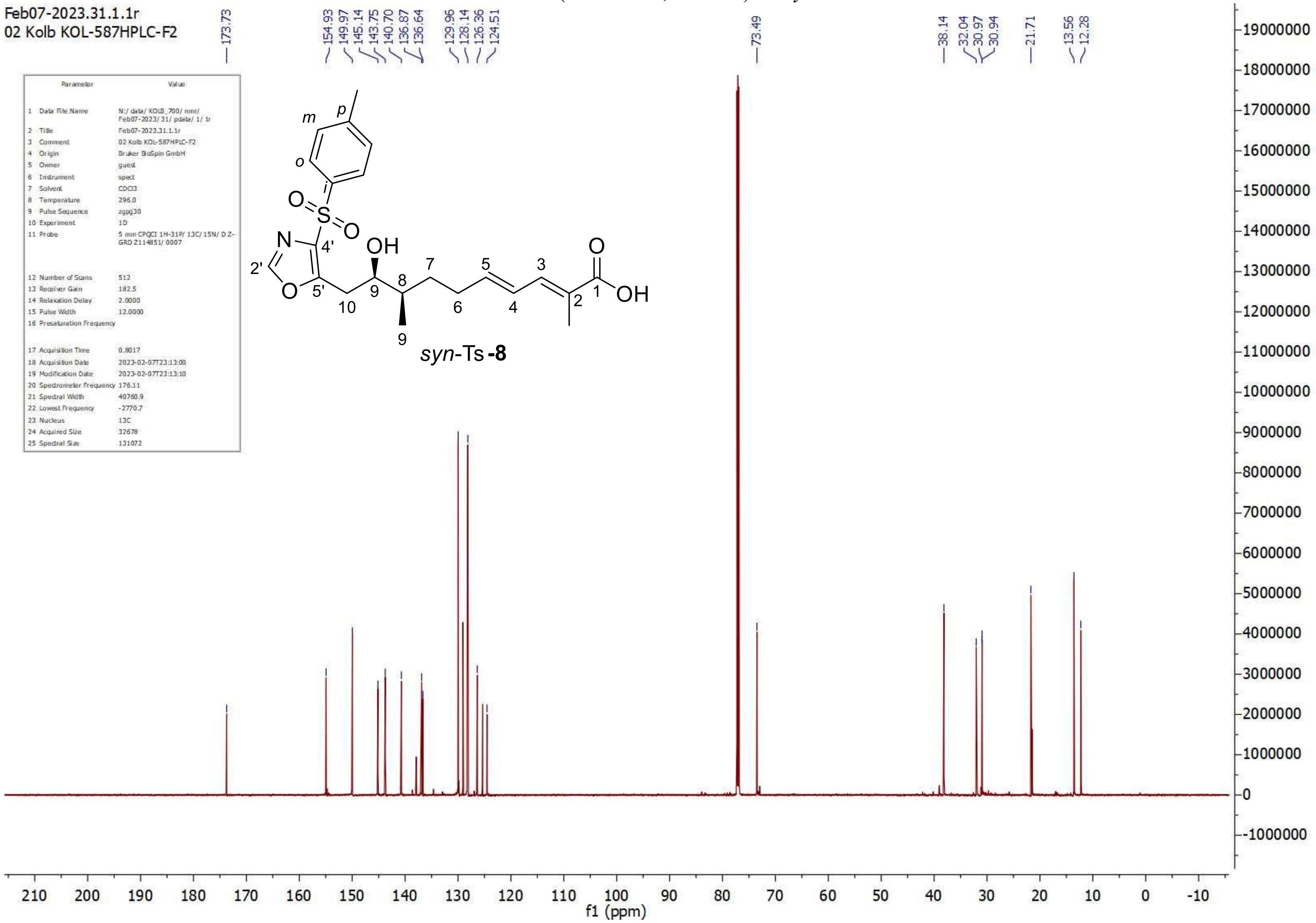

MS (ESI) of *syn*-Ts-**8**

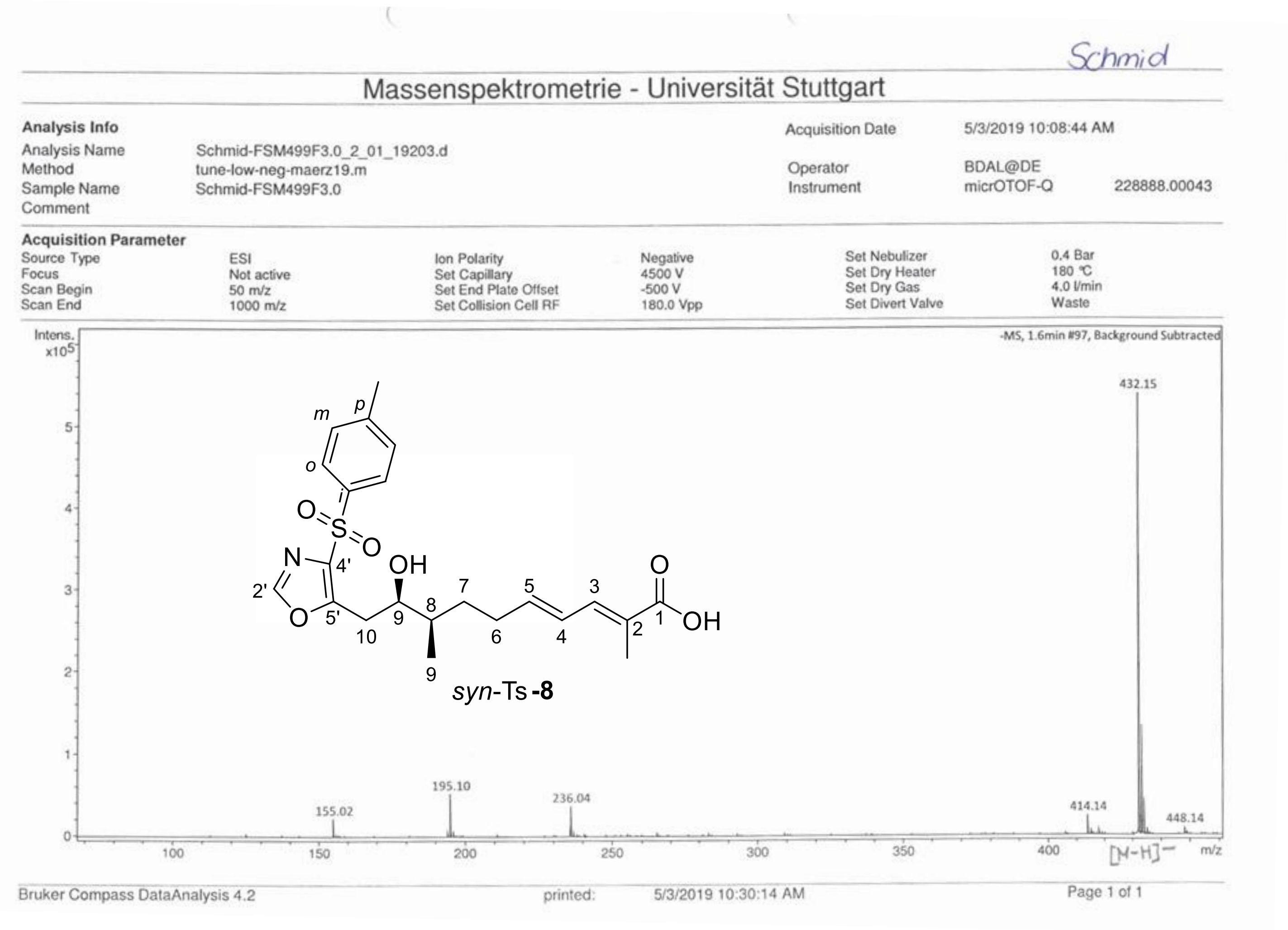

HRMS of *syn*-Ts-**8**

Massenspektrometrie - Universität Stuttgart

Analysis Info

Analysis Name	Schmid-FSM499F3.0_2_01_19203.d	Acquisition Date	5/3/2019 10:08:44 AM
Method	tune-low-neg-maerz19.m	Operator	BDAL@DE
Sample Name	Schmid-FSM499F3.0	Instrument	micrOTOF-Q 228888.00043
Comment			

Acquisition Parameter

Source Type	ESI	Ion Polarity	Negative	Set Nebulizer	0.4 Bar
Focus	Not active	Set Capillary	4500 V	Set Dry Heater	180 °C
Scan Begin	50 m/z	Set End Plate Offset	-500 V	Set Dry Gas	4.0 l/min
Scan End	1000 m/z	Set Collision Cell RF	180.0 Vpp	Set Divert Valve	Waste

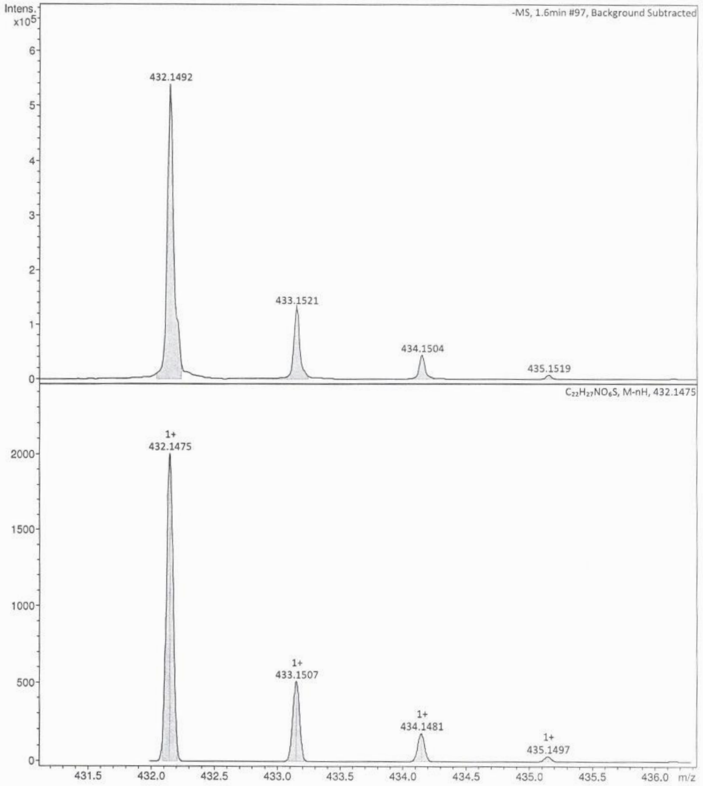

Bruker Compass DataAnalysis 4.2 printed: 5/3/2019 10:31:13 AM Page 1 of 1

^{1}H NMR (700 MHz, $CDCl_3$) of *anti*-Ts-**8**

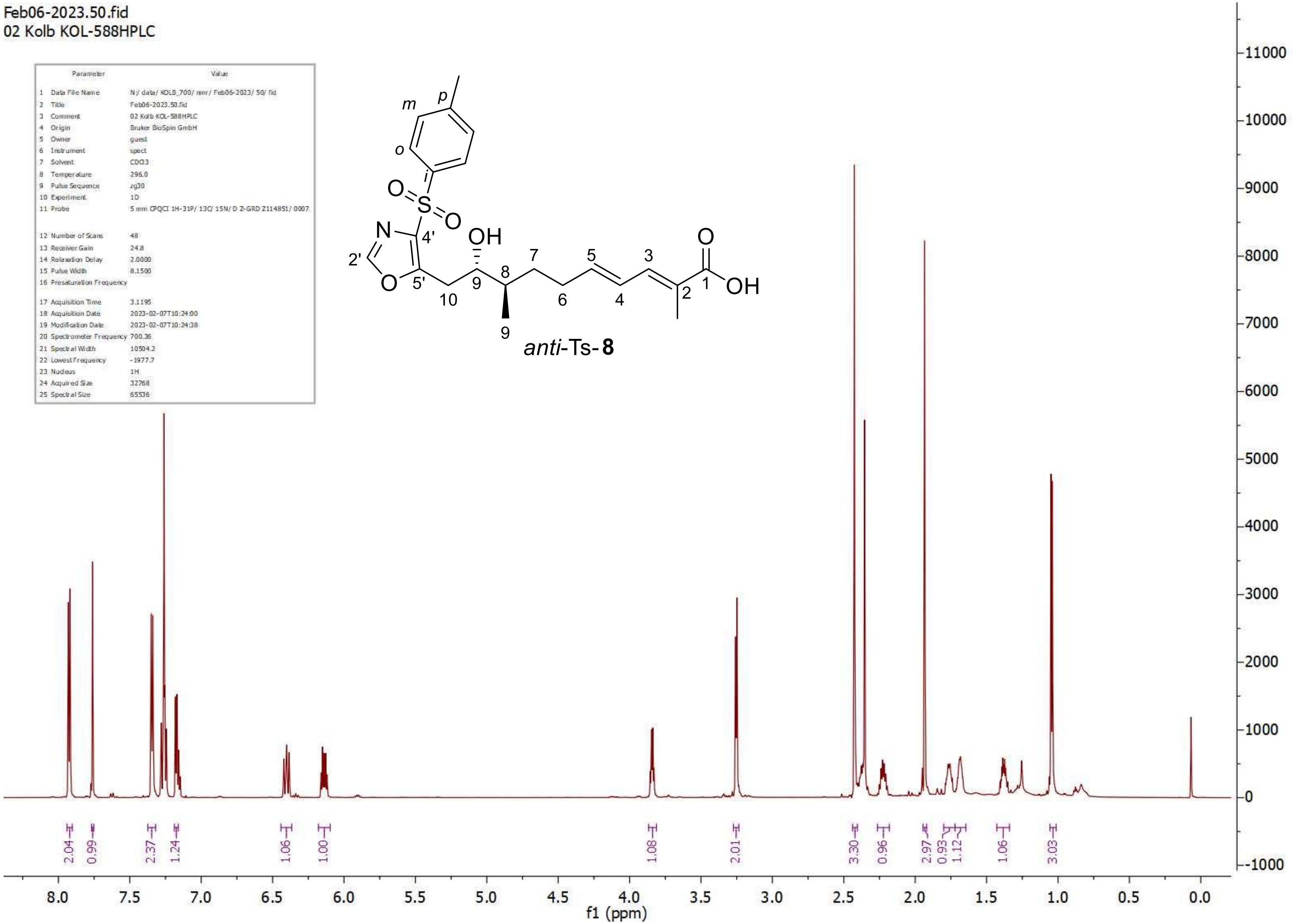

^{13}C NMR (176 MHz, CDCl$_3$) of *anti*-Ts-**8**

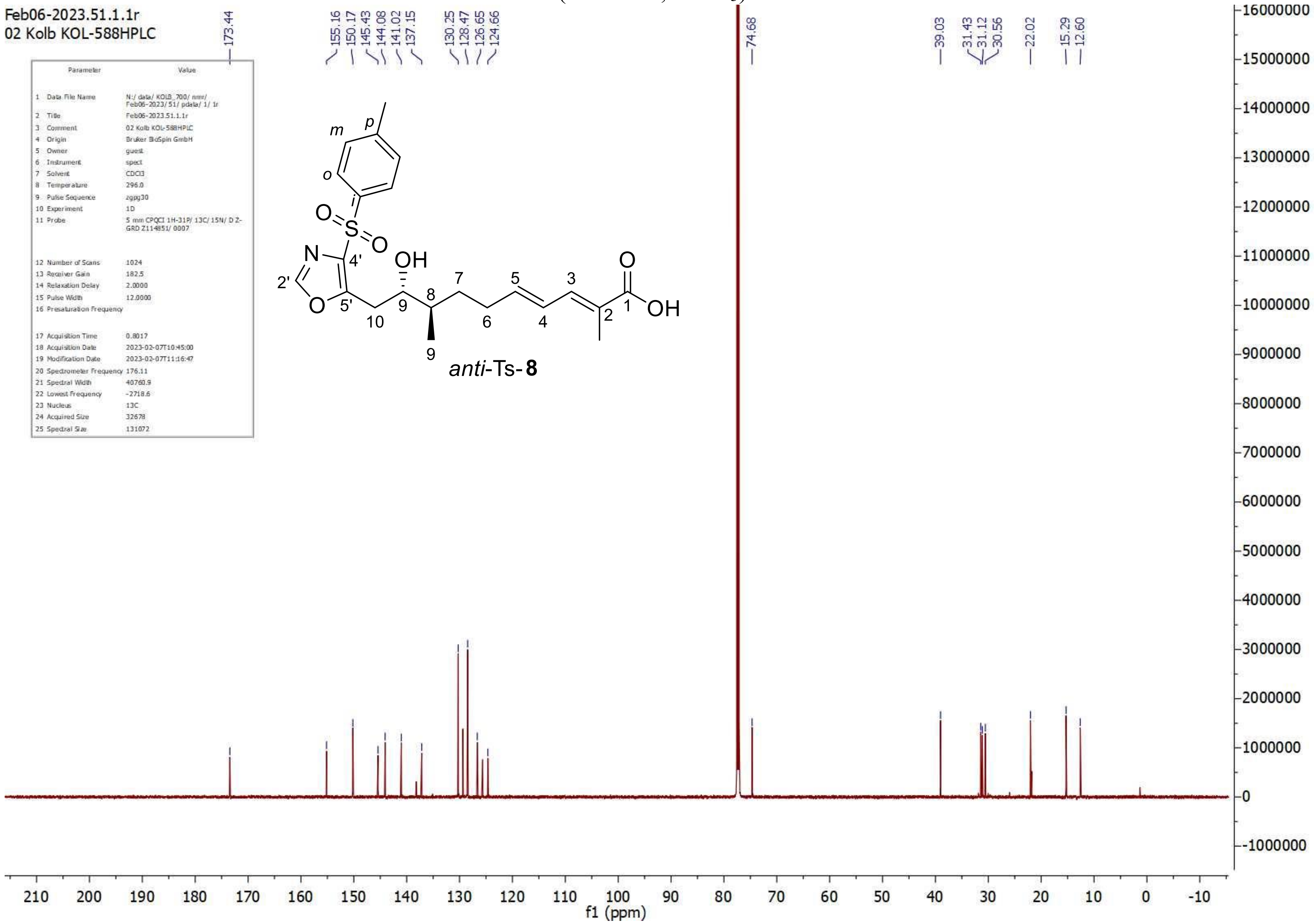

MS (ESI) of *anti*-Ts-**8**

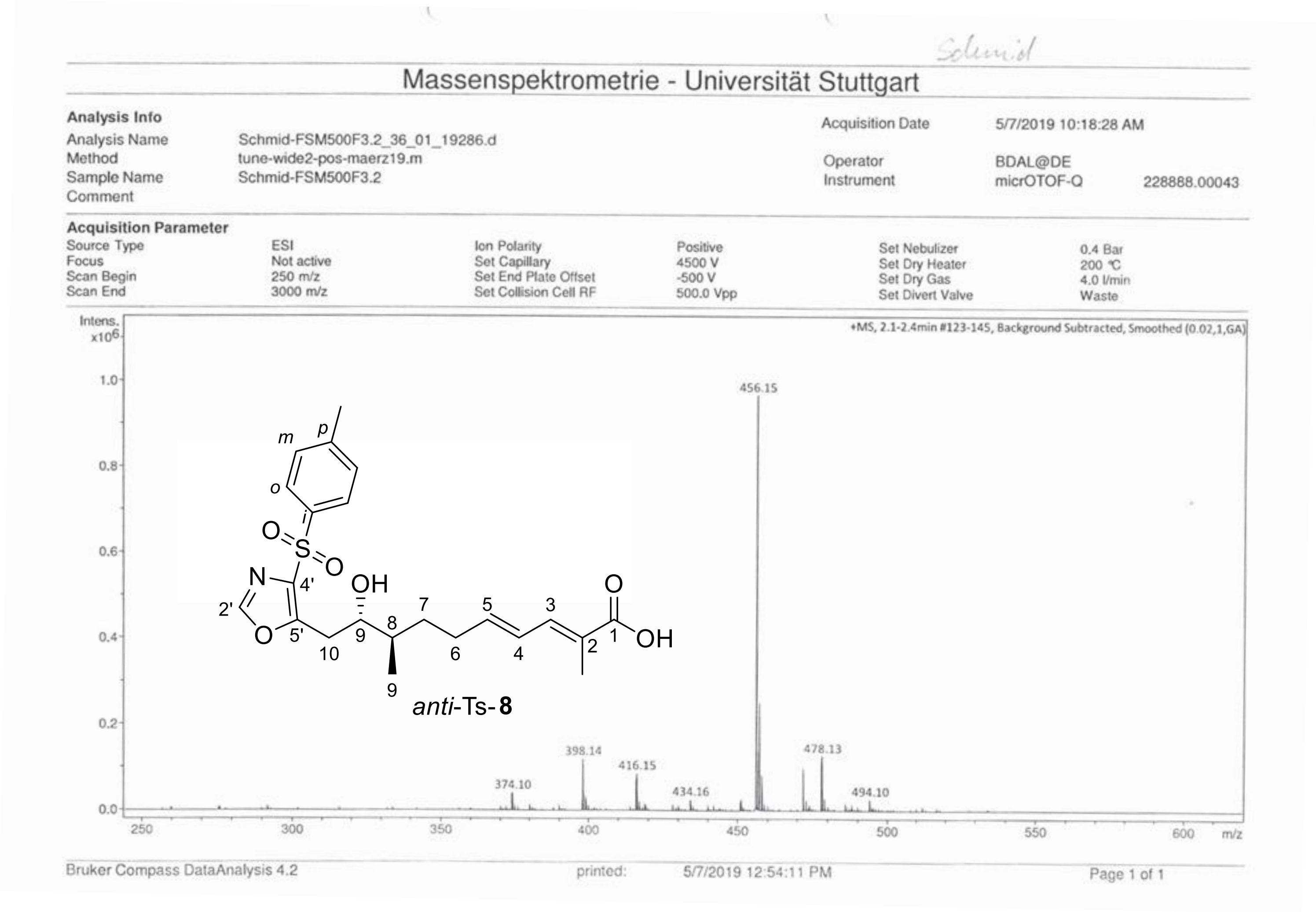

HRMS of *anti*-Ts-**8**

Massenspektrometrie - Universität Stuttgart

Analysis Info

Analysis Name	Schmid-FSM500F3.2_36_01_19286.d	Acquisition Date	5/7/2019 10:18:28 AM
Method	tune-wide2-pos-maerz19.m	Operator	BDAL@DE
Sample Name	Schmid-FSM500F3.2	Instrument	micrOTOF-Q 228888.00043
Comment			

Acquisition Parameter

Source Type	ESI	Ion Polarity	Positive	Set Nebulizer	0.4 Bar
Focus	Not active	Set Capillary	4500 V	Set Dry Heater	200 °C
Scan Begin	250 m/z	Set End Plate Offset	-500 V	Set Dry Gas	4.0 l/min
Scan End	3000 m/z	Set Collision Cell RF	500.0 Vpp	Set Divert Valve	Waste

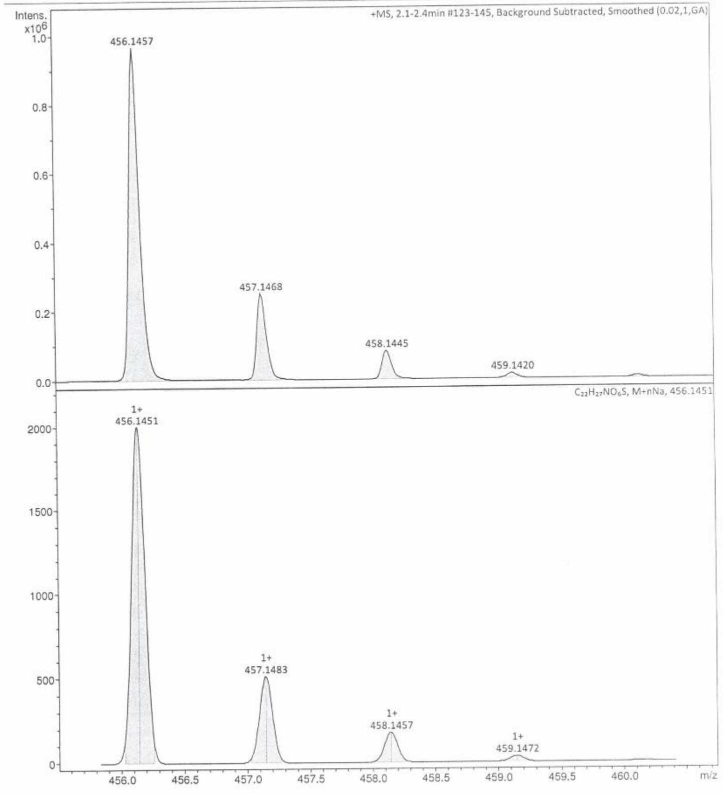

Bruker Compass DataAnalysis 4.2 printed: 5/7/2019 12:54:39 PM Page 1 of 1

^{1}H NMR (500 MHz, $CDCl_3$) of *syn*-**8**

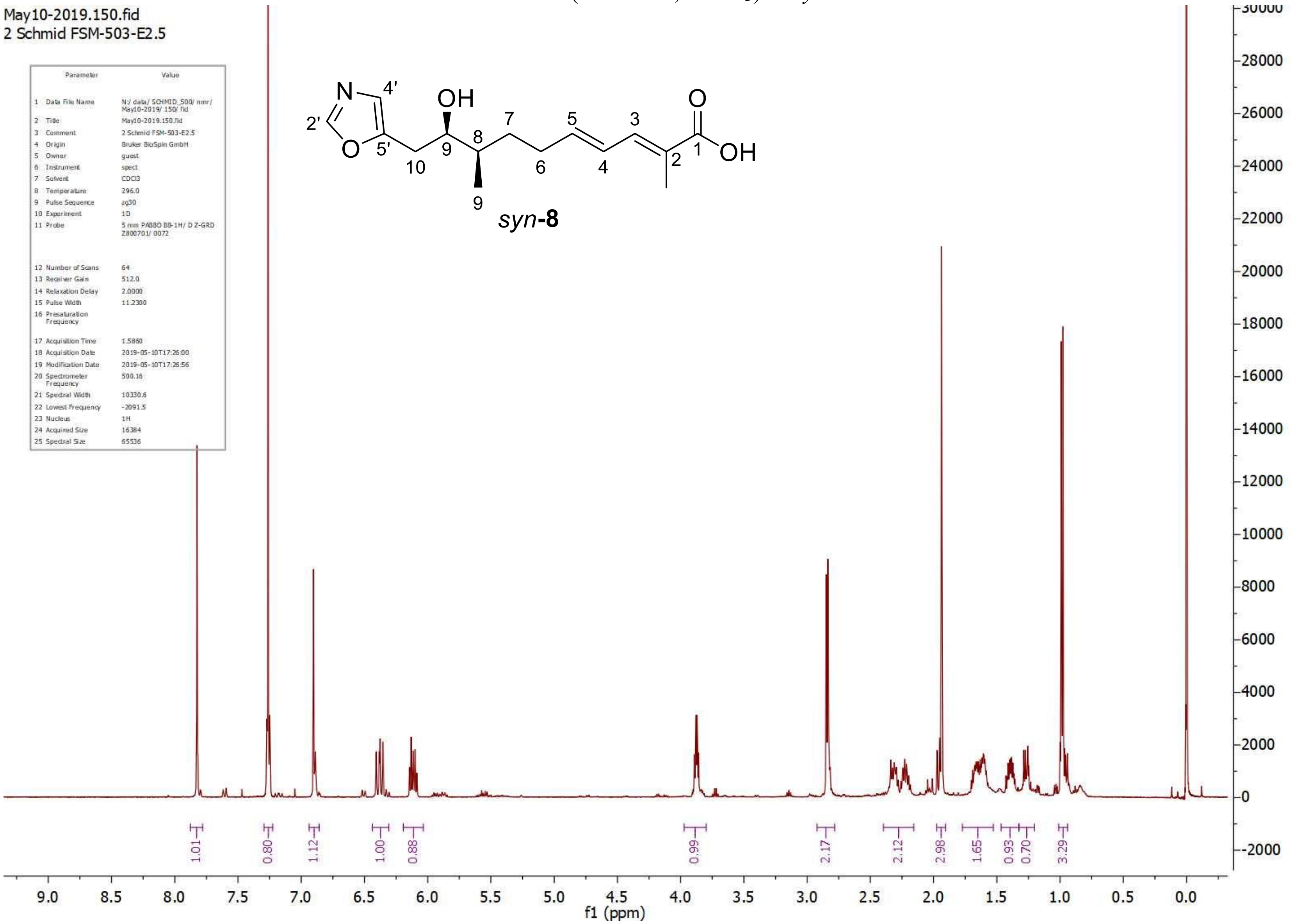

^{13}C NMR (125 MHz, $CDCl_3$) of *syn*-8

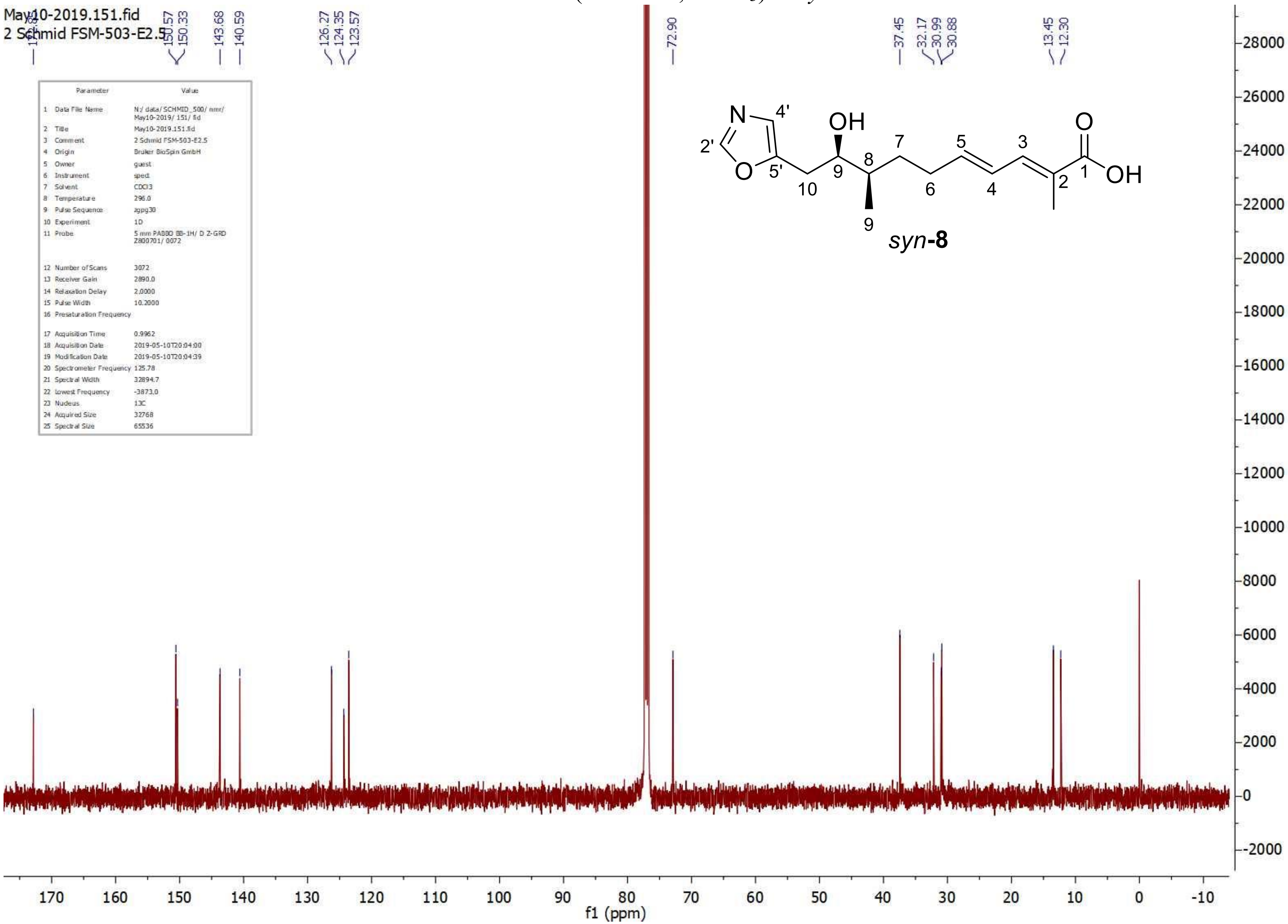

MS (ESI) of *syn*-**8**

Schmid

Massenspektrometrie - Universität Stuttgart

Analysis Info

Analysis Name	Schmid-FSM503F2.5_4_01_19383.d	Acquisition Date	5/13/2019 10:57:39 AM
Method	tune-low-neg-maerz19.m	Operator	BDAL@DE
Sample Name	Schmid-FSM503F2.5	Instrument	micrOTOF-Q 228888.00043
Comment			

Acquisition Parameter

Source Type	ESI	Ion Polarity	Negative	Set Nebulizer	0.4 Bar
Focus	Not active	Set Capillary	4500 V	Set Dry Heater	180 °C
Scan Begin	50 m/z	Set End Plate Offset	-500 V	Set Dry Gas	4.0 l/min
Scan End	1000 m/z	Set Collision Cell RF	180.0 Vpp	Set Divert Valve	Waste

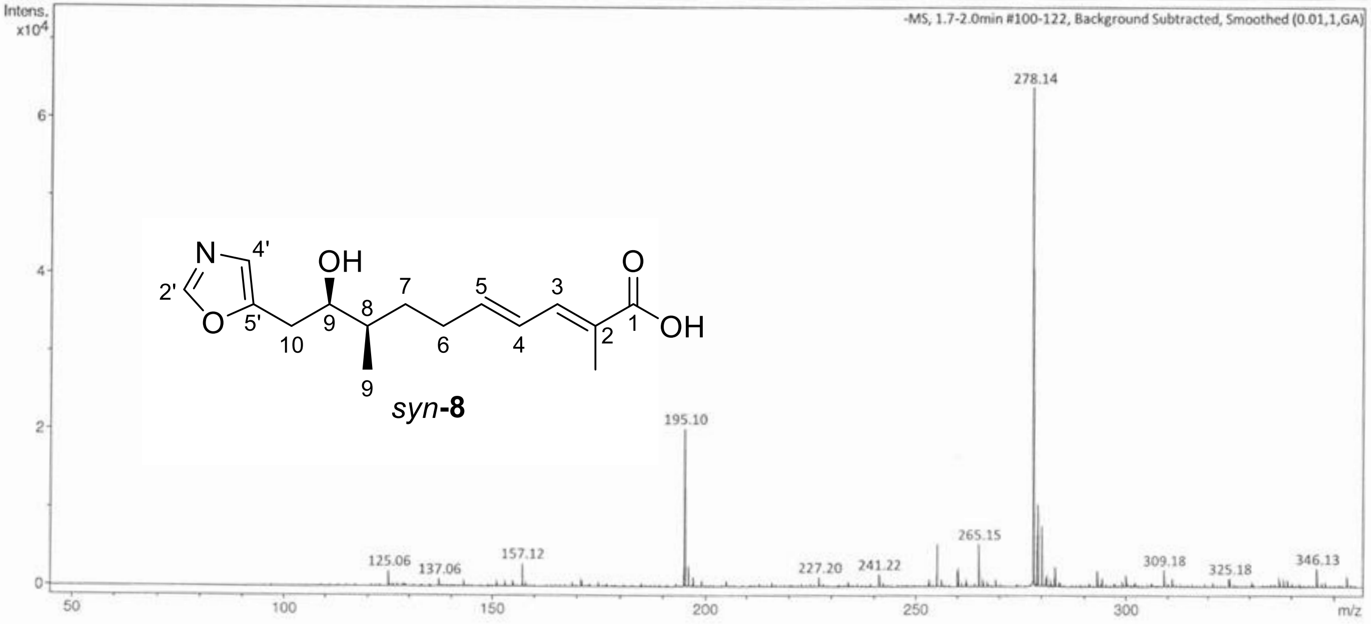

Bruker Compass DataAnalysis 4.2 printed: 5/13/2019 11:49:00 AM Page 1 of 1

HRMS of *syn*-**8**

Massenspektrometrie - Universität Stuttgart

Analysis Info

Analysis Name	Schmid-FSM503F2.5_4_01_19383.d	Acquisition Date	5/13/2019 10:57:39 AM
Method	tune-low-neg-maerz19.m	Operator	BDAL@DE
Sample Name	Schmid-FSM503F2.5	Instrument	micrOTOF-Q 228888.00043
Comment			

Acquisition Parameter

Source Type	ESI	Ion Polarity	Negative	Set Nebulizer	0.4 Bar
Focus	Not active	Set Capillary	4500 V	Set Dry Heater	180 °C
Scan Begin	50 m/z	Set End Plate Offset	-500 V	Set Dry Gas	4.0 l/min
Scan End	1000 m/z	Set Collision Cell RF	180.0 Vpp	Set Divert Valve	Waste

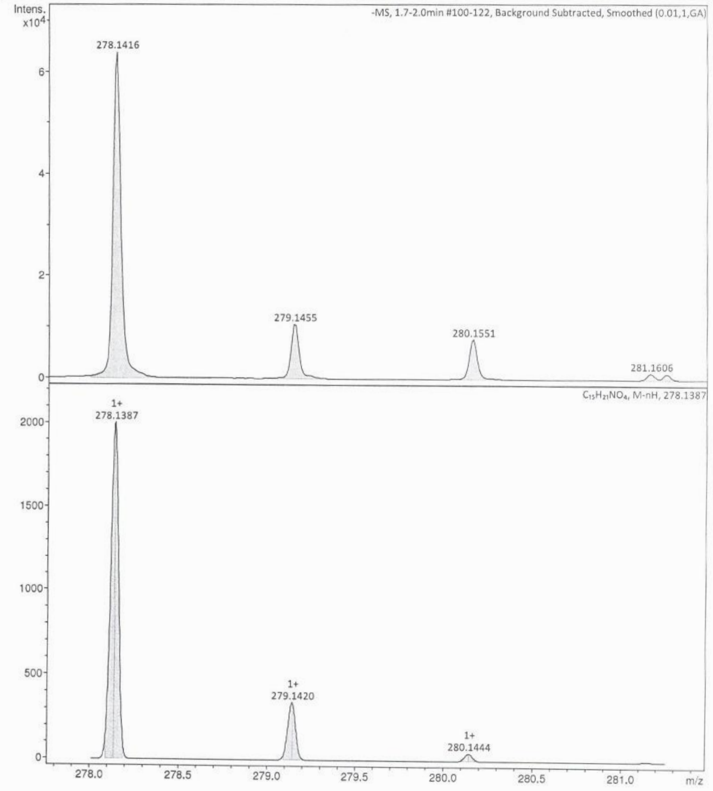

Bruker Compass DataAnalysis 4.2 printed: 5/13/2019 11:49:37 AM Page 1 of 1

^{1}H NMR (500 MHz, $CDCl_3$) of *anti*-**8**

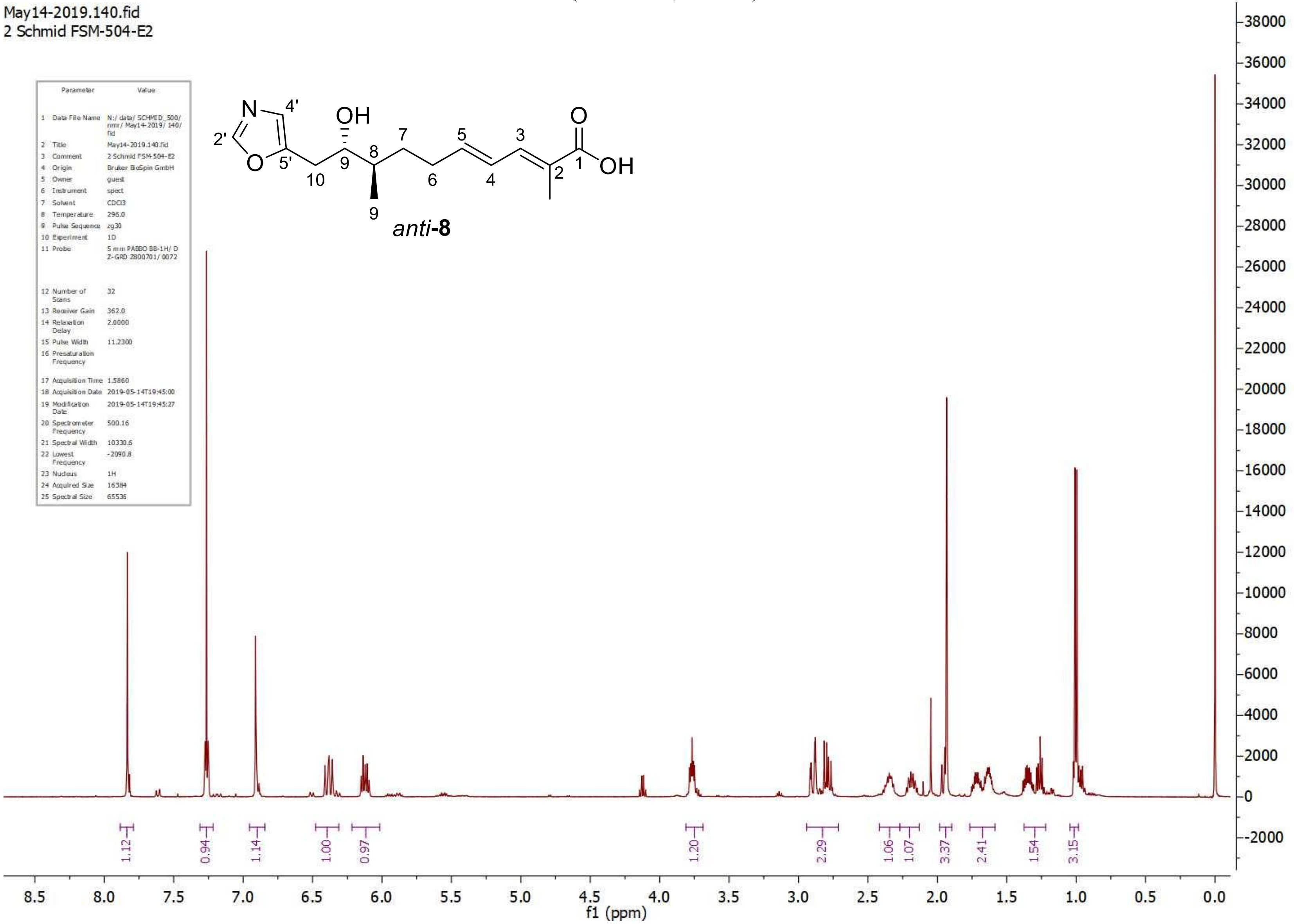

^{13}C NMR (125 MHz, $CDCl_3$) of *anti*-**8**

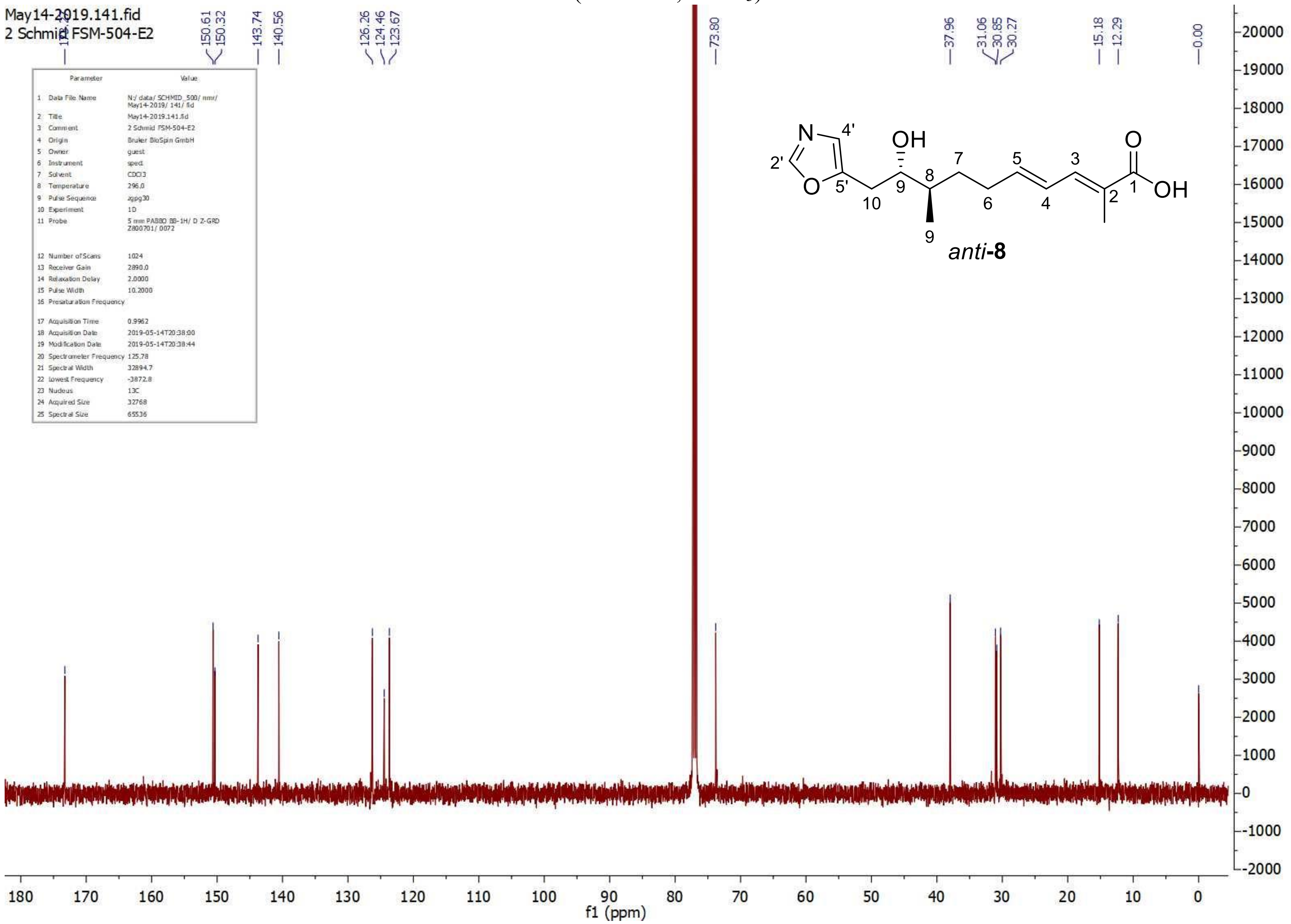

MS (ESI) of *anti*-**8**

Schmid

Massenspektrometrie - Universität Stuttgart

Analysis Info

Analysis Name	Schmid-FSM504E2_38_01_19437.d	Acquisition Date	5/15/2019 11:23:47 AM
Method	tune-low-neg-maerz19.m	Operator	BDAL@DE
Sample Name	Schmid-FSM504E2	Instrument	micrOTOF-Q 228888.00043
Comment			

Acquisition Parameter

Source Type	ESI	Ion Polarity	Negative	Set Nebulizer	0.4 Bar
Focus	Not active	Set Capillary	4500 V	Set Dry Heater	180 °C
Scan Begin	50 m/z	Set End Plate Offset	-500 V	Set Dry Gas	4.0 l/min
Scan End	1000 m/z	Set Collision Cell RF	180.0 Vpp	Set Divert Valve	Waste

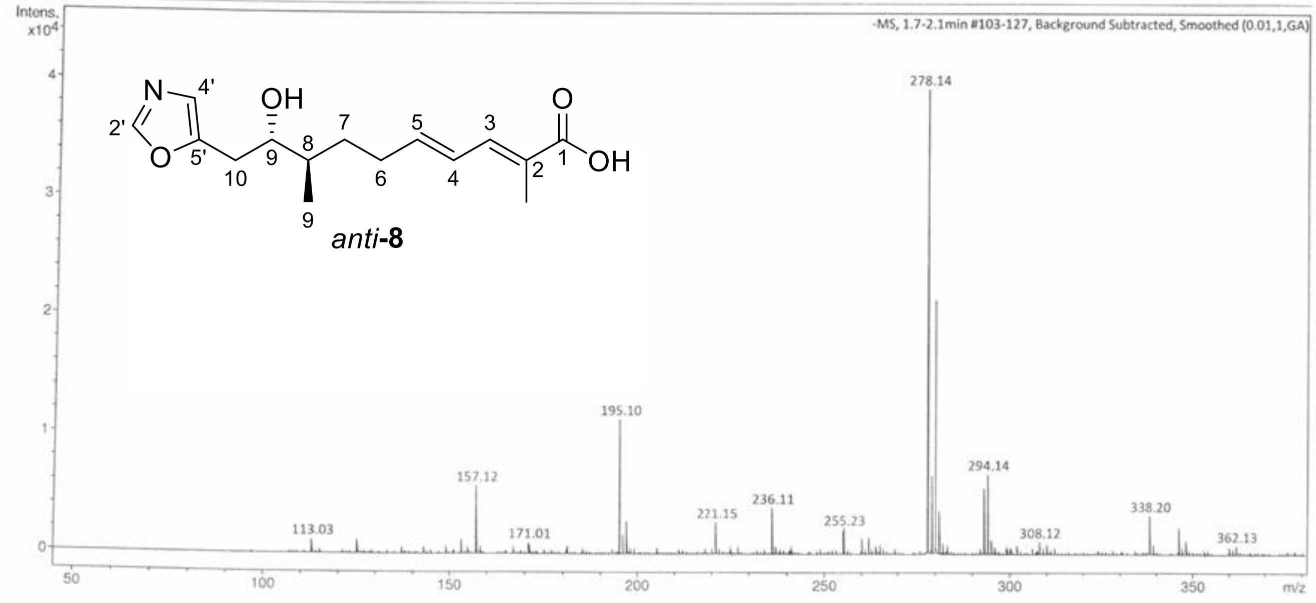

Bruker Compass DataAnalysis 4.2 printed: 5/15/2019 11:43:10 AM Page 1 of 1

HRMS of *anti*-**8**

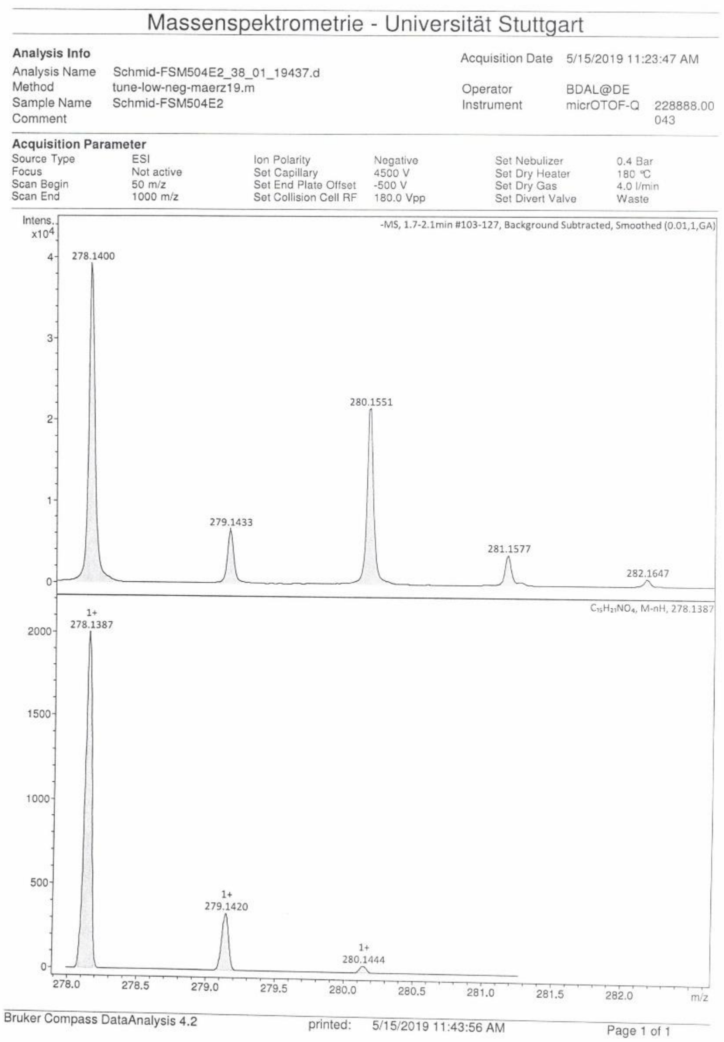

^{1}H NMR (700 MHz, $CDCl_3$) of *syn,syn*-Ts-**4a**

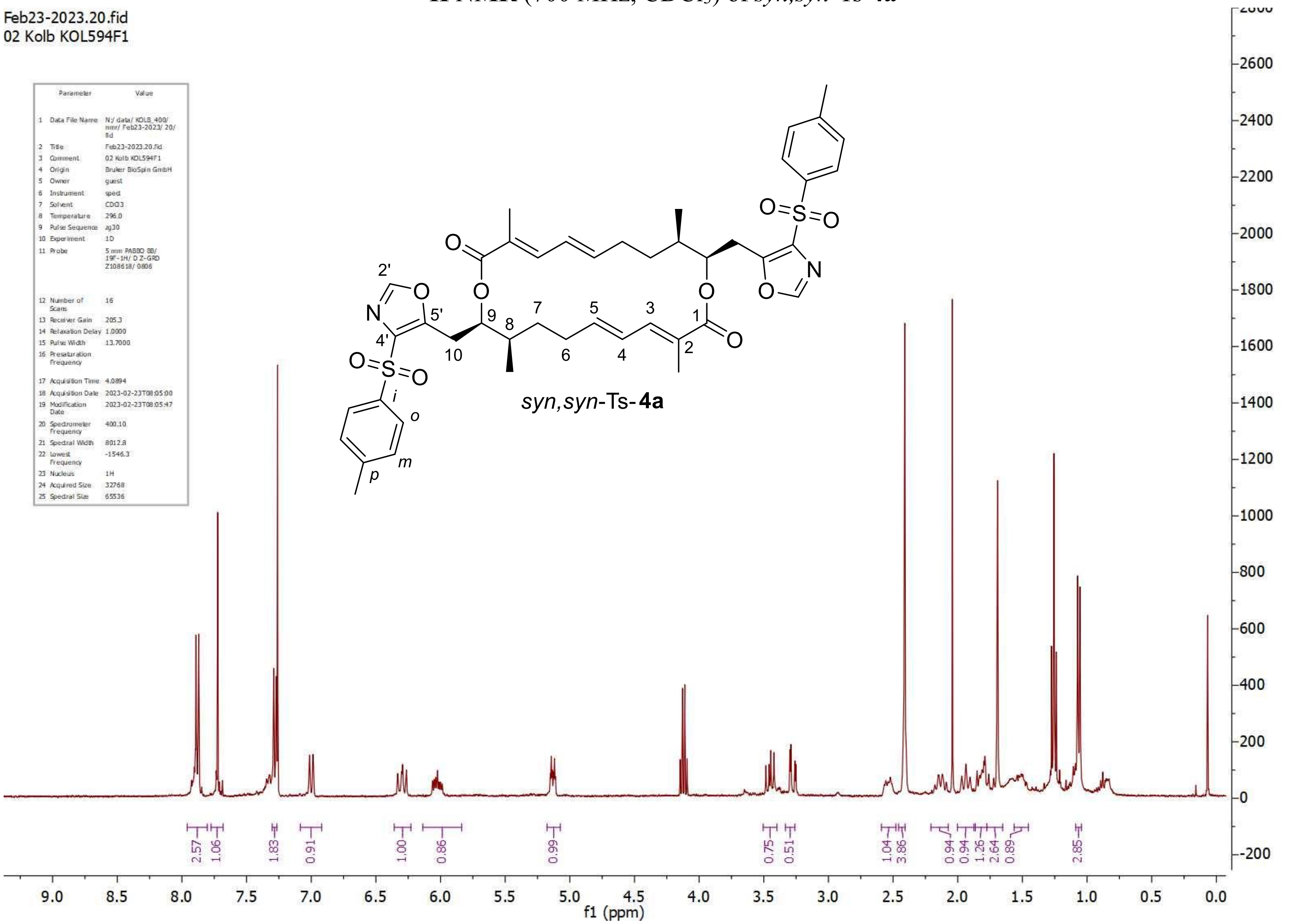

^{13}C NMR (176 MHz, $CDCl_3$) of *syn,syn*-Ts-**4a**

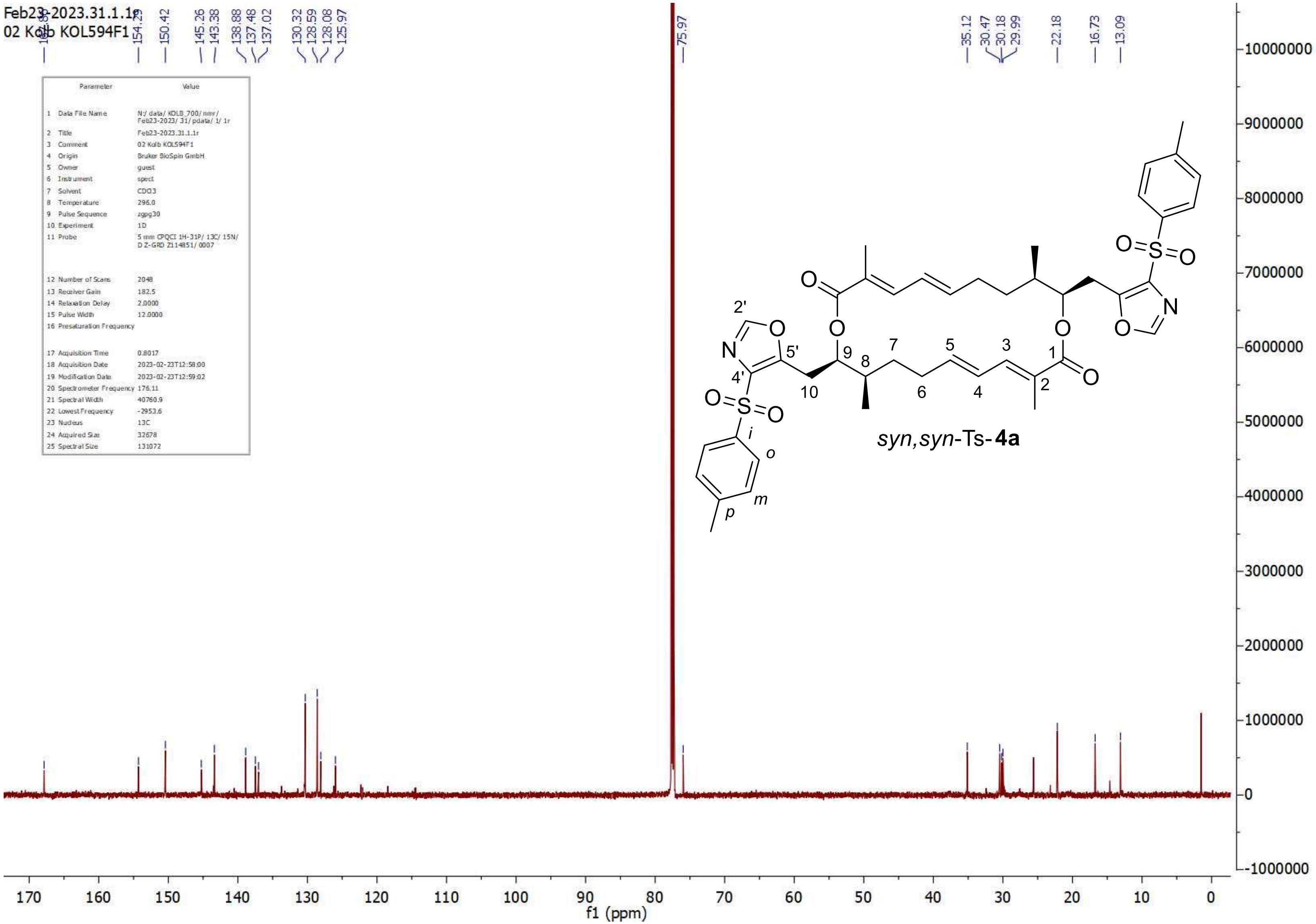

Parameter	Value
1 Data File Name	N:/ data/ KOLB_700/ nmr/ Feb23-2023/ 31/ pdata/ 1/ 1r
2 Title	Feb23-2023.31.1.1r
3 Comment	02 Kolb KOL594F1
4 Origin	Bruker BioSpin GmbH
5 Owner	guest
6 Instrument	spect
7 Solvent	CDCl3
8 Temperature	296.0
9 Pulse Sequence	zgpg30
10 Experiment	1D
11 Probe	5 mm CPQCI 1H-31P/ 13C/ 15N/ D Z-GRD Z114851/ 0007
12 Number of Scans	2048
13 Receiver Gain	182.5
14 Relaxation Delay	2.0000
15 Pulse Width	12.0000
16 Presaturation Frequency	
17 Acquisition Time	0.8017
18 Acquisition Date	2023-02-23T12:58:00
19 Modification Date	2023-02-23T12:59:02
20 Spectrometer Frequency	176.11
21 Spectral Width	40760.9
22 Lowest Frequency	-2953.6
23 Nucleus	13C
24 Acquired Size	32678
25 Spectral Size	131072

MS (ESI) of *syn,syn*-Ts-**4a**

Kolb

Massenspektrometrie - Universität Stuttgart

Analysis Info

Analysis Name	C:\Data\2023\laschat_2023\Kolb-KOL594F1_8_01_974.d	Acquisition Date	2/24/2023 12:51:57 PM
Method	tune-wide-oktober-2019.m	Operator	BDAL@DE
Sample Name	Kolb-KOL594F1	Instrument	micrOTOF-Q 228888.00043
Comment			

Acquisition Parameter

Source Type	ESI	Ion Polarity	Positive	Set Nebulizer	0.4 Bar
Focus	Not active	Set Capillary	4500 V	Set Dry Heater	200 °C
Scan Begin	250 m/z	Set End Plate Offset	-500 V	Set Dry Gas	4.0 l/min
Scan End	3000 m/z	Set Collision Cell RF	500.0 Vpp	Set Divert Valve	Waste

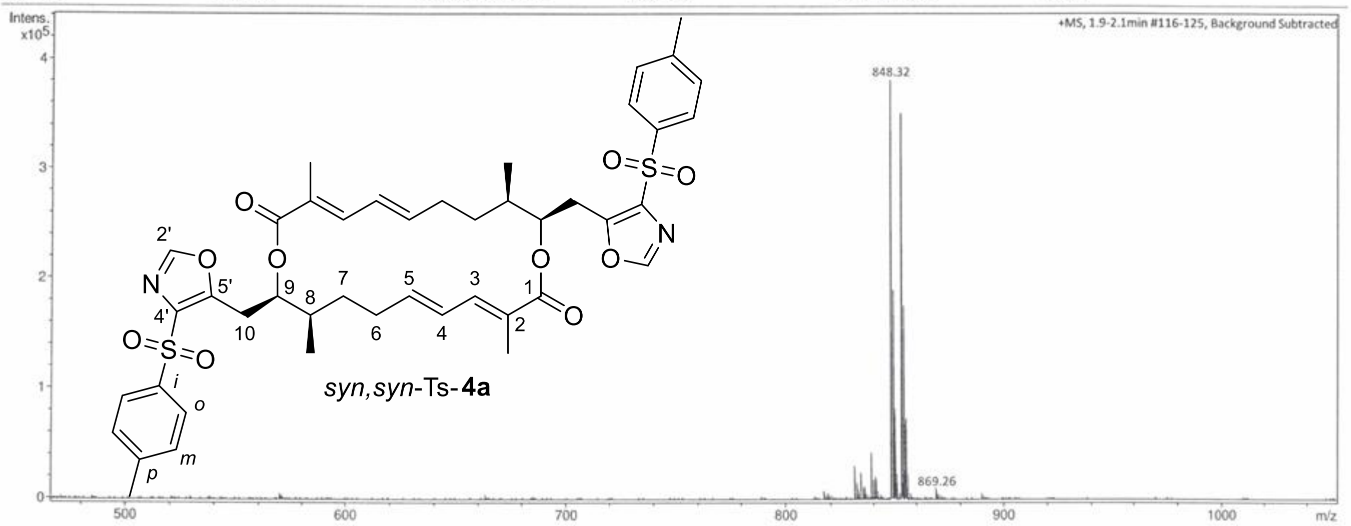

Bruker Compass DataAnalysis 4.2 printed: 2/24/2023 1:08:39 PM by: BDAL@DE Page 1 of 1

HRMS of *syn,syn*-Ts-**4a**

Massenspektrometrie - Universität Stuttgart

Analysis Info | Acquisition Date 2/24/2023 12:51:57 PM

Analysis Name	C:\Data\2023\laschat_2023\Kolb-KOL594F1_8_01_974.d		
Method	tune-wide-oktober-2019.m	Operator	BDAL@DE
Sample Name	Kolb-KOL594F1	Instrument	micrOTOF-Q 228888.00043
Comment			

Acquisition Parameter

Source Type	ESI	Ion Polarity	Positive	Set Nebulizer	0.4 Bar
Focus	Not active	Set Capillary	4500 V	Set Dry Heater	200 °C
Scan Begin	250 m/z	Set End Plate Offset	-500 V	Set Dry Gas	4.0 l/min
Scan End	3000 m/z	Set Collision Cell RF	500.0 Vpp	Set Divert Valve	Waste

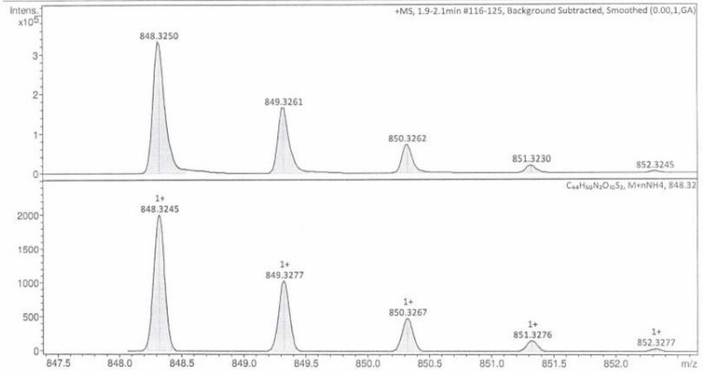

Bruker Compass DataAnalysis 4.2 printed: 2/24/2023 1:11:22 PM by: BDAL@DE Page 1 of 1

^{1}**H NMR** (700 MHz, $CDCl_3$) of *anti,anti*-Ts-**4a**

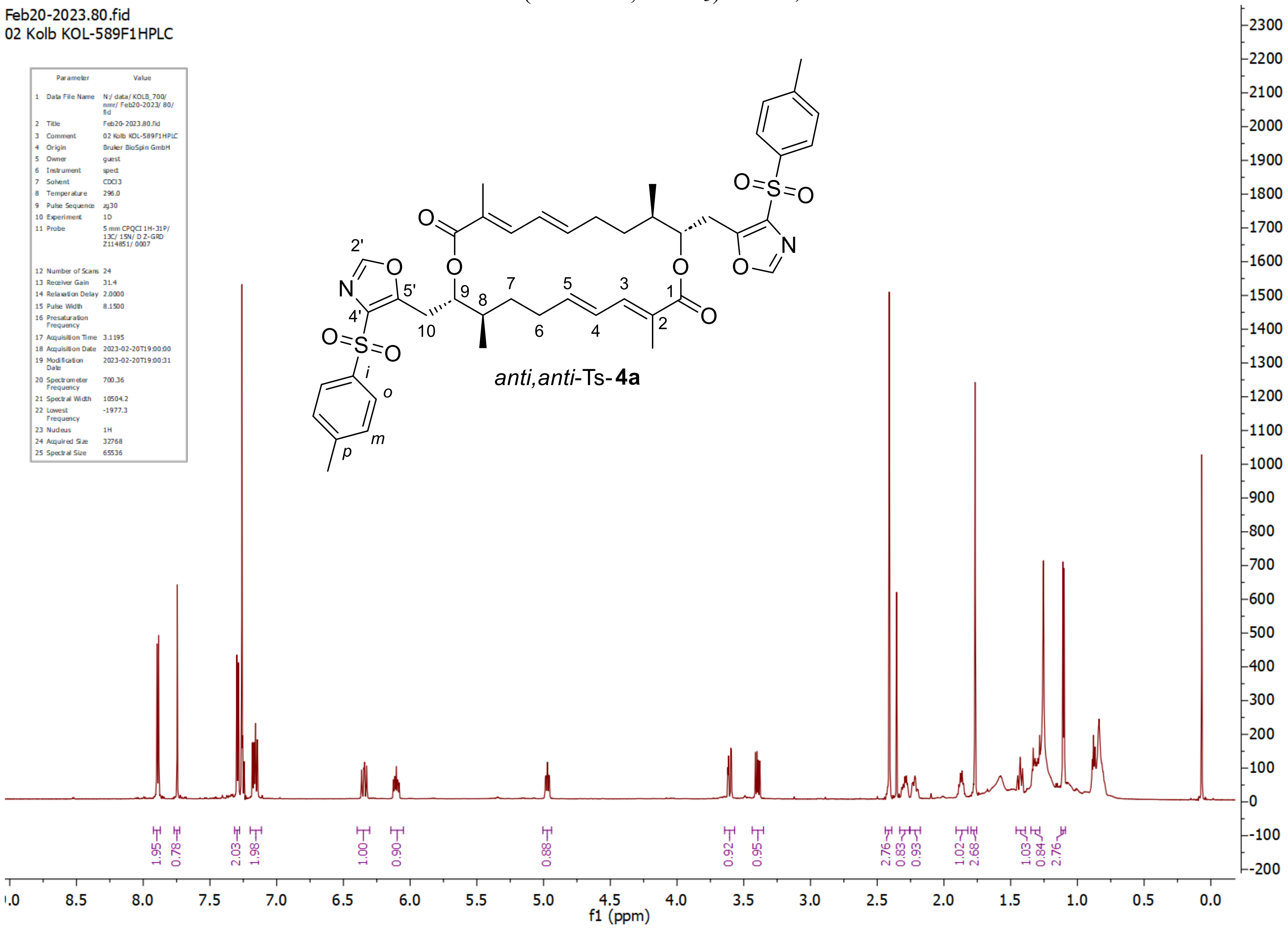

^{13}C NMR (176 MHz, $CDCl_3$) of *anti,anti*-Ts-**4a**

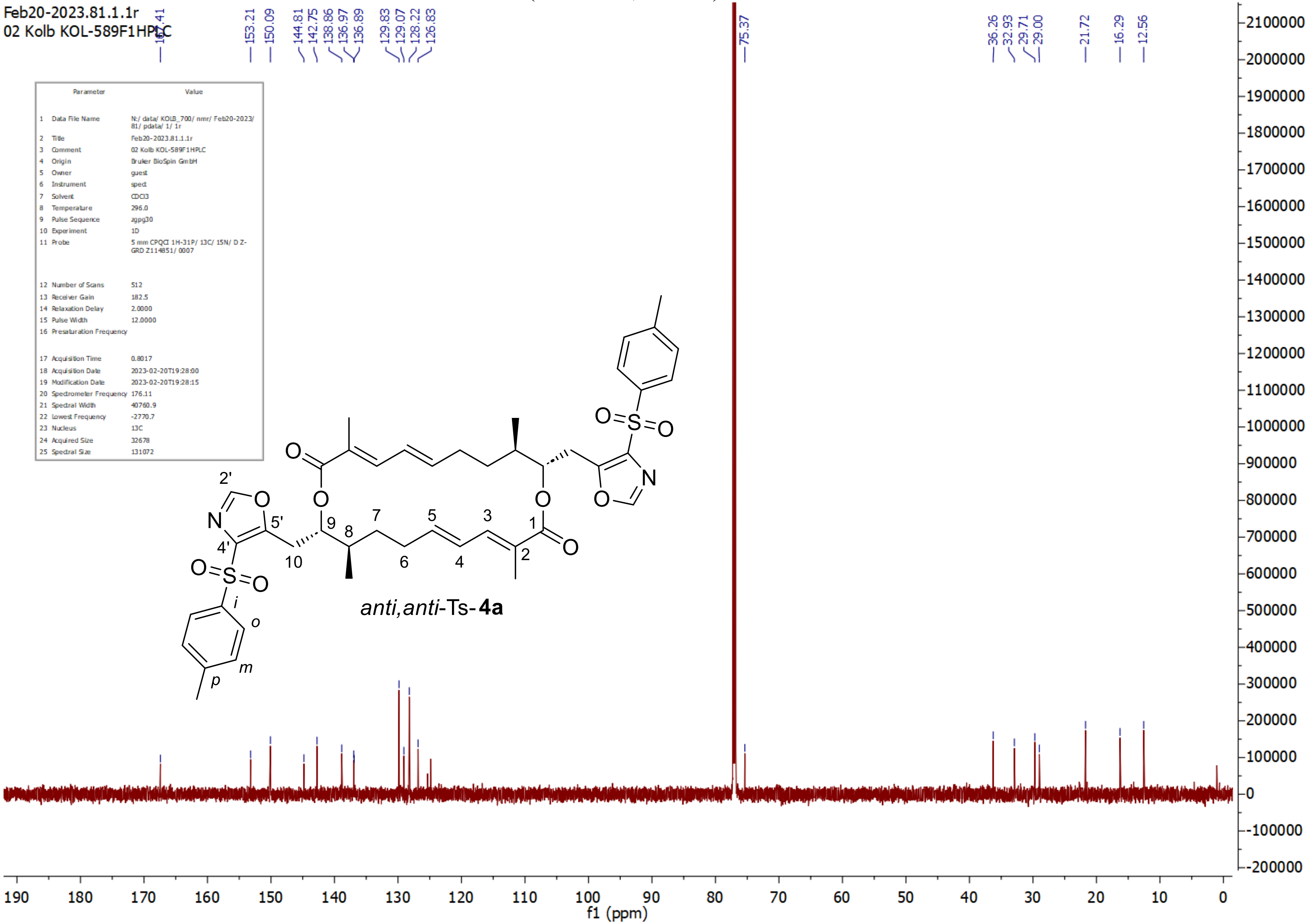

MS (ESI) of *anti,anti*-Ts-**4a**

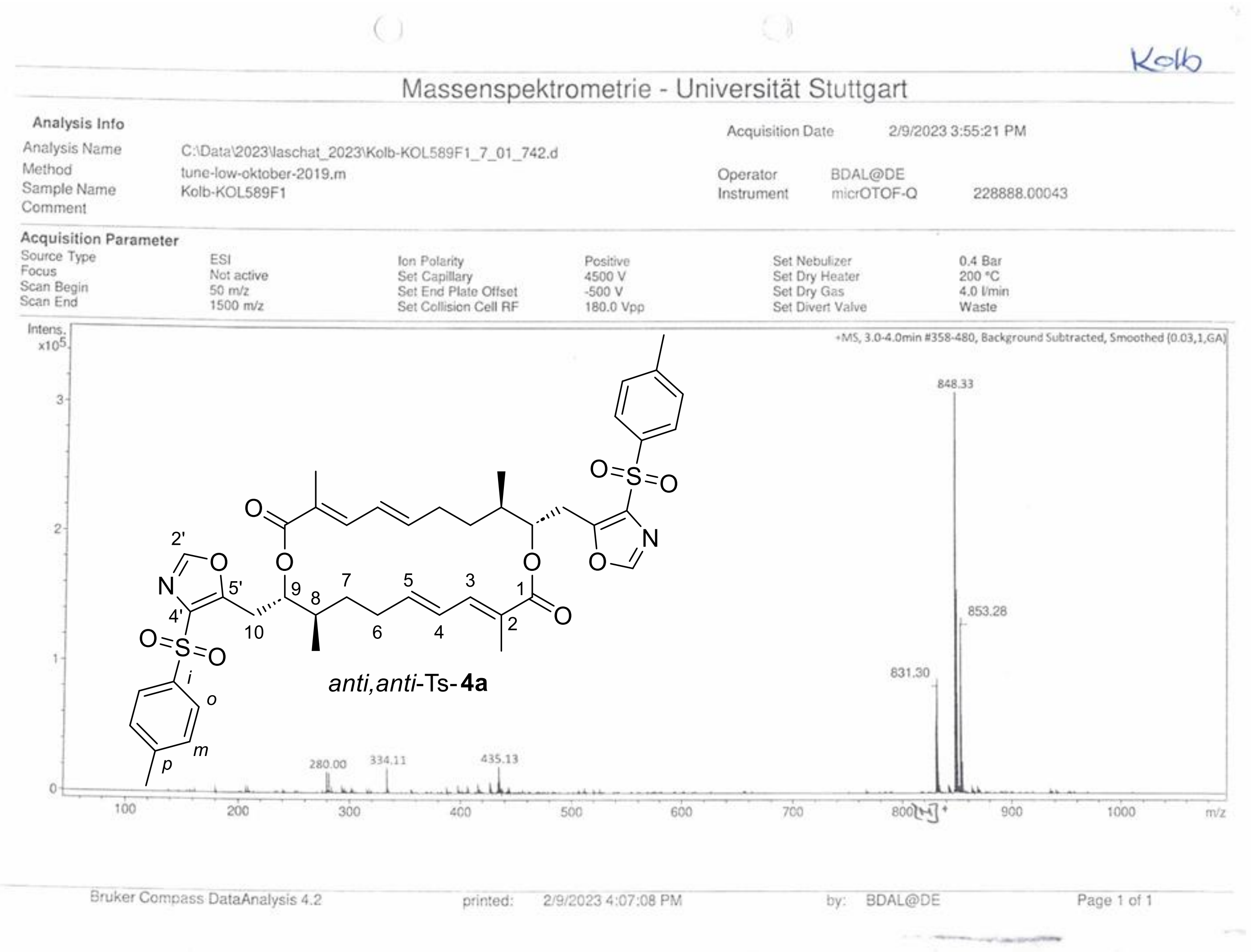

HRMS of *anti,anti*-Ts-**4a**

Massenspektrometrie - Universität Stuttgart

Analysis Info

Acquisition Date 2/9/2023 3:55:21 PM

Analysis Name C:\Data\2023\laschat_2023\Kolb-KOL589F1_7_01_742.d
Method tune-low-oktober-2019.m
Sample Name Kolb-KOL589F1
Comment

Operator BDAL@DE
Instrument micrOTOF-Q 228888.00043

Acquisition Parameter

Source Type	ESI	Ion Polarity	Positive	Set Nebulizer	0.4 Bar
Focus	Not active	Set Capillary	4500 V	Set Dry Heater	200 °C
Scan Begin	50 m/z	Set End Plate Offset	-500 V	Set Dry Gas	4.0 l/min
Scan End	1500 m/z	Set Collision Cell RF	180.0 Vpp	Set Divert Valve	Waste

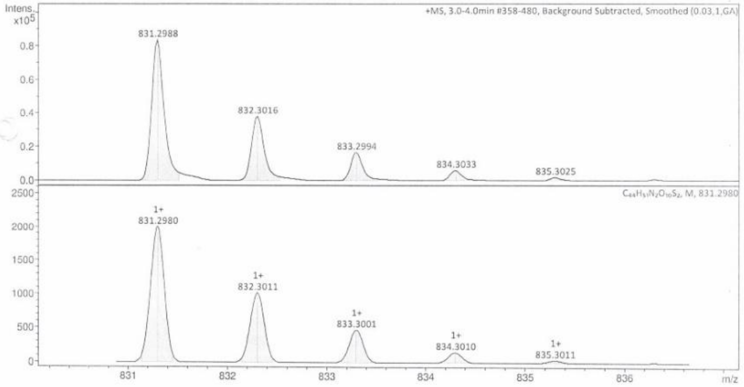

Bruker Compass DataAnalysis 4.2 printed: 2/9/2023 4:06:22 PM by: BDAL@DE Page 1 of 1

^{1}H NMR (400 MHz, $CDCl_3$) of **19**

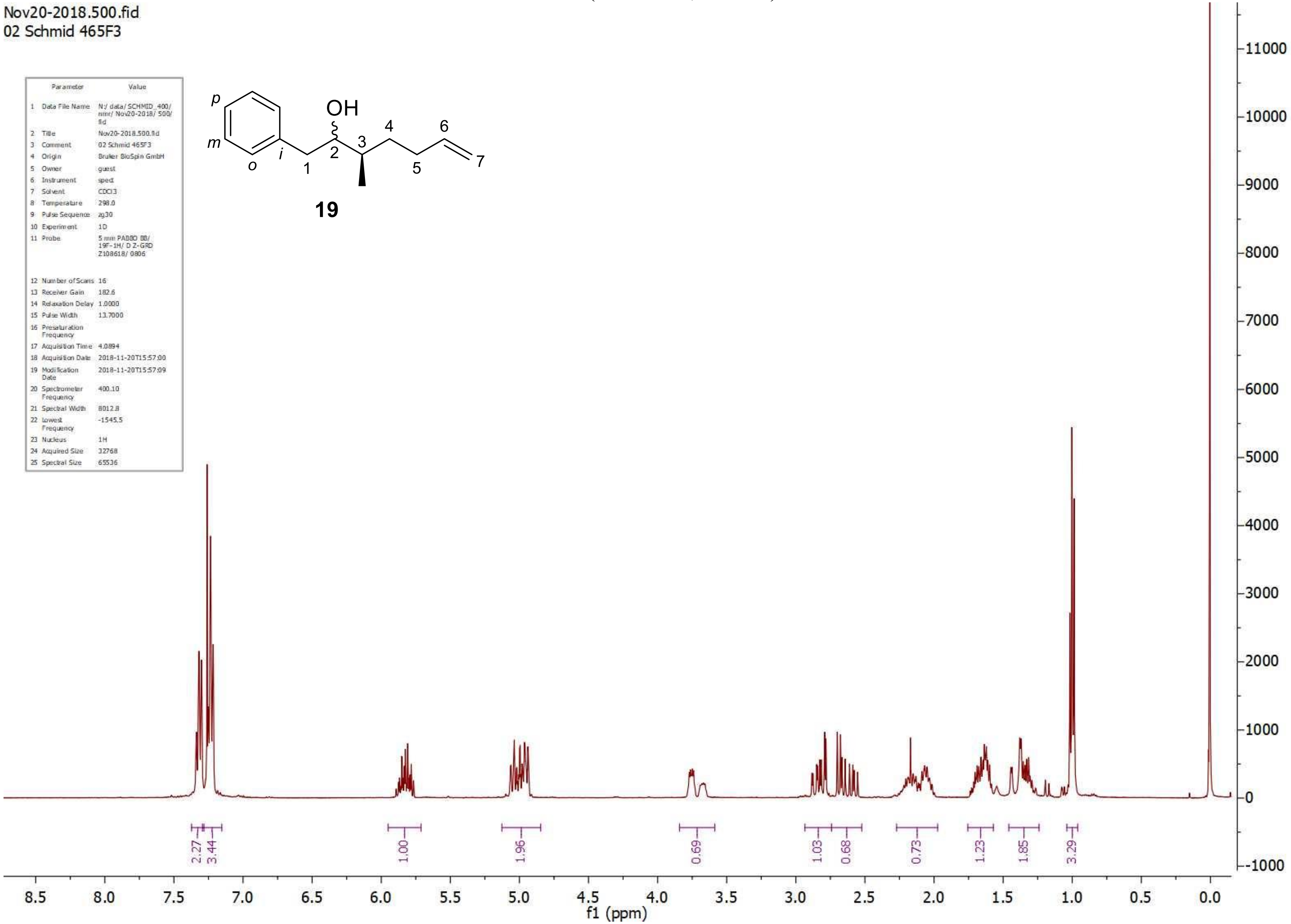

^{13}C NMR (100 MHz, $CDCl_3$) of **19**

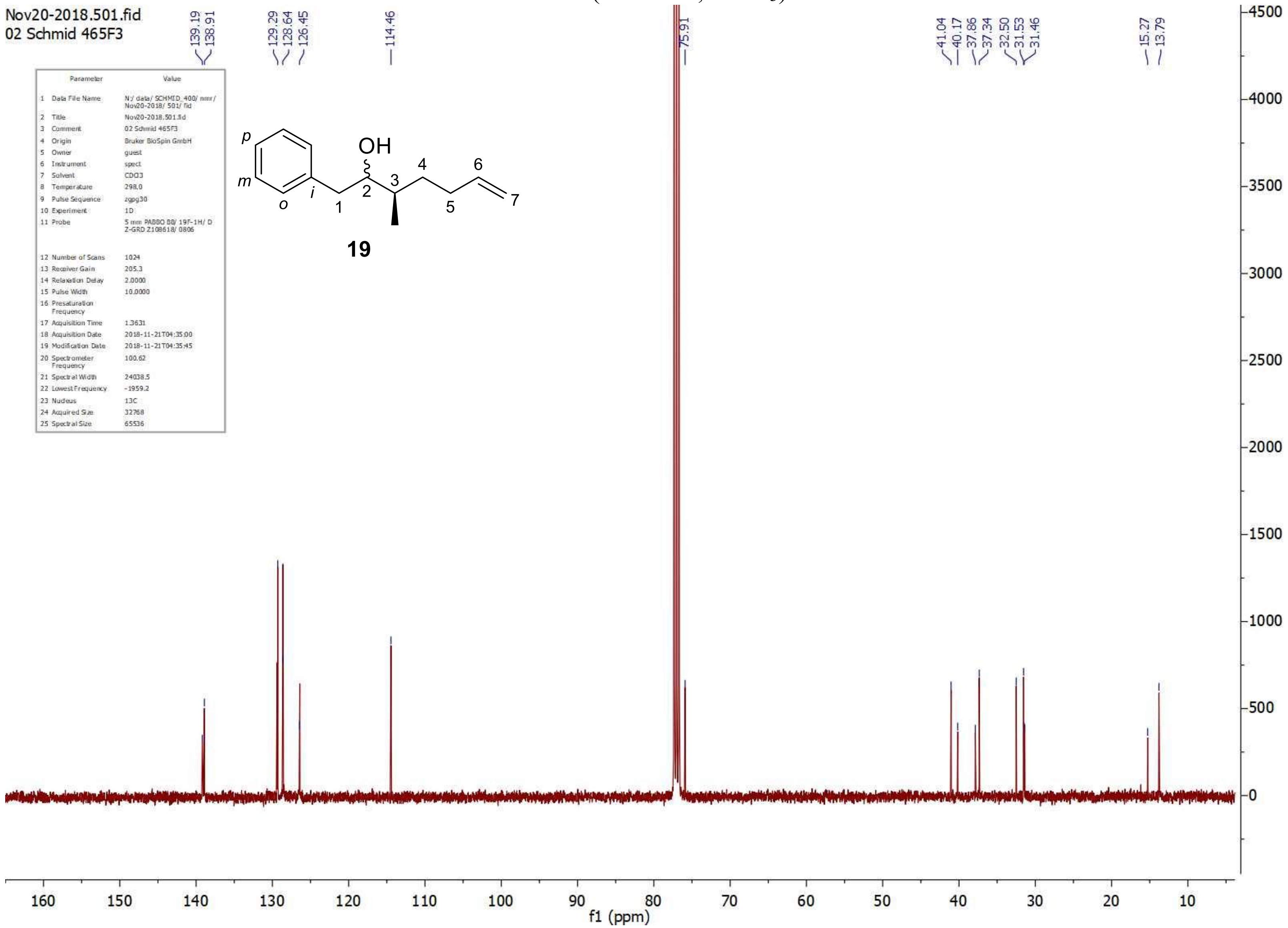

^{1}H NMR (700 MHz, $CDCl_3$) of **24a**

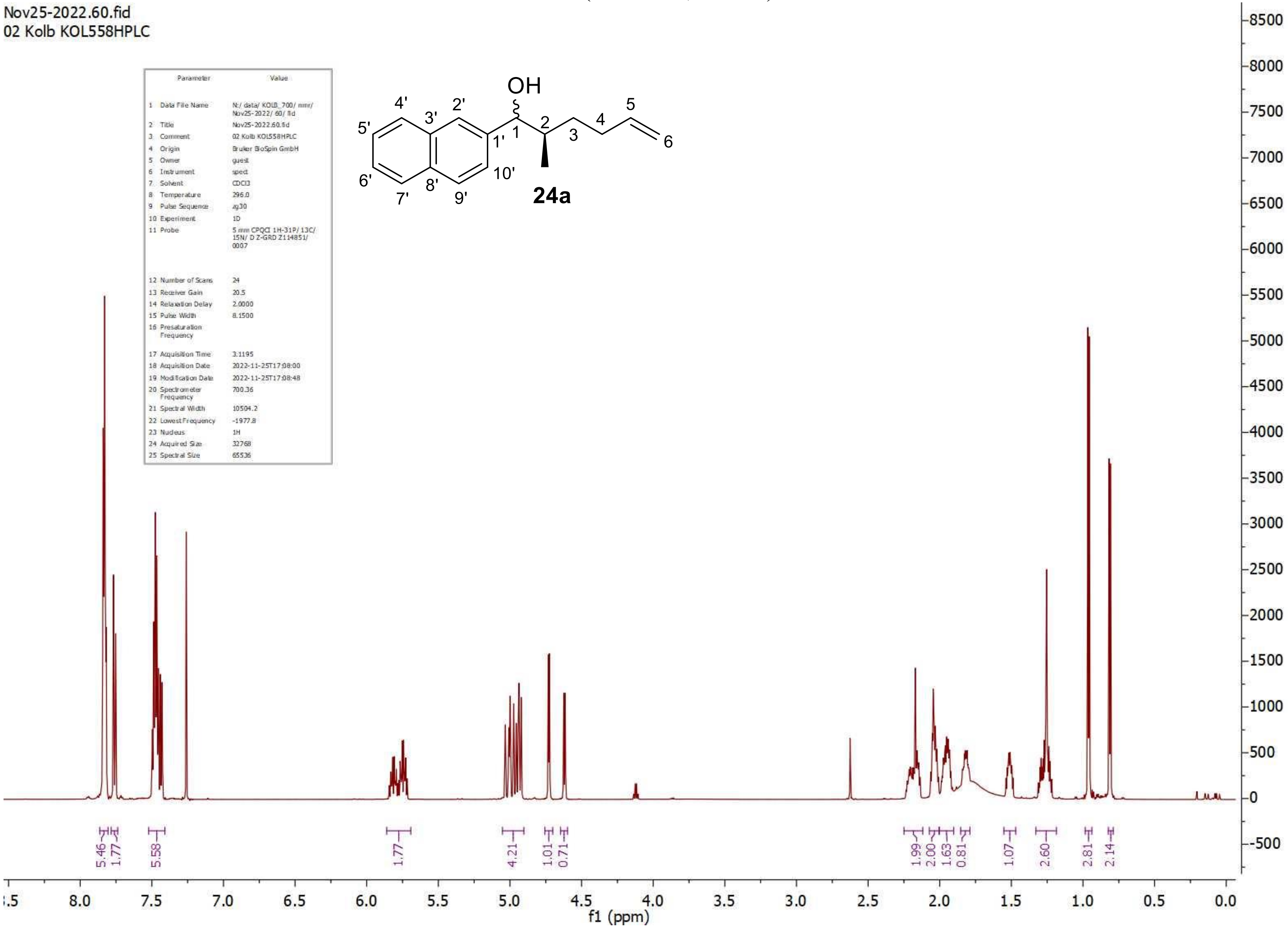

^{13}**C NMR** (176 MHz, $CDCl_3$) of **24a**

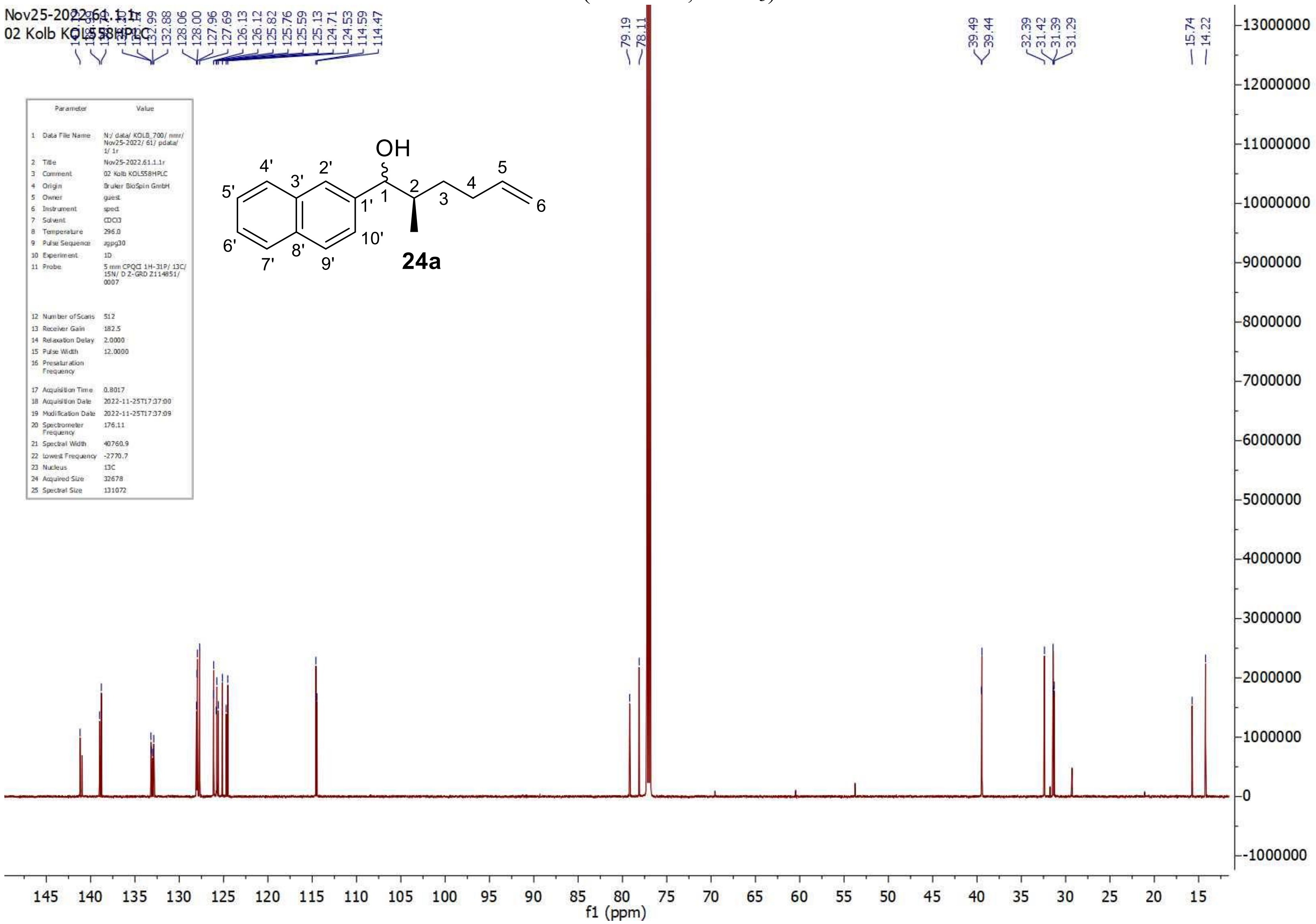

^{1}H NMR (700 MHz, $CDCl_3$) of *syn*-24b

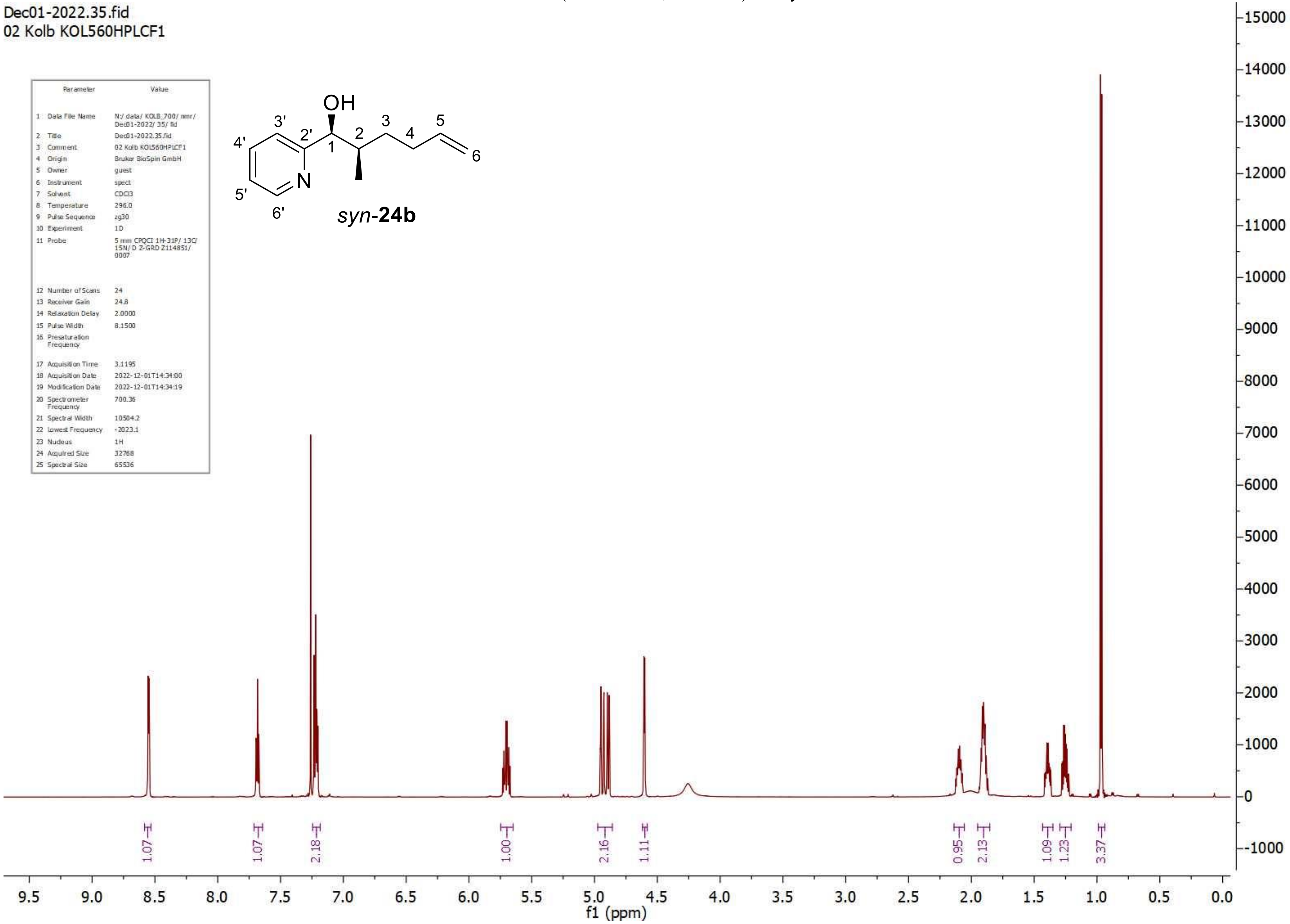

^{13}C NMR (176 MHz, $CDCl_3$) of *syn*-**24b**

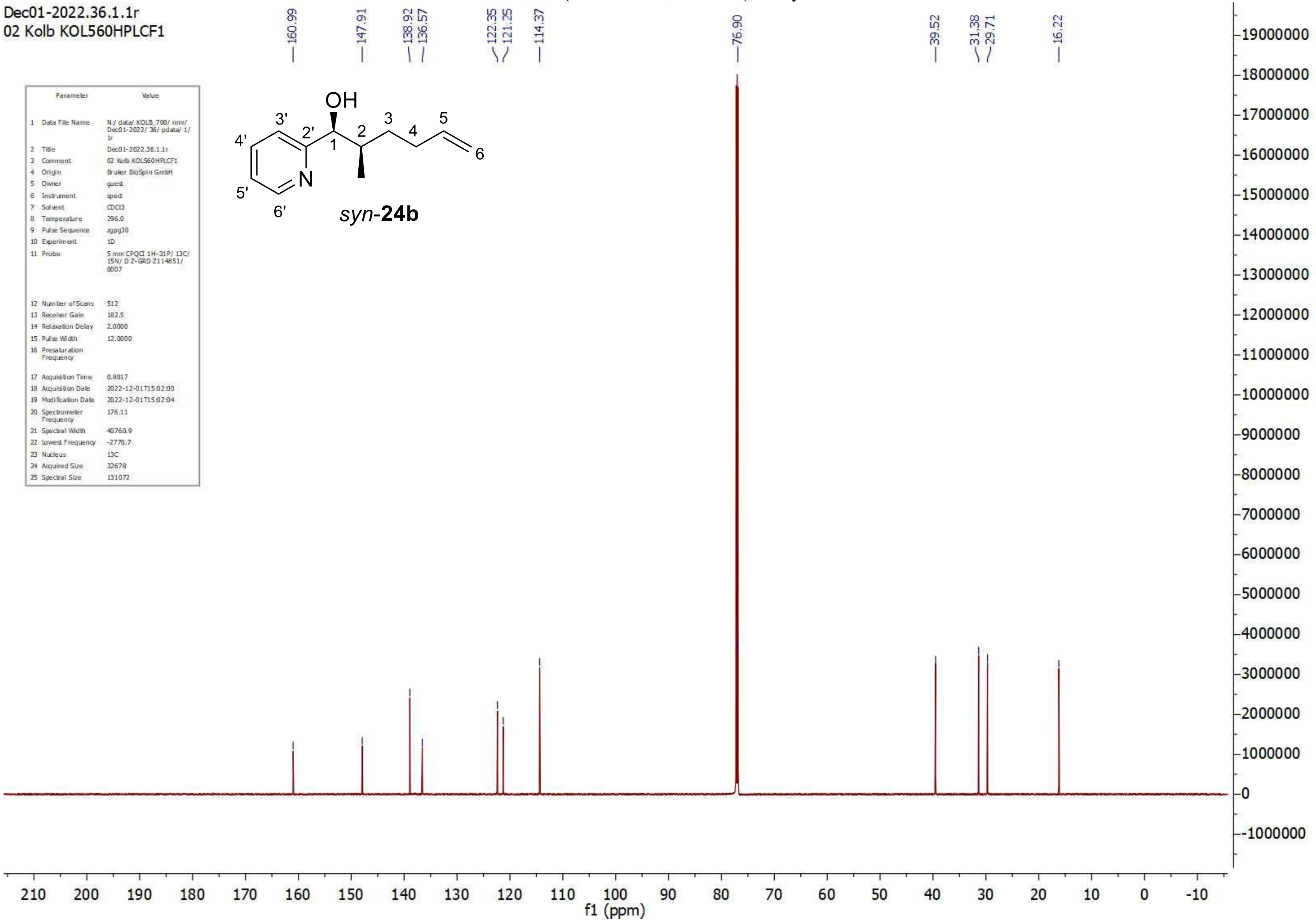

^{1}H NMR (700 MHz, $CDCl_3$) of *anti*-**24b**

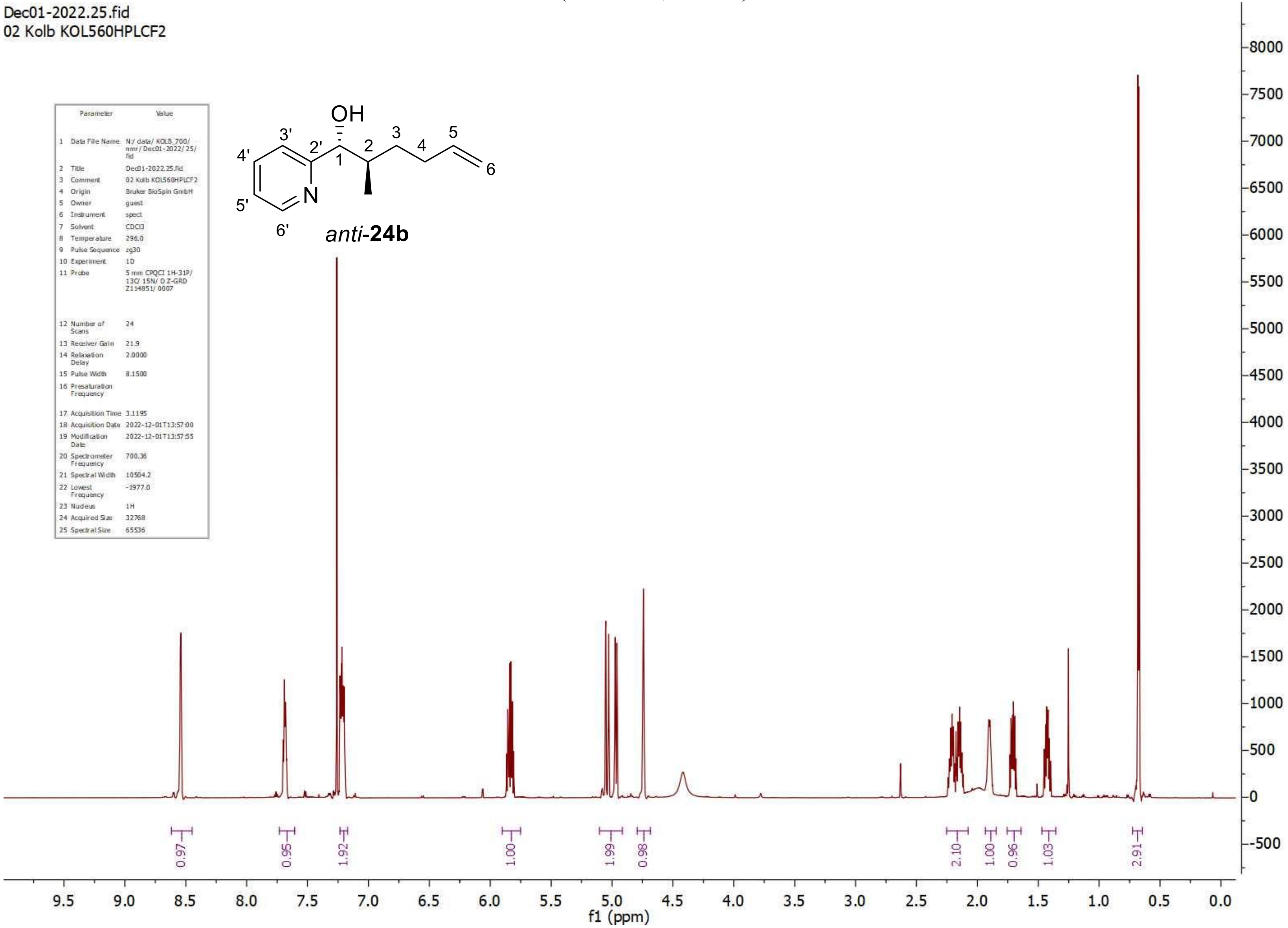

^{13}C NMR (176 MHz, $CDCl_3$) of *anti*-**24b**

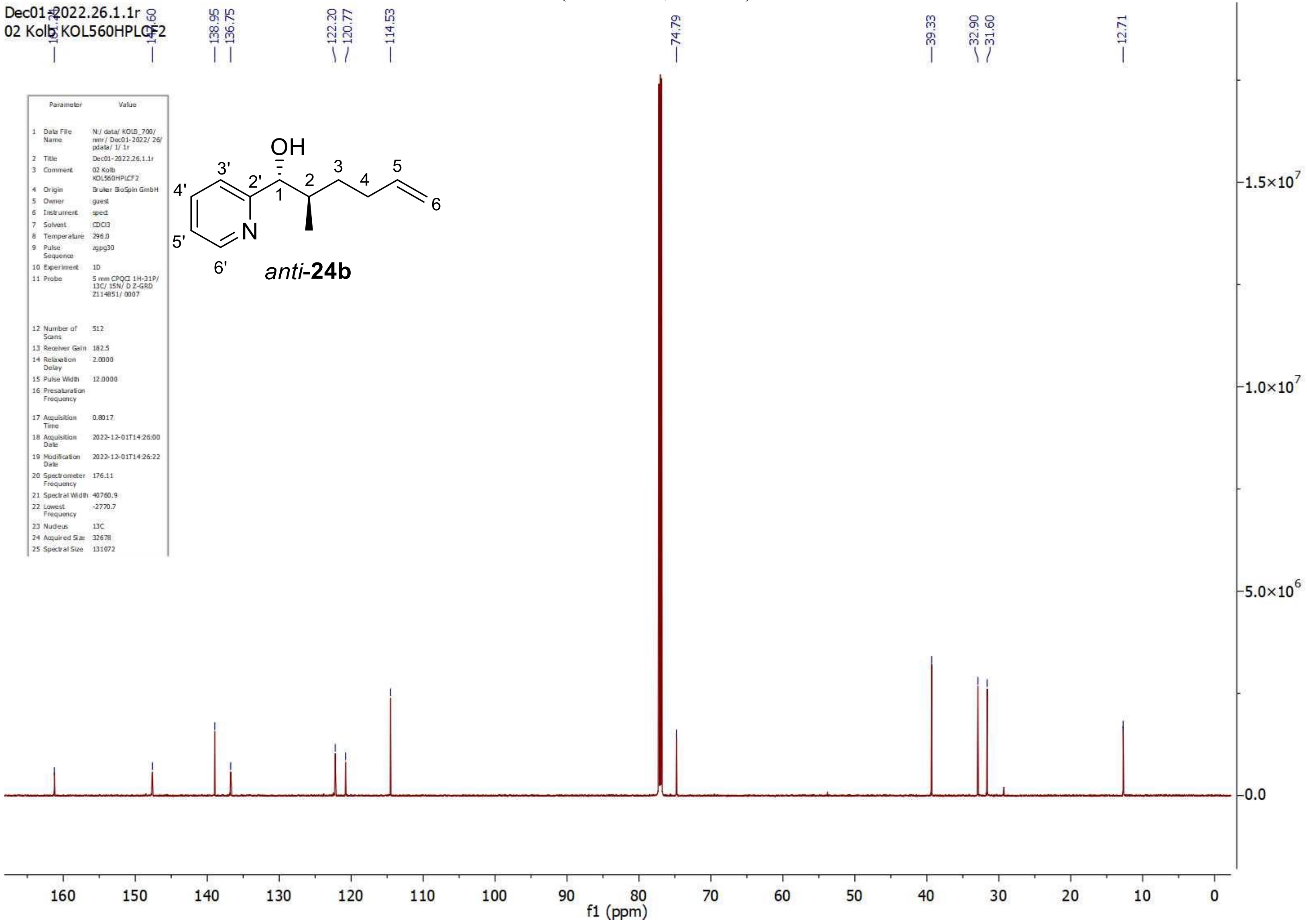

^{1}H NMR (700 MHz, $CDCl_3$) of **24c**

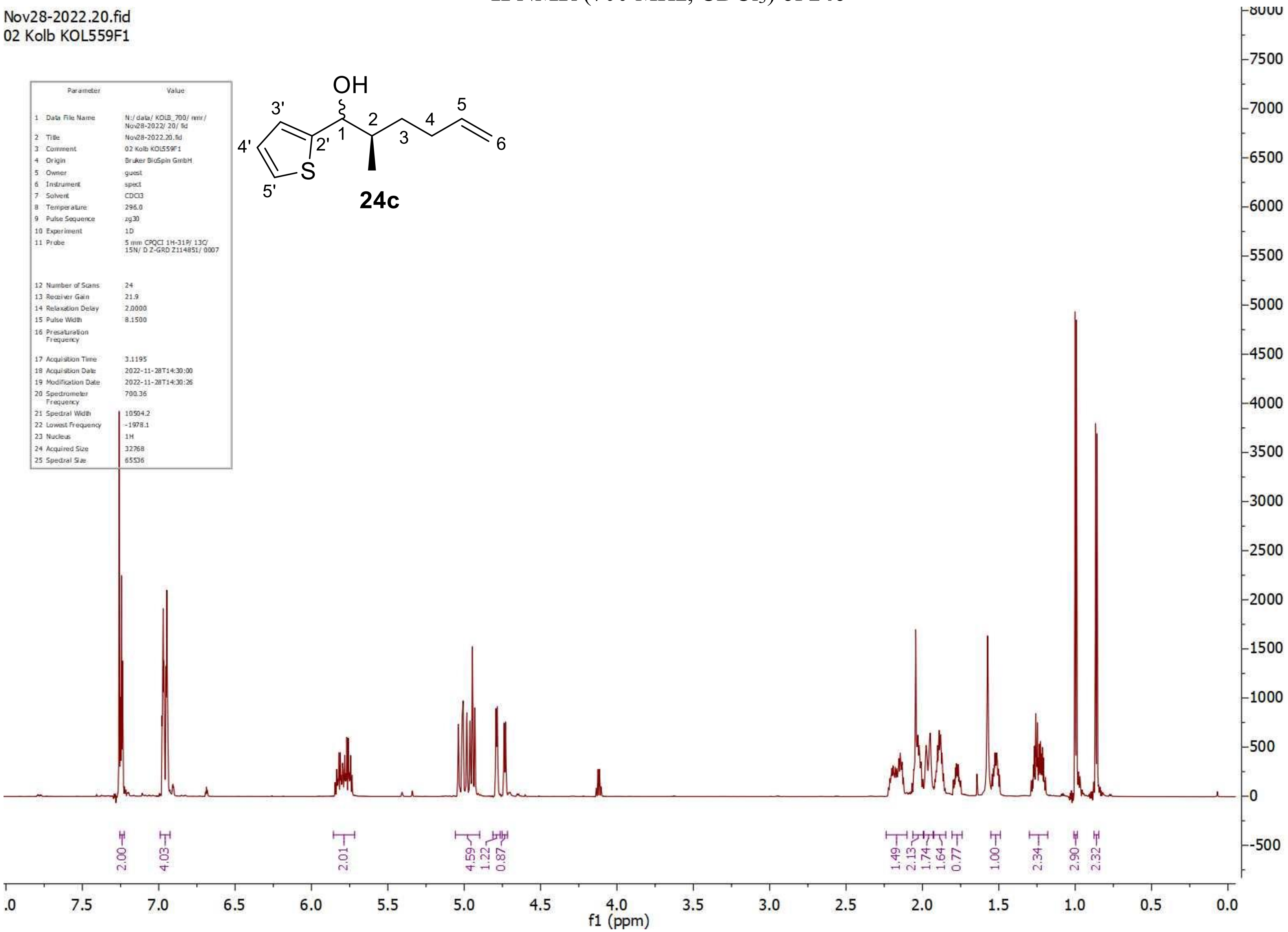

^{13}C NMR (176 MHz, $CDCl_3$) of *anti*-**24c**

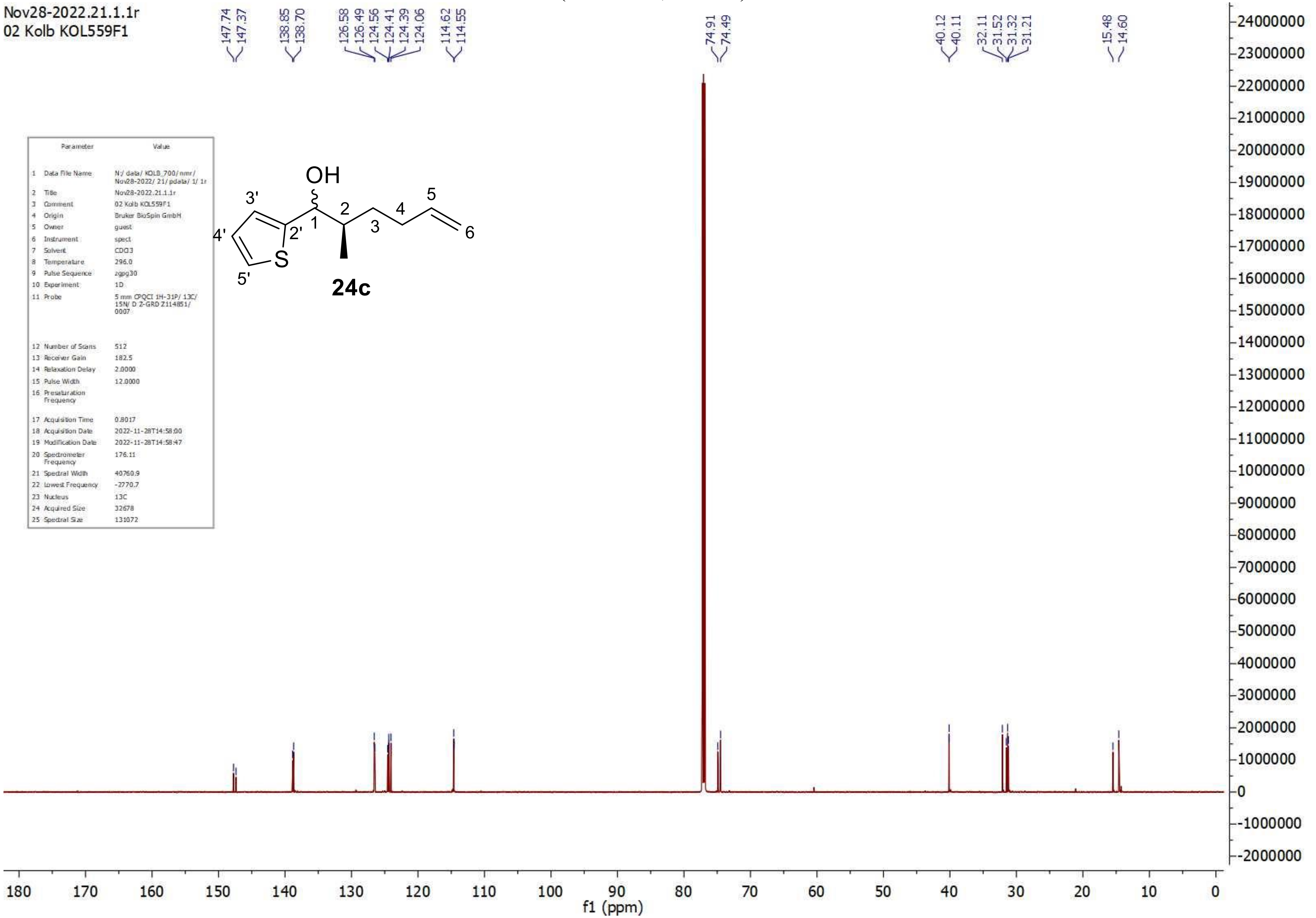

	Parameter	Value
1	Data File Name	N:/ data/ KOLB_700/ nmr/ Nov28-2022/ 21/ pdata/ 1/ 1r
2	Title	Nov28-2022.21.1.1r
3	Comment	02 Kolb KOL559F1
4	Origin	Bruker BioSpin GmbH
5	Owner	guest
6	Instrument	spect
7	Solvent	CDCl3
8	Temperature	296.0
9	Pulse Sequence	zgpg30
10	Experiment	1D
11	Probe	5 mm CPQCI 1H-31P/ 13C/ 15N/ D Z-GRD Z114851/ 0007
12	Number of Scans	512
13	Receiver Gain	182.5
14	Relaxation Delay	2.0000
15	Pulse Width	12.0000
16	Presaturation Frequency	
17	Acquisition Time	0.8017
18	Acquisition Date	2022-11-28T14:58:00
19	Modification Date	2022-11-28T14:58:47
20	Spectrometer Frequency	176.11
21	Spectral Width	40760.9
22	Lowest Frequency	-2770.7
23	Nucleus	13C
24	Acquired Size	32678
25	Spectral Size	131072

24c

^{1}H NMR (400 MHz, $CDCl_3$) of **25a**

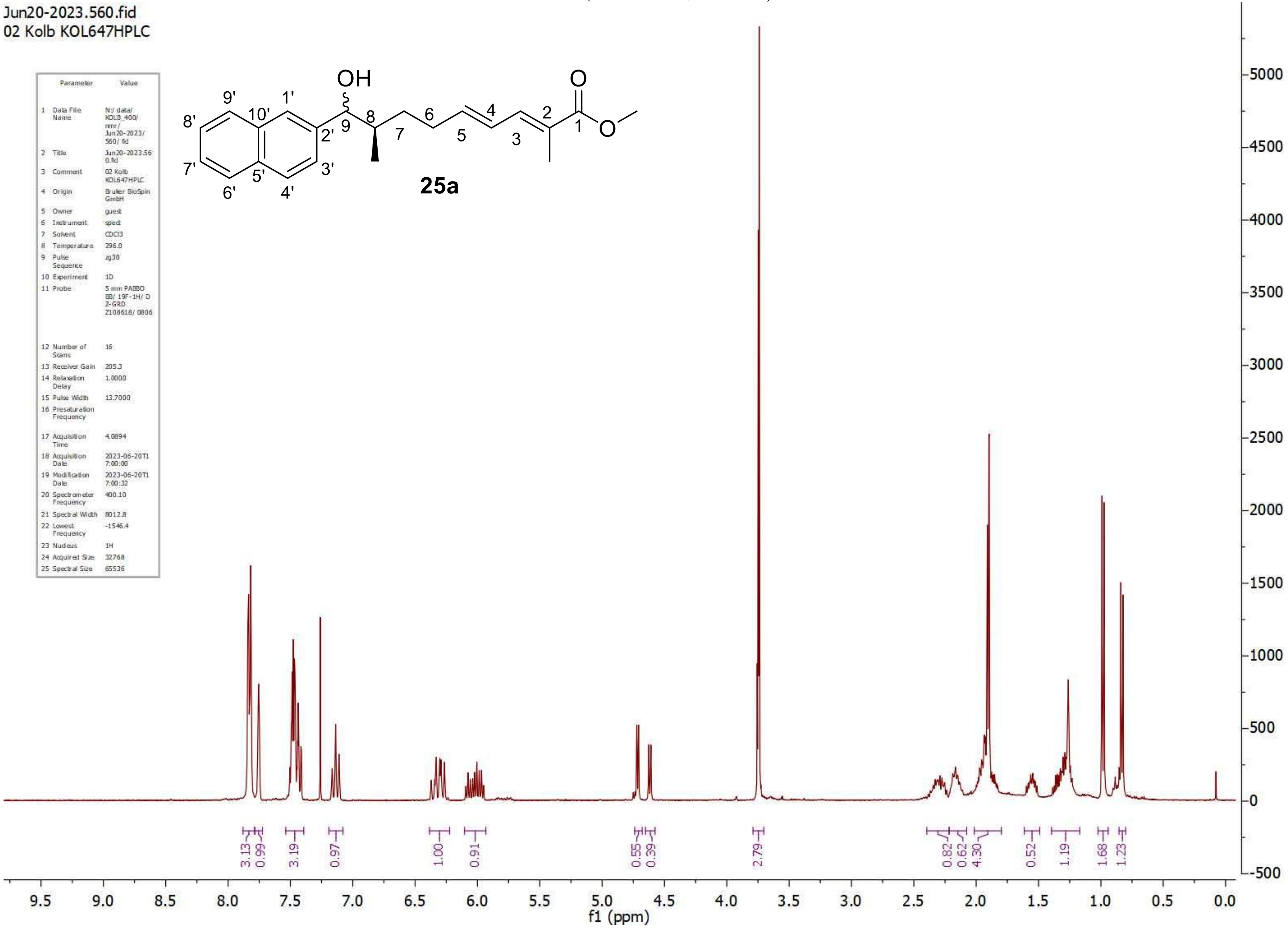

^{13}C NMR (100 MHz, $CDCl_3$) of **25a**

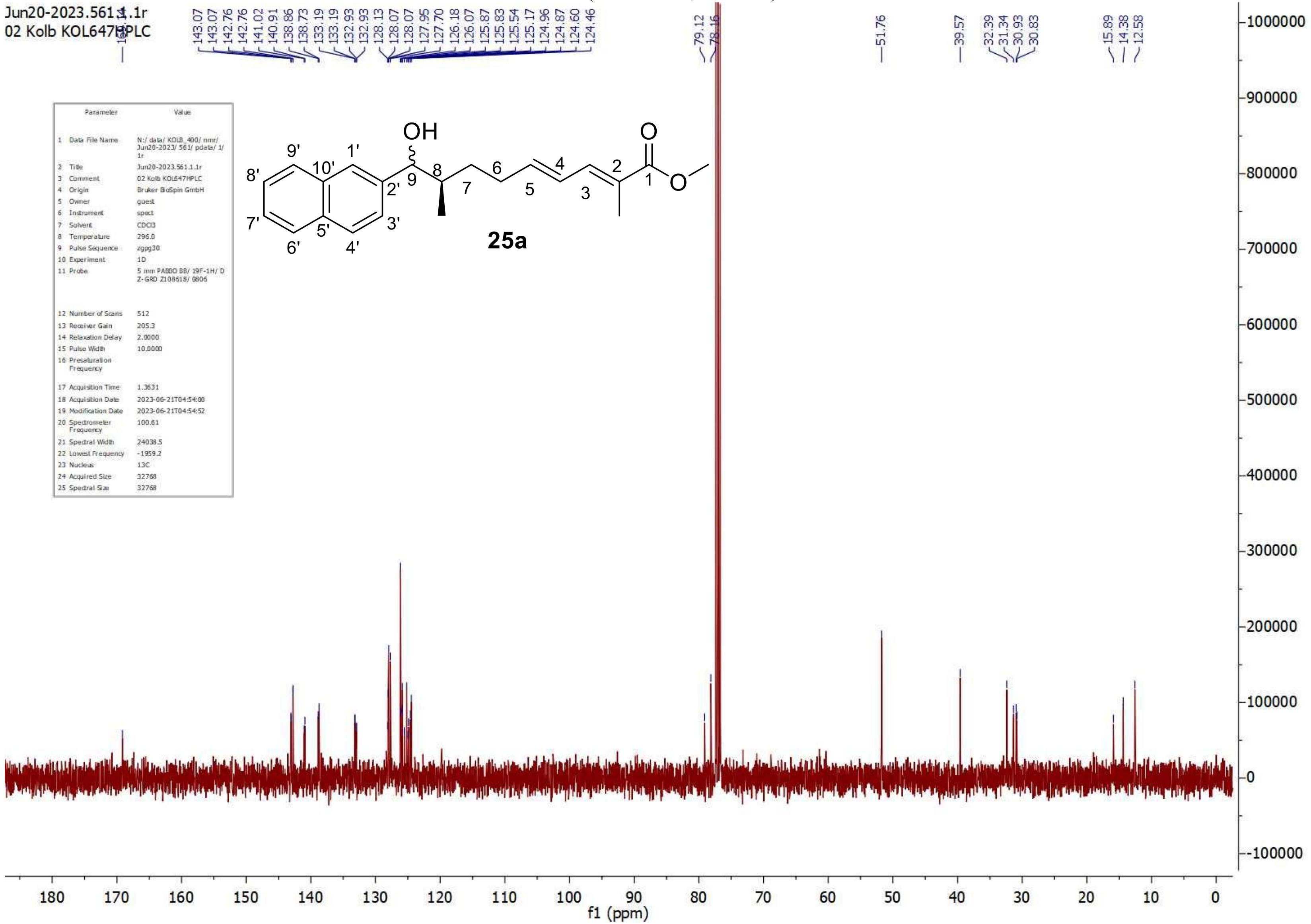

^{1}H NMR (700 MHz, $CDCl_3$) of *syn*-25b

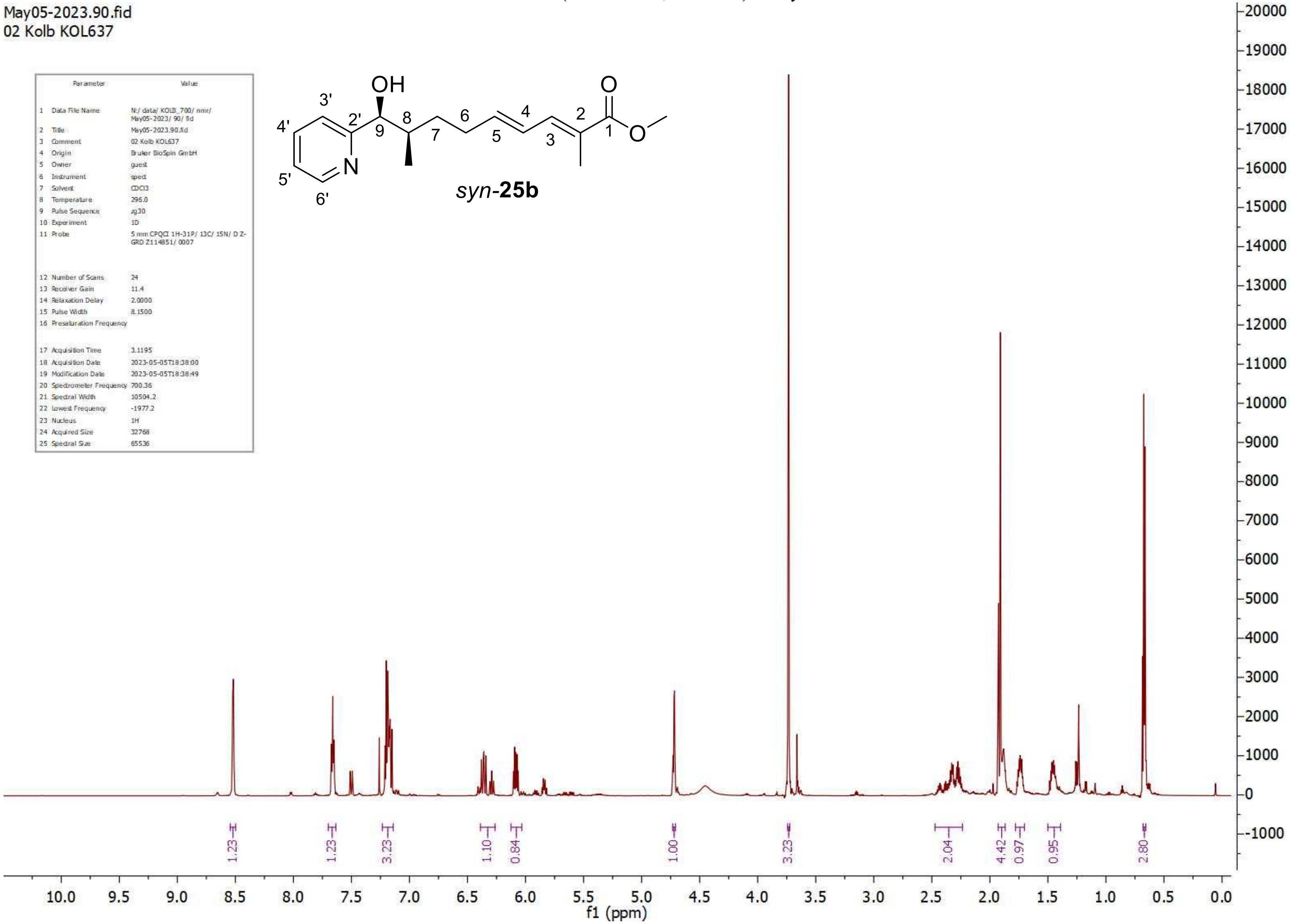

^{13}**C NMR** (100 MHz, $CDCl_3$) of *syn*-**25b**

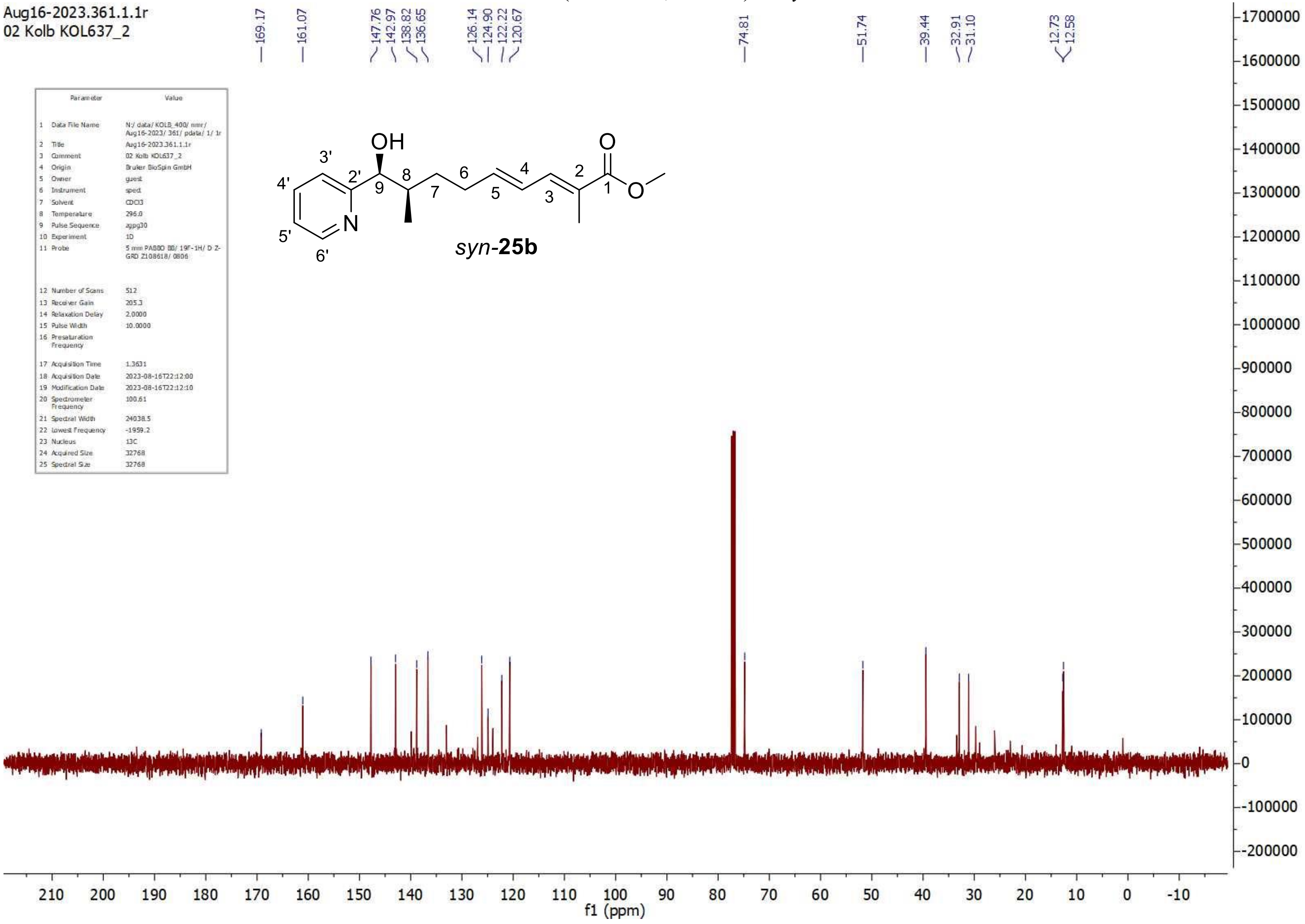

^{1}H NMR (700 MHz, CDCl$_3$) of *anti*-25b

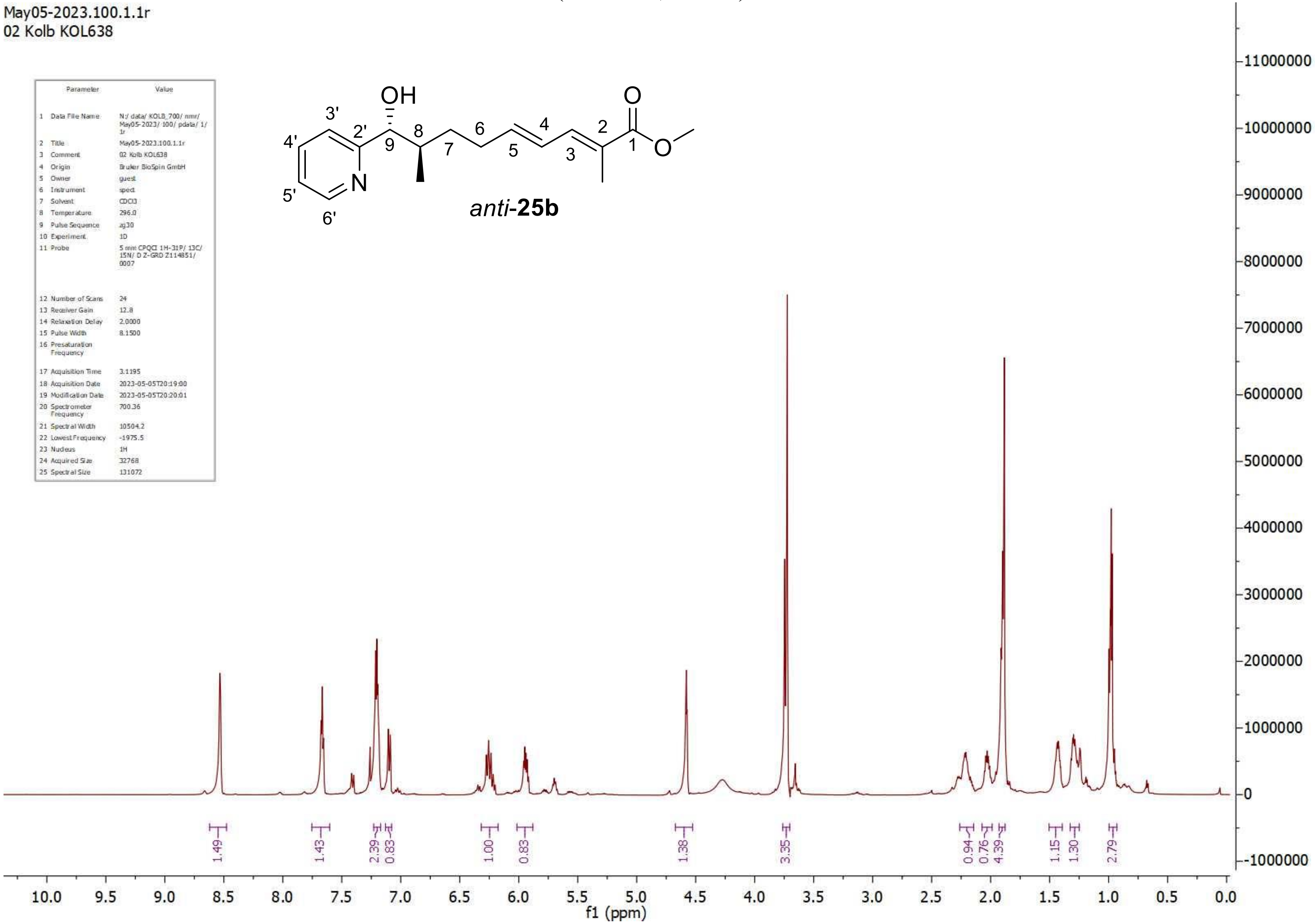

^{13}C NMR (100 MHz, $CDCl_3$) of *anti*-**25b**

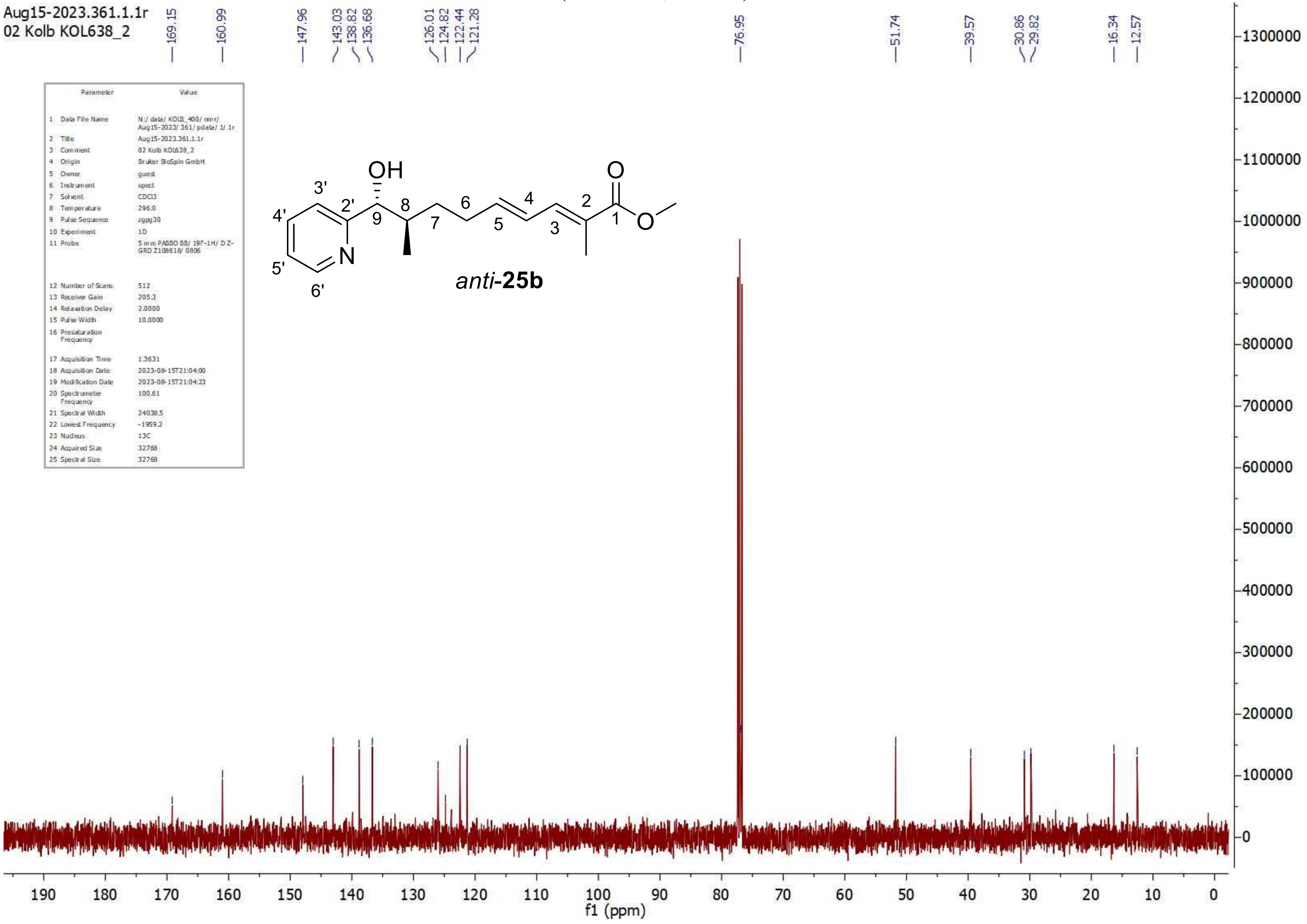

^{1}H NMR (400 MHz, $CDCl_3$) of **25c**

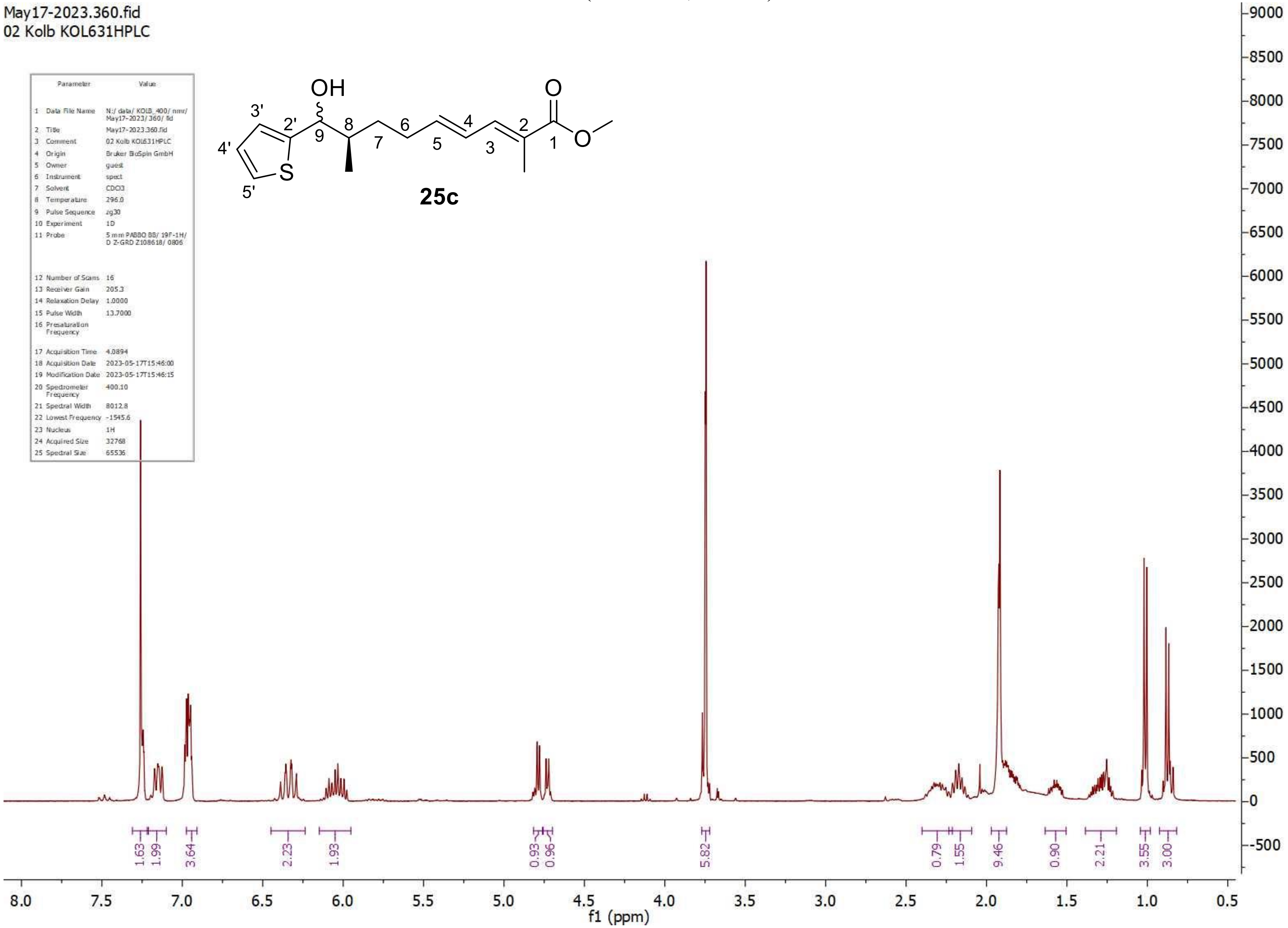

^{13}C NMR (100 MHz, $CDCl_3$) of **25c**

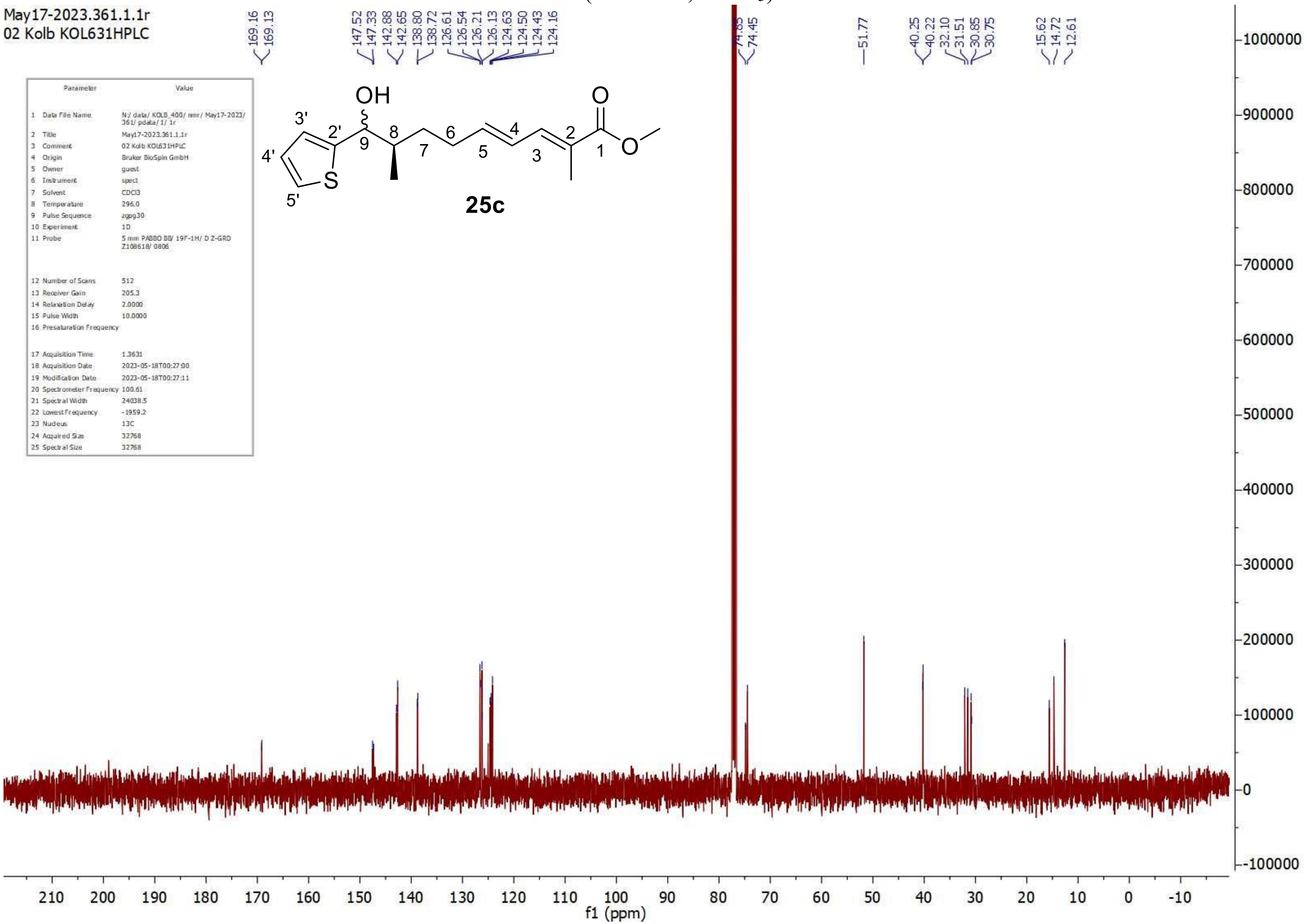

^{1}H NMR (500 MHz, $CDCl_3$) of **22a**

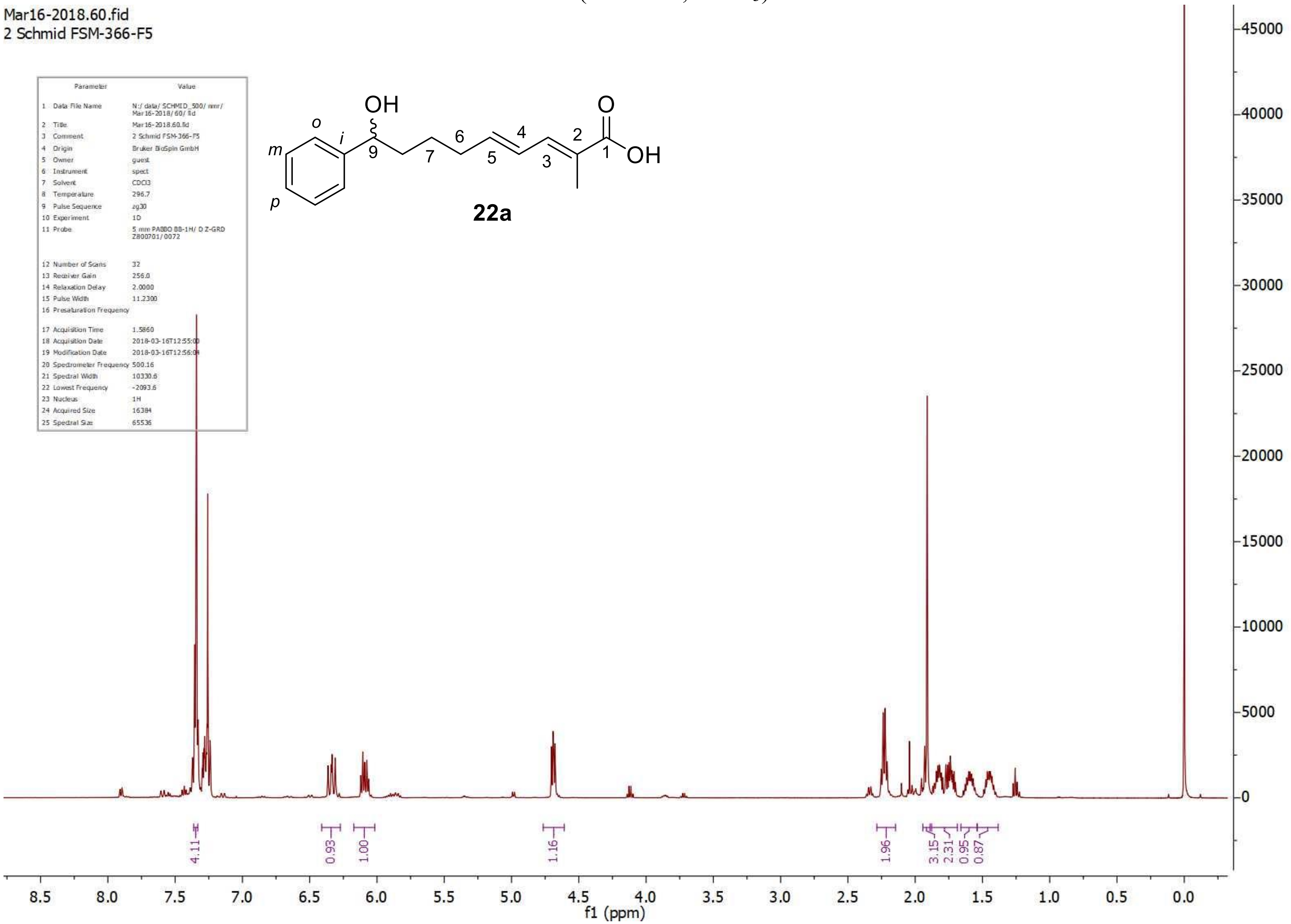

^{13}C **NMR** (125 MHz, $CDCl_3$) of **22a**

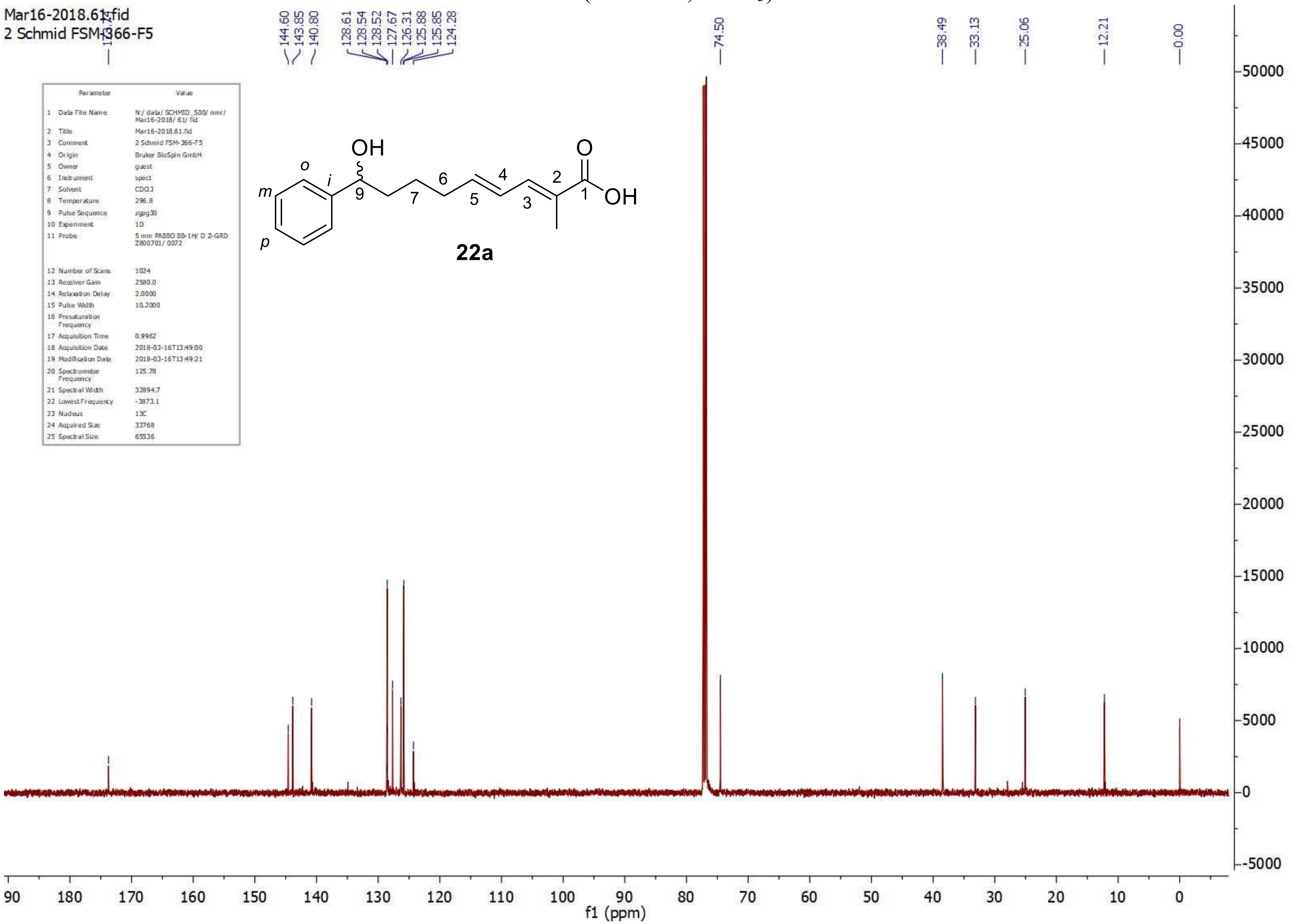

^{1}H NMR (400 MHz, $CDCl_3$) of **22b**

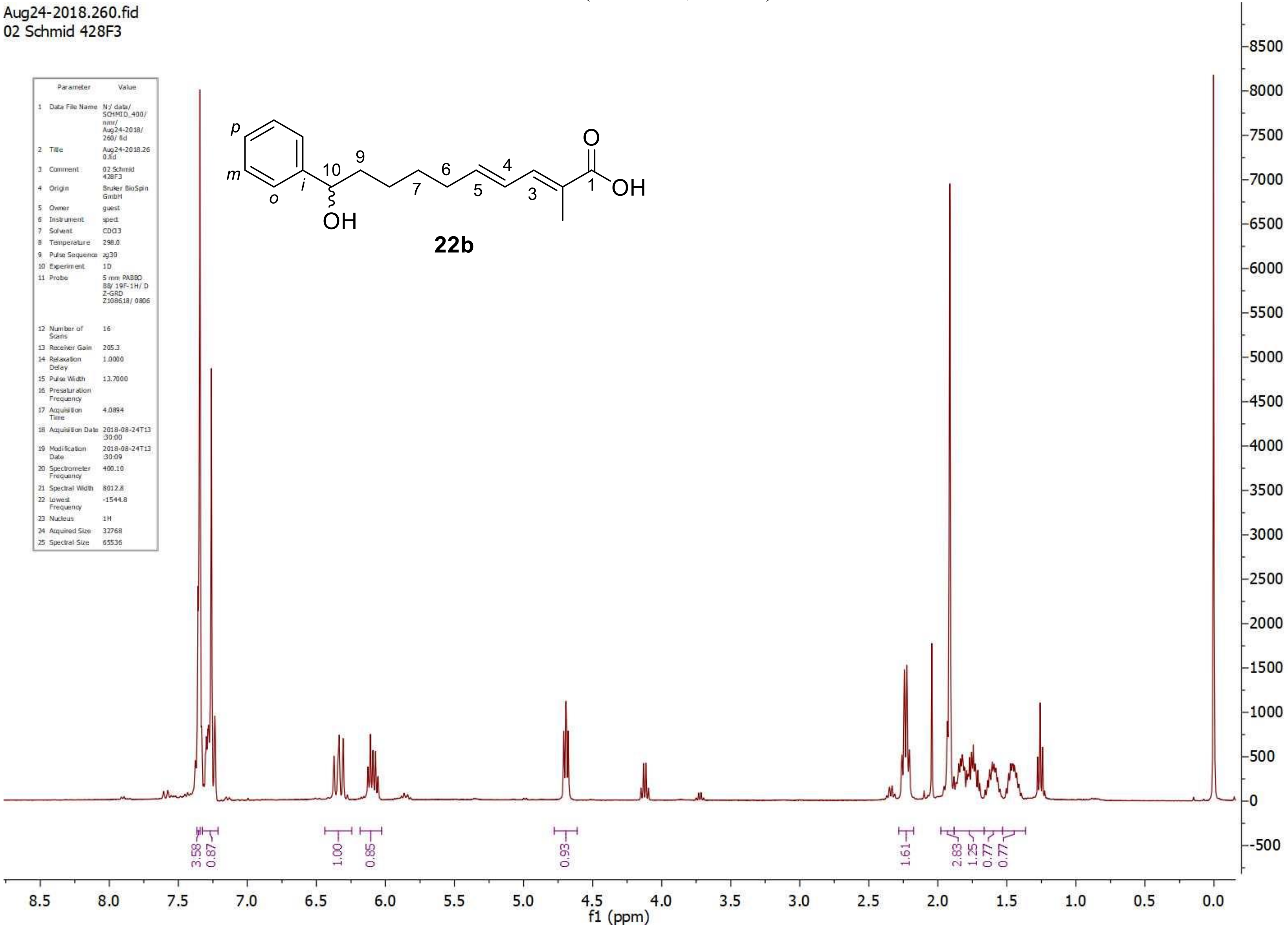

^{13}C NMR (100 MHz, $CDCl_3$) of **22b**

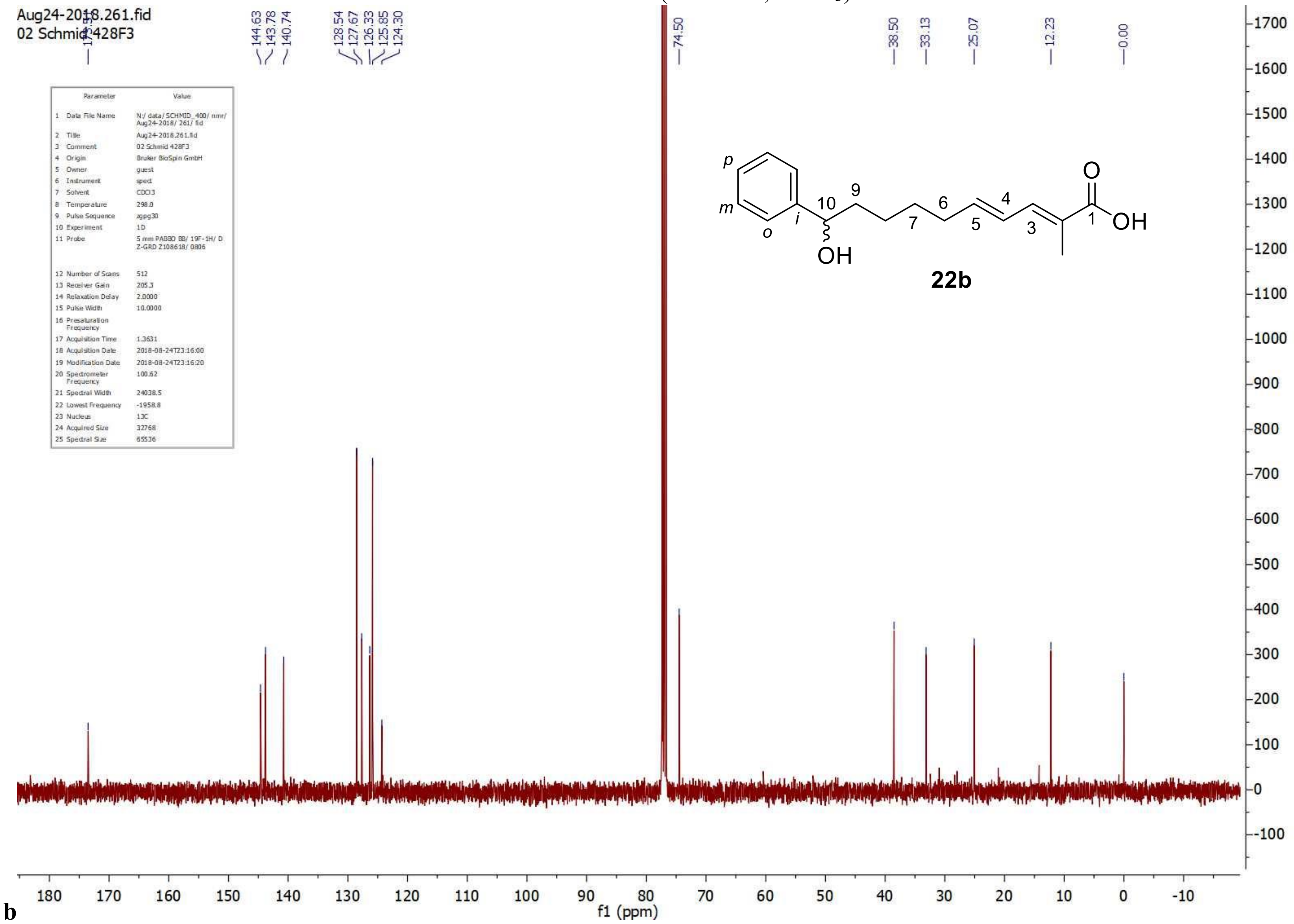

^{1}H NMR (400 MHz, $CDCl_3$) of **20**

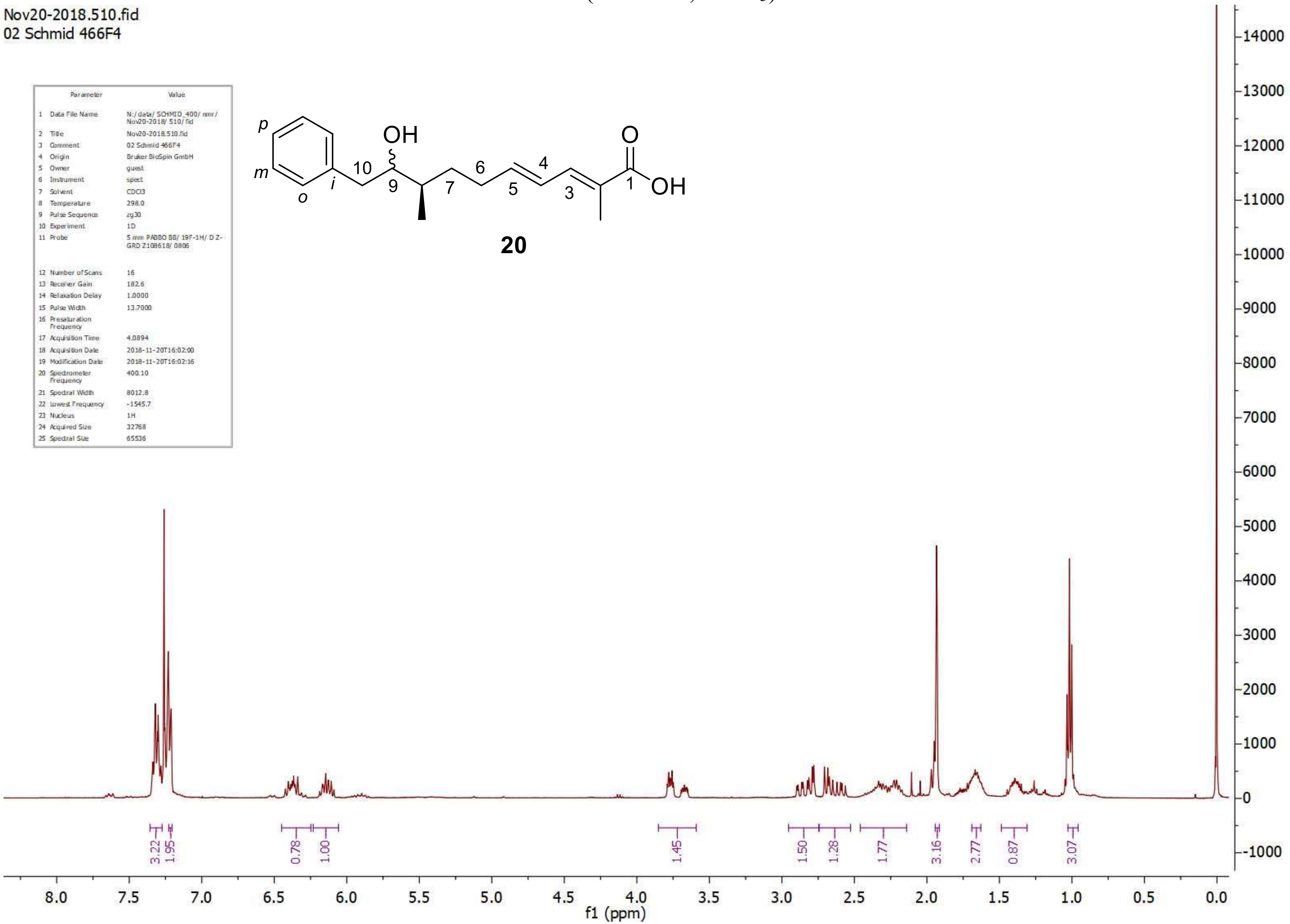

	Parameter	Value
1	Data File Name	N:/ data/ SCHMID_400/ nmr/ Nov20-2018/ 510/ fid
2	Title	Nov20-2018.510.fid
3	Comment	02 Schmid 466F4
4	Origin	Bruker BioSpin GmbH
5	Owner	guest
6	Instrument	spect
7	Solvent	CDCl3
8	Temperature	298.0
9	Pulse Sequence	zg30
10	Experiment	1D
11	Probe	5 mm PABBO BB/ 19F-1H/ D Z-GRD Z108618/ 0806
12	Number of Scans	16
13	Receiver Gain	182.6
14	Relaxation Delay	1.0000
15	Pulse Width	13.7000
16	Presaturation Frequency	
17	Acquisition Time	4.0894
18	Acquisition Date	2018-11-20T16:02:00
19	Modification Date	2018-11-20T16:02:16
20	Spectrometer Frequency	400.10
21	Spectral Width	8012.8
22	lowest Frequency	-1545.7
23	Nucleus	1H
24	Acquired Size	32768
25	Spectral Size	65536

^{13}C NMR (100 MHz, $CDCl_3$) of **20**

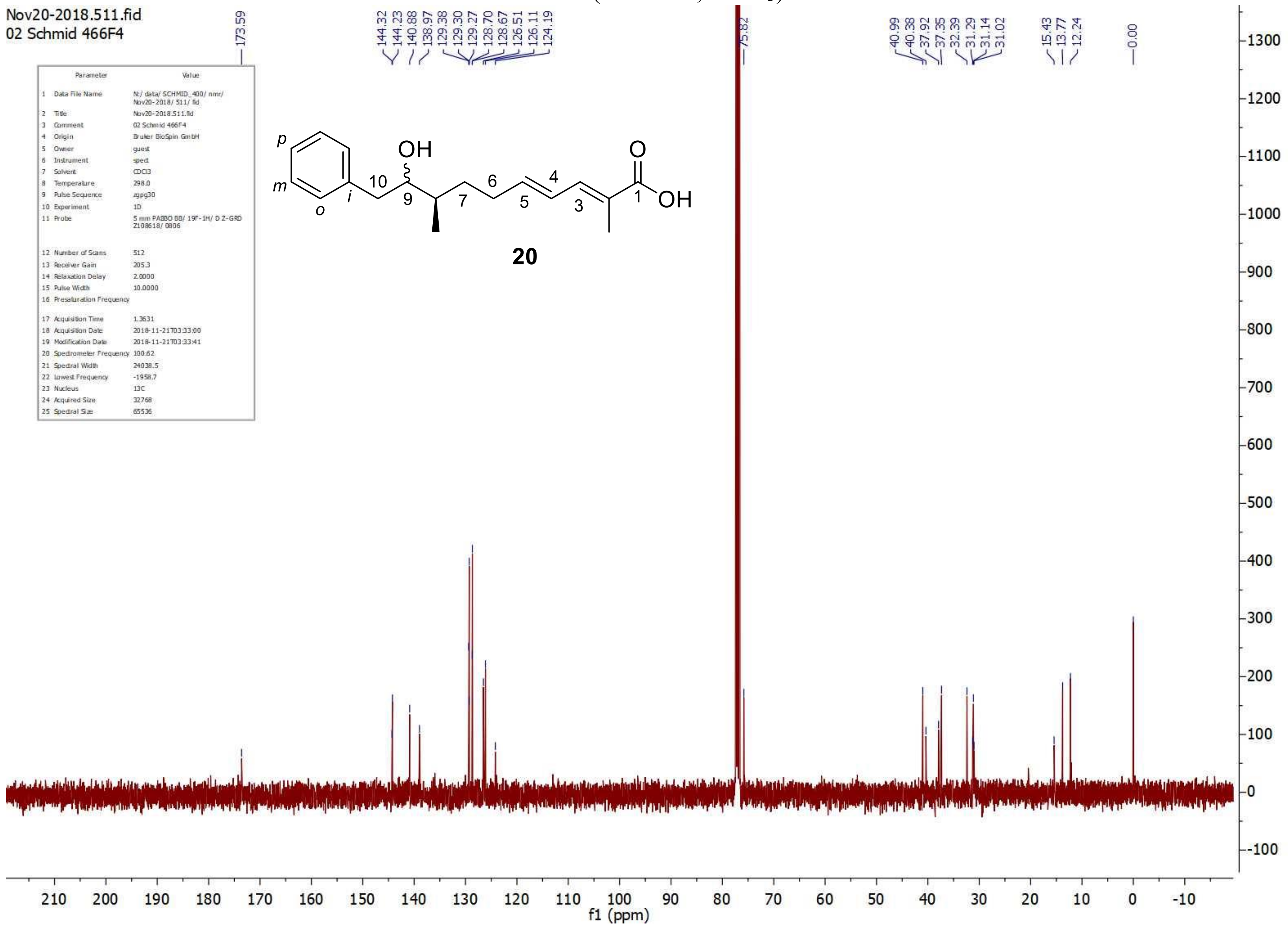

^{1}H NMR (700 MHz, $CDCl_3$) of **S13a**

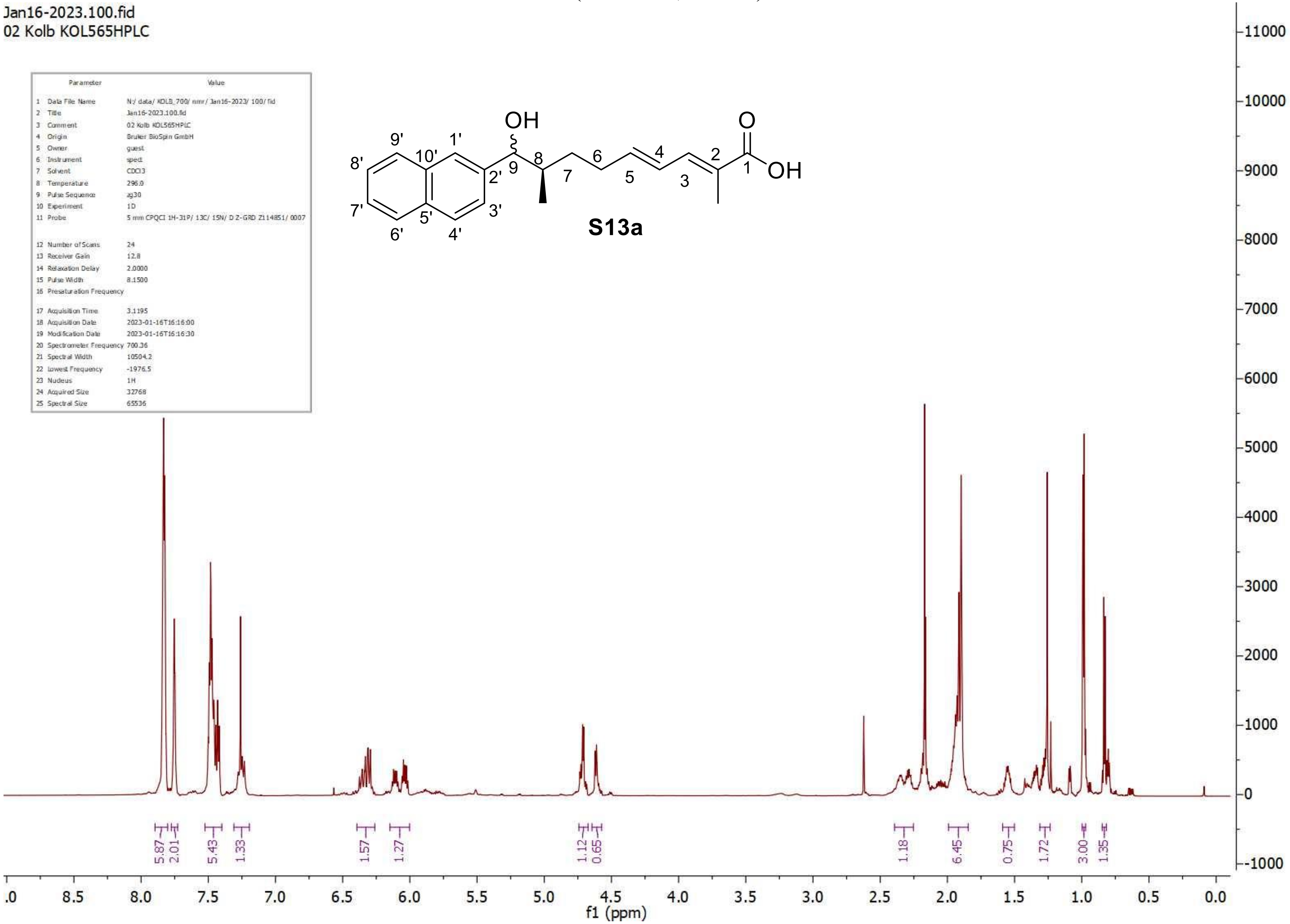

^{13}C NMR (176 MHz, $CDCl_3$) of **S13a**

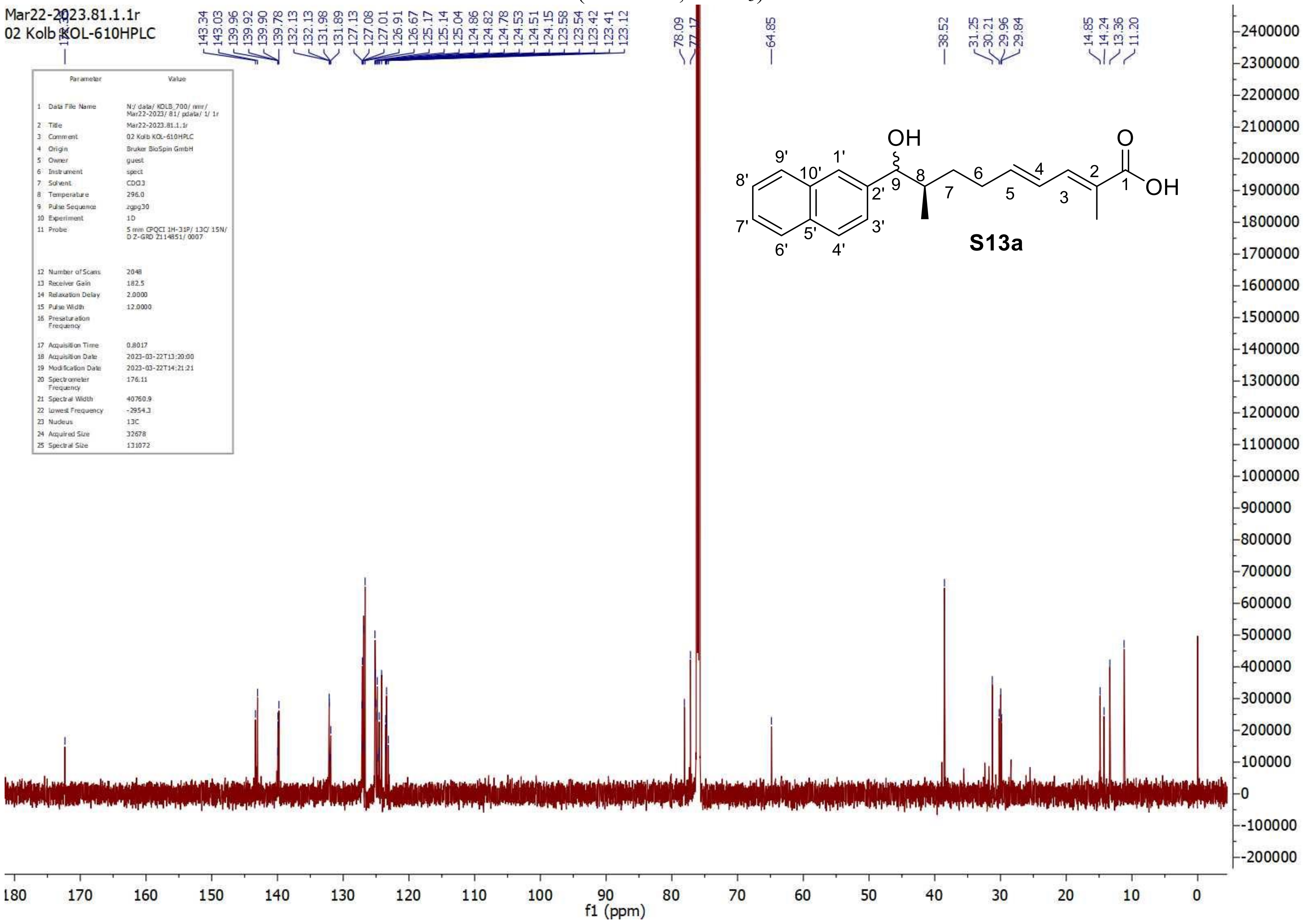

^{1}H NMR (400 MHz, $CDCl_3$) of **S13c**

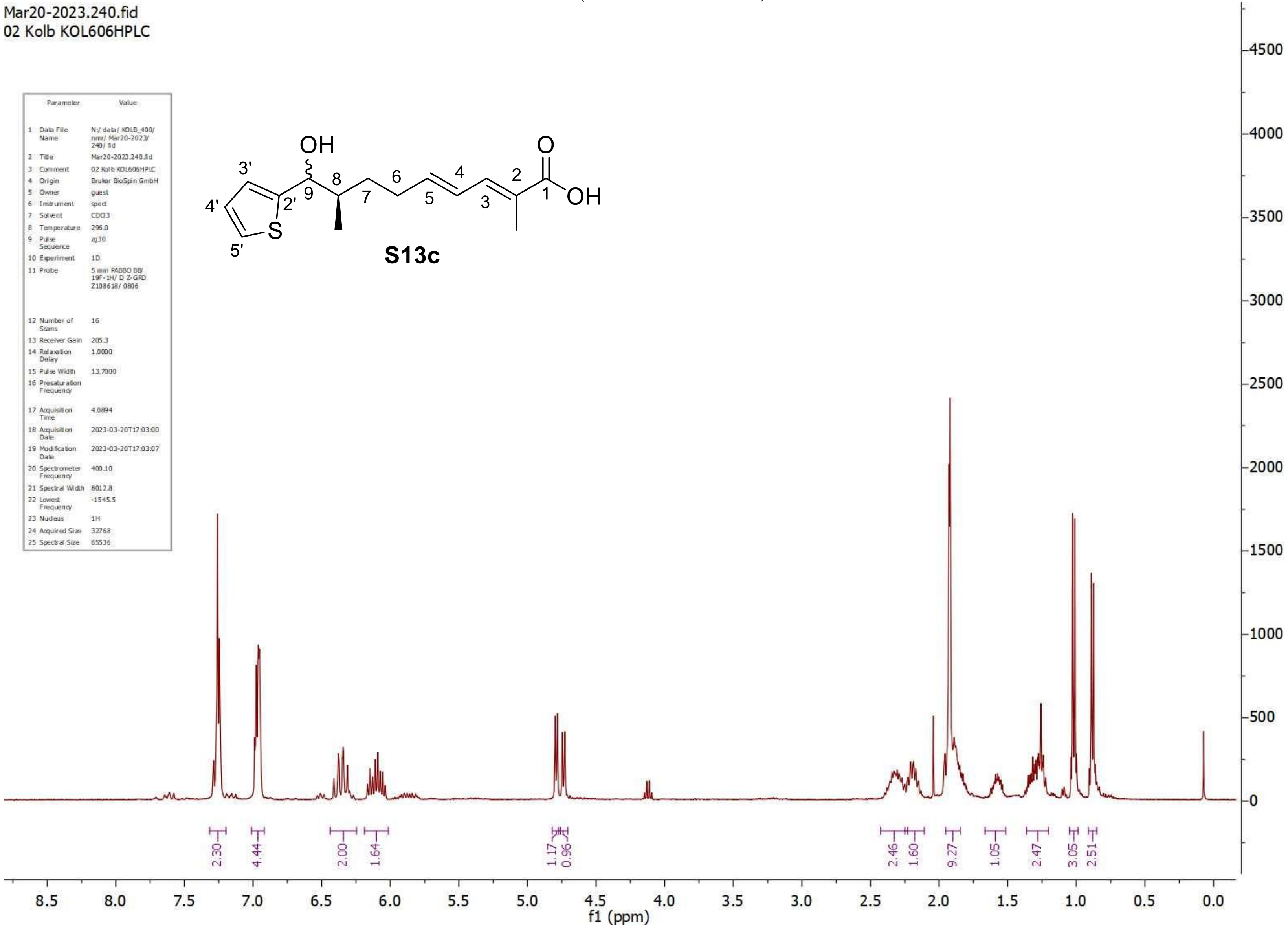

^{13}C NMR (100 MHz, $CDCl_3$) of **S13c**

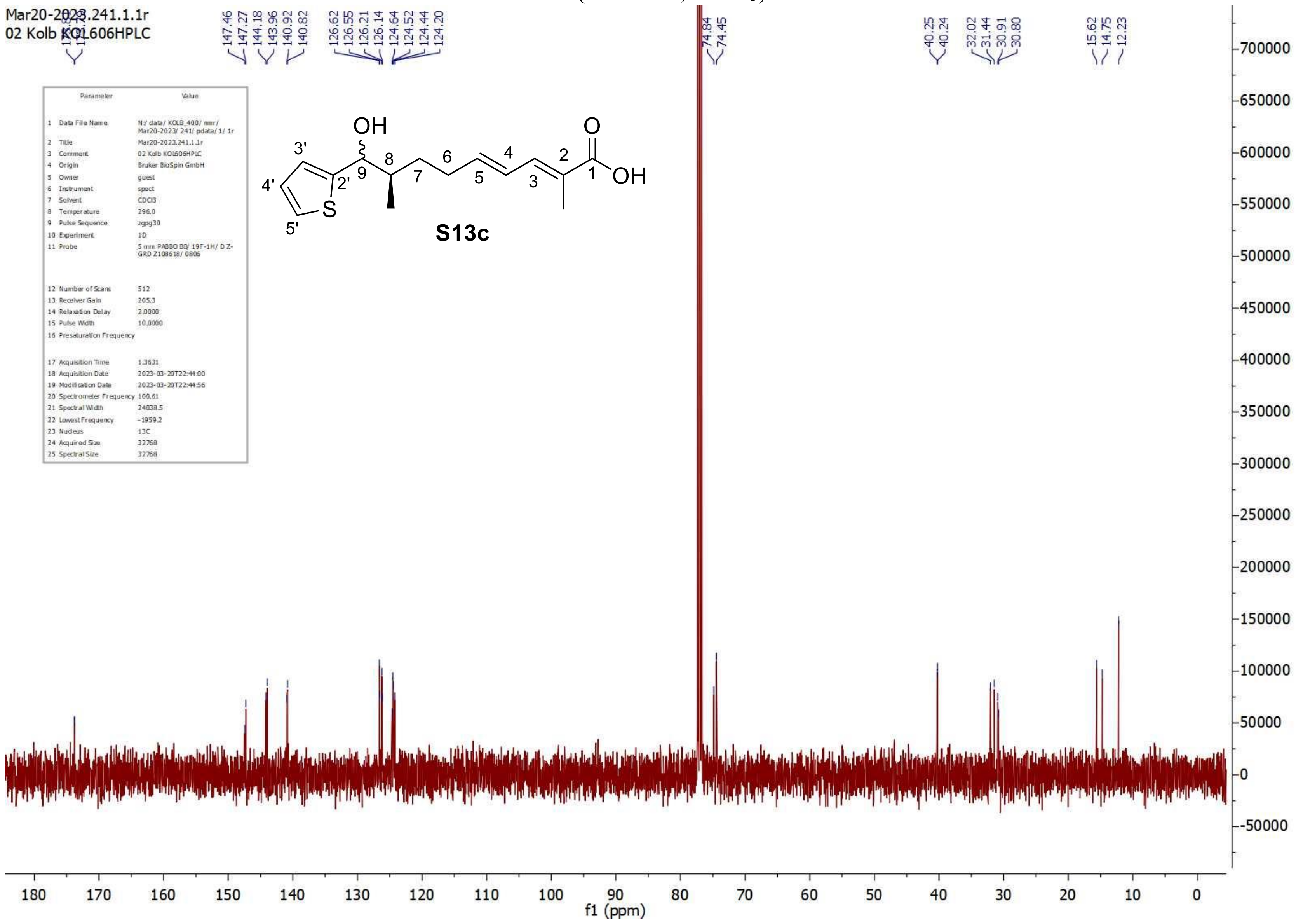

^{1}H NMR (700 MHz, $CDCl_3$) of **S15**

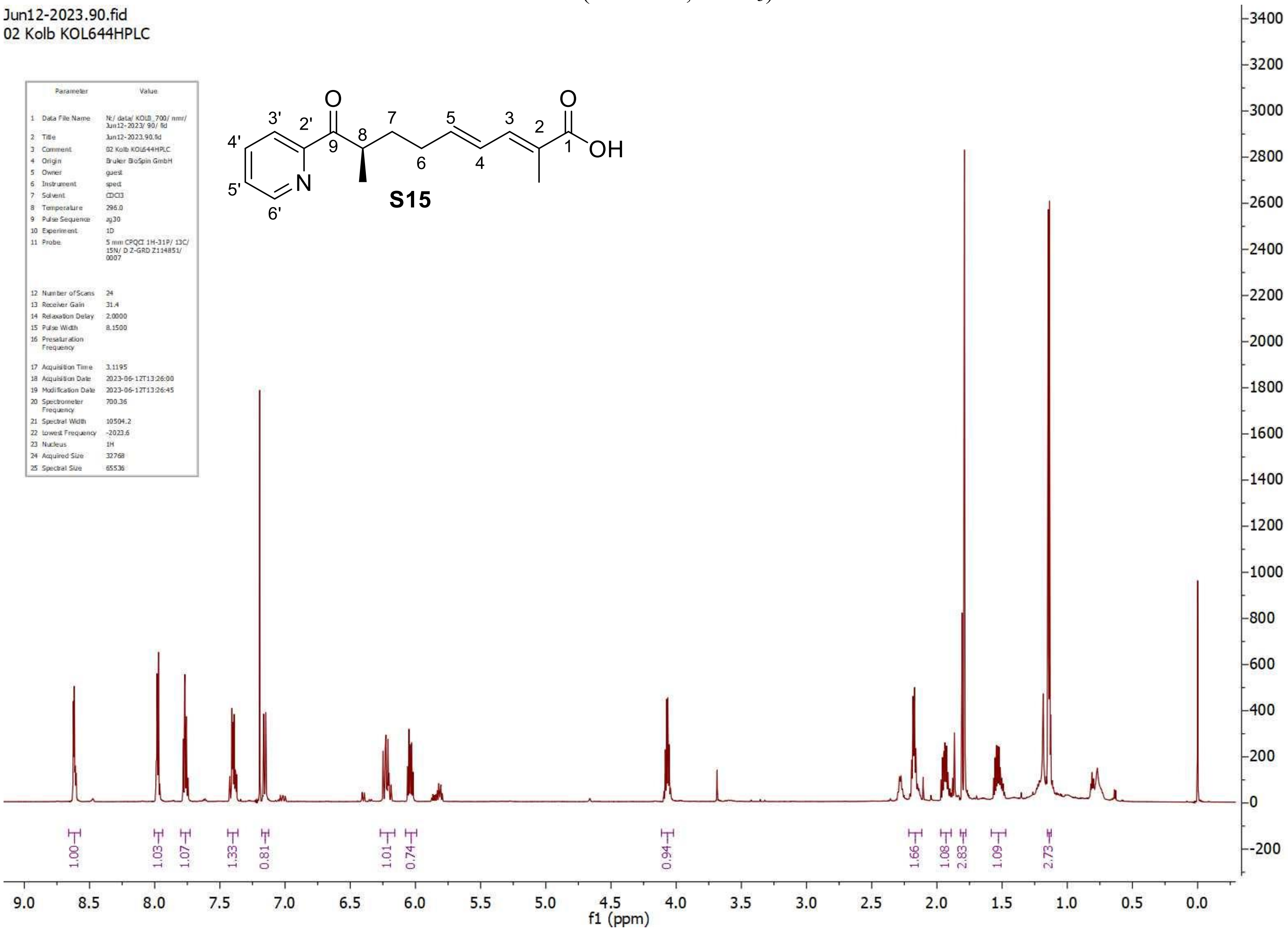

^{13}C NMR (176 MHz, $CDCl_3$) of **S15**

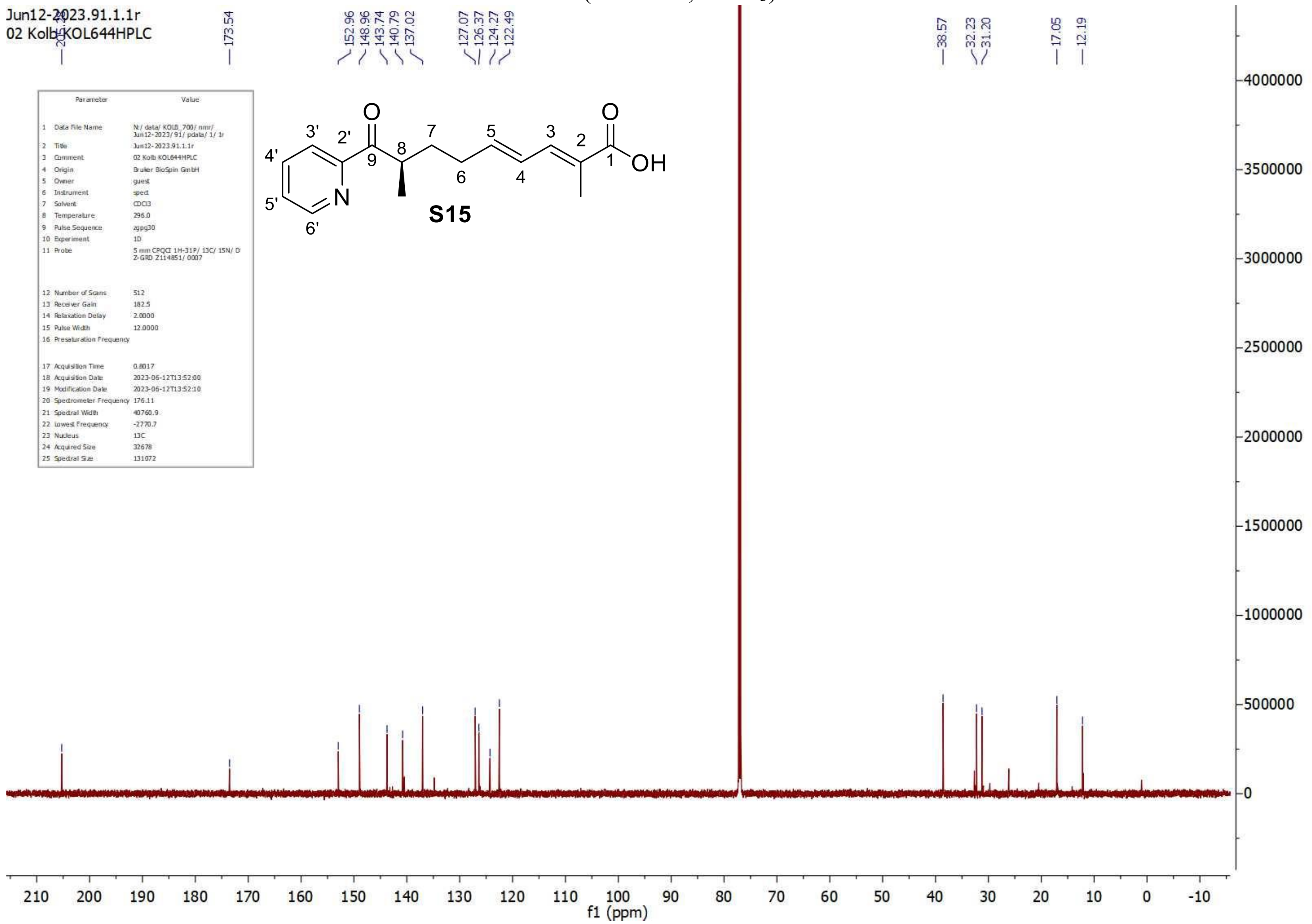

^{1}**H NMR** (400 MHz, $CDCl_3$) of **23a**

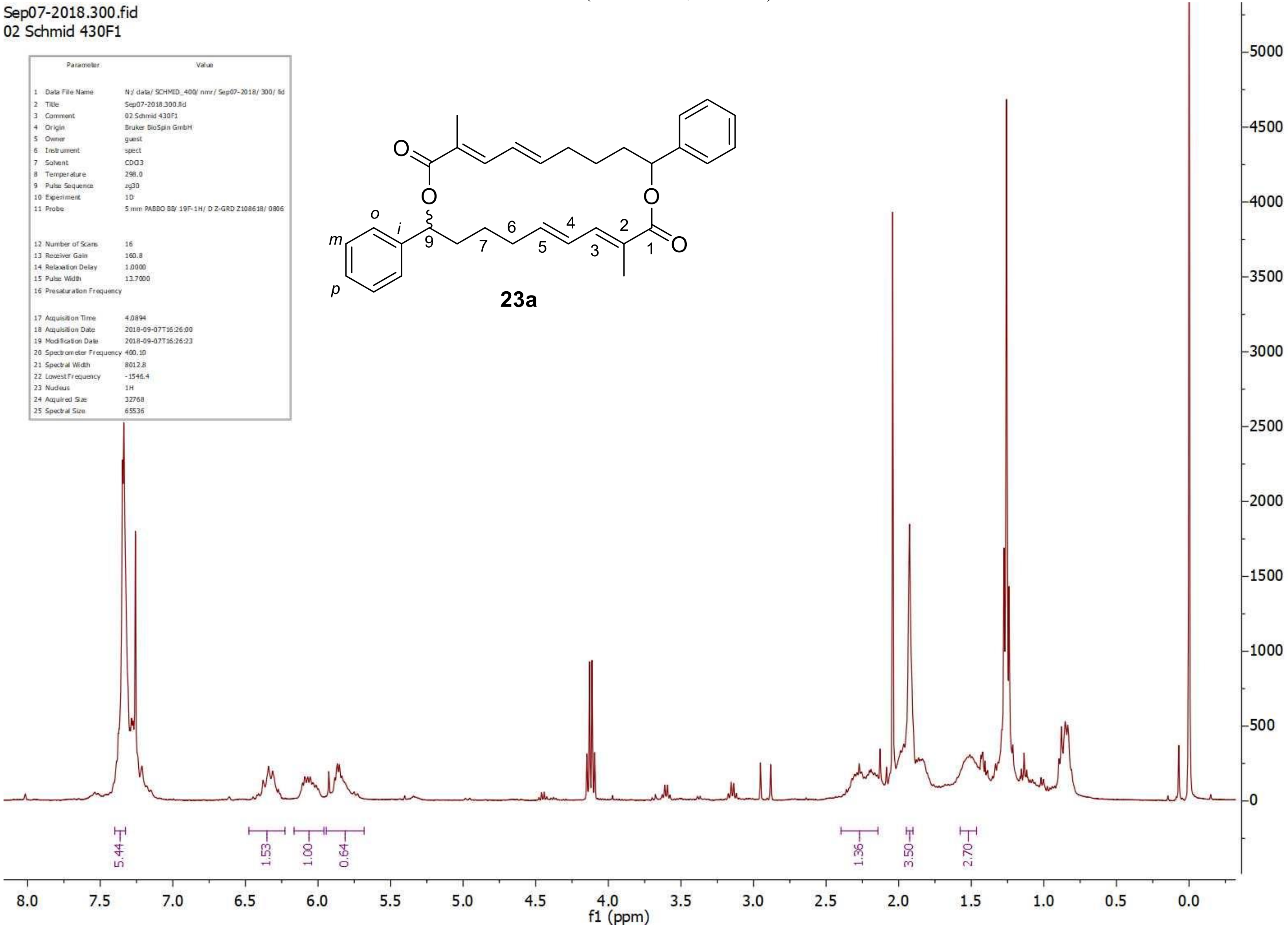

^{13}C NMR (100 MHz, CDCl$_3$) of 23a

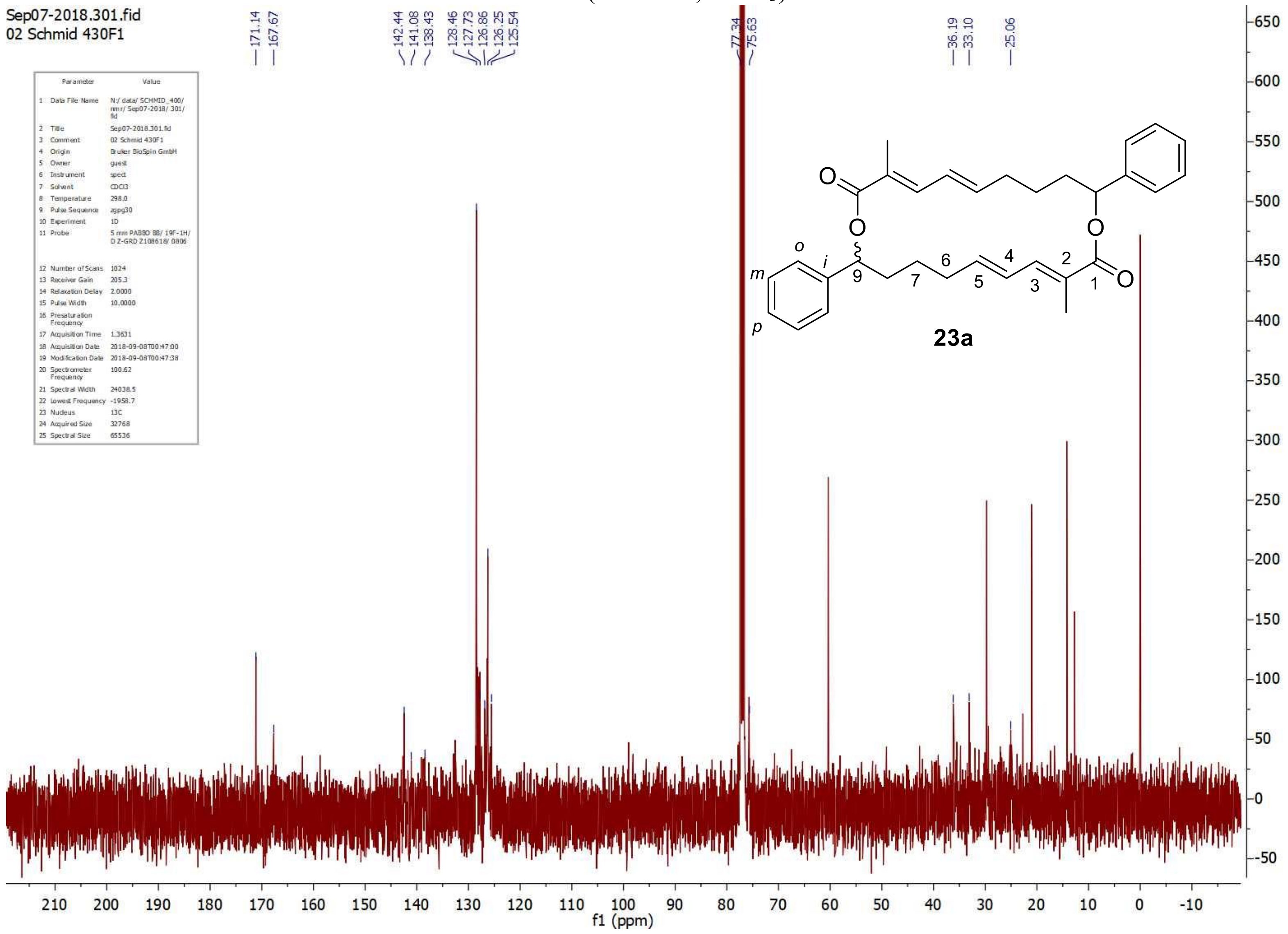

MS (ESI) of **23a**

Massenspektrometrie - Universität Stuttgart

Analysis Info

Analysis Name	Schmid1-FSM367F1_3_01_12773.d	Acquisition Date	3/19/2018 10:19:50 AM
Method	tune-low-low-rate-maerz-neg.m	Operator	BDAL@DE
Sample Name	Schmid1-FSM367F1	Instrument	micrOTOF-Q 228888.00043
Comment			

Acquisition Parameter

Source Type	ESI	Ion Polarity	Negative	Set Nebulizer	0.4 Bar
Focus	Not active	Set Capillary	4500 V	Set Dry Heater	200 °C
Scan Begin	50 m/z	Set End Plate Offset	-500 V	Set Dry Gas	4.0 l/min
Scan End	1000 m/z	Set Collision Cell RF	180.0 Vpp	Set Divert Valve	Waste

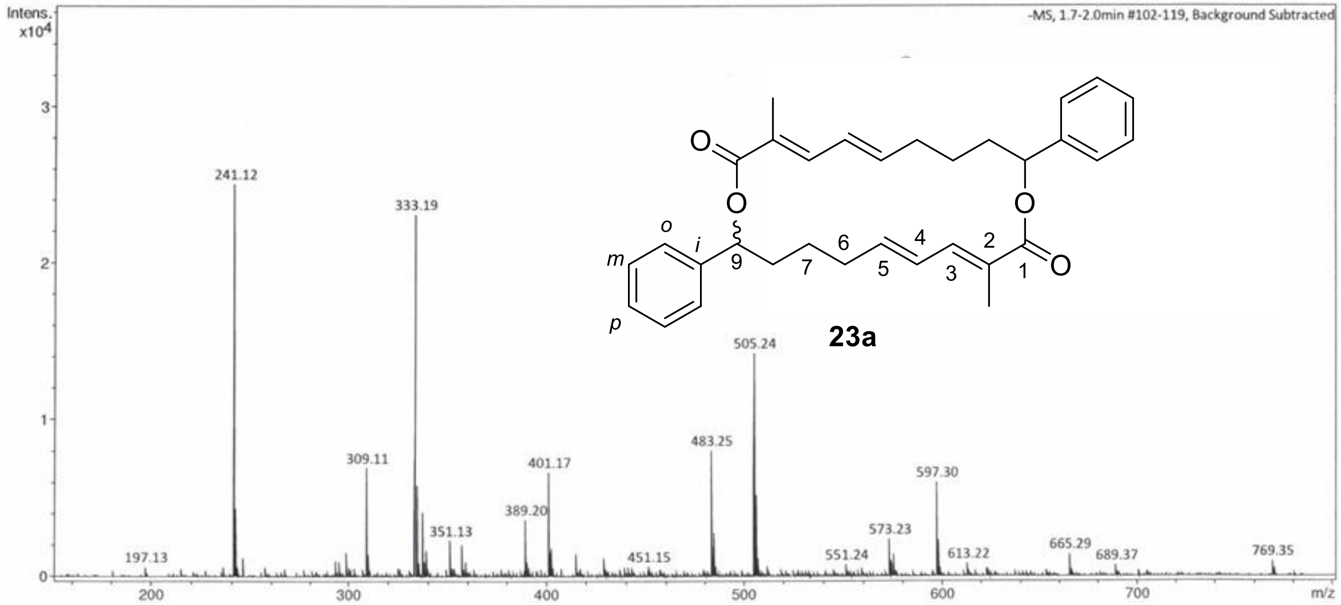

Bruker Compass DataAnalysis 4.2 printed: 3/19/2018 11:55:46 AM Page 1 of 1

HRMS of **23a**

Massenspektrometrie - Universität Stuttgart

Analysis Info

Analysis Name	Schmid1-FSM367F1_3_01_12773.d	Acquisition Date	3/19/2018 10:19:50 AM
Method	tune-low-low-rate-maerz-neg.m	Operator	BDAL@DE
Sample Name	Schmid1-FSM367F1	Instrument	micrOTOF-Q 228888.00043
Comment			

Acquisition Parameter

Source Type	ESI	Ion Polarity	Negative	Set Nebulizer	0.4 Bar		
Focus	Not active	Set Capillary	4500 V	Set Dry Heater	200 °C		
Scan Begin	50 m/z	Set End Plate Offset	-500 V	Set Dry Gas	4.0 l/min		
Scan End	1000 m/z	Set Collision Cell RF	180.0 Vpp	Set Divert Valve	Waste		

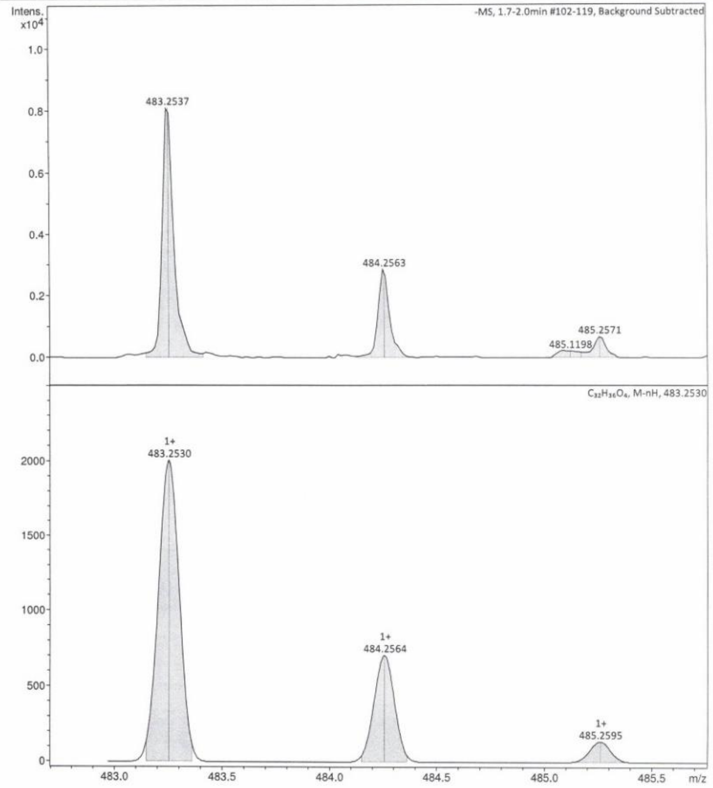

Bruker Compass DataAnalysis 4.2 printed: 3/19/2018 11:58:50 AM Page 1 of 1

¹H NMR (500 MHz, CDCl$_3$) of **23b**

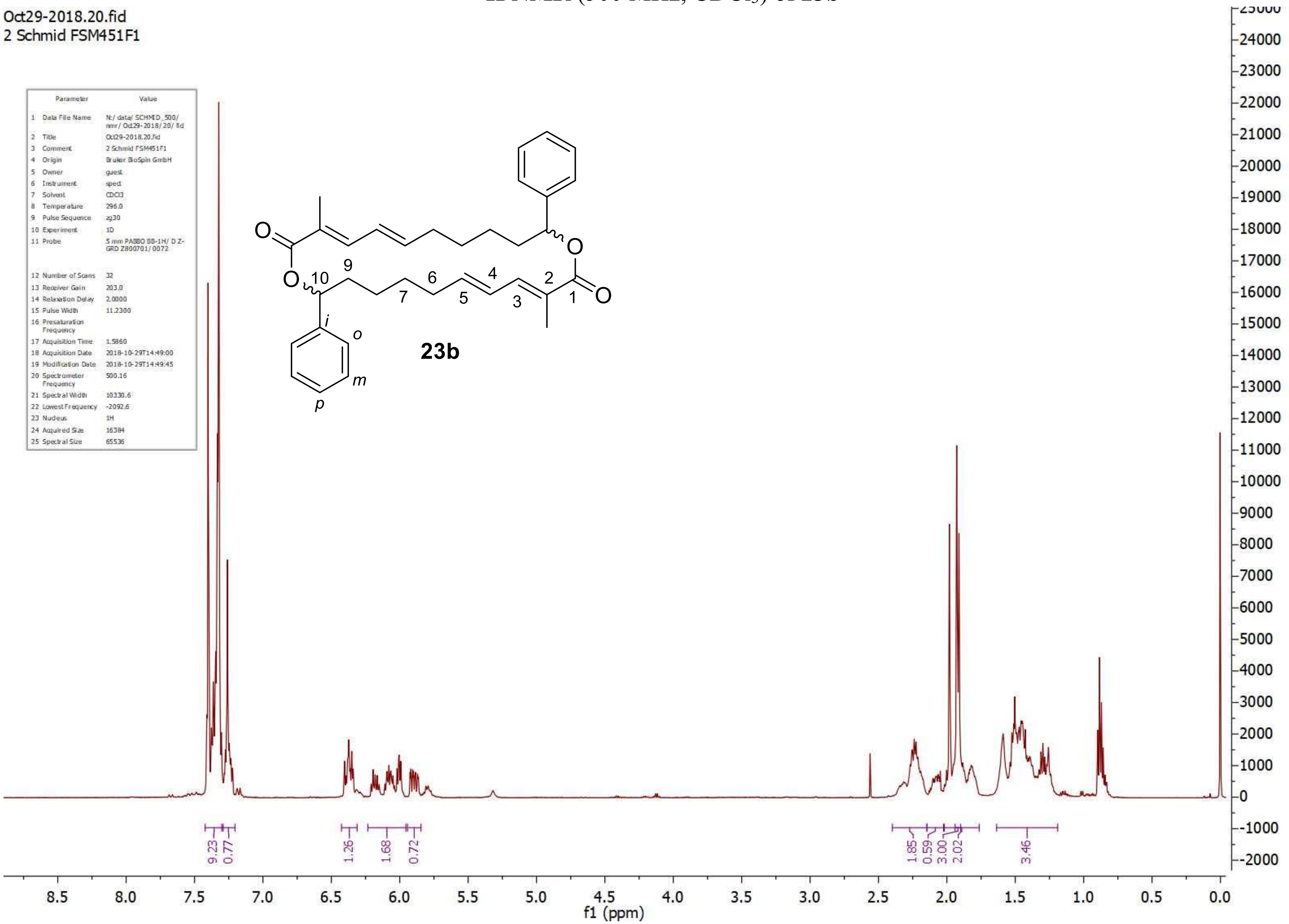

^{13}C NMR (125 MHz, $CDCl_3$) of **23b**

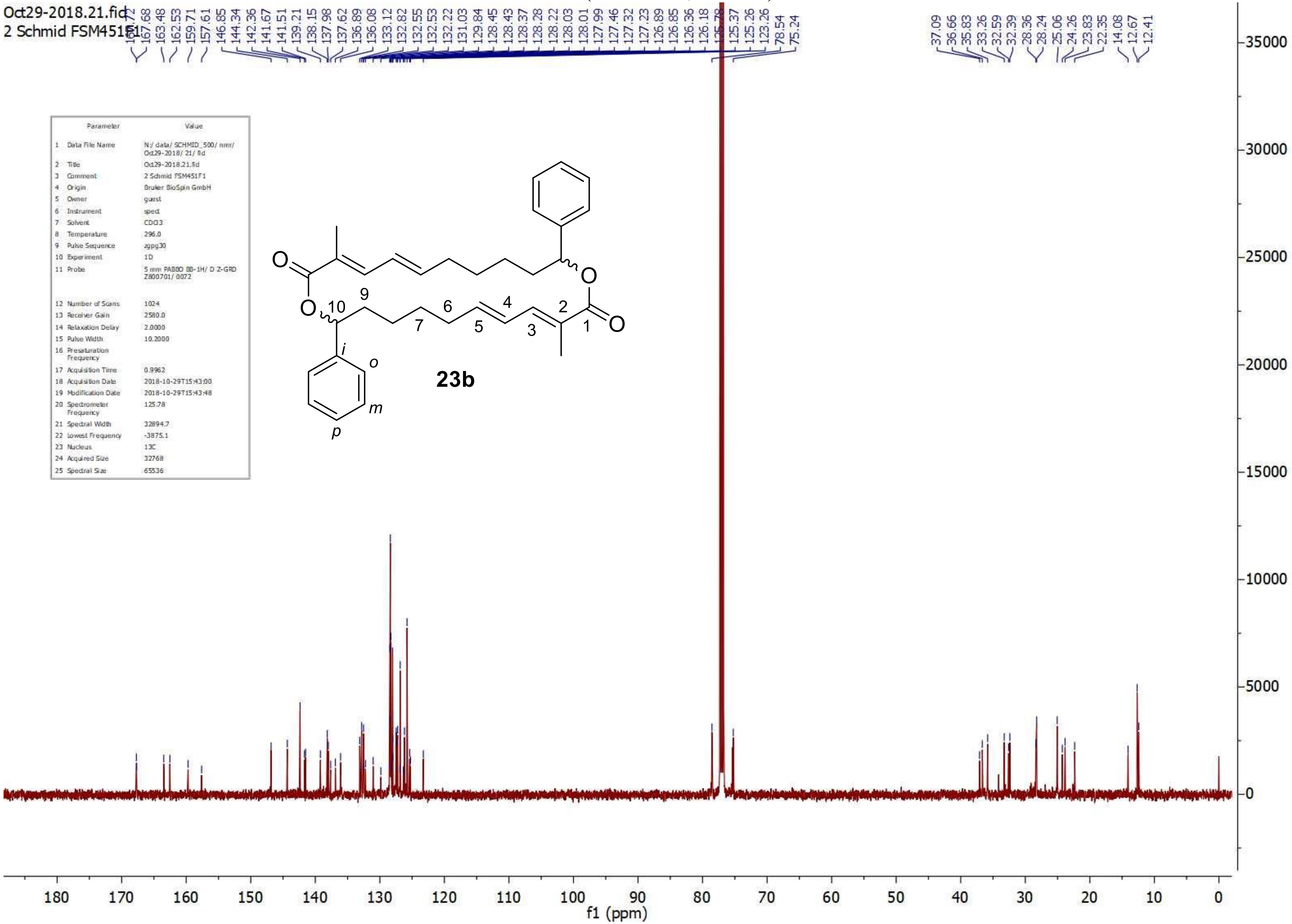

MS (ESI) of **23b**

Schmid

Massenspektrometrie - Universität Stuttgart

Analysis Info

Analysis Name	Schmid-FSM448F2_1_01_17166.d	Acquisition Date	12/6/2018 11:01:36 AM
Method	tune-low-pos-fast-rate-juli-18.m	Operator	BDAL@DE
Sample Name	Schmid-FSM448F2	Instrument	micrOTOF-Q 228888.00043
Comment			

Acquisition Parameter

Source Type	ESI	Ion Polarity	Positive	Set Nebulizer	1.0 Bar
Focus	Not active	Set Capillary	4500 V	Set Dry Heater	210 °C
Scan Begin	50 m/z	Set End Plate Offset	-500 V	Set Dry Gas	8.0 l/min
Scan End	1000 m/z	Set Collision Cell RF	180.0 Vpp	Set Divert Valve	Waste

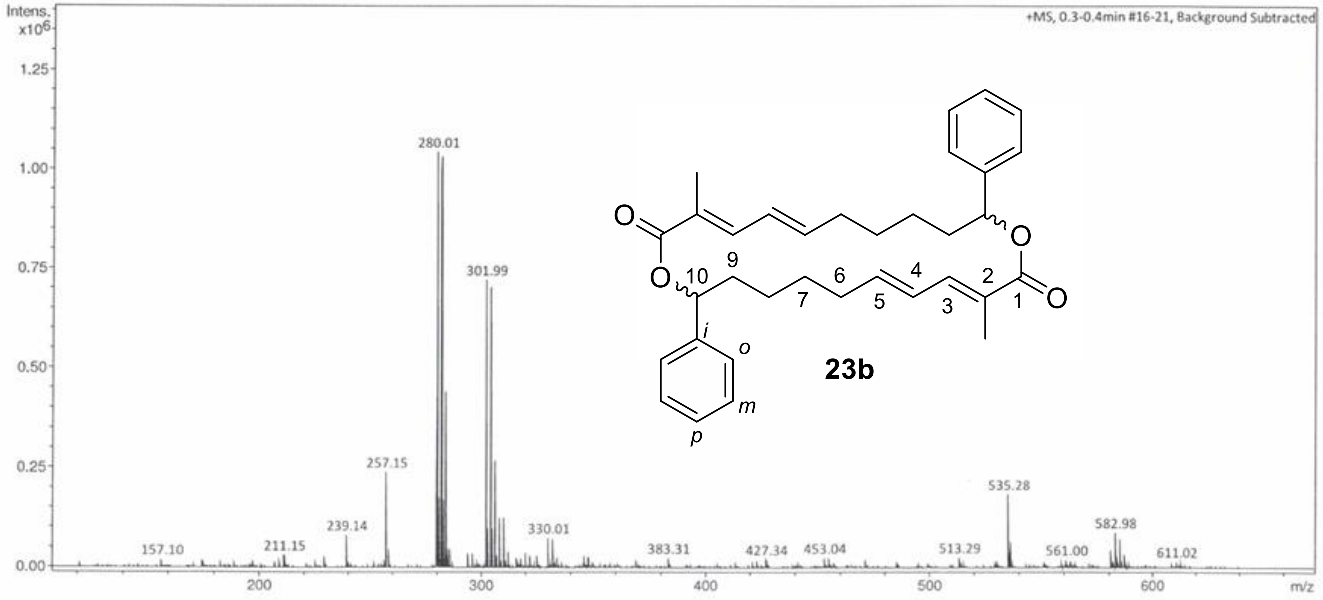

Bruker Compass DataAnalysis 4.2 printed: 12/6/2018 11:05:03 AM Page 1 of 1

HRMS of **23b**

Massenspektrometrie - Universität Stuttgart

Analysis Info

Analysis Name	Schmid-FSM448F2_1_01_17166.d	Acquisition Date	12/6/2018 11:01:36 AM
Method	tune-low-pos-fast-rate-juli-18.m	Operator	BDAL@DE
Sample Name	Schmid-FSM448F2	Instrument	micrOTOF-Q 228888.00043
Comment			

Acquisition Parameter

Source Type	ESI	Ion Polarity	Positive	Set Nebulizer	1.0 Bar
Focus	Not active	Set Capillary	4500 V	Set Dry Heater	210 °C
Scan Begin	50 m/z	Set End Plate Offset	-500 V	Set Dry Gas	8.0 l/min
Scan End	1000 m/z	Set Collision Cell RF	180.0 Vpp	Set Divert Valve	Waste

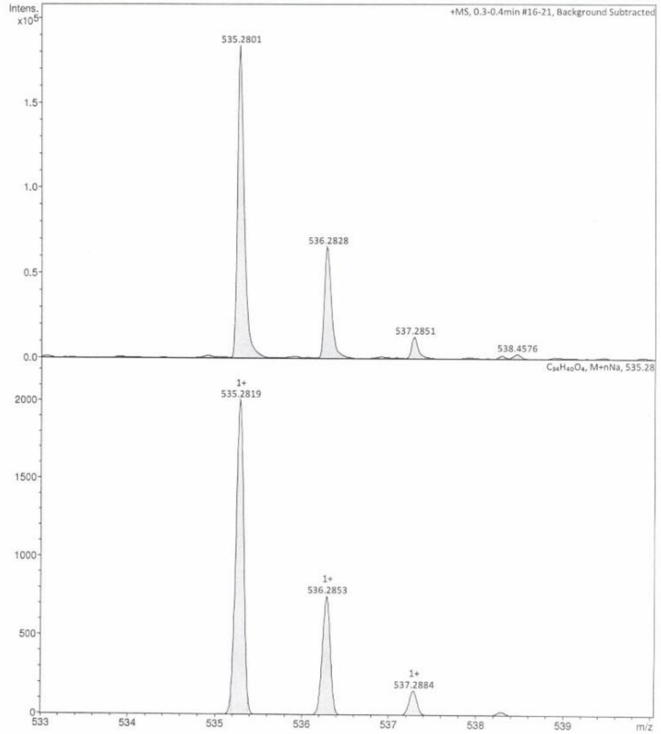

Bruker Compass DataAnalysis 4.2 printed: 12/6/2018 11:05:53 AM Page 1 of 1

^{1}H NMR (500 MHz, CDCl$_3$) of **21**

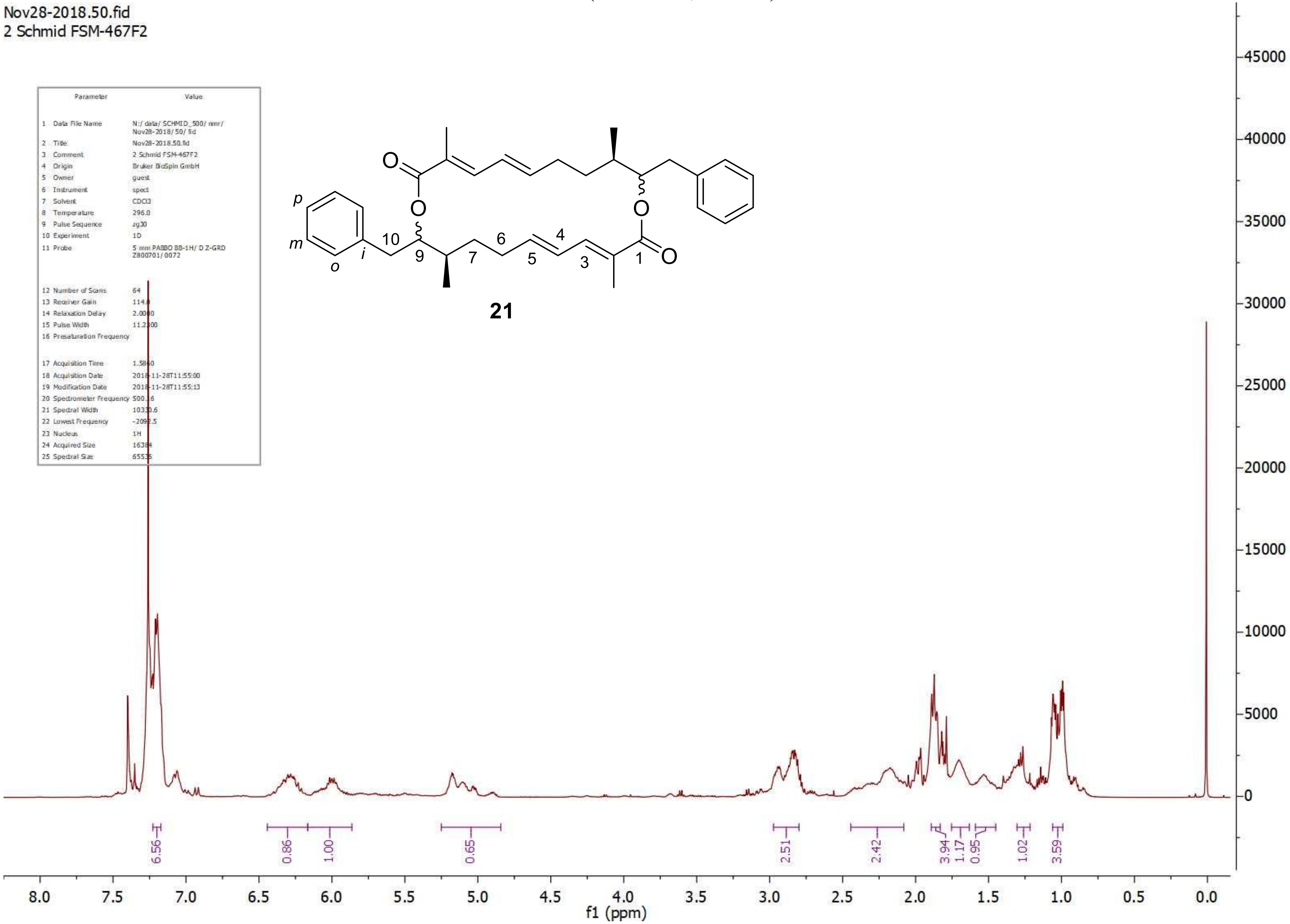

^{13}C NMR (125 MHz, $CDCl_3$) of **21**

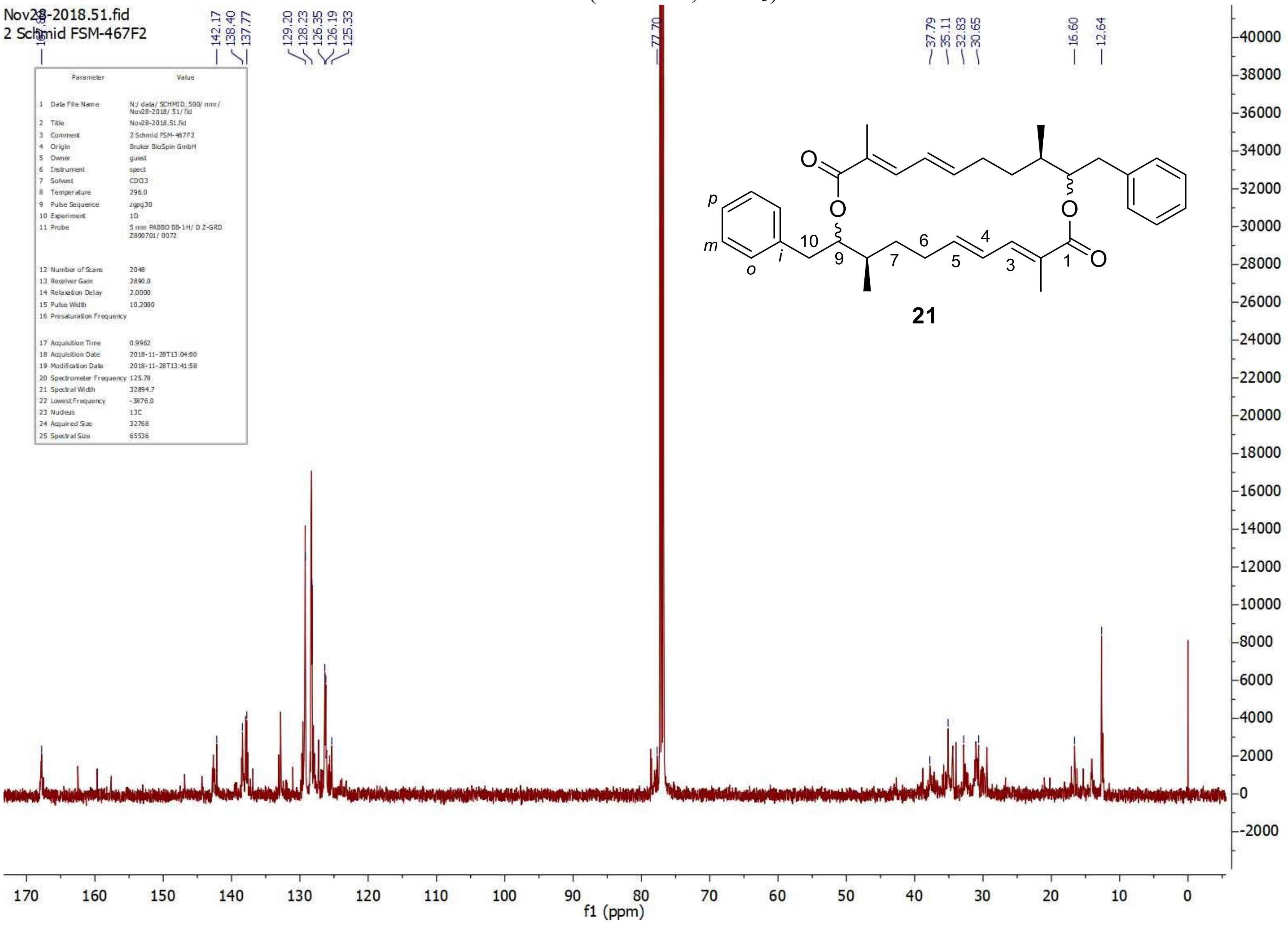

MS (ESI) of **21**

Massenspektrometrie - Universität Stuttgart

Analysis Info

Analysis Name	Schmid-FSM467F2_3_01_17076.d	Acquisition Date	11/27/2018 1:41:02 PM
Method	tune-wide-pos-low-rate-juli.m	Operator	BDAL@DE
Sample Name	Schmid-FSM467F2	Instrument	micrOTOF-Q 228888.00043
Comment			

Acquisition Parameter

Source Type	ESI	Ion Polarity	Positive		Set Nebulizer	0.4 Bar
Focus	Not active	Set Capillary	4500 V		Set Dry Heater	200 °C
Scan Begin	250 m/z	Set End Plate Offset	-500 V		Set Dry Gas	4.0 l/min
Scan End	3000 m/z	Set Collision Cell RF	500.0 Vpp		Set Divert Valve	Waste

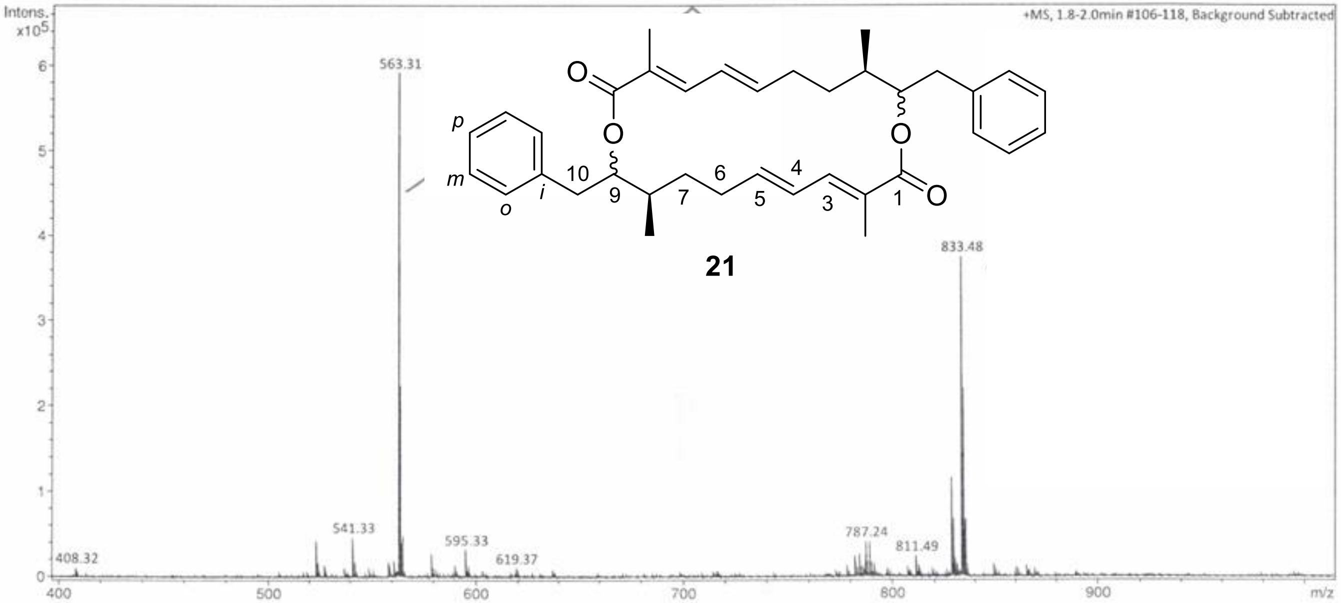

Bruker Compass DataAnalysis 4.2 printed: 11/27/2018 1:57:19 PM Page 1 of 1

HRMS of **21**

Massenspektrometrie - Universität Stuttgart

Analysis Info

Analysis Name	Schmid-FSM467F2_3_01_17076.d	Acquisition Date	11/27/2018 1:41:02 PM
Method	tune-wide-pos-low-rate-juli.m	Operator	BDAL@DE
Sample Name	Schmid-FSM467F2	Instrument	micrOTOF-Q 228888.00043
Comment			

Acquisition Parameter

Source Type	ESI	Ion Polarity	Positive	Set Nebulizer	0.4 Bar
Focus	Not active	Set Capillary	4500 V	Set Dry Heater	200 °C
Scan Begin	250 m/z	Set End Plate Offset	-500 V	Set Dry Gas	4.0 l/min
Scan End	3000 m/z	Set Collision Cell RF	500.0 Vpp	Set Divert Valve	Waste

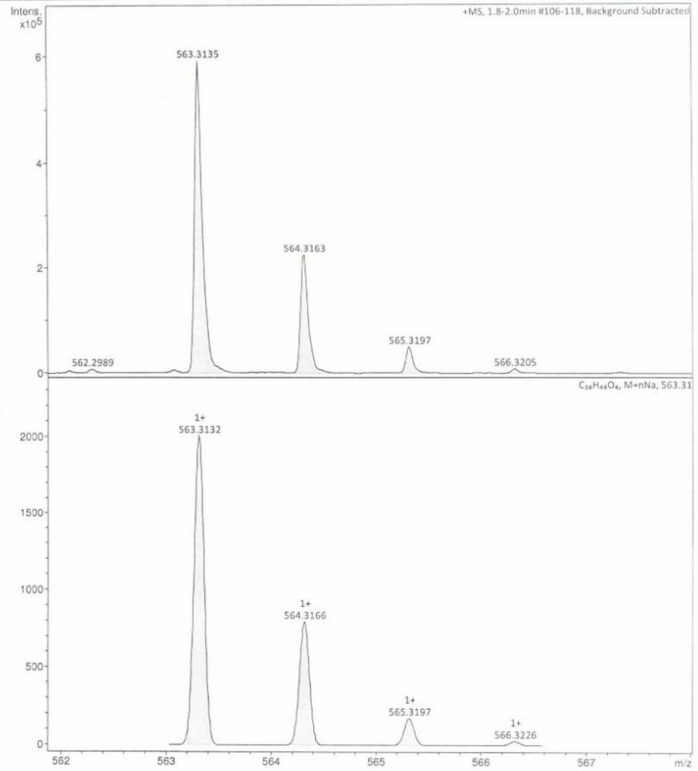

Bruker Compass DataAnalysis 4.2 printed: 11/27/2018 1:58:32 PM Page 1 of 1

^{1}H NMR (500 MHz, $CDCl_3$) of Ts-**S5**

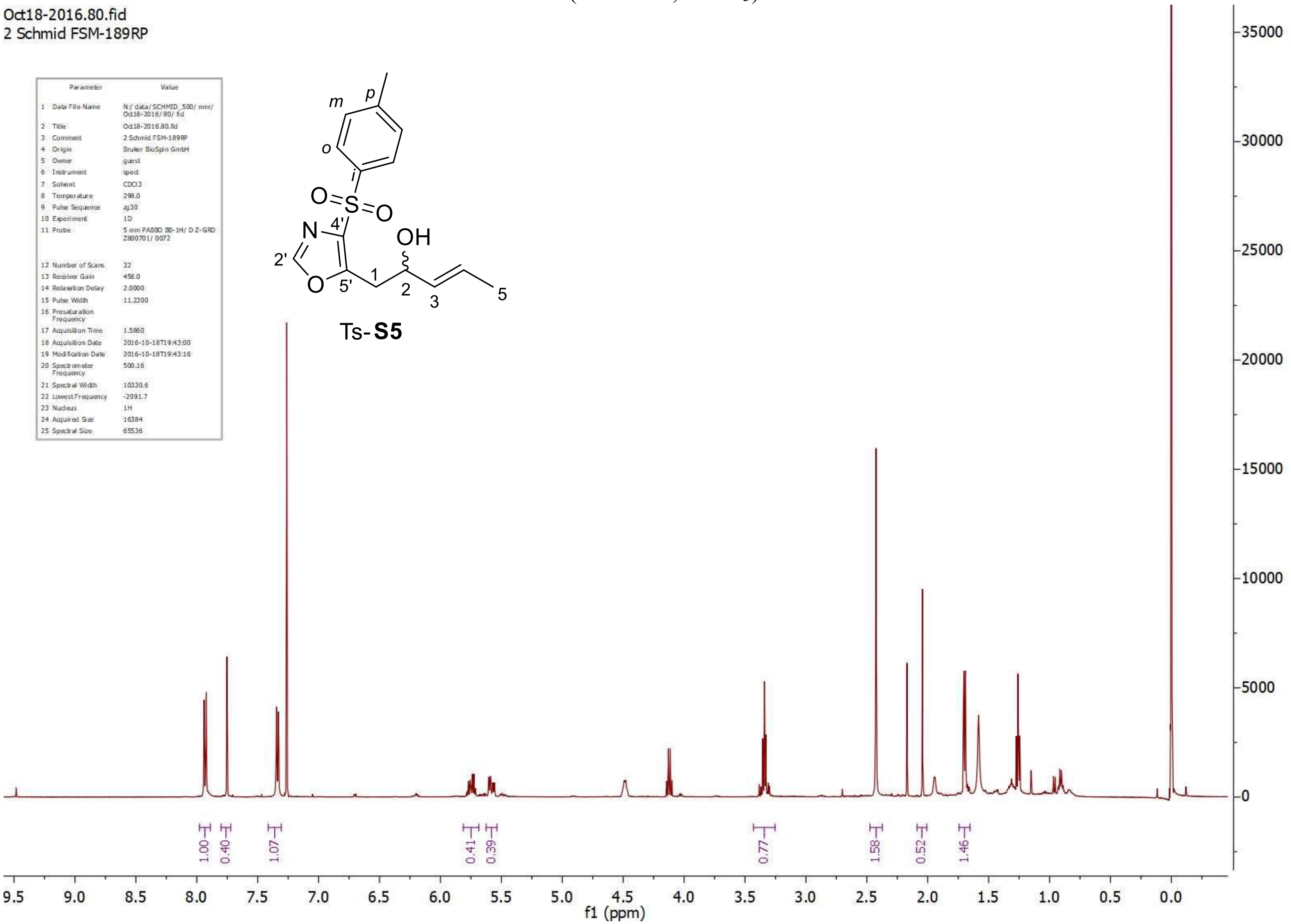

^{13}**C NMR** (125 MHz, $CDCl_3$) of Ts-**S5**

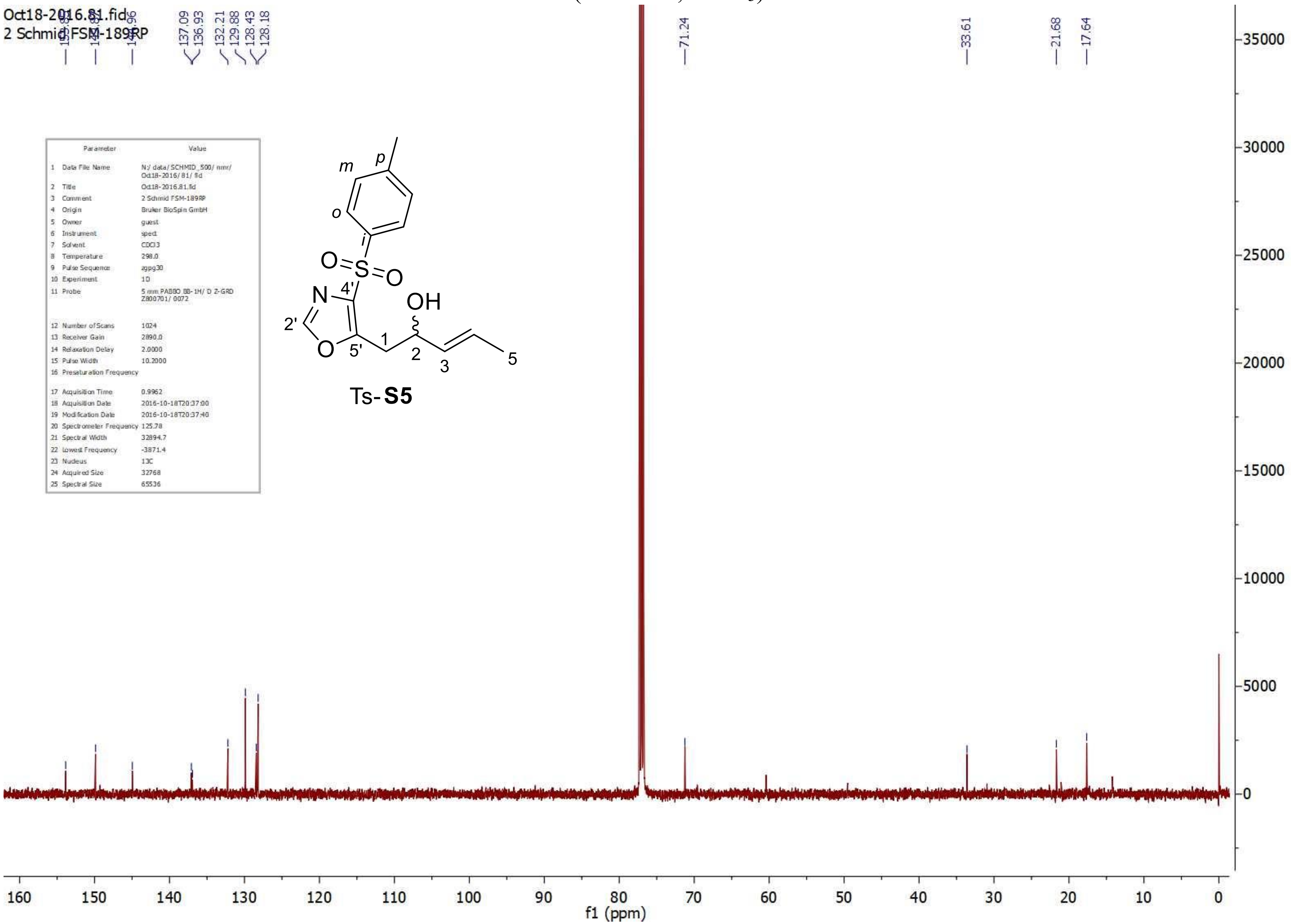

^{1}H NMR (400 MHz, $CDCl_3$) of **S5**

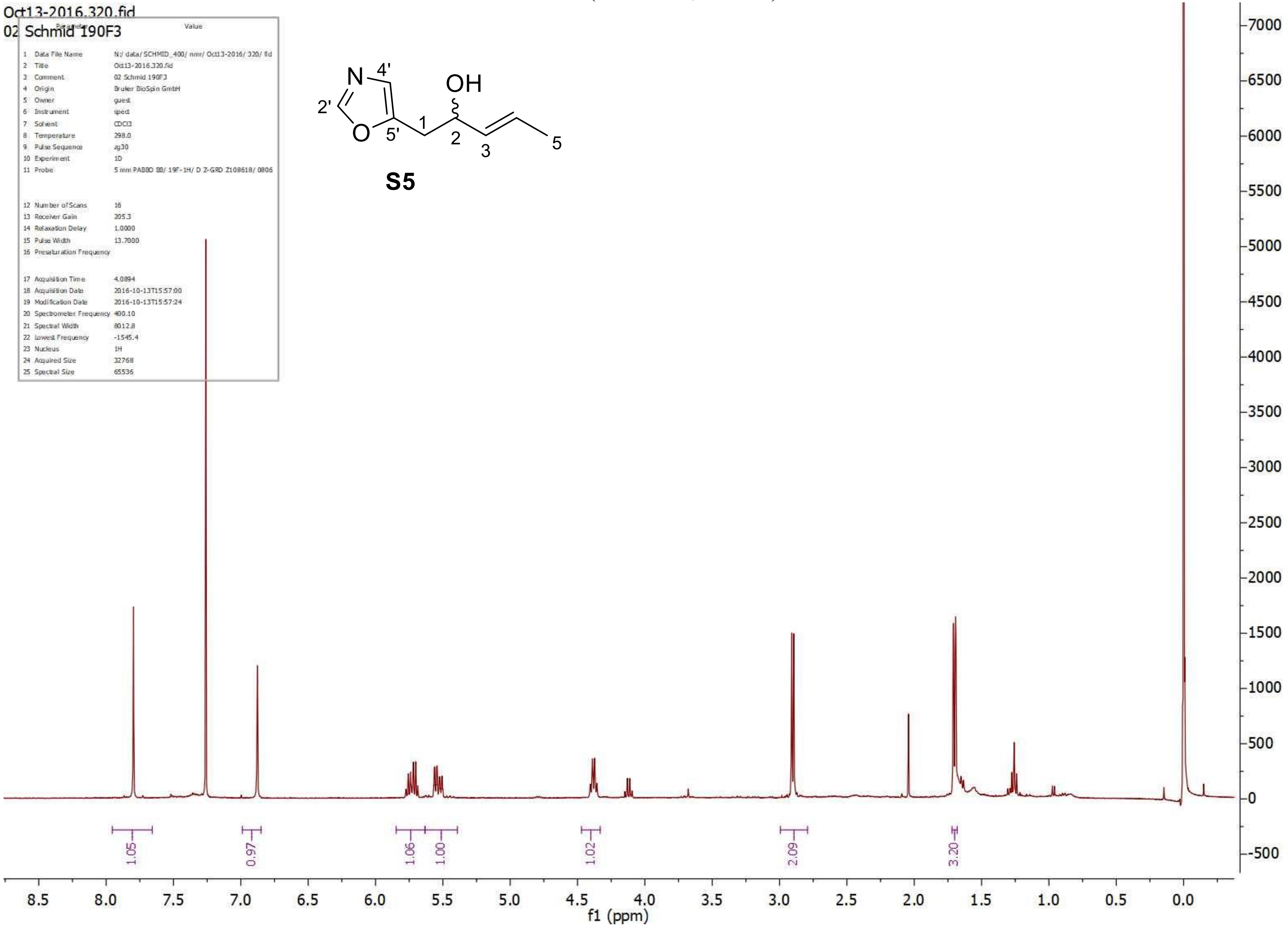

^{13}**C NMR** (100 MHz, $CDCl_3$) of **S5**

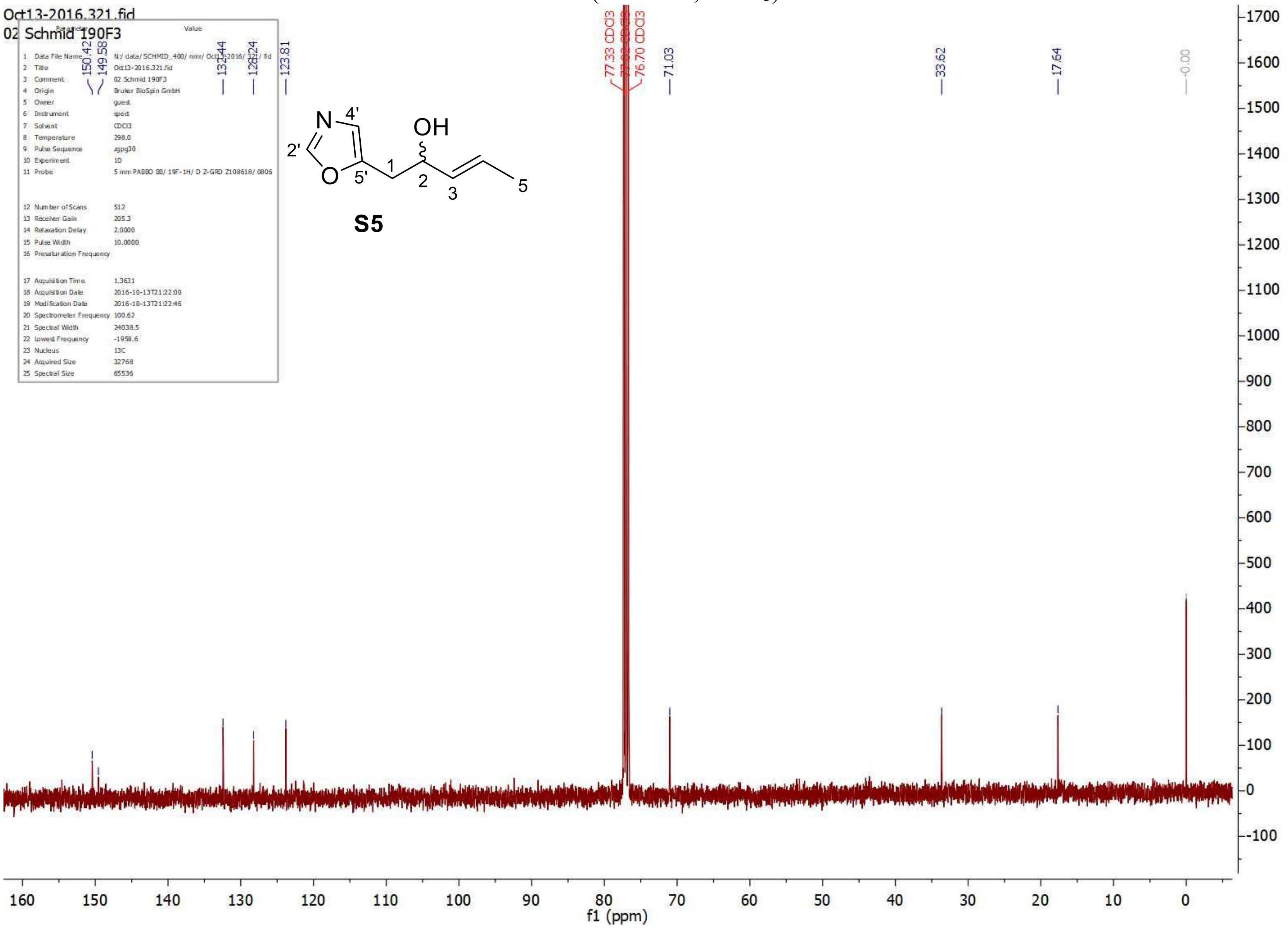

^{1}**H NMR** (500 MHz, $CDCl_3$) of **Ts-S6**

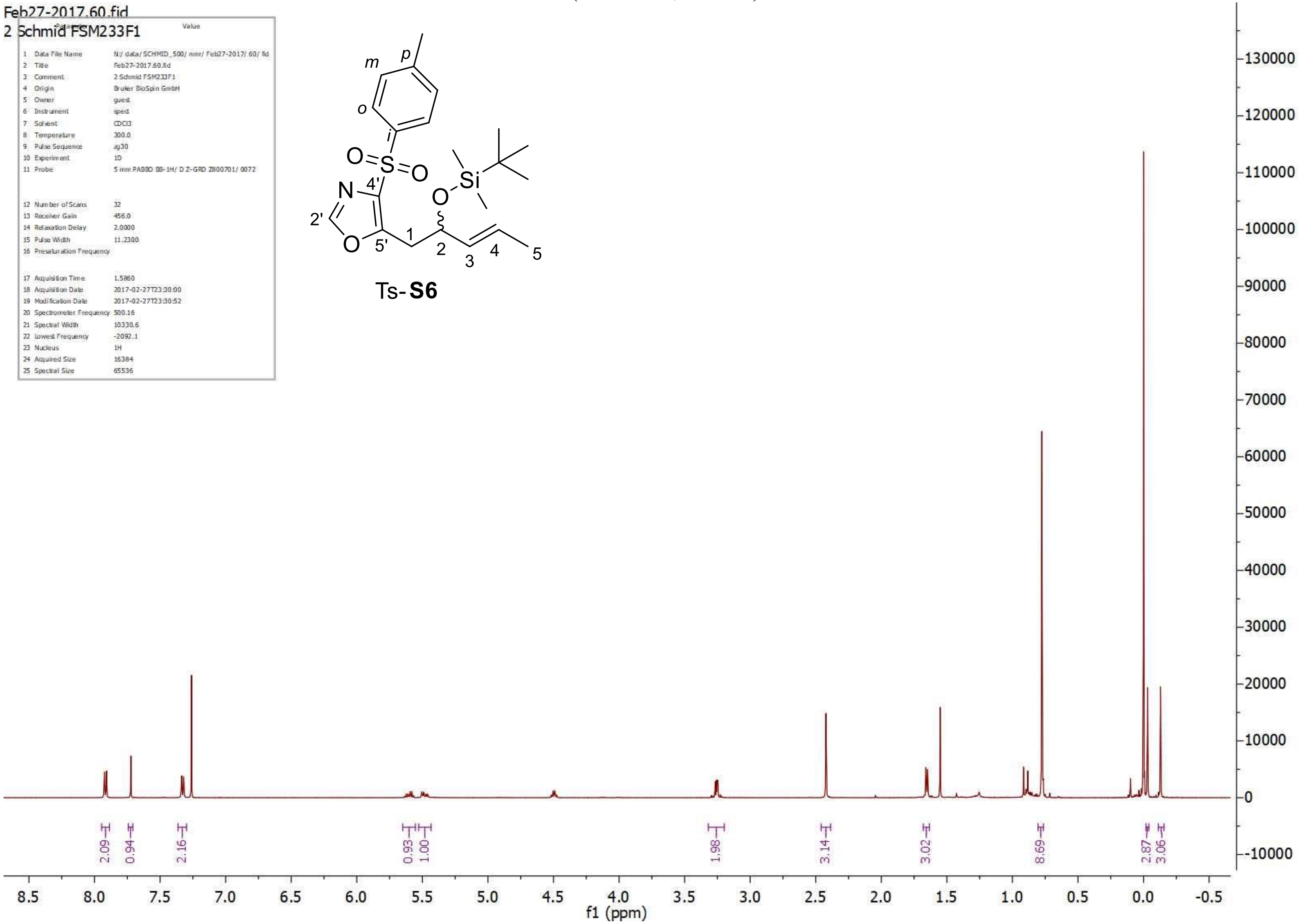

^{13}**C NMR** (125 MHz, $CDCl_3$) of Ts-**S6**

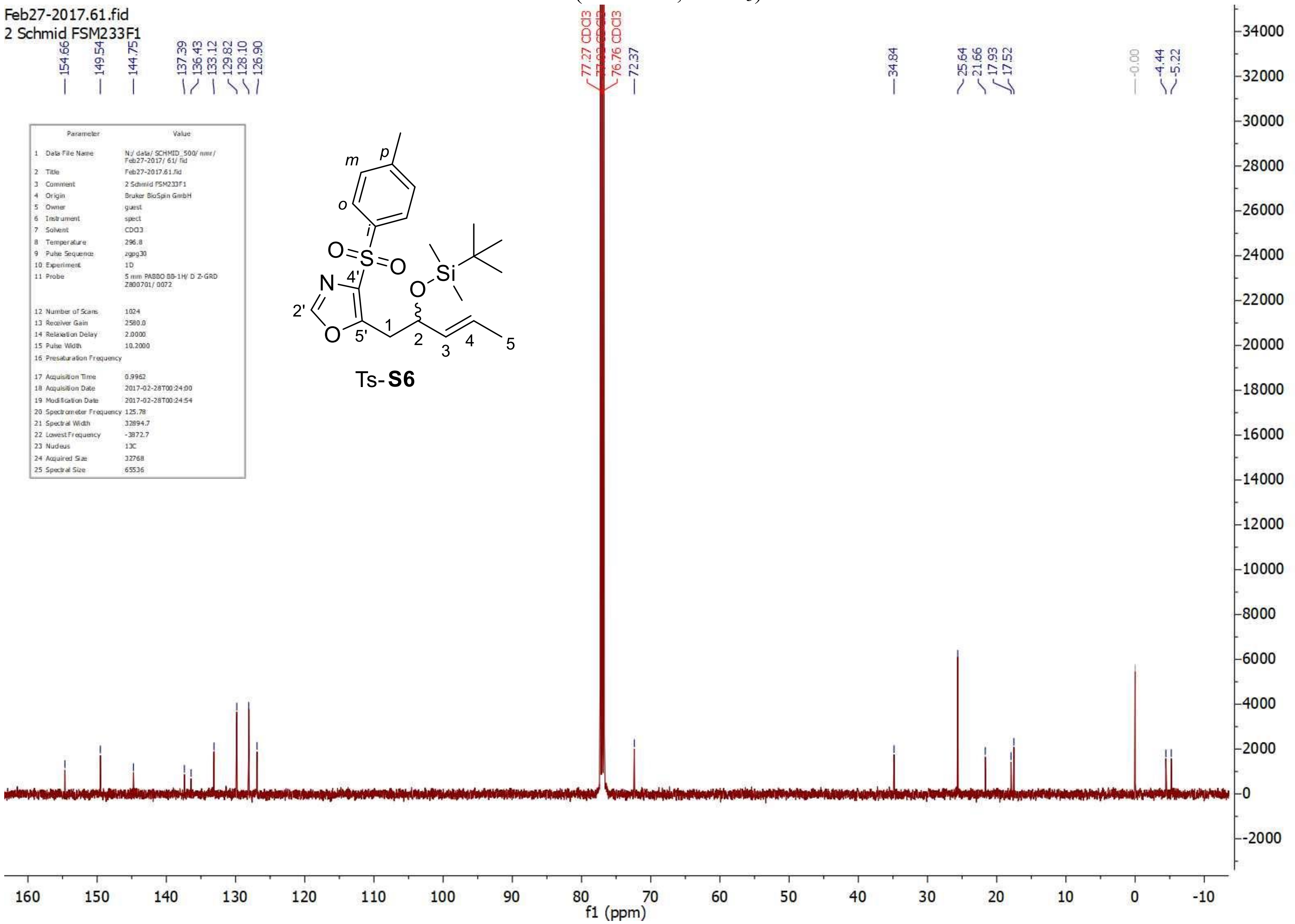

^{1}H NMR (500 MHz, $CDCl_3$) of S6

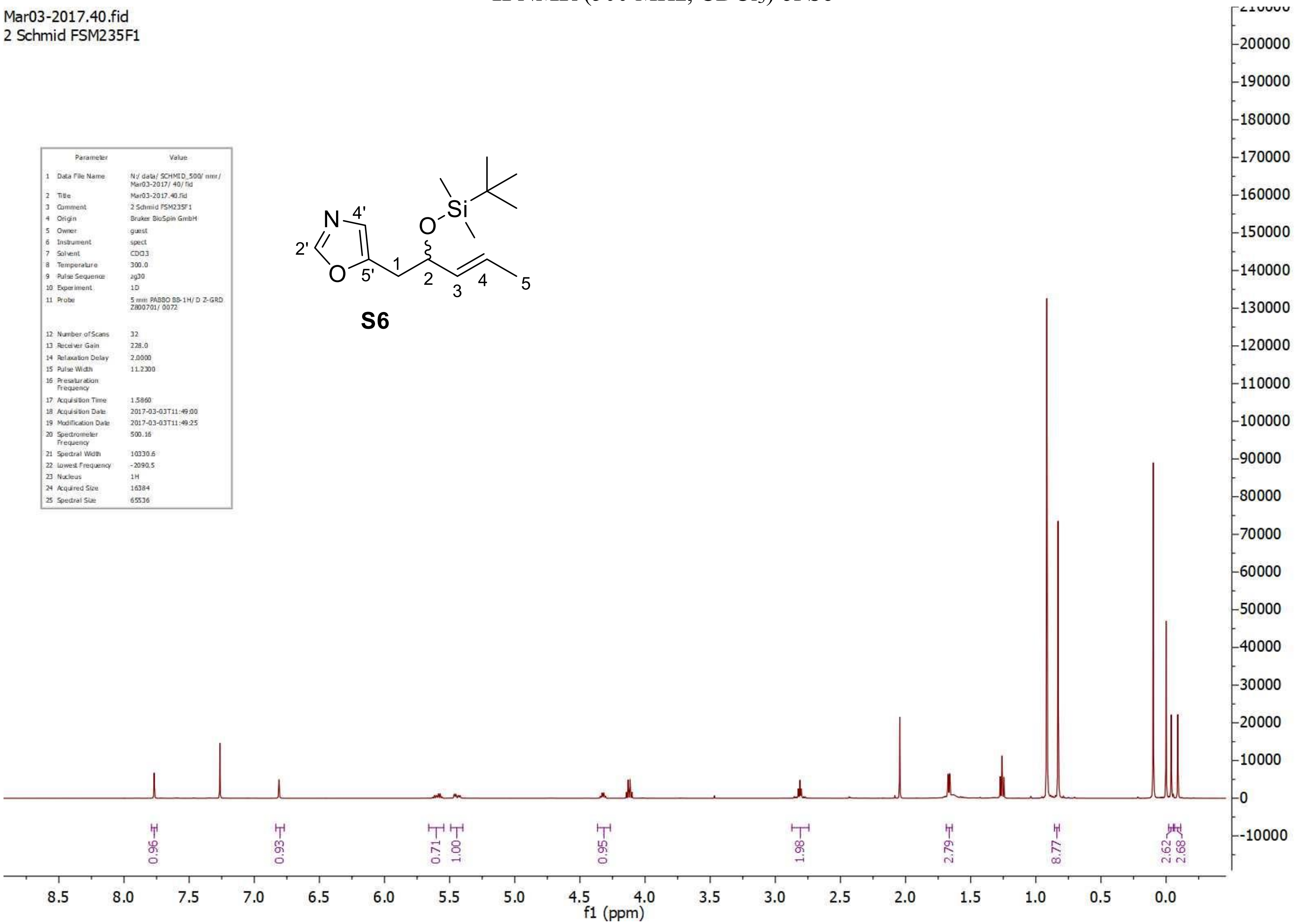

	Parameter	Value
1	Data File Name	N:/ data/ SCHMID_500/ nmr/ Mar03-2017/ 40/ fid
2	Title	Mar03-2017.40.fid
3	Comment	2 Schmid FSM235F1
4	Origin	Bruker BioSpin GmbH
5	Owner	guest
6	Instrument	spect
7	Solvent	CDCl3
8	Temperature	300.0
9	Pulse Sequence	zg30
10	Experiment	1D
11	Probe	5 mm PABBO BB-1H/ D Z-GRD Z800701/ 0072
12	Number of Scans	32
13	Receiver Gain	228.0
14	Relaxation Delay	2.0000
15	Pulse Width	11.2300
16	Presaturation Frequency	
17	Acquisition Time	1.5860
18	Acquisition Date	2017-03-03T11:49:00
19	Modification Date	2017-03-03T11:49:25
20	Spectrometer Frequency	500.16
21	Spectral Width	10330.6
22	Lowest Frequency	-2090.5
23	Nucleus	1H
24	Acquired Size	16384
25	Spectral Size	65536

^{13}**C NMR** (125 MHz, $CDCl_3$) of **S6**

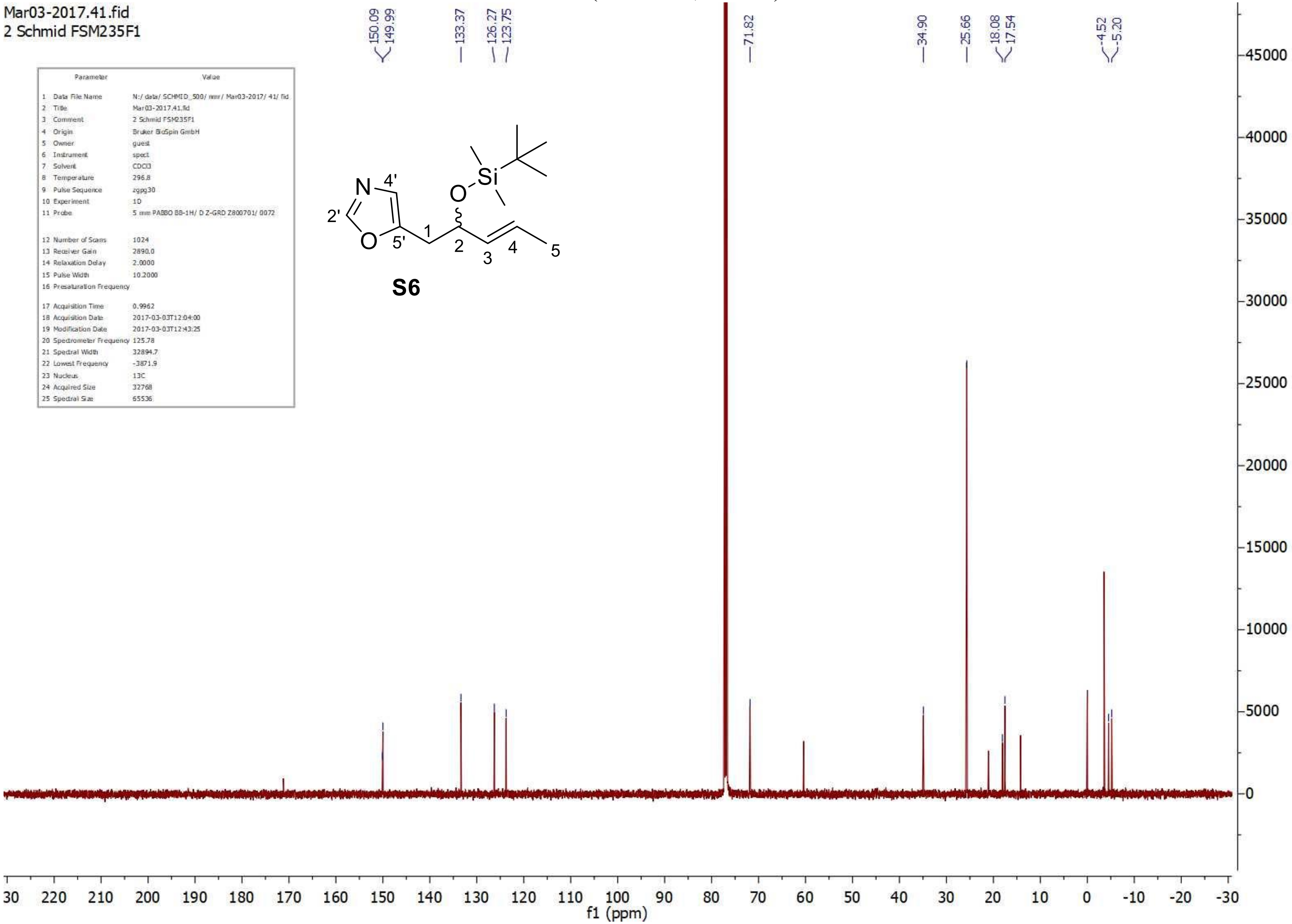

^{1}H NMR (400 MHz, $CDCl_3$) of Ts-**S15**

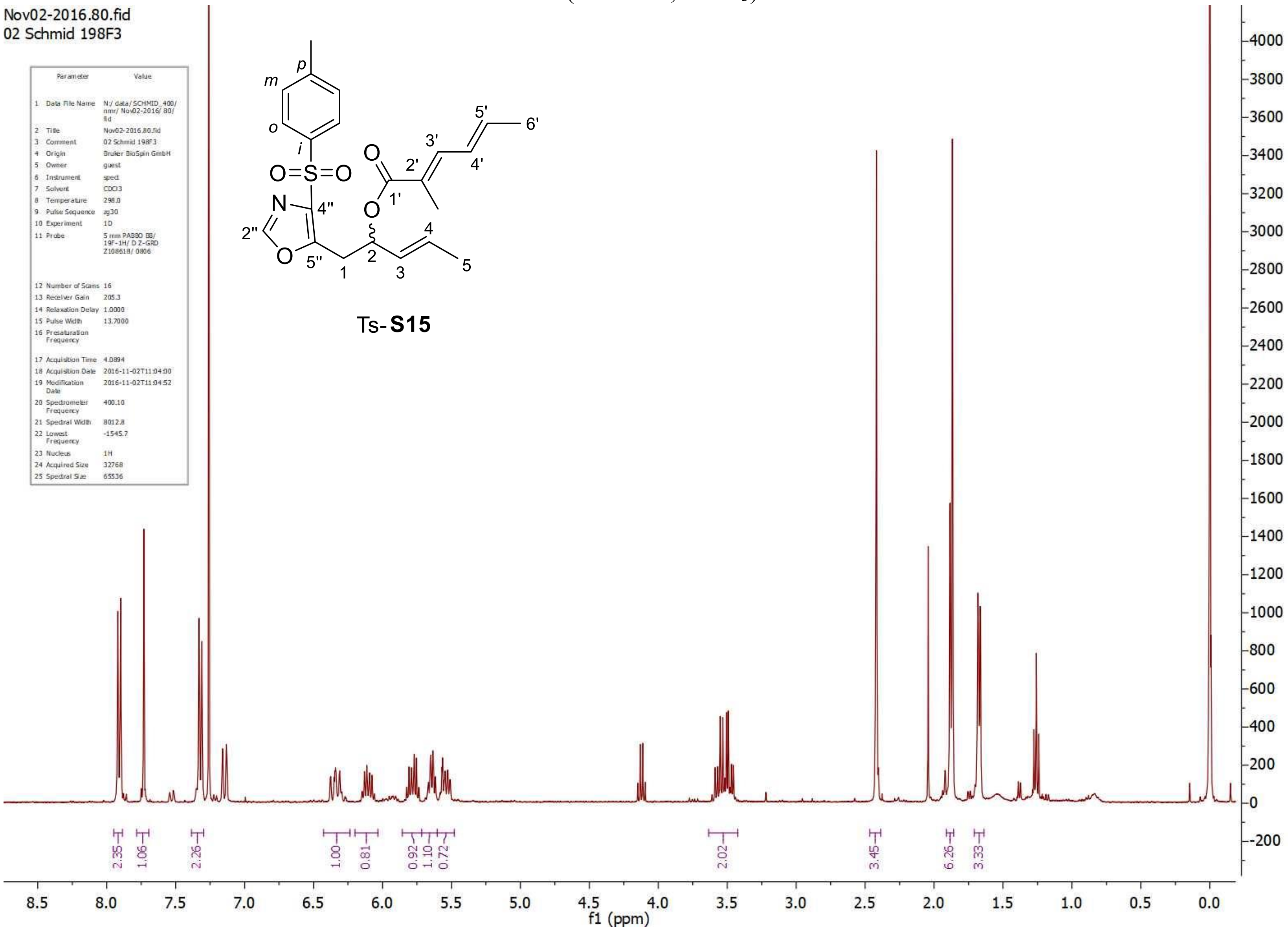

^{13}**C NMR** (100 MHz, $CDCl_3$) of Ts-**S15**

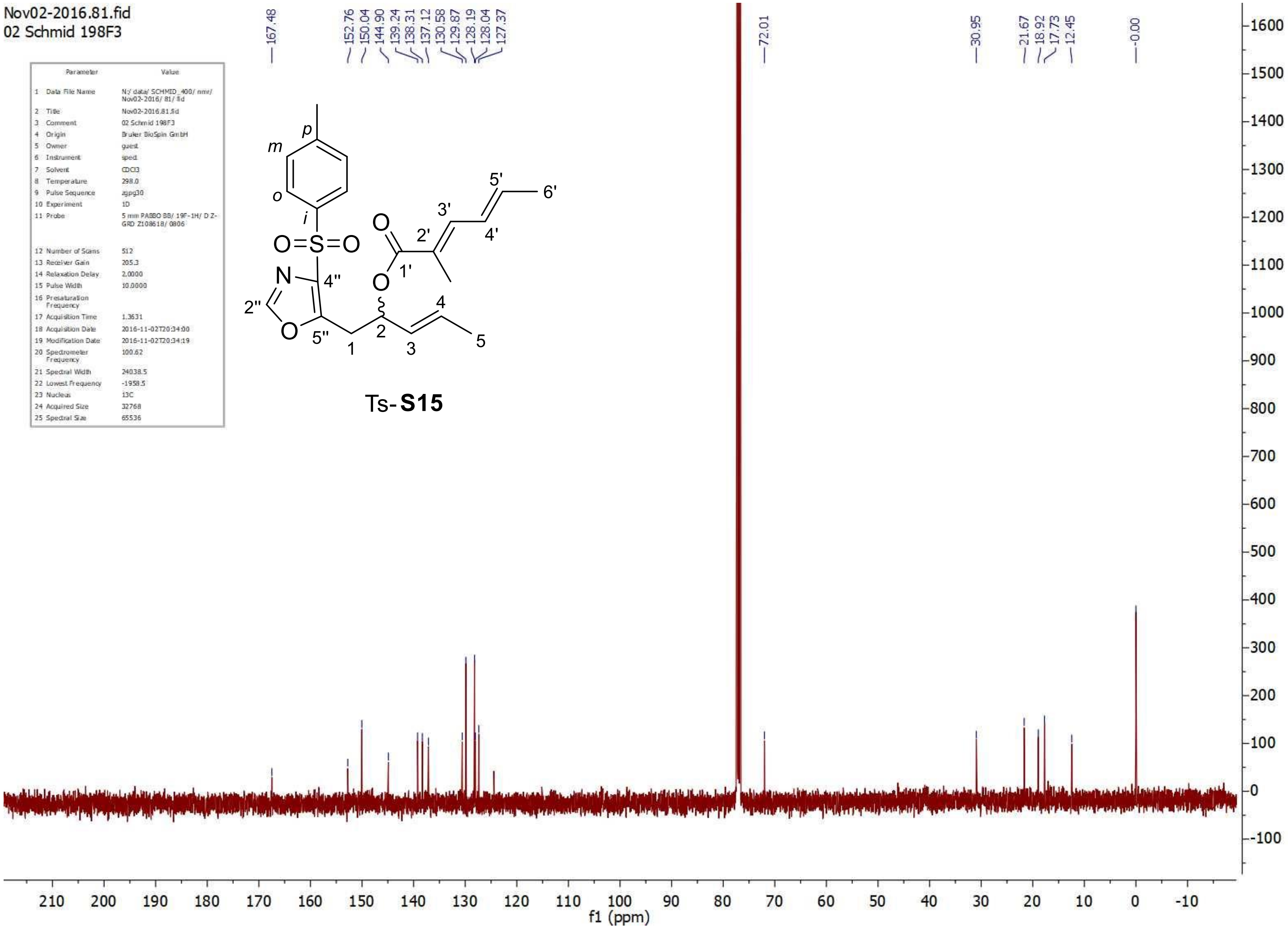

^{1}H NMR (500 MHz, $CDCl_3$) of *anti*-**S4**

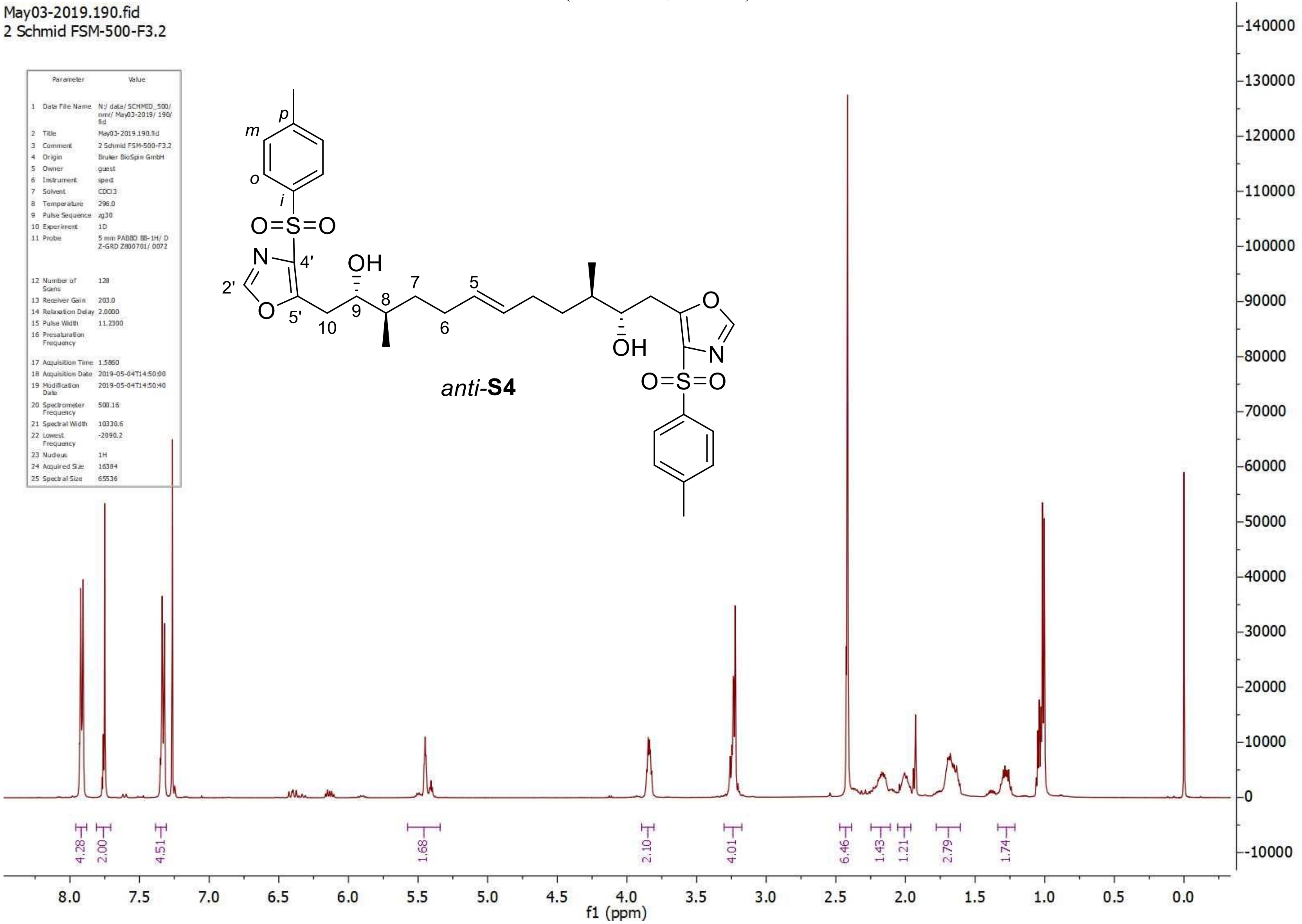

^{13}C NMR (125 MHz, $CDCl_3$) of *anti*-**S4**

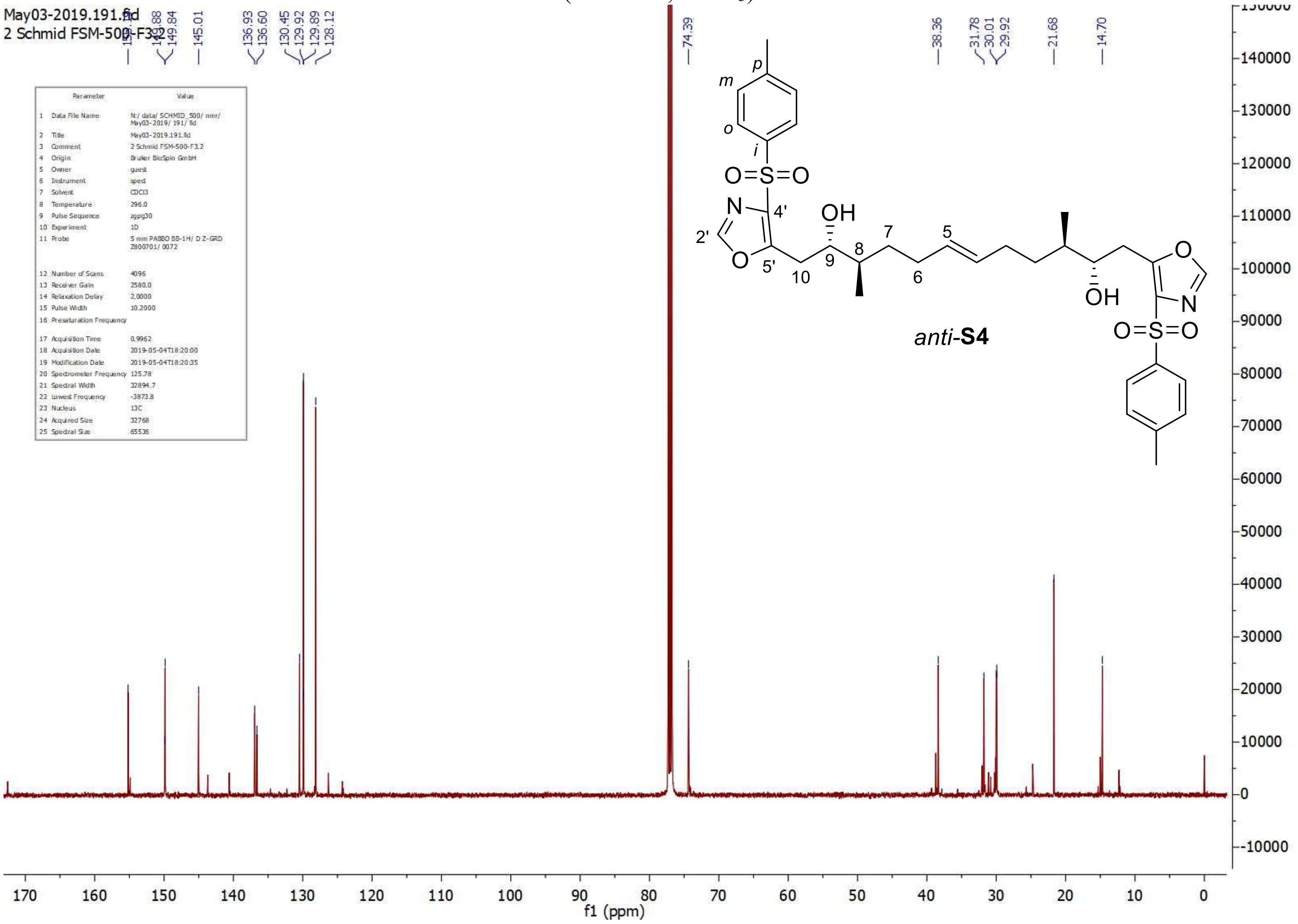

¹H NMR (500 MHz, $CDCl_3$) of *(R)*-**S1**

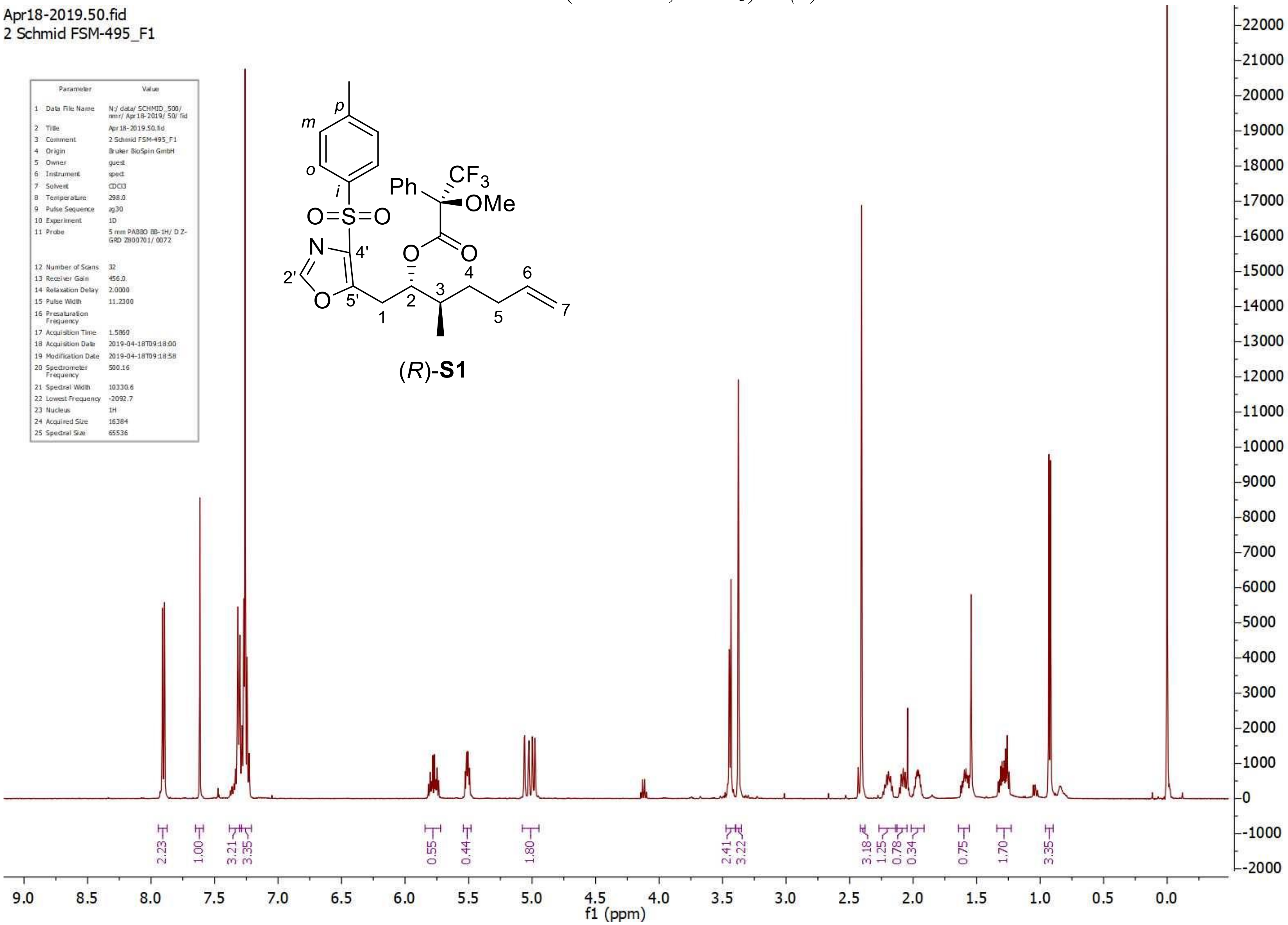

^{13}C NMR (125 MHz, $CDCl_3$) of *(R)*-**S1**

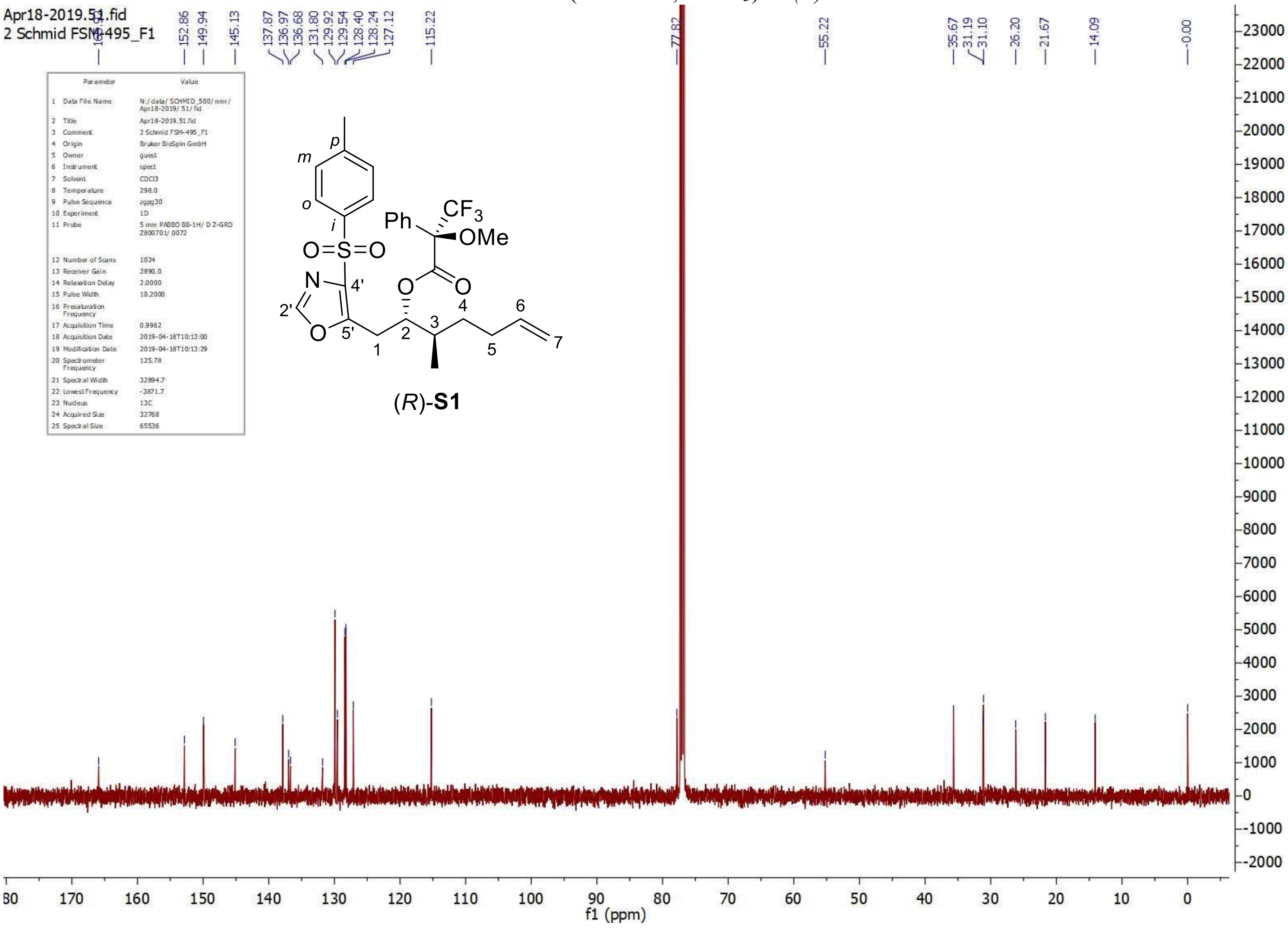

^{1}H NMR (500 MHz, CDCl$_3$) of *(S)*-**S1**

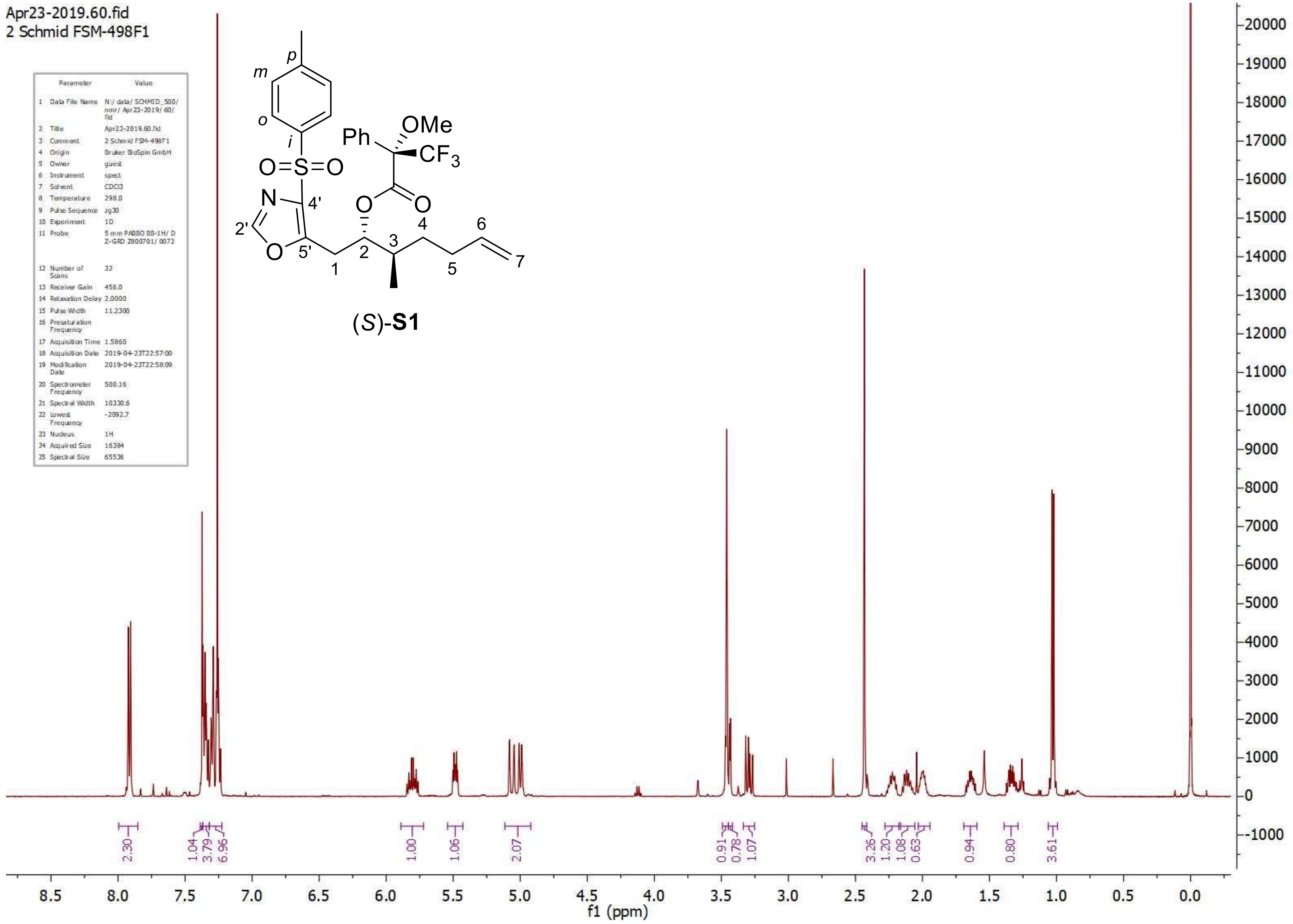

^{13}C NMR (125 MHz, CDCl$_3$) of *(S)*-**S1**

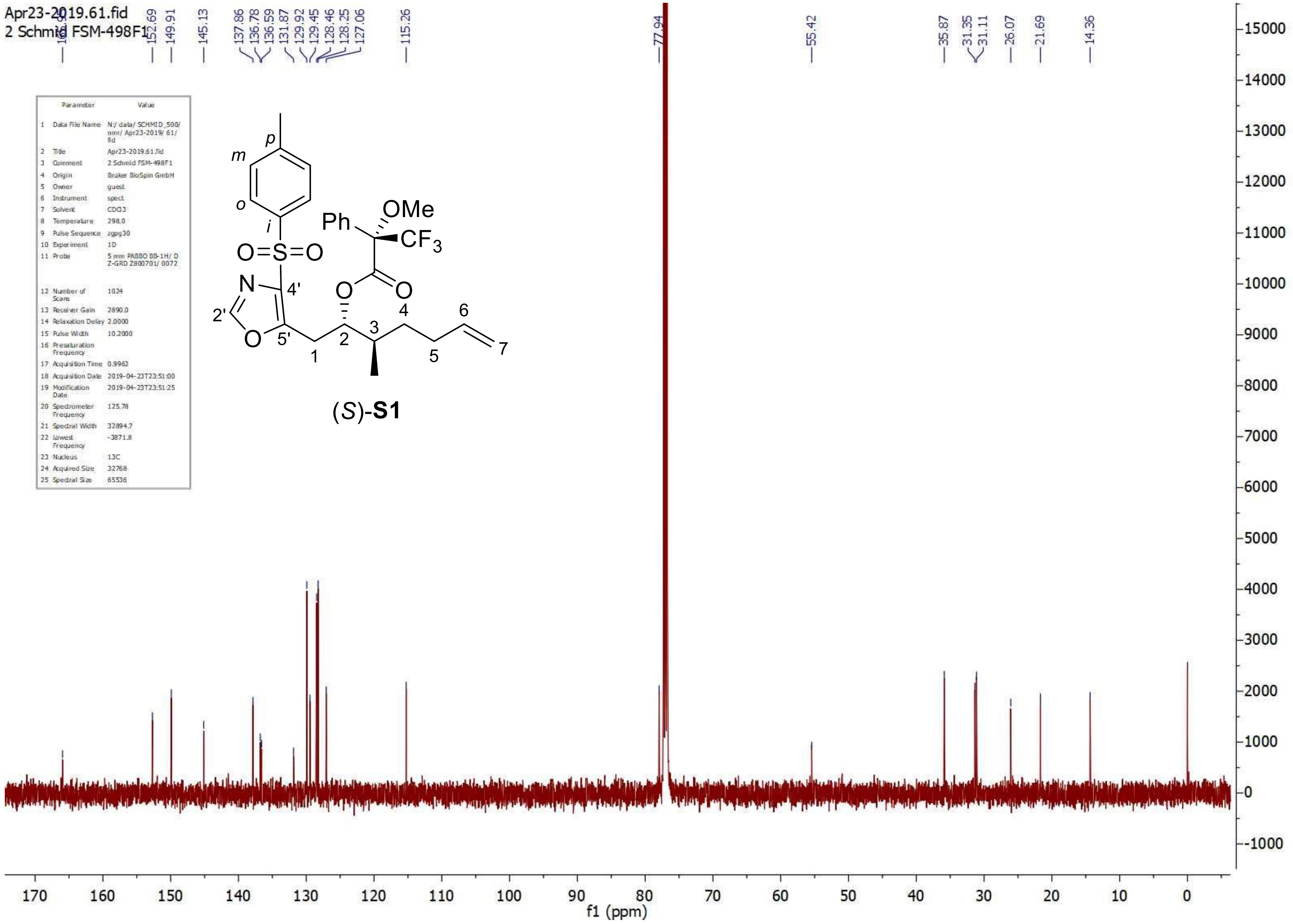

¹H NMR (400 MHz, $CDCl_3$) of the isolated natural samroiytomycin A *(syn,syn*-**4a**)

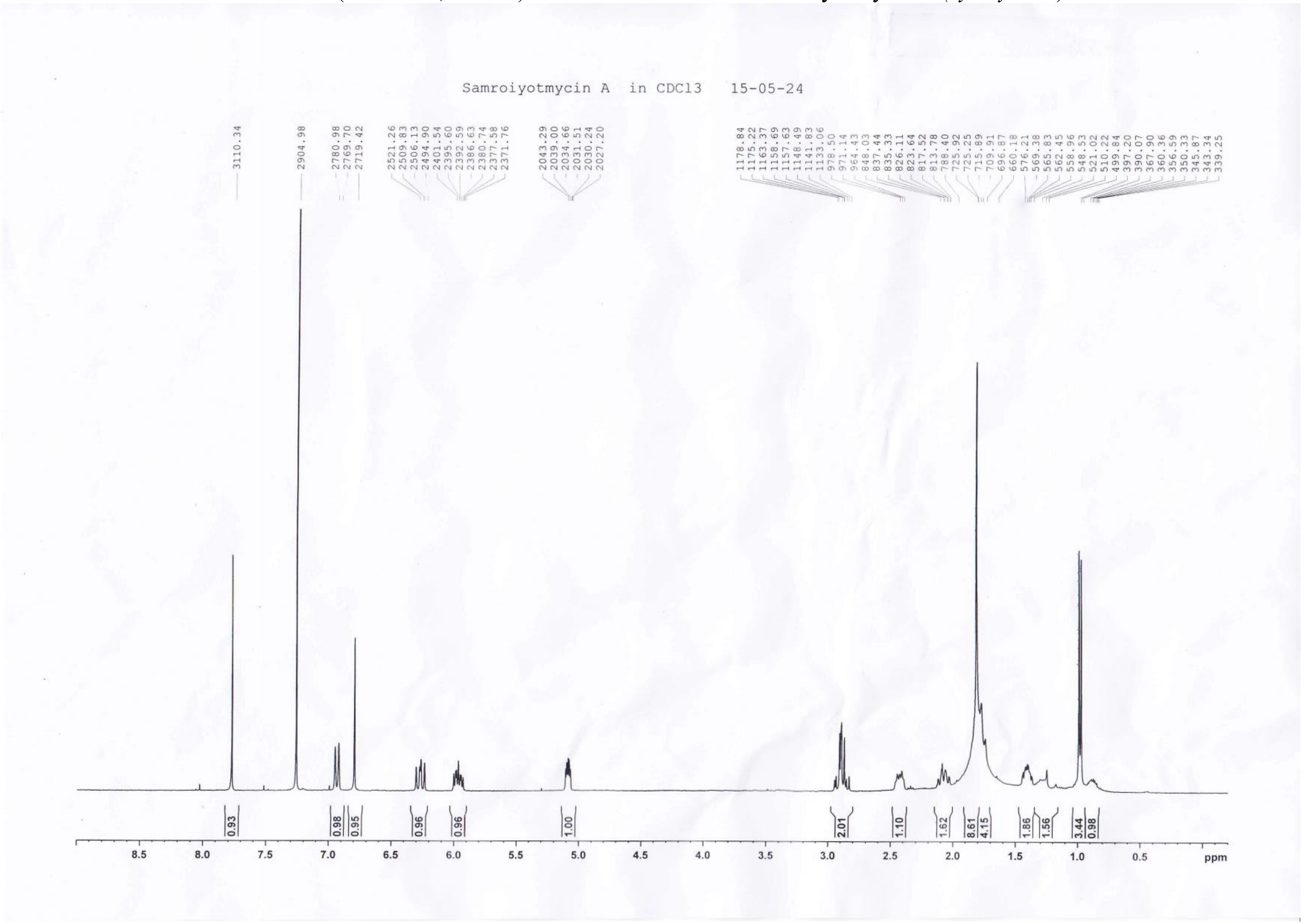

Supplement: Supplementary file 1 — Supporting Information [file CHEM-30-e202403408-s001.pdf]
